# Ruthenium-mediated nucleophilic aromatic substitution of hydrogen in benzene†

**DOI:** 10.1039/d5sc02090e

**Published:** 2025-06-16

**Authors:** Stanislav Melnikov, Donghun Hwang, Philip Gabbert, Bohyun Park, Martin Lutz, Mu-Hyun Baik, Daniël L. J. Broere

**Affiliations:** a Organic Chemistry and Catalysis, Institute for Sustainable and Circular Chemistry, Faculty of Science, Utrecht University Universiteitsweg 99 3584 CG Utrecht The Netherlands d.l.j.broere@uu.nl; b Department of Chemistry, Korea Advanced Institute of Science and Technology (KAIST) Daejeon 34141 Republic of Korea mbaik2805@kaist.ac.kr; c Center for Catalytic Hydrocarbon Functionalizations, Institute for Basic Science (IBS) Daejeon 34141 Republic of Korea; d Structural Biochemistry, Bijvoet Centre for Biomolecular Research, Faculty of Science, Utrecht University Universiteitsweg 99 Utrecht The Netherlands

## Abstract

The direct functionalization of unactivated hydrocarbons remains a significant challenge in modern chemistry. In this study, we demonstrate that a simple ruthenium complex featuring a chelating ^*t*Bu^PN ligand can mediate the nucleophilic aromatic substitution of hydrogen (S_N_ArH) in benzene. Key intermediates were kinetically trapped in low-temperature NMR experiments, providing crucial insights into the reaction mechanism. These findings are further supported by isotopic labeling and comprehensive DFT studies. The data shows that the substitution proceeds *via* an unprecedented mechanism, involving reversible rear-side nucleophilic addition of the exogenous nucleophile to the ruthenium-bound benzene, followed by an intramolecular hydride migration facilitated by deprotonation of ^*t*Bu^PN ligand. The broad range of nucleophiles amenable to this reaction, including classical non-nucleophilic bases, showcases the versatility of this reaction and makes it a promising candidate for further developments in the area of S_N_ArH.

## Abstract 2

The first example of oxidant-free SNArH reaction on benzene that is mediated by a piano-stool transition metal complex.

## Graphical abstract

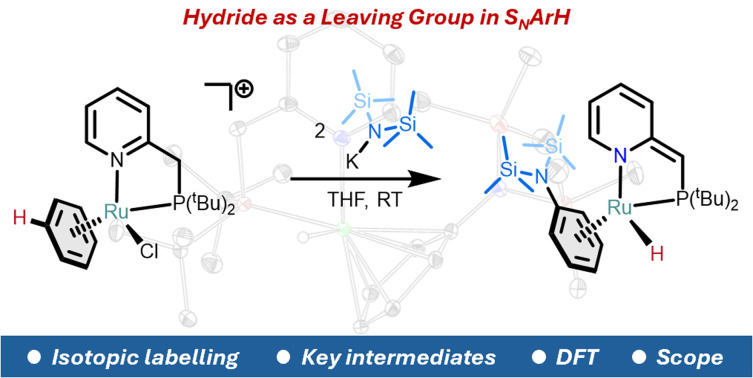

## Introduction

**Fig. 1:**
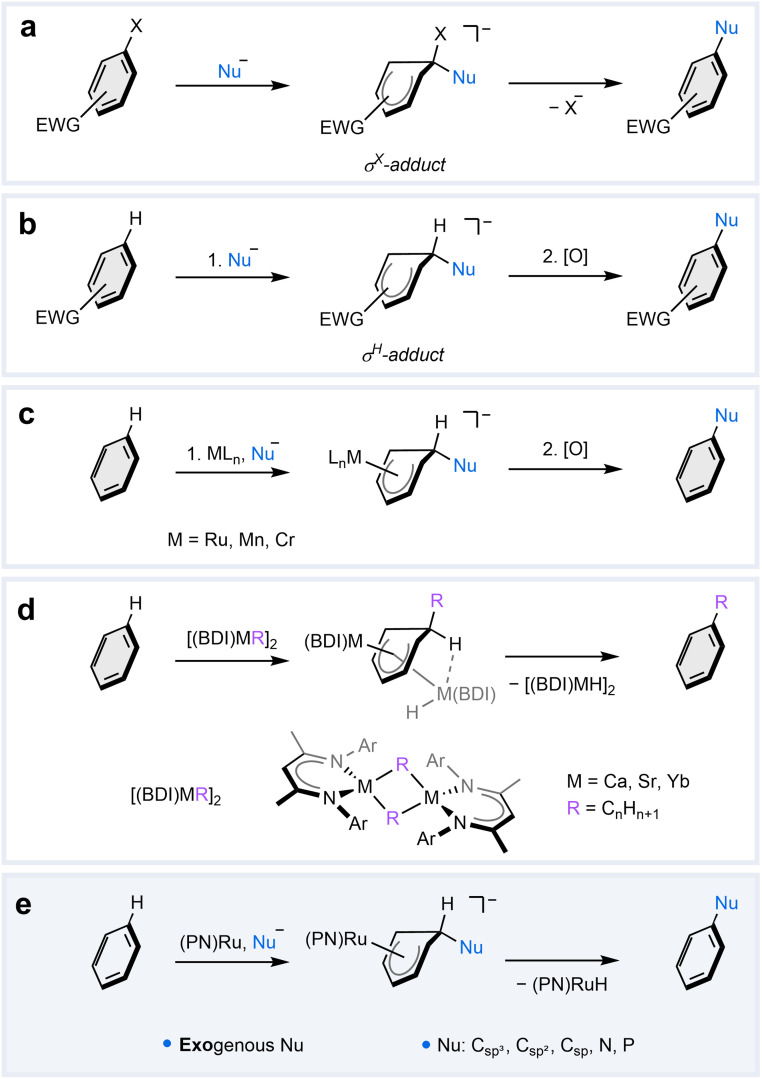
Strategies for nucleophilic aromatic substitution. (a) S_N_Ar in electron-deficient arenes through initial formation of a σ^X^-adduct followed by elimination of a leaving group (X). (b) Nucleophilic addition to electron-deficient arenes to give a σ^H^-adduct, which can be oxidized in a subsequent reaction to realize a formal S_N_ArH. (c) Nucleophilic addition to in transition metal arene complexes resulting in the formation of metal-stabilized σ^H^-adducts, which can be oxidized to mediate a formal S_N_ArH reaction. (d) S_N_ArH alkylation of benzene with β-diketiminate (BDI) complexes of Ca,^29^ Sr,^30^ and Yb,^31^ which employ *endogenous* nucleophiles; no oxidation step is required. (e) This work: metal-mediated S_N_ArH in benzene with a readily accessible ^*t*Bu^PN–Ru complex and various types of *exogenous* nucleophiles; no oxidation step is required.

Nucleophilic aromatic substitution (S_N_Ar) reactions are widely employed in both academic and industrial settings to structurally modify aromatic ring scaffolds.^1–5^ While the electron-rich π-system in aromatic molecules typically makes them react as nucleophiles in substitution reactions,^6–8^ several strategies have been developed to render them susceptible to nucleophilic attack.^1,9–12^ The most common strategy involves functionalization of the aromatic ring with electron-withdrawing groups (EWGs), which reduce the electron density on the arene. This facilitates nucleophilic addition at the position bearing a leaving group, such as F or Cl, leading to the formation of a σ^X^-adduct (Fig. 1a), also known as the Jackson–Meisenheimer complex. For benzene derivatives, this step is generally rate-determining due to the loss of aromaticity, which is restored upon subsequent extrusion of the leaving group.^1,9^ Although the addition of nucleophiles is kinetically more facile in positions occupied by hydrogen to give a σ^H^-adduct (Fig. 1b), the nucleophilic aromatic substitution of hydrogen (S_N_ArH) is prohibited by unfavorable elimination of a hydride anion.^1,2^ Nevertheless, formal S_N_ArH reactions have been realized in a two-step procedure involving a separate oxidation of the pre-formed σ^H^-adduct or through incorporation of a leaving group in the nucleophile.^12–15^

An alternative strategy that circumvents the functionalization of the aromatic ring with EWGs involves the coordination of arenes to transition metal complexes. Nucleophilic addition to the metal-bound arene yields stable cyclohexadienyl complexes, which are considered metal-stabilized analogs of the σ^H^-adducts. These reactions have been extensively investigated, particularly with Cr, Mn, and Ru complexes.^16–28^ However, to realize the substitution reaction, this strategy still requires subsequent oxidation of the metal-stabilized σ^H^-complex (Fig. 1c) or a conventional leaving group on the arene.

The first example of a metal-mediated S_N_ArH reaction that requires neither EWGs nor leaving groups on the arene was reported in 2017 by Hill, Maron, and co-workers. They found that dimeric β-diketiminate (BDI) supported organocalcium nucleophiles are capable of the direct nucleophilic alkylation of benzene.^29^ Subsequently, similar reactivity has been demonstrated by BDI-supported strontium^30^ and ytterbium^31^ complexes. Recent computational studies on the Ca and Sr complexes suggest that the mechanism involves an intramolecular front-side nucleophilic addition of the alkyl to the metal-coordinated arene (Fig. 1d).^32^ Subsequently, a second metal complex performs a rear-side hydride abstraction in an intermolecular manner. Although this process allows for oxidant-free S_N_ArH, it has thus far been limited to alkylation due to the required pre-formation of *endogenous* metal-bound nucleophiles. Consequently, the potential for other types of nucleophiles in metal-mediated S_N_ArH reactions remains unclear.

Herein, we report a new metal-mediated pathway for S_N_ArH in benzene with *exogenous* nucleophiles (Fig. 1e). This distinct reactivity is enabled by a simple ruthenium complex, which not only activates the arene towards nucleophilic attack but also mediates a unique intramolecular hydride transfer that completes the S_N_ArH reaction. In this article, we elucidate the underlying mechanism of this unusual reaction through a combination of isotopic labeling, kinetic trapping of key intermediates, and computational studies. Finally, we demonstrate the versatility of this reaction, highlighting its compatibility with a range of exogenous nucleophiles.

## Results and discussion

### Synthesis and characterization

**Fig. 2:**
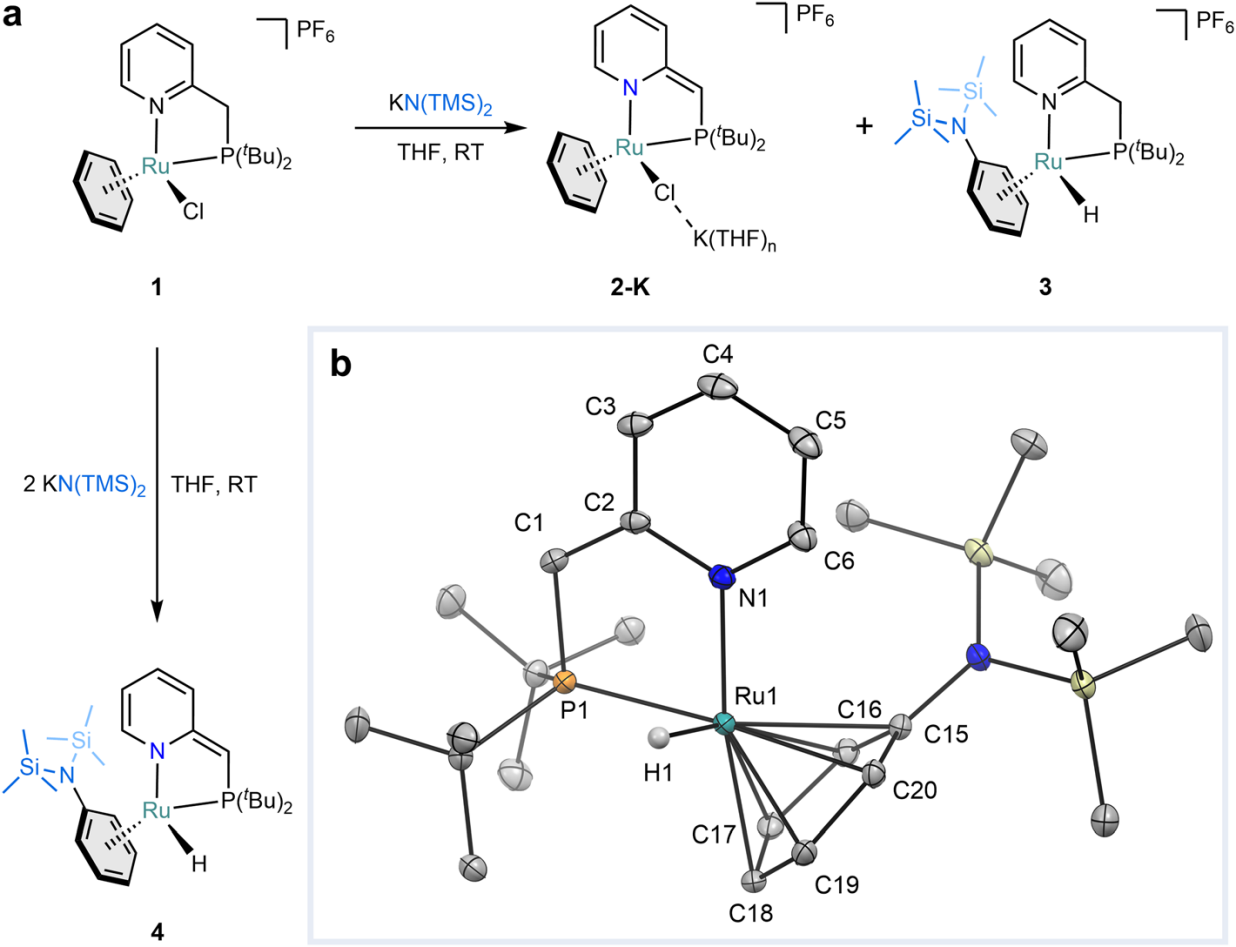
Substitution of a hydride at a coordinated benzene with KN(TMS)_2_. (a) The reaction between 1 and KN(TMS)_2_. The addition of 1 equiv. of the base leads to the mixture of 2-K and 3 (70 : 30), whereas 2 equiv. yield compound 4. (b) Solid-state molecular structure of 4 (ellipsoids drawn at the 30% probability level). The hydrogen atoms, except the hydride, are omitted for clarity. Selected bonds (Å) and angles (°): Ru(1)–PhN(TMS)^centroid^_2_ = 1.7821(7), Ru(1)–N(1) = 2.0961(11), Ru(1)–P(1) = 2.3325(3), Ru(1)–C(15) = 2.4275(13), Ru(1)–C(16) = 2.3775(14), Ru(1)–C(17) = 2.2091(14), Ru(1)–C(18) = 2.2055(14), Ru(1)–C(19) = 2.1776(14), Ru(1)–C(20) = 2.2409(13), C(1)–C(2) = 1.382(2), C(2)–N(1) = 1.3910(18), C(2)–C(3) = 1.4415(19), C(3)–C(4) = 1.359(2), C(4)–C(5) = 1.407(2), C(5)–C(6) = 1.370(2), N(1)–C(6) = 1.3555(18), N(1)–Ru(1)–P(1) = 81.75(3), N(1)–Ru(1)–PhN(TMS)^centroid^_2_ = 131.28(4), P(1)–Ru(1)–PhN(TMS)^centroid^_2_ = 140.17(3).

While exploring Milstein-like aromatization–dearomatization cooperativity^33^ with complex [(^*t*Bu^PN)RuCl(C_6_H_6_)]PF_6_ (1, where ^*t*Bu^PN is 2-((di-*tert*-butylphosphaneyl)methyl)pyridine), we discovered that the addition of 1 equiv. of KN(TMS)_2_ to 1 in THF at room temperature results in the formation of a mixture of two inseparable species in a 70 : 30 ratio (Fig. 2a). Analysis of the mixture by ^1^H nuclear magnetic resonance (NMR) spectroscopy in THF-*d*_8_ revealed that the major species featured all the characteristic resonances of the anticipated deprotonated compound [(^*t*Bu^PN*)RuCl(C_6_H_6_)K(THF)_*n*_]PF_6_ (2-K) (see ESI Section S1† for characterization). Surprisingly, the second species featured a doublet at *δ* = −7.77 ppm and two doublets of doublets at *δ* = 3.63 and 3.26 ppm, all integrating to ^1^H. These distinct resonances are characteristic of the presence of a hydride and diastereotopic methylene hydrogens in a non-deprotonated form of the ^*t*Bu^PN ligand, respectively. Additionally, this new species lacked a coordinated benzene molecule; instead, five separated multiplets were found in the ^1^H spectrum in the range from *δ* = 6.27 to 4.64 ppm. Based on the spectroscopic data we assign this species to [(^*t*Bu^PN)RuH(PhN(TMS)_2_)]PF_6_ (3, Fig. 2a). Intrigued by the formation of this unusual species, which we hypothesized to be the result of S_N_ArH at the coordinated benzene, we undertook a more detailed investigation into this surprising reactivity.

Adding complex 1 to 2 equiv. of KN(TMS)_2_ in THF at room temperature results in an immediate color change from yellow to brown. From this mixture (^*t*Bu^PN*)RuH(PhN(TMS)_2_) (4) was isolated in 97% yield (Fig. 2a). The ^1^H NMR spectrum of 4 in C_6_D_6_ shows a doublet at *δ* = −7.76 ppm and a doublet at *δ* = 3.49 ppm, which both integrate to ^1^H and are characteristic of a hydride and a methine linker of a dearomatized ^*t*Bu^PN ligand (^*t*Bu^PN*), respectively. Similar to 3, the ^1^H NMR spectrum of 4 also displays five magnetically coupled multiplets (all integrating to ^1^H) between *δ* = 5.11 and 4.14 ppm and a large singlet at 0.25 ppm, which integrates to 18H. From this data, we concluded that complex 4 is the deprotonated analog of 3. This is supported by the X-ray crystal structure determination (Fig. 2b, ESI Section S6.2†) of brown single crystals of 4 obtained from a concentrated pentane solution at −40 °C.

The solid-state structure of 4 revealed a piano-stool type complex in which ruthenium is bound to a silylated aniline, a ^*t*Bu^PN* ligand, and a hydride, which was located in difference Fourier map. The found interatomic distances in the ^*t*Bu^PN* ligand are characteristic of a dearomatized pyridine ring and a deprotonated methylene linker (Fig. 2b), which agrees with the NMR data. This anionic binding mode of the bidentate ligand enforces the small N(1)–C(2)–C(1)–P(1) dihedral angle of 2.10(19)°. The coordination sphere of the metal center is best described as a distorted piano-stool geometry with a very long Ru–aniline^centroid^ distance of 1.7821(7) Å (only 4% of reported η^6^-arene Ru structures have this distance longer according to the Cambridge Structural Database,^34^ see ESI Fig. S167–S169†). The ruthenium is bound unevenly to all the carbons of the aniline moiety, and a slight ring slippage is observed away from the N(TMS)_2_ side with respect to the coordinated metal (0.194 Å). It is noticeable that the Ru–C bonds are clustered into two groups. Two of the bonds are longer compared to the other four (Ru–C = 2.3775(14)–2.4275(13) Å for C(15)–C(16) *vs.* Ru–C = 2.1776(14)–2.2409(13) Å for C(17)–C(20), see ESI Tables S7 and S8†). This type of arene coordination can also be considered as ‘η^4^+η^2^’ and is associated with the *trans*-effect of the tertiary phosphine^35,36^ as well as steric repulsion emerged between PhN(TMS)_2_ fragment and ^*t*Bu^PN* ligand. This explains the relatively large separation of the aromatic aniline resonances in the ^1^H NMR spectrum.

### Mechanistic investigations

**Fig. 3:**
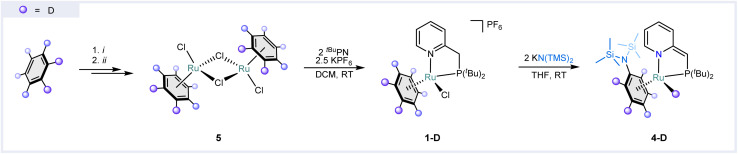
Isotopic labeling experiments. Synthetic route towards compound 4-D. The purple balls represent the positions where a significant amount of deuterium was detected by qNMR spectroscopy. The reaction conditions: (i) Li metal, ethylenediamine, EtOD in THF at 0 °C; (ii) RuCl_3_·*x*H_2_O in EtOD (reflux). For more details on isotopic labeling see ESI Section S1.†

The reactivity observed for complex 1 is particularly remarkable given that KN(TMS)_2_ is typically regarded as a non-nucleophilic base, while benzene is typically inert to nucleophilic aromatic substitution. Hence, we set out to elucidate the underlying mechanism of this reaction. To ascertain the origin of the hydride in 4, we synthesized an isotopically labeled analog, 4-D (Fig. 3). For that, benzene-*d*_6_ was reduced with lithium metal and ethylenediamine in Et_2_O/EtOD according to a modified birch-like procedure,^37^ which gave a mixture of partially deuterated cyclohexadienes. A subsequent reaction with ruthenium trichloride gave partially deuterated Ru_2_Cl_4_(benzene)_2_ (5), which was used to synthesize 1-D with 61% deuteration of the coordinated benzene. Reacting 1-D with two equiv. of KN(TMS)_2_ yielded compound 4-D with 57% and 56% deuteration for the coordinated aniline and hydride, respectively. The relative integration of the signals in ^1^H NMR spectra of compound 4-D shows an equal degree of deuteration between the hydridic and aromatic resonances, confirming that the hydride originates from the benzene (ESI Fig. S45 and S46†). It is noteworthy that no H/D scrambling with the ^*t*Bu^PN ligand was observed in the product, excluding arene C–H bond activation *via* metal–ligand cooperation.^38,39^

**Fig. 4:**
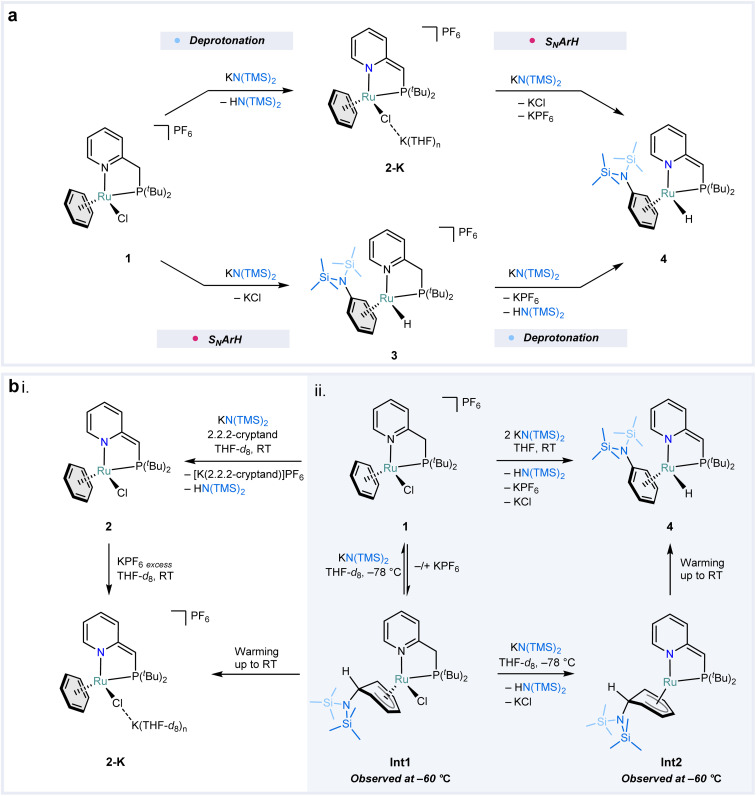
Plausible mechanisms and mechanistic investigations of the S_N_ArH reaction with KN(TMS)_2_. (a) Plausible mechanistic pathway involving first deprotonation of the methylene linker of the ^*t*Bu^PN ligand followed by nucleophilic aromatic substitution of a hydride (top path) and a pathway wherein the S_N_ArH precedes the deprotonation of the ^*t*Bu^PN ligand (bottom path). (b) Mechanistic NMR experiments at RT (i) and −60 °C (ii) featuring the structures of two kinetically-trapped metal-stabilized Jackson–Meisenheimer intermediates Int1 and Int2.

Based on the isotopic labeling experiments, two plausible mechanistic pathways were hypothesized for the substitution reaction: (i) deprotonation of ^*t*Bu^PN with subsequent aromatic substitution of the hydride (Fig. 4a top) and (ii) aromatic substitution followed by deprotonation of the ligand (Fig. 4a bottom). To shed light on the operational mechanism, a series of low-temperature NMR experiments were performed involving the reaction between 1 and KN(TMS)_2_. After the addition of only 1 equiv. of KN(TMS)_2_ to 1 at −78 °C in THF-*d*_8_, ^1^H NMR analysis at −60 °C showed conversion to a single major species that neither contained a hydride nor a symmetrical benzene. The proton signals corresponding to the former aromatic ring of the benzene were found as six equally integrating multiplets in a wide range of *δ* = 5.10 to 2.76 ppm. Based on extensive 2D NMR analysis, we assign this compound as intermediate Int1 (Fig. 4b, ESI Fig. S66–S71†), which contains a η^5^-cyclohexadienyl ligand formed by nucleophilic addition of N(TMS)_2_^−^ to the coordinated benzene, resembling the Jackson–Meisenheimer complex. The extremely upfield-shifted proton signal at *δ* = 2.76 ppm corresponds to the allylic proton of the cyclohexadienyl fragment. Surprisingly, the methylene linker of the complex is intact despite the addition of such a potent base as KN(TMS)_2_. Slow warming up of the reaction mixture to RT results in the decomposition of Int1 into a mixture of species that converted to predominantly complex 2-K after 16 hours at RT (ESI Fig. S72†). This shows that the nucleophilic addition that forms Int1 is a kinetically driven process, contrarily to the deprotonation of the ligand that leads to 2-K, which is a more thermodynamically favorable pathway. Moreover, it also shows that the nucleophilic addition is reversible (Fig. 4b).

^1^H NMR analysis at −60 °C of a mixture resulting from the addition of 2 equiv. of KN(TMS)_2_ to 1 in THF-*d*_8_ at −78 °C allowed us to observe a new species Int2. Similar to the analogous experiment with 1 equiv. of KN(TMS)_2_ that gave Int1, no resonances in the hydridic region were detected, and seven equally integrating resonances within *δ* = 5.28 and 3.10 ppm were found. Together with ^1^H–^31^P HMBC analysis, a broad signal at *δ* = 3.25 ppm was assigned as a methine proton, suggesting deprotonation of the ^*t*Bu^PN ligand (Fig. 4b, ESI Fig. S73–S78†). The other six signals were magnetically coupled, as shown by ^1^H–^1^H TOCSY NMR analysis, and were assigned as η^5^-cyclohexadienyl protons. From this data, we conclude that compound Int2 is another metal-stabilized Jackson–Meisenheimer intermediate, which forms upon the deprotonation of Int1. Warming up the reaction mixture to RT resulted in a clean formation of compound 4 already at −20 °C, supporting our findings regarding the nature of Int2 (ESI Fig. S79–S82†). The fact that compound 4 forms cleanly only upon warming up demonstrates that the hydride migration is the rate-determining step of the reaction from 1 to 4.

As noted above, nucleophilic addition to transition metal complexes has been well established since the 1960s. However, metal-stabilized Jackson–Meisenheimer intermediates are typically very stable and do not undergo hydride migration to the metal center. To gain insight into the role of the PN ligand in this unusual reactivity, we conducted a comparative study using a series of alternative ligands (see ESI, Section S2.3†). Specifically, we synthesized five analogous complexes featuring bidentate ligands with varying electronic and steric properties: 2-((di-*tert*-butylphosphaneyl)oxy)pyridine (PON), *N*-ethyl-*N*-(pyridin-2-ylmethyl)ethanamine (NN), 4,4′-di-*tert*-butyl-2,2′-bipyridine (^*t*^Bu-bpy), 1,10-phenanthroline (phen), N1,N1,N2,N2-tetramethylethane-1,2-diamine (TMEDA). Remarkably, upon reacting them in an analogous manner to complex 1 with KN(TMS)_2_, only the reaction with [(PON)RuCl(C_6_H_6_)]PF_6_ led to the formation of the corresponding hydride complex [(PON)RuH(C_6_H_5_N(TMS)_2_)]PF_6_, which displayed similar spectral features as complex 3. The fact that the other complexes showed no reactivity toward nucleophilic aromatic substitution, underscores the critical role of ligand design in enabling S_N_ArH reactivity. Furthermore, the successful hydride migration observed with the PON ligand demonstrates that the ligand deprotonation observed in 4 is not vital for the Ru-mediated S_N_ArH.

### Computational studies

**Fig. 5:**
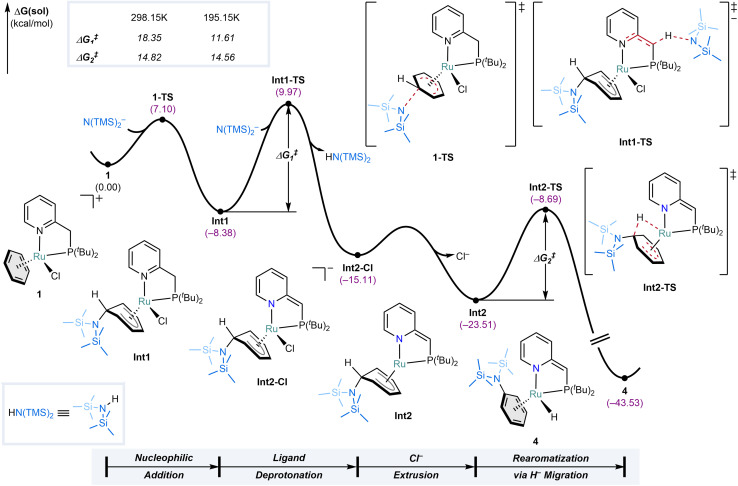
Computed reaction profile of the S_N_ArH mediated by complex 1. Only the most facile pathway is shown. For details and other calculated energy profiles, see ESI Section S3.1.†

To gain a deeper understanding of the mechanism of the S_N_ArH reaction, density functional theory (DFT) calculations were performed using the ORCA 5.0.3 software at the B3LYP-D3(BJ)/def2-TZVPD/def2-ECP(Ru)//B3LYP-D3(BJ)/def2-SVP/def2-ECP(Ru) level of theory (see Methods for details). Given the complexity and potential inaccuracies associated with modeling the solvated structure of the complex associated with K^+^ and THF,^40^ the N(TMS)_2_^−^ anion without its cationic partner was utilized to model the entire reaction. Both mechanistic scenarios depicted in Fig. 4a were analyzed – where nucleophilic addition to 1 (1-TS, Fig. 5) precedes deprotonation of the PN ligand or *vice versa* (1-TS′, ESI Fig. S123, see ESI Section S3 for more detail†). Consistent with the experimental results, the calculations show that the formation of metal-stabilized Jackson–Meisenheimer complex Int1*via*1-TS, featuring a barrier of 7.1 kcal mol^−1^, is more facile than the formation of deprotonated complex 2*via*1-TS′, which has a 3.0 kcal mol^−1^ higher barrier (for comparison of both pathways computed at RT see ESI Fig. S125†). The subsequent deprotonation of Int1 by a N(TMS)_2_^−^ anion forms Int2-Cl with a barrier of 18.4 kcal mol^−1^. This deprotonation results in the dearomatization of the PN ligand, enhancing the electron-donating ability of the nitrogen in the pyridine moiety and rendering the metal center more electron-rich. As such, it facilitates Cl^−^ dissociation to generate the required vacant site for hydride migration. Combined with the anionic nature of Int2-Cl, extrusion of Cl^−^ to give Int2 is more favorable compared to an alternative pathway where Cl^−^ extrusion occurs prior to PN ligand deprotonation (see ESI Fig. S124†). That said, our computations show that this alternative pathway is still feasible, and this is further underlined by the observation that the S_N_ArH is also possible with the analogous complex featuring the PON ligand that cannot dearomatize. The final step that completes the S_N_ArH reaction from Int2 to 4 traverses Int2-TS with a barrier of 14.5 kcal mol^−1^, and is best described as an intramolecular hydride migration. Aromatization of η^5^-cyclohexadienyl ligands has been proposed *via* indirect reductive elimination with metal hydrides^41^ and alkyls,^42^ but not *via* direct migration to the metal center. Notably, it was investigated for re-aromatization of a η^5^-cyclohexadienyl Rh complex, but calculations showed a far too high barrier of 49.9 kcal mol^−1^ for this to be feasible.^43^ Instead, a more favorable stepwise pathway involving ring slippage followed by allylic β-hydride elimination was found. This shows that the facile hydride transfer from Int2 to 4 features an exceptionally low barrier. Notably, based on the calculated reaction profile at 298.15 K, the barrier for the deprotonation step of Int1*via*Int1-TS (Δ*G*^‡^_1_ = 18.4 kcal mol^−1^) is higher than that of the intramolecular hydride migration *via*Int2-TS (Δ*G*^‡^_2_ = 14.8 kcal mol^−1^), which seems inconsistent with the observation of Int2 in low-temperature NMR experiments. However, when calculated at the reaction temperature at which the experiment was performed (195.15 K), the barrier for the deprotonation decreases to 11.6 kcal mol^−1^, while the barrier for the intramolecular hydride migration remains nearly unaffected between the two temperatures. This drop in the barrier of Int1-TS can be attributed to the diminished entropic penalty for the bimolecular process at lower temperatures. As a result, at 195.15 K, the deprotonation barrier becomes lower than that of the hydride migration (see ESI Fig. S126† for the reaction profiles computed at 195.15 K). These results corroborate the experimental observation of Int2 at low temperatures and its selective conversion into 4 upon warming to RT.

### Scope of the reaction

**Fig. 6:**
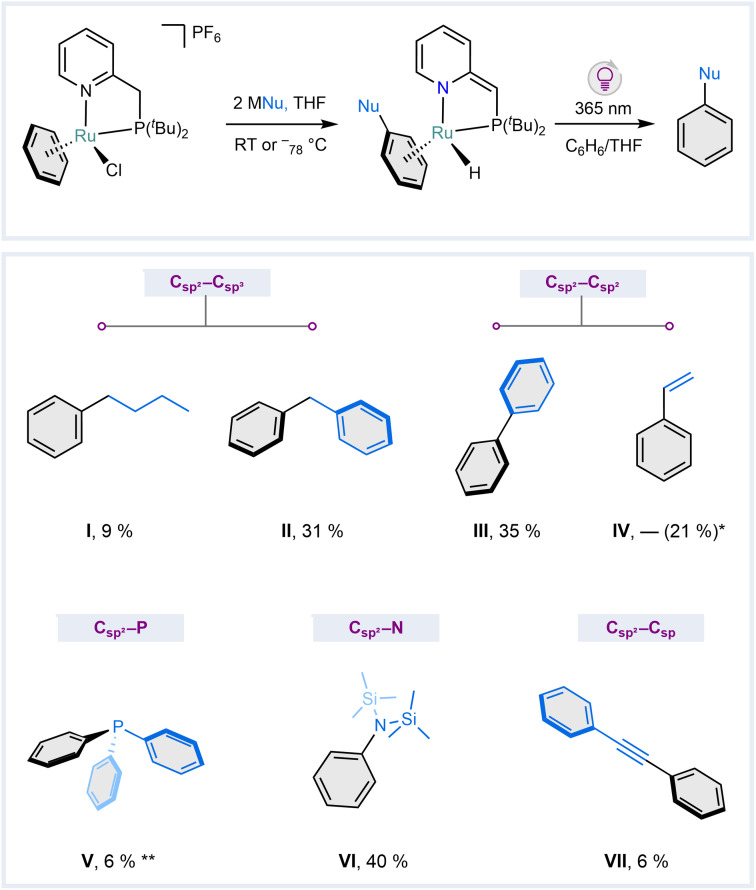
S_N_ArH on benzene mediated by complex 1 with various nucleophiles. I: *n*-butyllithium, II: benzyl potassium, III: phenylmagnesium bromide, IV: vinylmagnesium bromide (quantified based on the qNMR data of the corresponding complex), V: potassium diphenylphosphide (analyzed in the form of its oxide), VI: potassium bis(trimethylsilyl)amide, VII: lithium phenylacetylide (basic work-up was done instead of the UV irradiation). The yields were determined by GC analysis. * based on spectroscopic yield of the corresponding hydride complex; ** based on GC yield of O=PPh_3_ following a reaction with H_2_O_2_; for more details, see ESI Section S4.2.†

The state-of-the-art S_N_ArH reactions using β-diketiminate complexes (Fig. 1d) are limited to intramolecular alkyl nucleophiles. Given that the reactivity described above involves an exogenous nucleophile that is commonly used as a non-nucleophilic bulky base, we investigated the scope of nucleophiles amenable to the Ru-mediated S_N_ArH reaction. Motivated by both the need to obtain the metal-free substitution product and the difficulties in accurately determining spectroscopic yields with some nucleophiles used, we first developed a protocol that enables liberation of the silylated aniline in 4 (ESI Section S4.1†). Although it is known that arene exchange reactions on Ru are challenging, especially in neutral complexes featuring electron-rich arenes,^44,45^ we found that irradiation of a refluxing benzene solution of 4 with a 365 nm UV light for 72 h enables partial arene exchange to give “free” PhN(TMS)_2_ in a modest yield of 40% based on GC analysis (VI, Fig. 6). This confirms the strong binding energy between the ruthenium center and bis-silylated aniline (see ESI Section S3.3† for TD-DFT calculations). Although it is not optimal, we used this protocol to assess the scope of nucleophiles amenable to the S_N_ArH reaction in 1. GC analysis of the reaction mixtures (after arene exchange) with C(sp^3^)-based nucleophiles *n*-butyl lithium (^*n*^BuLi) and benzyl potassium (BnK) showed the formation of the targeted butyl benzene (I) in 9% yield and diphenylmethane (II) in 31% yield. Similarly, a reaction with phenylmagnesium bromide as a C(sp^2^)-based nucleophile showed the formation of biphenyl (III) in 35% yield. A reaction with vinylmagnesium bromide to give styrene (IV) is incompatible with our protocol, given that the reaction product polymerizes under UV light.^46^ However, ^1^H NMR analysis of the reaction mixture after mixing 1 and vinyl magnesium bromide showed 21% formation of two hydride species at similar chemical shifts as those observed for complex 4 (ESI Fig. S145 and S146†). This suggests the formation of a similar complex as 4 but with coordinated styrene instead. Using different arene exchange protocols, triphenylphosphine (VI) and diphenylacetylene (VII) were generated by reaction with potassium diphenyl phosphide or lithium phenyl acetylide, respectively, albeit in only 6% yield. Although these non-optimized yields and protocols are far from practical, they demonstrate that the Ru-mediated S_N_ArH reaction is compatible with a variety of nucleophiles.

## Conclusion

In conclusion, we have uncovered a new ruthenium-mediated pathway for nucleophilic aromatic substitution of hydrogen (S_N_ArH) on benzene. Remarkably, this is the first example of S_N_ArH on a transition metal arene complex using exogenous nucleophiles that do not require a leaving group on the arene nor subsequent oxidation steps. Our detailed mechanistic studies show that the reaction involves reversible rear-side addition of the nucleophile followed by an unprecedented intramolecular hydride migration from the metal-stabilized Jackson–Meisenheimer intermediate to the metal center. This last key step is facilitated by deprotonation of the ^*t*Bu^PN ligand, which makes the metal center more electron-rich, thereby promoting chloride dissociation and subsequent hydride migration. The reaction demonstrates broad versatility, accommodating diverse nucleophiles, including C_sp^3^_, C_sp^2^_, C_sp_, N, and P-based ones. We envision that this study opens up new avenues for developing new stoichiometric and catalytic S_N_ArH reactions. To achieve this, our ongoing efforts are aimed at enabling efficient arene exchange and vacant site regeneration *via* hydride loss.

### Methods

#### General considerations

All manipulations were performed under an inert atmosphere using standard Schlenk techniques or inside of an N_2_-filled M. Braun glovebox using dry solvents and reagents unless stated otherwise. Glassware was dried at 130 °C in an oven or with a heat gun under a dynamic vacuum unless noted otherwise. Hexane and DCM were collected from an M. Braun MB-SPS-800 solvent purification system and degassed and stored over 4 Å molecular sieves, except for DCM which was dried over 3 Å molecular sieves. THF was dried over Na/benzophenone ketyl (purple), or over Na dispersed on silica spheres, vacuum-transferred, and degassed, subsequently followed by storage over 4 Å molecular sieves. Benzene (Scharlab, >99%) and pentane (technical, VWR chemicals) were degassed, then dried and stored over 4 Å molecular sieves. All non-deuterated solvents were degassed by bubbling N_2_ (g) through the solvent for at least 30 min. Water content in non-deuterated solvents was tested using Karl-Fischer titration, and all but DCM were also tested by titration with a standard purple solution of sodium benzophenone ketyl in THF to confirm effective oxygen and water removal (for 1 mL of solvent, max 1–2 drops for most solvents, max 4 drops for THF). Deuterated solvents were obtained from Cambridge Isotope Laboratories except for THF-*d*_8_, which was obtained from ABCR, degassed by the standard freeze–pump–thaw procedure, and stored over 4 Å molecular sieves (3 Å for CD_2_Cl_2_). All commercial reagents were used as received and were obtained from Sigma Aldrich, Acros, and Strem. ^*t*Bu^PN,^47^ PON,^48^ NN,^49^ PhN(SiMe_3_)_2_,^50^ lithium phenylacetylide^51^ were prepared according to literature procedures.

#### NMR measurements

NMR data was recorded on a VNMRS-400 Varian 400 MHz (9.4 T) NMR system equipped with an OneNMR probe (with quartz liner) and Optima Tune system and a Performa IV PFG amplifier capable of generating a 65 G cm^−1^ gradient or a Jeol JNM-ECZL G 400 MHz (9.4 T) NMR system equipped with an auto-tunable ROYALPROBE HFX (with quartz liner) and a gradient amplifier capable of generating a 90 G cm^−1^ gradient. All chemical shifts are reported in the standard *δ* notation of parts per million, referenced to the residual solvent peak. All resonances in ^1^H NMR and ^13^C NMR spectra were referenced to residual solvent peaks (^1^H NMR: 7.16 for C_6_D_6_, 3.58 for THF-*d*_8_, 5.32 for CD_2_Cl_2_, ^13^C NMR: 128.06 for C_6_D_6_, 67.57 for THF-*d*_8_, 53.84 for CD_2_Cl_2_). The resonances in the ^31^P NMR spectra are referenced using the absolute reference method from a correctly referenced ^1^H NMR spectrum of the same sample. The assignment of peaks is based on relative integration, chemical shift, and 2D NMR analysis (COSY, TOCSY, HMBC, HMQC, NOESY, and J-resolved experiments). For ^1^H NMR spectra in non-deuterated solvents, solvent suppression is used (PRESAT). All NMR experiments involving air-sensitive compounds were conducted in J. Young NMR tubes under an N_2_ atmosphere. Peak multiplicity was quoted as s (singlet), d (doublet), t (triplet), and so on. For quantitative analyses, the acquisition time was chosen so that the full FID was recorded, and the relaxation delay was set to 7 times the longest T1, determined by an individual T1 measurement. Inverse gated decoupling was employed where necessary.

#### Other physical methods

GC analyses were performed on a PerkinElmer Clarus 500 Gas Chromatograph equipped with a PE Elite-5 column ((30 m × 0.32 mm × 0.25 μm), (5% phenyl)–(95% methyl)polysiloxane) and a flame-ionization detector. The calculations and plotting of the GC calibration were performed using an in-house script written in the Python programming language (Python Software Foundation. Python Language Reference, version 3.11.4. Available at http://www.python.org). ATR-FTIR spectra were recorded on a PerkinElmer Frontier FTIR spectrometer equipped with a PerkinElmer Universal Attenuated Total Reflectance (ATR) sampling accessory with a diamond/ZnSe plate and a LiTaO_3_ mid-IR detector. IR analysis of air-sensitive compounds was performed by dropcasting a solution (THF, DCM, or pentane) onto the ATR crystal, which was covered by a continuous N_2_ flow. Elemental analysis was performed by MEDAC Ltd. Based in the United Kingdom.

#### UV experiments

For the UV light irradiation experiments, a set-up that consisted of a double-walled quartz tube and a UV light source^52^ was used. The UV light source consists of flexible Waveform Lighting realUVTM 365 nm LED strip lights (2.46 W per 1 meter) wrapped around a brass rod. This type of LED has a single sharp peak at 365 nm in the spectrum. The rod with the LED lights is placed inside the double-walled quartz tube, which is actively cooled with water during irradiation experiments (“cold” UV). When the water cooling is not used (“hot” UV), the quartz tube gets warm (∼45 °C). J. Young valved NMR tubes containing the solutions of complexes were then placed around the quartz tube. The standard distance of 1–3 mm between the lamp and an NMR tube was used unless different is stated (see ESI Fig. S129†).

### Synthetic procedures

*Note: see ESI Section S1† for synthetic procedures and characterization of the compounds presented in this study.*

#### [(^*t*Bu^PN)RuCl(C_6_H_6_)][PF_6_] (1)

A 100 mL Schlenk tube was charged with ^*t*Bu^PN (118.7 mg, 0.50 mmol), KPF_6_ (115.0 mg, 0.63 mmol), and [Ru_2_Cl_4_(C_6_H_6_)_2_] (125.0 mg, 0.25 mmol). Next, DCM (10.0 mL) was added to give an orange suspension. The reaction mixture was kept stirring in a glovebox at RT for 18 h, and the color of the reaction mixture became dark brown. The mixture was filtered through a glass filter from unreacted KPF_6_ and KCl, to give a dark brown filtrate. After removing volatiles under a dynamic vacuum, the resulting residue was suspended in 3.0 mL of THF and stirred for 15 min. The mixture was filtered and the residue was dried under a dynamic vacuum, giving 132.0 mg (44%) of a bright yellow powder. Crystals suitable for X-ray diffraction analysis were grown by vapor diffusion of THF into a solution of 1 in DCM at room temperature.

*Note: the product has moderate solubility only in DCM and MeCN. Washing with THF (note that as little as possible of THF should be used as 1 is partially soluble in THF) is required to get rid of byproducts of the reaction, the crystal structure of one of them – (* ^*tBu*^ *PN)*_*2*_ *RuCl*_*2*_ *– was fortuitously also obtained (see ESI Section S6.3†).*

^1^H NMR (400 MHz, CD_2_Cl_2_, 298 K): *δ* = 9.24 (d, ^3^*J*_H,H_ = 5.0 Hz, 1H), 7.87–7.80 (m, 1H), 7.44 (d, ^3^*J*_H,H_ = 7.8 Hz, 1H), 7.39–7.34 (m, 1H), 6.11 (d, ^3^*J*_H,P_ = 0.7 Hz, 6H), 3.89 (dd, ^2^*J*_H,H_ = 16.4, ^2^*J*_H,P_ = 8.7 Hz, 1H), 3.31 (dd, ^2^*J*_H,H_ = 16.4, ^2^*J*_H,P_ = 13.3 Hz, 1H), 1.57 (d, ^3^*J*_H,P_ = 14.5 Hz, 9H), 1.21 (d, ^3^*J*_H,P_ = 13.4 Hz, 9H).

^13^C{^1^H} NMR (101 MHz, CD_2_Cl_2_, 298 K): *δ* = 163.0 (d, ^4^*J*_C,P_ = 3.1 Hz), 157.5 (s), 140.5 (d), 125.1 (s), 125.0 (s), 89.7 (d, ^2^*J*_C,P_ = 2.4 Hz), 39.6 (d, ^1^*J*_C,P_ = 2.3 Hz), 39.5 (d, ^2^*J*_C,P_ = 3.1 Hz), 33.6 (d, ^1^*J*_C,P_ = 23.7 Hz), 31.6 (d, ^2^*J*_C,P_ = 2.3 Hz), 29.9 (d, ^2^*J*_C,P_ = 2.7 Hz).

^31^P{^1^H} NMR (162 MHz, CD_2_Cl_2_, 298 K): *δ* = 90.8 (s, 1P), −144.4 (hept, ^1^*J*_P,F_ = 710.8 Hz, 1P).

^19^F NMR (376 MHz, CD_2_Cl_2_, 298 K): *δ* = −72.7 (d, ^1^*J*_F,P_ = 711.0 Hz, 6F).

Anal. calcd for C_20_H_30_ClNP_2_RuF_6_: C, 40.24; H, 5.07; N, 2.35. Found: C, 39.69; H, 5.04; N, 2.22.

ATR-IR (film, N_2_ flow): *ν* = 3090 (w), 2964 (m), 2924 (m), 2873 (w), 1607 (w), 1474 (m), 1441 (m), 1387 (w), 1373 (w), 1312 (w), 1269 (w), 1178 (w), 1024 (w), 876 (w), 835 (s), 776 (w), 734 (m), 702 (w), 621 (w), 557 (s), 493 (w), 460 (w) cm^−1^.

#### [(^*t*Bu^PN*)RuCl(C_6_H_6_)K(THF)_*n*_]PF_6_ (2-K) and [(^*t*Bu^PN)RuH(PhN(TMS)_2_)]PF_6_ (3)

A colorless solution of KN(TMS)_2_ (8.0 mg, 0.04 mmol) in THF-*d*_8_ (1.5 mL) was added dropwise to a yellow suspension of complex 1 (23.9 mg, 0.04 mmol) in THF-*d*_8_ (1.5 mL), resulting in a dark brown solution. The vial with the reaction mixture was kept stirring for 15 min at RT after which a sample was transferred into a J. Young tube and analyzed by NMR spectroscopy.

*Note: the crude* ^*1*^ *H NMR spectrum shows the formation of ∼50% species 2-K and ∼20% species 3 based on the relative integral values. For a cleaner synthesis route towards 2-K as well as an alternative route to the mixture of 2K and 3 see ESI Section S1.†*

For 2-K:

^1^H NMR (400 MHz, THF-*d*_8_, 298 K): *δ* = 8.21 (ddd, *J* = 5.3, 1.6, 1.2 Hz, 1H), 7.62 (ddd, *J* = 7.9, 7.9, 1.5 Hz, 1H), 7.06 (dddd, *J* = 7.8, 5.3, 1.3, 1.3 Hz, 1H), 6.72 (dd, *J* = 7.9, 1.0 Hz, 1H), 5.82 (s, 6H), 3.33 (d, ^2^*J*_H,P_ = 3.0 Hz, 1H), 1.36 (d, ^3^*J*_H,P_ = 14.9 Hz, 9H), 1.08 (d, ^3^*J*_H,P_ = 15.4 Hz, 9H).

^31^P{^1^H} NMR (162 MHz, THF-*d*_8_, 298 K): *δ* = 97.5 (s, 1P), −144.5 (hept, ^1^*J*_P,F_ = 710.1 Hz, 1P).

For 3:

^1^H NMR (400 MHz, THF-*d*_8_, 298 K): *δ* = 8.86 (d, *J* = 5.4 Hz, 1H), 7.71 (t, *J* = 8.3 Hz, 1H), 7.51 (d, *J* = 7.6 Hz, 1H), 7.14 (dd, *J* = 7.6, 6.7 Hz, 1H), 6.27 (t, *J* = 6.2 Hz, 1H), 6.21 (d, *J* = 5.5 Hz, 1H), 5.69 (dd, *J* = 6.2, 1.5 Hz, 1H), 5.44–5.39 (m, 1H), 4.64 (dt, *J* = 5.9, 1.6 Hz, 1H), 3.64–3.56 (m, 1H, overlapped with a THF signal), 3.26 (dd, *J* = 17.2, 7.6 Hz, 1H), 1.34 (d, ^3^*J*_H,P_ = 13.7 Hz, 9H), 1.25 (d, ^3^*J*_H,P_ = 13.0 Hz, 9H), 0.30 (s, 18H), −7.77 (d, *J* = 42.5 Hz, 1H).

^31^P{^1^H} NMR (162 MHz, THF-*d*_8_, 298 K): *δ* = 111.8 (d*, ^2^*J*_P,H_ = 11.2 Hz, 1P), −144.5 (hept, ^1^*J*_P,F_ = 710.1 Hz, 1P).

*The doublet appears due to partial coupling with the hydride.

#### (^*t*Bu^PN*)RuH(PhN(TMS)_2_) (4)

A yellow suspension of complex 1 (96.8 mg, 0.16 mmol) in THF (6.0 mL) was added dropwise to a colorless solution of KN(TMS)_2_ (64.7 mg, 0.32 mmol) in THF (4.0 mL). The starting complex instantly dissolved upon the addition, resulting in a color change to dark brown. After stirring for 0.5 h the mixture was dried under a dynamic vacuum to give a dark brown solid. The residue was extracted with pentane (5.0 mL), and the extracts were dried under a dynamic vacuum to give a dark brown sticky solid (90.3 mg, 97%). Crystals suitable for X-ray diffraction analysis were grown by keeping a concentrated solution of 4 in pentane at −40 °C.

^1^H NMR (400 MHz, C_6_D_6_, 298 K): *δ* = 7.33 (dd, ^3^*J*_H,H_ = 6.3, ^4^*J*_H,H_ = 0.9 Hz, 1H), 6.53 (dddd, ^3^*J*_H,H_ = 9.0, ^3^*J*_H,H_ = 6.3, ^5^*J*_H,P_ = 2.1, ^4^*J*_H,H_ = 1.4 Hz, 1H), 6.41 (d, ^3^*J*_H,H_ = 8.8 Hz, 1H), 5.38 (ddd, ^3^*J*_H,H_ = 7.1, ^3^*J*_H,H_ = 6.3, ^4^*J*_H,H_ = 1.4 Hz, 1H), 5.11 (dd, ^3^*J*_H,H_ = 6.2, ^3^*J*_H,H_ = 5.9 Hz, 1H), 4.94 (dd, ^3^*J*_H,H_ = 6.2, ^3^*J*_H,H_ = 5.2 Hz, 1H), 4.77 (dd, ^3^*J*_H,H_ = 6.0, ^3^*J*_H,H_ = 4.9 Hz, 1H), 4.67 (dd, ^3^*J*_H,H_ = 6.0, ^3^*J*_H,H_ = 1.9 Hz, 1H), 4.14 (ddd, ^3^*J*_H,H_ = 5.7, ^4^*J*_H,H_ = 2.2, ^4^*J*_H,H_ = 1.8 Hz, 1H), 3.49 (d, ^2^*J*_H,P_ = 2.6 Hz, 1H), 1.31 (d, ^3^*J*_H,P_ = 12.4 Hz, 9H), 1.27 (d, ^3^*J*_H,P_ = 13.2 Hz, 9H), 0.25 (s, 18H), −7.76 (d, ^2^*J*_H,P_ = 43.4 Hz, 1H).

^13^C{^1^H} NMR (101 MHz, C_6_D_6_, 298 K): *δ* = 170.9 (d, ^2^*J*_C,P_ = 15.6 Hz), 154.4 (s), 130.8 (d, ^4^*J*_C,P_ = 2.3 Hz), 130.7 (s), 115.2 (d, ^3^*J*_C,P_ = 17.2 Hz), 101.4 (s), 92.4 (s), 90.6 (d, ^2^*J*_C,P_ = 5.7 Hz), 83.4 (s), 77.4 (s), 74.1 (d, ^2^*J*_C,P_ = 3.1 Hz), 62.3 (d, ^1^*J*_C,P_ = 60.3 Hz), 38.3 (d, ^1^*J*_C,P_ = 14.5 Hz), 36.2 (d, ^1^*J*_C,P_ = 34.3 Hz), 31.1 (d, ^2^*J*_C,P_ = 3.4 Hz), 30.2 (d, ^2^*J*_C,P_ = 5.0 Hz), 3.3 (s).

^31^P{^1^H} NMR (162 MHz, C_6_D_6_, 298 K): *δ* = 98.8 (s).

ATR-IR (film, N_2_ flow): *ν* = 3045 (m), 2954 (s), 2864 (s), 2893 (m), 2034 (w, br), 1604 (s), 1535 (w), 1511 (w), 1488 (s), 1446 (s), 1381 (w), 1359 (w), 1358 (w), 1285 (m), 1253 (m), 1225 (m), 1205 (m), 1179 (w), 1146 (w), 1101 (w), 1017 (w), 1000 (m), 933 (m), 892 (s), 840 (m), 810 (m), 758 (w), 726 (w), 687 (w), 667 (w), 616 (w), 503 (w), 463 (w) cm^−1^.

*Despite several attempts using spectroscopically pure samples, the reactive nature of 4 precluded obtaining a satisfactory elemental analysis.*

### X-ray crystal structure determination of 4

C_26_H_47_N_2_PRuSi_2_, Fw = 575.87, orange block, 0.41 × 0.40 × 0.17 mm^3^, monoclinic, *P*2_1_/*c* (no. 14), *a* = 13.9298(4), *b* = 16.2626(4), *c* = 13.8275(4) Å, *β* = 112.534(2), *V* = 2893.25(15) Å^3^, *Z* = 4, *D*_x_ = 1.322 g cm^−3^, *μ* = 0.70 mm^−1^. The diffraction experiment was performed on a Bruker Kappa ApexII diffractometer with a sealed tube and Triumph monochromator (*λ* = 0.71073 Å) at a temperature of 150(2) K up to a resolution of (sin *θ*/*λ*)_max_ = 0.65 Å^−1^. The crystal was broken into several fragments. Two orientation matrices were used for the intensity integration of the major fragments using the Eval15 software.^53^ Only the non-overlapping reflections were used for structure solution and refinement. A multi-scan absorption correction and scaling were performed with SADABS^54^ (correction range 0.68–0.75). A total of 41381 reflections were measured, 6635 reflections were unique (*R*_int_ = 0.021), and 6179 reflections were observed [*I* > 2*σ*(*I*)]. The structure was solved with Patterson superposition methods using SHELXT.^55^ Structure refinement was performed with SHELXL-2018 (ref. 56) on *F*^2^ of all reflections. Non-hydrogen atoms were refined freely with anisotropic displacement parameters. All hydrogen atoms were located in difference Fourier maps. Metal-bound hydrogen atom H^1^ and hydrogens H^16^–H^20^ of the coordinated phenyl group were refined freely with isotropic displacement parameters. All other hydrogen atoms were refined with a riding model. 329 parameters were refined with no restraints. *R*_1_/*wR*_2_ [*I* > 2*σ*(I)]: 0.0200/0.0507. *R*_1_/*wR*_2_ [all refl.]: 0.0217/0.0516. *S* = 1.045. Residual electron density between −0.42 and 0.49 e Å^−3^. Geometry calculations and checking for higher symmetry were performed with the PLATON program.^57^

*Note: for further structural details see ESI Section S6.†*

### Computational details

All calculations were carried out using DFT^58^ as implemented in ORCA 5.0.3 ^59–61^ with the B3LYP,^62,63^ including Grimme's D3 dispersion correction with Becke–Johnson damping.^64–69^ Geometry optimizations and analytical vibrational frequency calculations were carried out with the def2-SVP basis set^70^ with def2-ECP for Ru.^71^ For all optimized structures, the intermediates were confirmed with no imaginary vibrational frequency, while transition states showed a single imaginary frequency with a motion corresponding to the proper transitions. The solvated energies of optimized structures were re-evaluated by additional single-point calculations on each optimized geometry using the def2-TZVPD basis set.^70^ For all calculations, the RIJCOSX approximation^72,73^ was utilized with the auxiliary basis set def2/J.^74^ To model the solution environment for tetrahydrofuran, the solvation model based on density (SMD)^75^ was utilized with parameters that have been implemented in ORCA. TD-DFT calculations for modeling excited states were conducted as implemented in Q-Chem 5.4 software.^76^ Geometries from the optimized geometry with ORCA were utilized for the calculations of excited states. Single Excitation Configuration Interaction (CIS)^77^ and Tamm–Dancoff approximation^78^ were utilized to reduce the computation cost without damage to the quality of the results. The functional and basis set for the calculations of the excited state are identical to those for DFT calculations.

## Author contributions

S. M. and D. L. J. B. conceived the project. S. M. performed the experiments, including the synthesis, characterization, and analysis with support from P. G. D. H. and B. P. carried out computational calculations. M. L. performed crystallographic measurements, including the acquisition, solving, and interpretation of the crystal structures. D. L. J. B. supervised the project. The original draft was written by S. M. and was reviewed by M.-H. B. and D. L. J. B. with contributions by all authors.

## Conflicts of interest

The authors declare no conflict of interest.

## Supplementary Material

SC-OLF-D5SC02090E-s001

SC-OLF-D5SC02090E-s002

SC-OLF-D5SC02090E-s003

## Data Availability

All data can be found in the main text, Methods section, or the ESI Materials.† CCDC 2355856–2355858 contains the supplementary crystallographic data for this paper. These data can be obtained free of charge from The Cambridge Crystallographic Data Centre *via*https://www.ccdc.cam.ac.uk/data_request/cif. The spectroscopic files that support the findings of this study are openly available in the Yoda data repository at DOI: https://doi.org/10.24416/UU01-BYOFTG.
