# Supplementary material for: Ruthenium-mediated nucleophilic aromatic substitution of hydrogen in benzene
Source: Chem Sci. 2025 Jun 16;16(29):13422–34. doi: 10.1039/d5sc02090e (PMC12198976; doi:10.1039/d5sc02090e)
Supplement: SC-016-D5SC02090E-s001 [file SC-016-D5SC02090E-s001.pdf]

Supplementary Information (SI) for Chemical Science.
This journal is © The Royal Society of Chemistry 2025

Supplementary Materials

Ruthenium-Mediated Nucleophilic Aromatic Substitution of Hydrogen in Benzene

Stanislav Melnikov[1], Donghun Hwang[2,3], Philip Gabbert[1], Bohyun Park[2,3], Martin Lutz[4], Mu-Hyun Baik*[3,2], Daniël L.J. Broere*[1]

[1]Organic Chemistry and Catalysis, Institute for Sustainable and Circular Chemistry, Faculty of Science, Utrecht University, Universiteitsweg 99, 3584 CG Utrecht, The Netherlands

Email: d.l.j.broere@uu.nl

[2]Department of Chemistry, Korea Advanced Institute of Science and Technology (KAIST), Daejeon 34141, Republic of Korea

[3]Center for Catalytic Hydrocarbon Functionalizations, Institute for Basic Science (IBS), Daejeon 34141, Republic of Korea

Email: mbaik2805@kaist.ac.kr

[4]Structural Biochemistry, Bijvoet Centre for Biomolecular Research, Faculty of Science, Utrecht University Universiteitsweg 99, Utrecht, The Netherlands

***Corresponding Author**

Table of content

S1 Synthesis and Characterization 4
[(^{tBu}PN)RuCl(C_6H_6)][PF_6] (**1**) 4
[(^{tBu}PN*)RuCl(C_6H_6)K(THF)$_n$]PF_6 (**2-K**) and [(^{tBu}PN)RuH(PhN(TMS)$_2$)]PF_6 (**3**) 10
(^{tBu}PN*)RuH(PhN(TMS)$_2$) (**4**) 15
Hexadeuterocyclohexadienes (**CHDs**) 23
$Ru_2Cl_4(\eta^6$-$C_6D_6)_2$ (**5**) 25
[(^{tBu}PN)RuCl(C_6D_6)][PF_6] (**1-D**) 28
(^{tBu}PN*)RuD(PhN(TMS)$_2$-d_5) (**4-D**) 33
S2 Mechanistic Studies 38
S2.1 Room-Temperature Experiments 38
Nature of Complex **2-K** 38
On the origin of the formation of **3** 38
(^{tBu}PN*)RuCl(C_6H_6) (**2**) 43
Direct synthesis of **2-K** from **2** 45
S2.2 Low-temperature NMR experiments 48
S2.3 Experiments with other ligands 60
[(PON)RuCl(C_6H_6)]PF_6 61
[(NN)RuCl(C_6H_6)]PF_6 67
[(^{t}Bu-bpy)RuCl(C_6H_6)]PF_6 71
[(phen)RuCl(C_6H_6)]PF_6 75
[(TMEDA)RuCl(C_6H_6)]PF_6 80
Reactions of the various metal complexes with KN(TMS)$_2$ 84
S3 Computational details 89
S3.1 Alternative Pathways 90
S3.2 Analysis of Fukui Functions 94
S3.3 TD-DFT studies 95
S4 Scope of nucleophiles 97
S4.1 Optimisation of the arene decoordination protocol 97
Fate of [Ru] after UV irradiation experiments 99
S4.2 Experiments with other nucleophiles 102
S5 GC Measurements 111
S5.2 GC chromatograms of the products 116
S6 Crystal Structures 122
S6.1 X-ray crystal structure determination of **1** 122

S6.2 X-ray crystal structure determination of **4**.......... 124
S6.3 X-ray crystal structure determination of **(^{tBu}PN)$_2$RuCl$_2$**.......... 127
S8 References.......... 129

S1 Synthesis and Characterization

[(^{tBu}PN)RuCl(C_6H_6)][PF_6] (1)

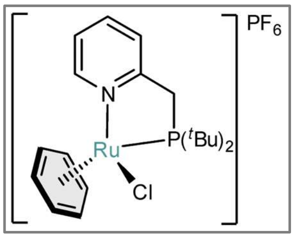

A 100 mL Schlenk tube was charged with ^{tBu}PN (118.7 mg, 0.50 mmol), KPF_6 (115.0 mg, 0.63 mmol), and [$Ru_2Cl_4(C_6H_6)_2$] (125.0 mg, 0.25 mmol). Next, DCM (10.0 mL) was added to give an orange suspension. The reaction mixture was kept stirring in a glovebox at RT for 18 h, and the color of the reaction mixture became dark brown. The mixture was filtered through a glass filter from unreacted KPF_6 and KCl, to give a dark brown filtrate. After removing volatiles under a dynamic vacuum, the resulting residue was suspended in 3.0 mL of THF and stirred for 15 min. The mixture was filtered and the residue was dried under a dynamic vacuum giving 132.0 mg (44%) of a bright yellow powder. Crystals suitable for X-ray diffraction analysis were grown by vapor diffusion of THF into a solution of **1** in DCM at room temperature.

*Note: the product has moderate solubility only in DCM and MeCN. Washing with THF (note that as little as possible of THF should be used as **1** is partially soluble in THF) is required to get rid of byproducts of the reaction, the crystal structure of one of them – (^{tBu}PN)$_2$RuCl$_2$ – was fortuitously also obtained (see Supplementary Section S6.3).*

^{1}H NMR (400 MHz, CD_2Cl_2, 298K): δ = 9.24 (d, $^3J_{H,H}$ = 5.0 Hz, 1H), 7.87 – 7.80 (m, 1H), 7.44 (d, $^3J_{H,H}$ = 7.8 Hz, 1H), 7.39 – 7.34 (m, 1H), 6.11 (d, $^3J_{H,P}$ = 0.7 Hz, 6H), 3.89 (dd, $^2J_{H,H}$ = 16.4, $^2J_{H,P}$ = 8.7 Hz, 1H), 3.31 (dd, $^2J_{H,H}$ = 16.4, $^2J_{H,P}$ = 13.3 Hz, 1H), 1.57 (d, $^3J_{H,P}$ = 14.5 Hz, 9H), 1.21 (d, $^3J_{H,P}$ = 13.4 Hz, 9H).

^{13}C{^{1}H} NMR (100 MHz, CD_2Cl_2, 298K): δ = 163.0 (d, $^4J_{C,P}$ = 3.1 Hz), 157.5 (s), 140.5 (d,), 125.1 (s), 125.0 (s), 89.7 (d, $^2J_{C,P}$ = 2.4 Hz), 39.6 (d, $^1J_{C,P}$ = 2.3 Hz), 39.5 (d, $^2J_{C,P}$ = 3.1 Hz), 33.6 (d, $^1J_{C,P}$ = 23.7 Hz), 31.6 (d, $^2J_{C,P}$ = 2.3 Hz), 29.9 (d, $^2J_{C,P}$ = 2.7 Hz).

^{31}P{^{1}H} NMR (162 MHz, CD_2Cl_2, 298K): δ = 90.8 (s, 1P), –144.4 (hept, $^1J_{P,F}$ = 710.8 Hz, 1P).

^{19}F NMR (376 MHz, CD_2Cl_2, 298K): δ = –72.7 (d, $^1J_{F,P}$ = 711.0 Hz, 6F).

Anal. Calcd. For $C_{20}H_{30}ClNP_2RuF_6$: C, 40.24; H, 5.07; N, 2.35. **Found:** C, 39.69; H, 5.04; N, 2.22.

ATR-IR (film, N_2 flow): ν = 3090 (w), 2964 (m), 2924 (m), 2873 (w), 1607 (w), 1474 (m), 1441 (m), 1387 (w), 1373 (w), 1312 (w), 1269 (w), 1178 (w), 1024 (w), 876 (w), 835 (s), 776 (w), 734 (m), 702 (w), 621 (w), 557 (s), 493 (w), 460 (w) cm^{-1}.

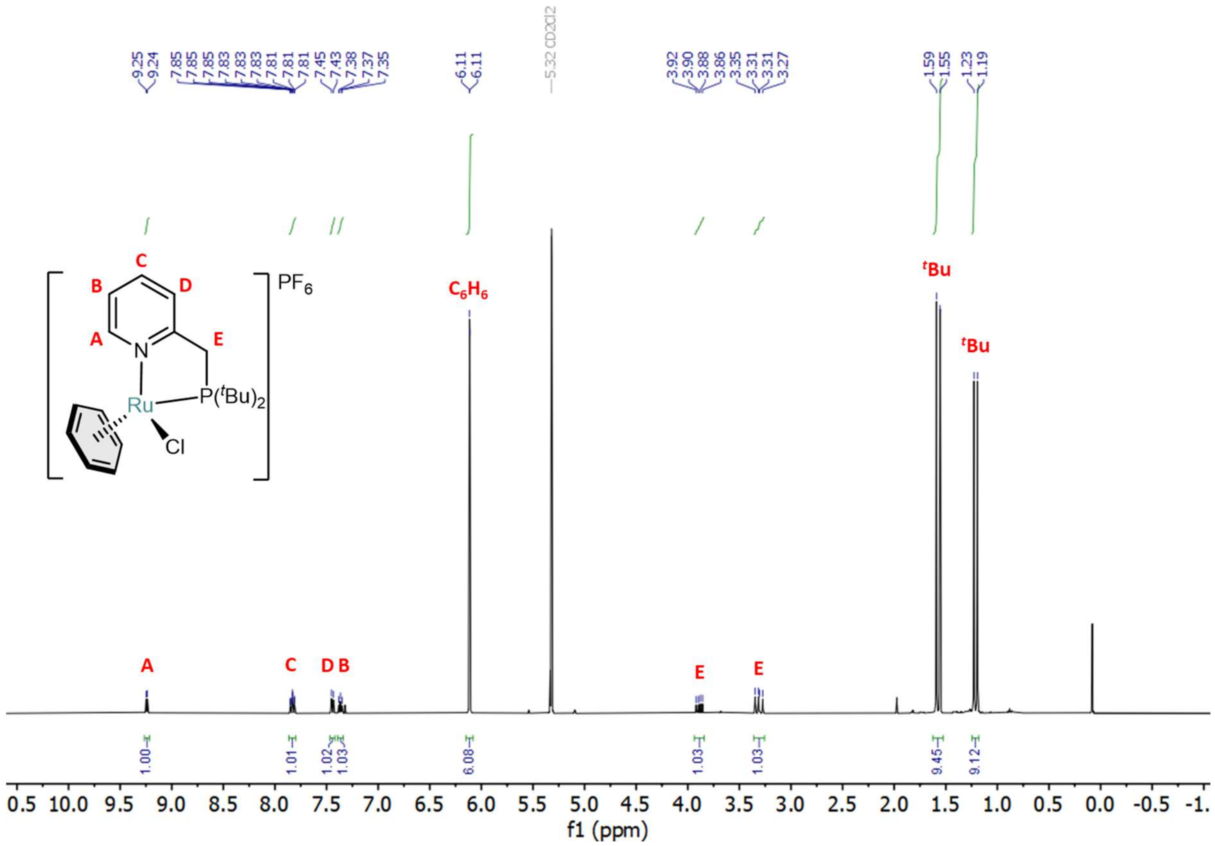

Figure S1. The ^{1}H NMR spectrum of complex **1** in CD_2Cl_2 at 298 K.

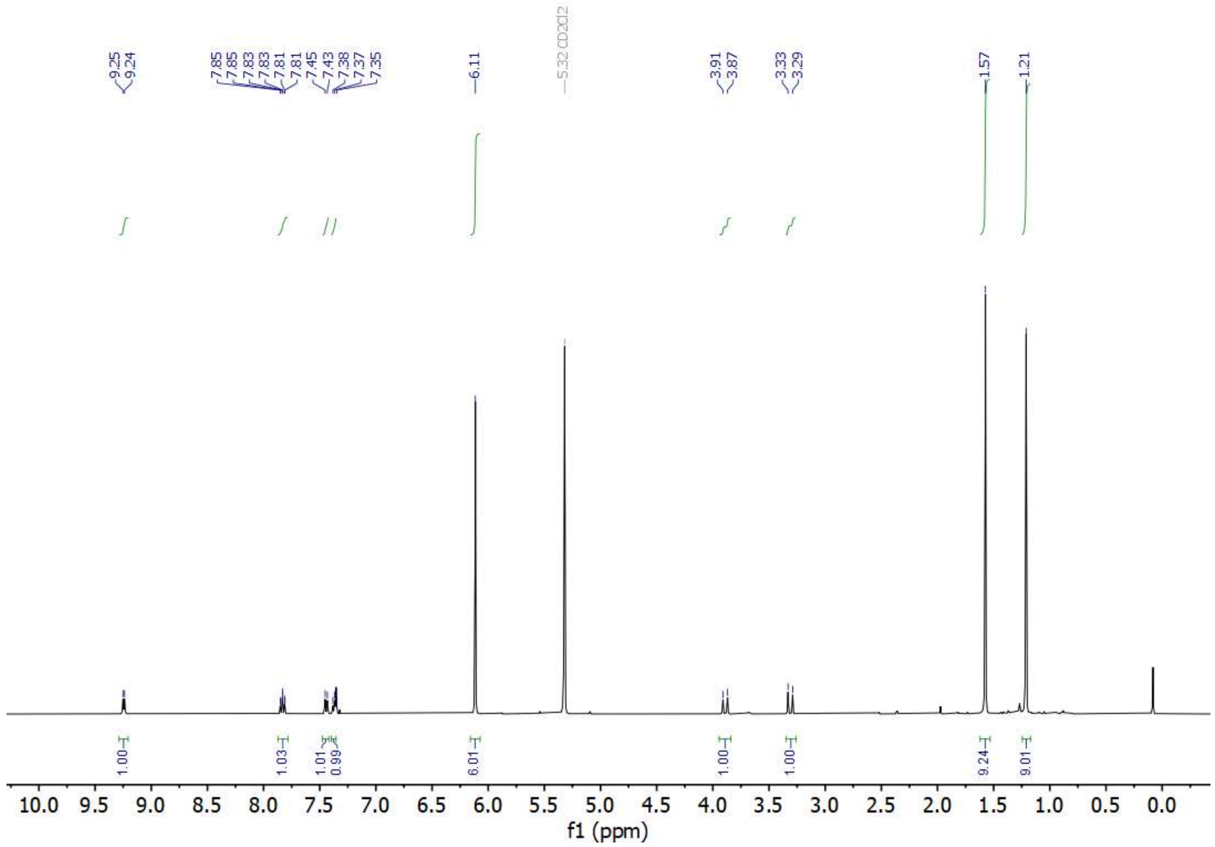

Figure S2. The $^{1}H\{^{31}P\}$ NMR spectrum of complex **1** in CD_2Cl_2 at 298 K.

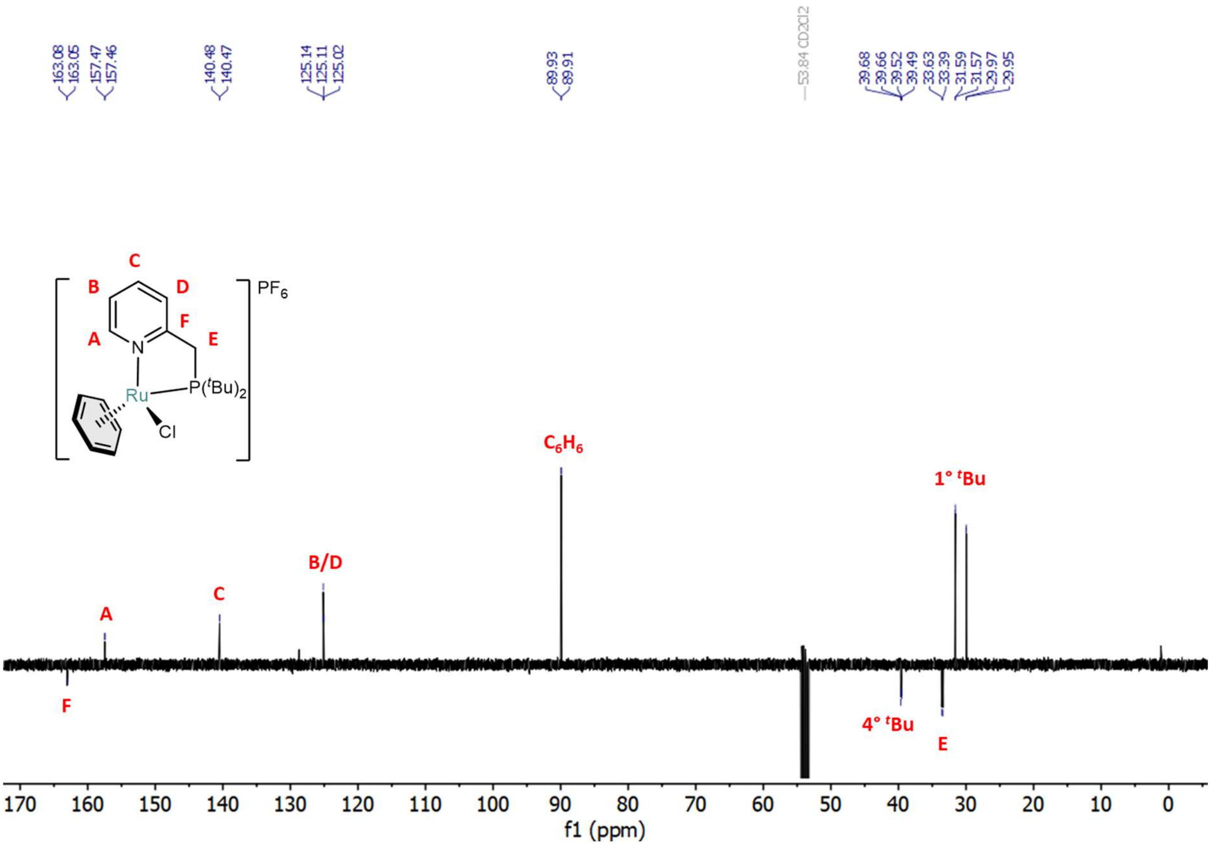

Figure S3. The ^{13}C(APT) NMR spectrum of complex **1** in CD_2Cl_2 at 298 K.

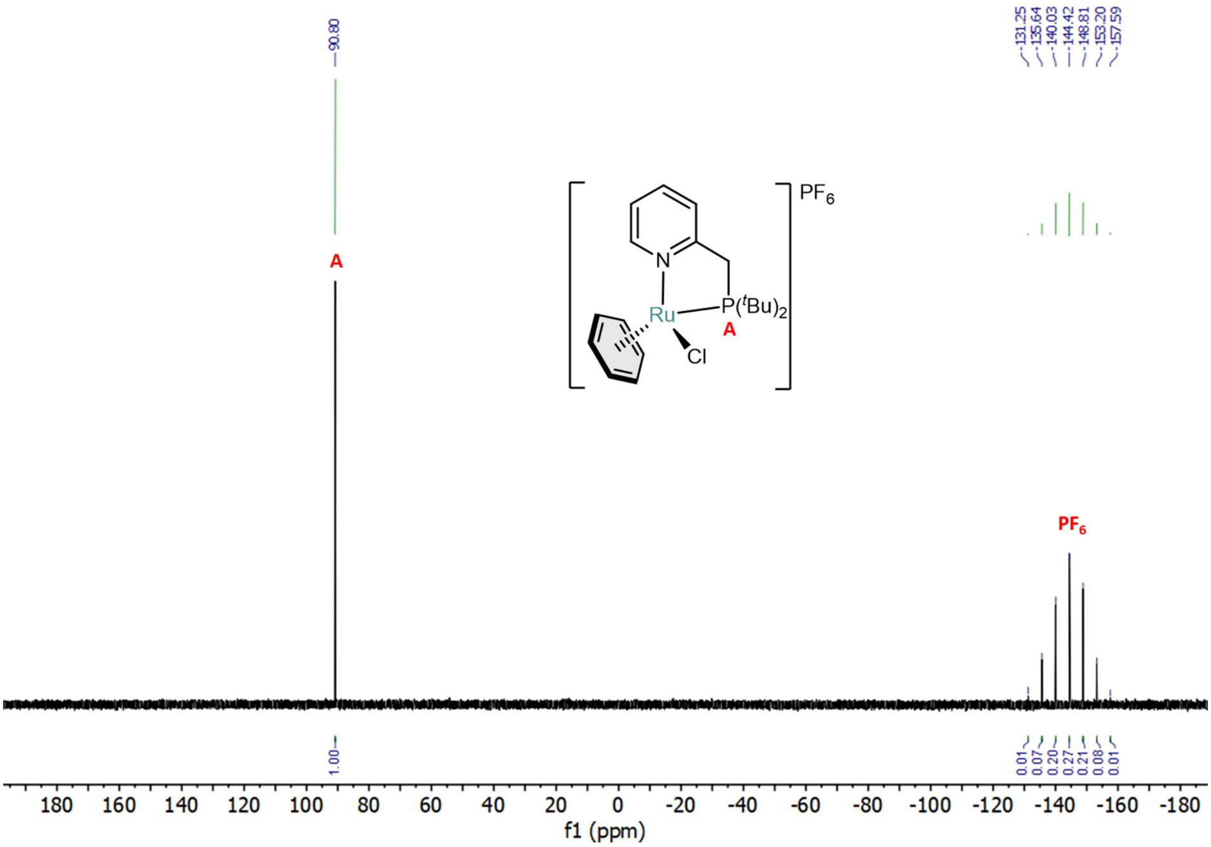

Figure S4. The $^{31}P\{^1H\}$ NMR spectrum of complex **1** in CD_2Cl_2 at 298 K.

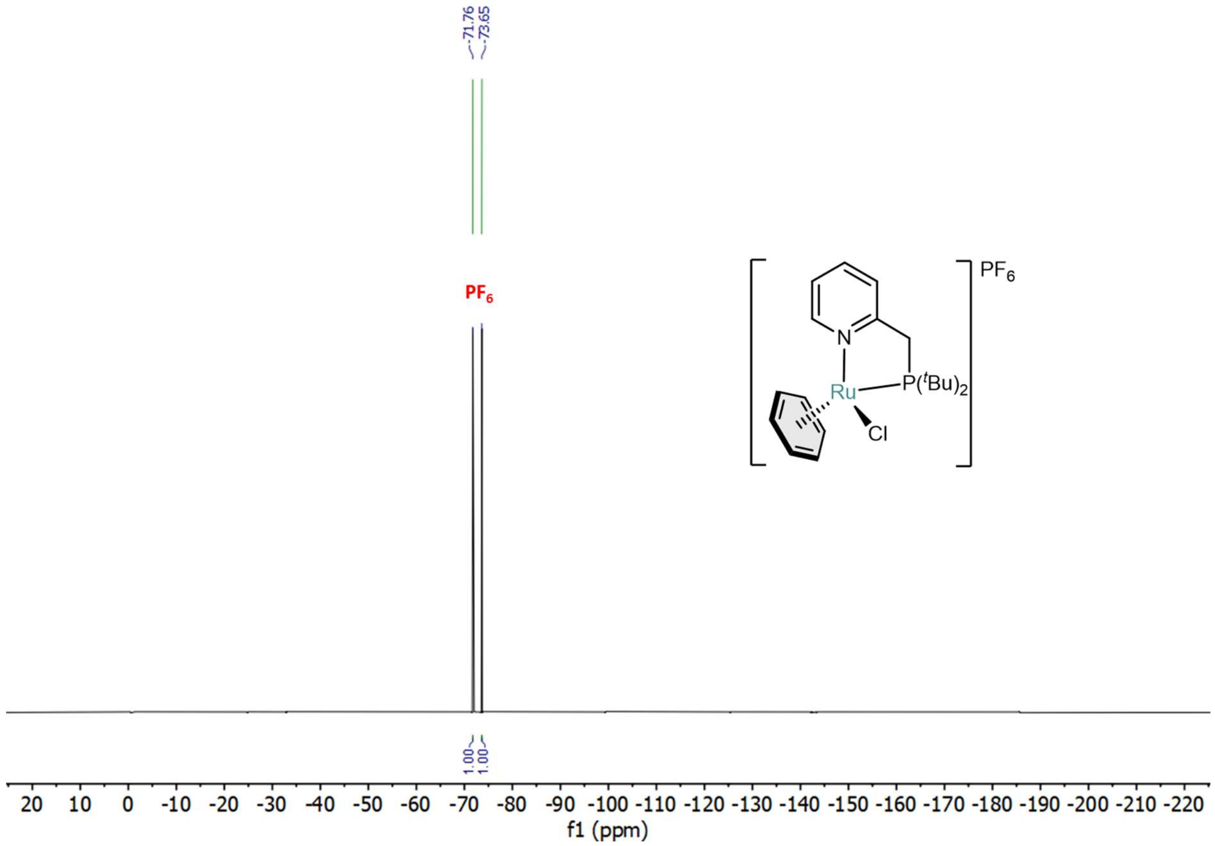

Figure S5. The $^{19}F\{^1H\}$ NMR spectrum of complex **1** in CD_2Cl_2 at 298 K.

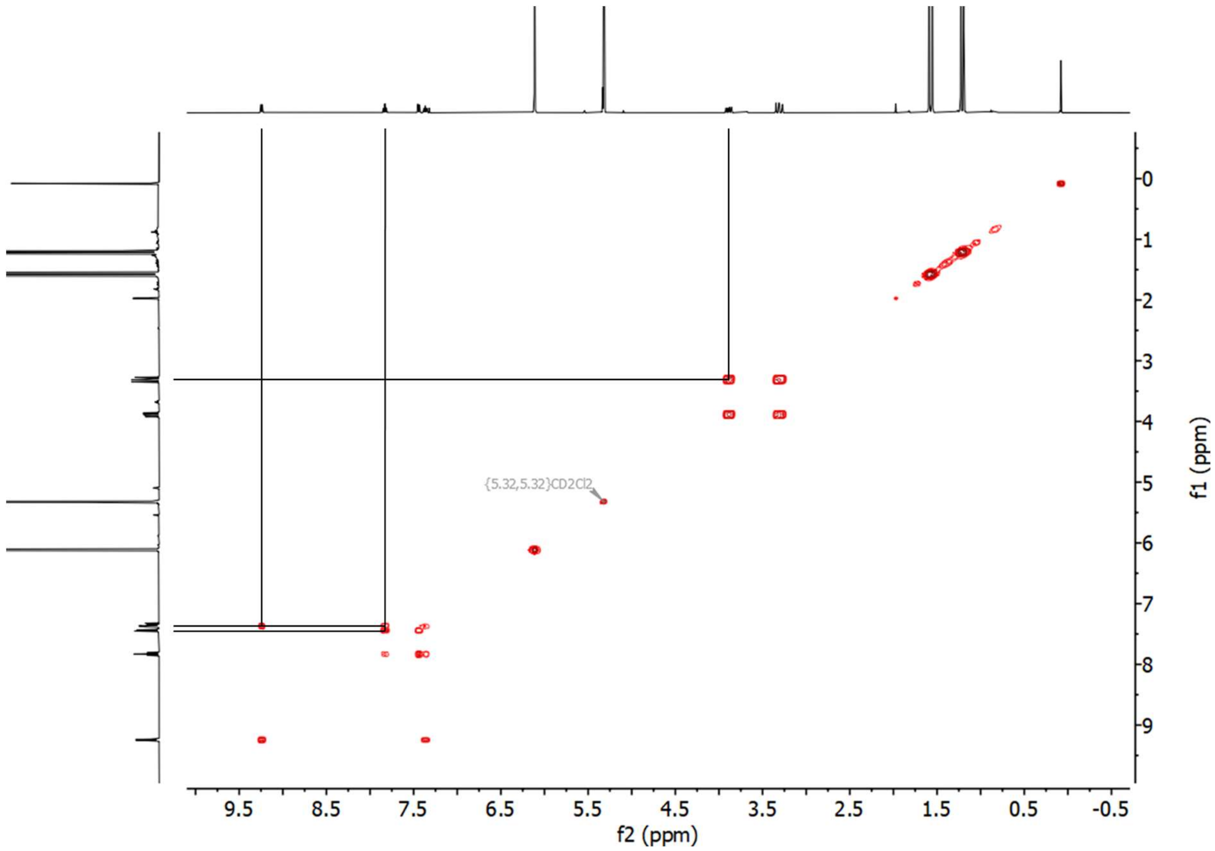

Figure S6. The ^1H-^1H COSY NMR spectrum of complex **1** in CD_2Cl_2 at 298 K.

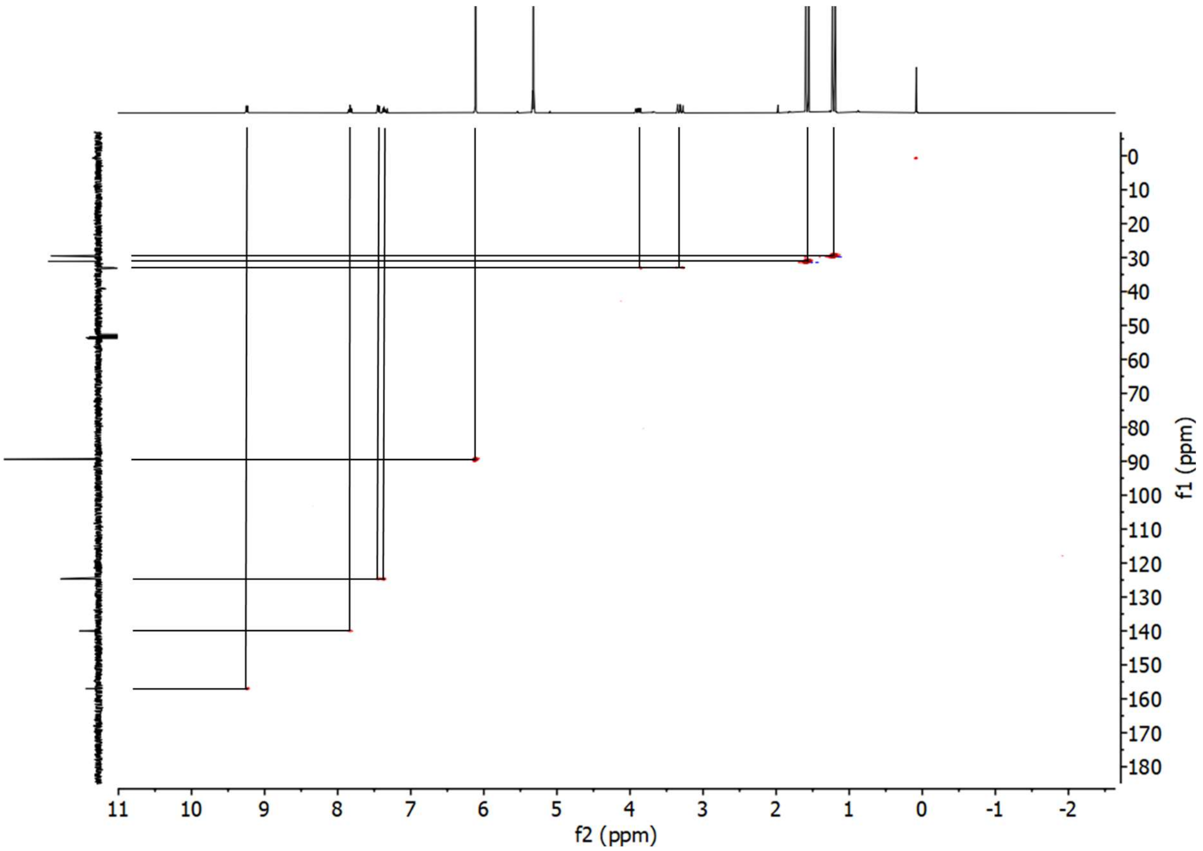

Figure S7. The ^{1}H-^{13}C ASAP-HMQC NMR spectrum of complex **1** in CD_2Cl_2 at 298 K.

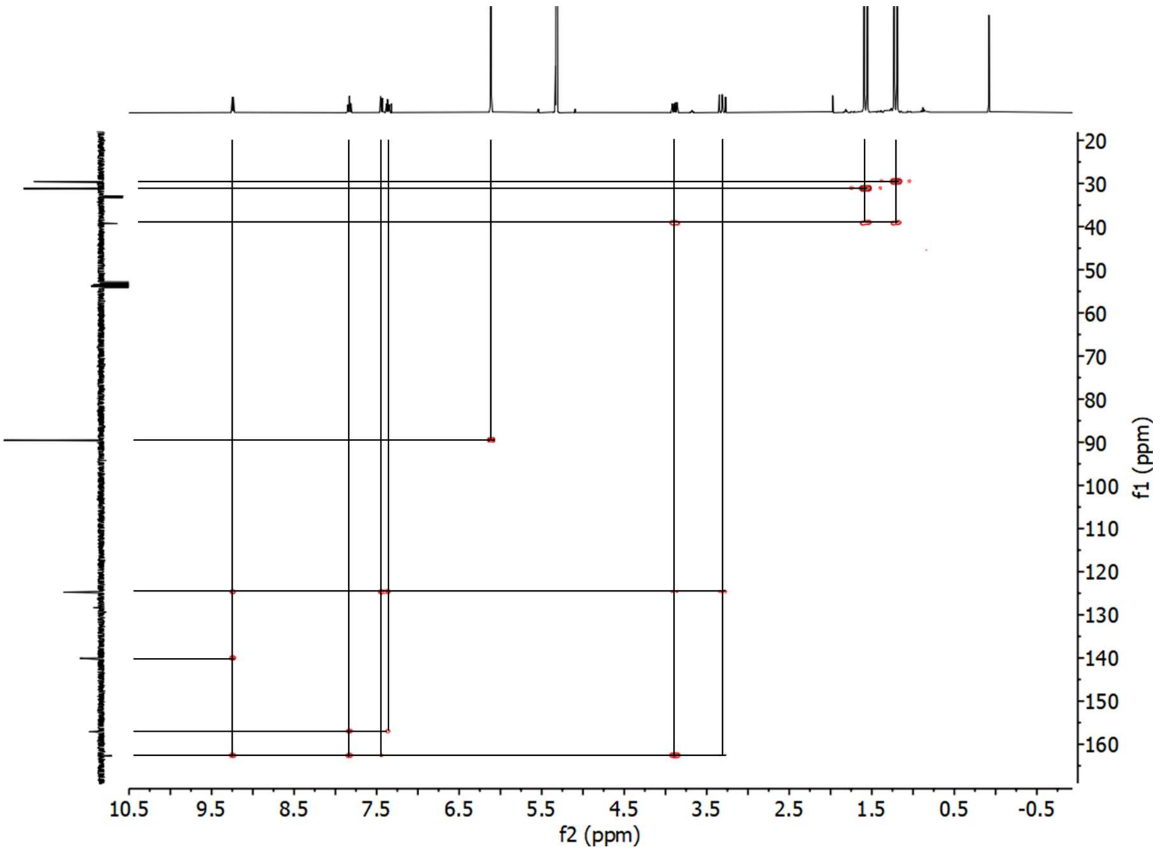

Figure S8. The ^{1}H-^{13}C HMBC NMR spectrum of complex **1** in CD_2Cl_2 at 298 K.

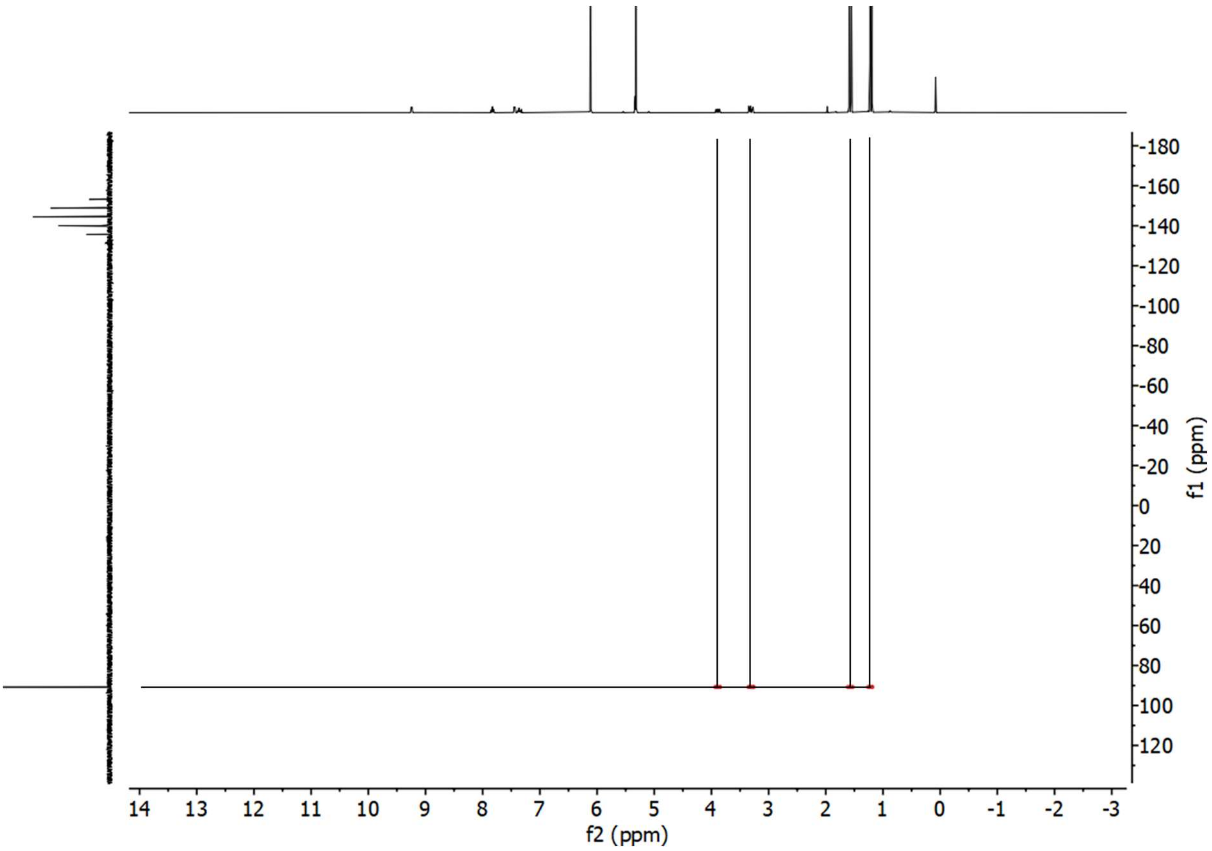

Figure S9. The ^{1}H-^{31}P HMBC NMR spectrum of complex **1** in CD_2Cl_2 at 298 K.

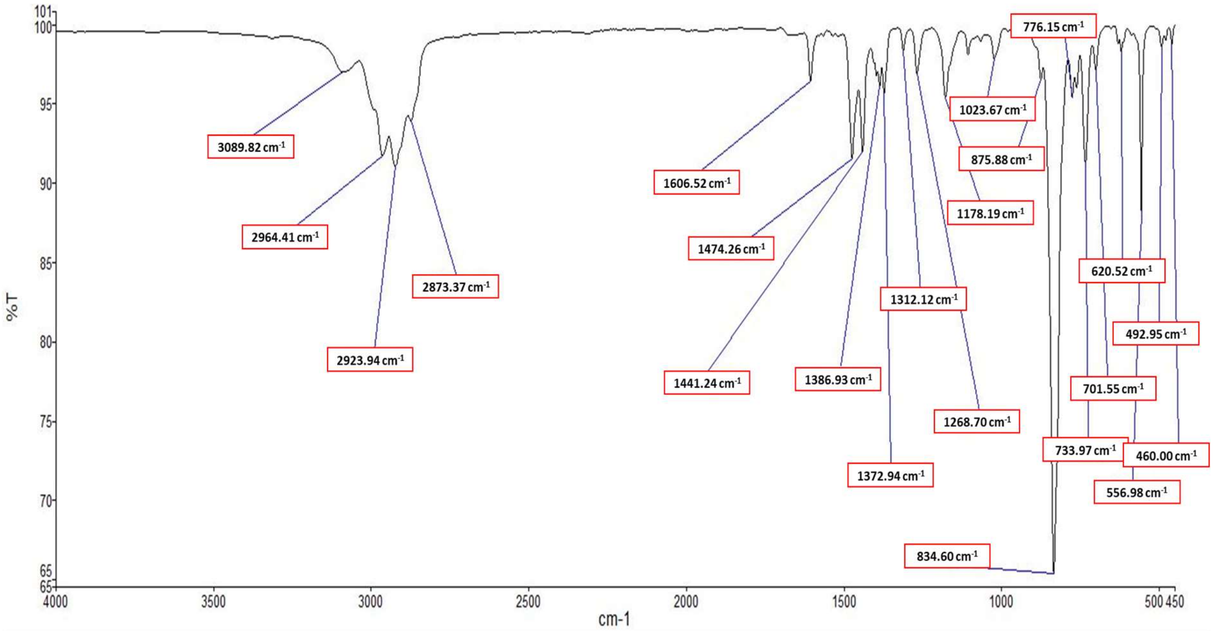

Figure S10. The ATR-IR spectrum of complex **1** measured as a film under N_2 flow at 298 K.

[(^{tBu}PN*)RuCl(C_6H_6)K(THF)$_n$]PF_6 (2-K) and [(^{tBu}PN)RuH(PhN(TMS)$_2$)]PF_6 (3)

A colorless solution of KN(TMS)$_2$ (8.0 mg, 0.04 mmol) in THF-d_8 (1.5 mL) was added dropwise to a yellow suspension of complex **1** (23.9 mg, 0.04 mmol) in THF-d_8 (1.5 mL), resulting in a dark brown solution. The vial with the reaction mixture was kept stirring for 15 min at RT after which a sample was transferred into a J. Young tube and analyzed by NMR spectroscopy.

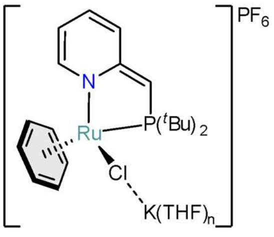

2-K

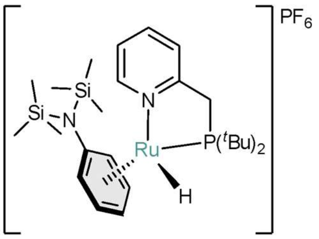

3

*Note: the crude ^{1}H NMR spectrum shows the formation of ~50% species **2-K** and ~20% species **3** based on the relative integral values. For a cleaner synthesis route towards **2-K** as well as an alternative route to the mixture of **2K** and **3** see Supplementary Section S1.2.*

For **2-K**:

^{1}H NMR (400 MHz, THF-*d*$_8$, 298 K): δ = 8.21 (ddd, *J* = 5.3, 1.6, 1.2 Hz, 1H), 7.62 (ddd, *J* = 7.9, 7.9, 1.5 Hz, 1H), 7.06 (dddd, *J* = 7.8, 5.3, 1.3, 1.3 Hz, 1H), 6.72 (dd, *J* = 7.9, 1.0 Hz, 1H), 5.82 (s, 6H), 3.33 (d, $^2J_{H,P}$ = 3.0 Hz, 1H), 1.36 (d, $^3J_{H,P}$ = 14.9 Hz, 9H), 1.08 (d, $^3J_{H,P}$ = 15.4 Hz, 9H).

^{31}P{^{1}H} NMR (162 MHz, THF-*d*$_8$, 298K): δ = 97.5 (s, 1P), –144.5 (hept, $^1J_{P,F}$ = 710.1 Hz, 1P).

For **3**:

^{1}H NMR (400 MHz, THF-*d*$_8$, 298 K): δ = 8.86 (d, *J* = 5.4 Hz, 1H), 7.71 (t, *J* = 8.3 Hz, 1H), 7.51 (d, *J* = 7.6 Hz, 1H), 7.14 (dd, *J* = 7.6, 6.7 Hz, 1H), 6.27 (t, *J* = 6.2 Hz, 1H), 6.21 (d, *J* = 5.5 Hz, 1H), 5.69 (dd, *J* = 6.2, 1.5 Hz, 1H), 5.44 – 5.39 (m, 1H), 4.64 (dt, *J* = 5.9, 1.6 Hz, 1H), 3.64 – 3.56 (m, 1H, overlapped with a THF signal), 3.26 (dd, *J* = 17.2, 7.6 Hz, 1H), 1.34 (d, $^3J_{H,P}$ = 13.7 Hz, 9H), 1.25 (d, $^3J_{H,P}$ = 13.0 Hz, 9H), 0.30 (s, 18H), –7.77 (d, *J* = 42.5 Hz, 1H).

^{31}P{^{1}H} NMR (162 MHz, THF-*d*$_8$, 298K): δ = 111.8 (d*, $^2J_{P,H}$ = 11.2 Hz, 1P), –144.5 (hept, $^1J_{P,F}$ = 710.1 Hz, 1P).

**The doublet appears due to partial coupling with the hydride.*

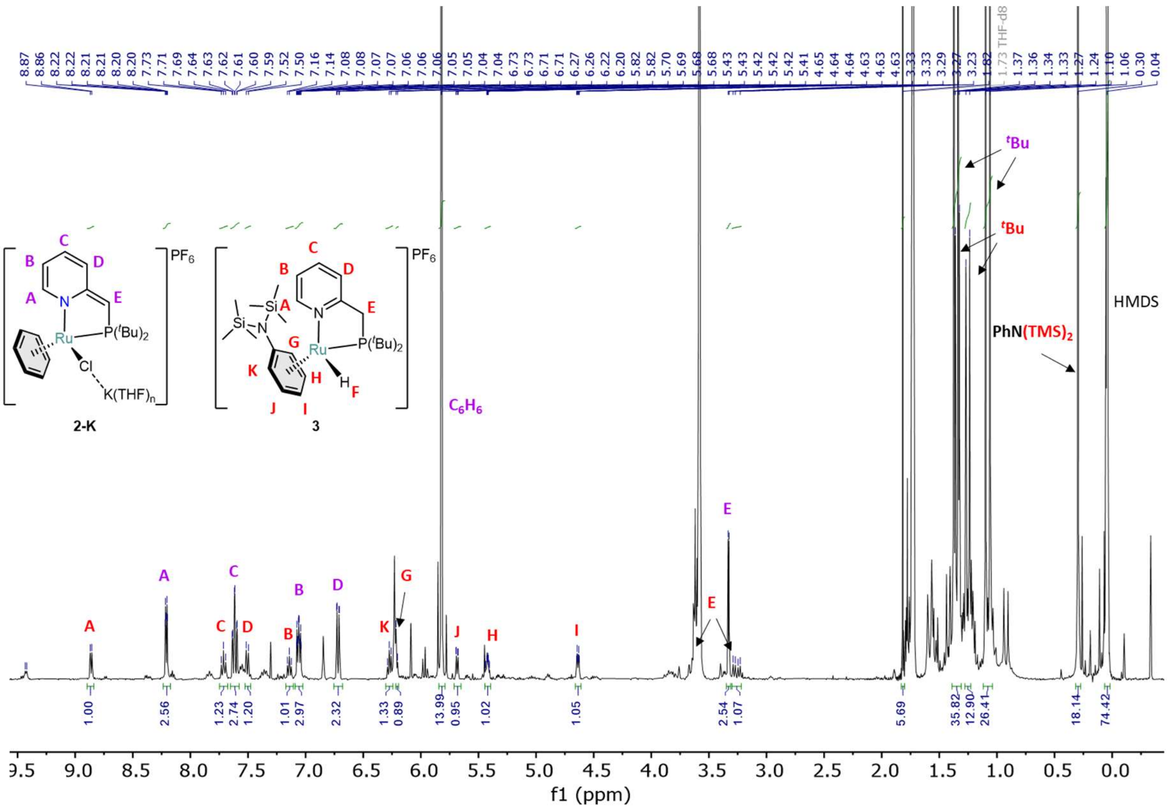

Figure S11. The ^{1}H NMR spectrum of the mixture of **2-K** and **3** in THF-d_8 at 298 K (the positive region of the spectrum).

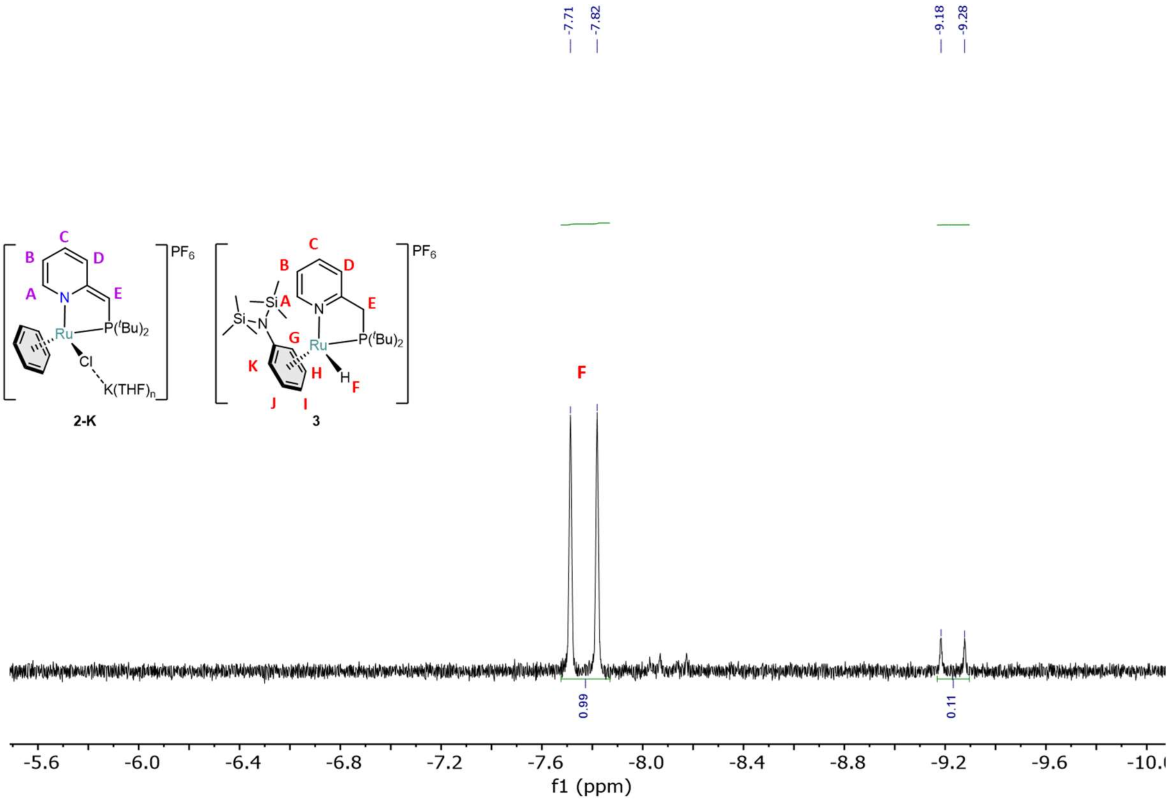

Figure S12. The ^{1}H NMR spectrum of the mixture of **2-K** and **3** in THF-d_8 at 298 K (the negative region of the spectrum).

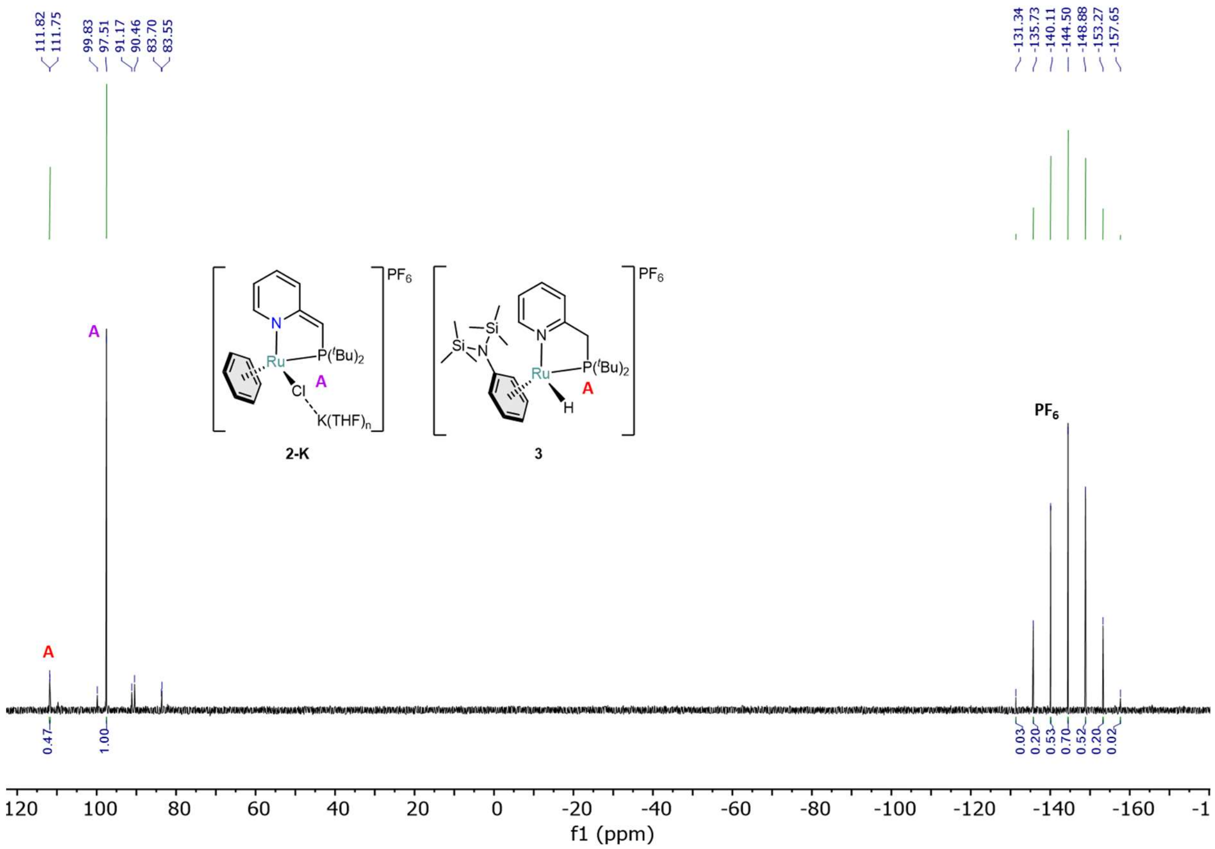

Figure S13. The ^{31}P NMR spectrum of the mixture of **2-K** and **3** in THF-d_8 at 298 K.

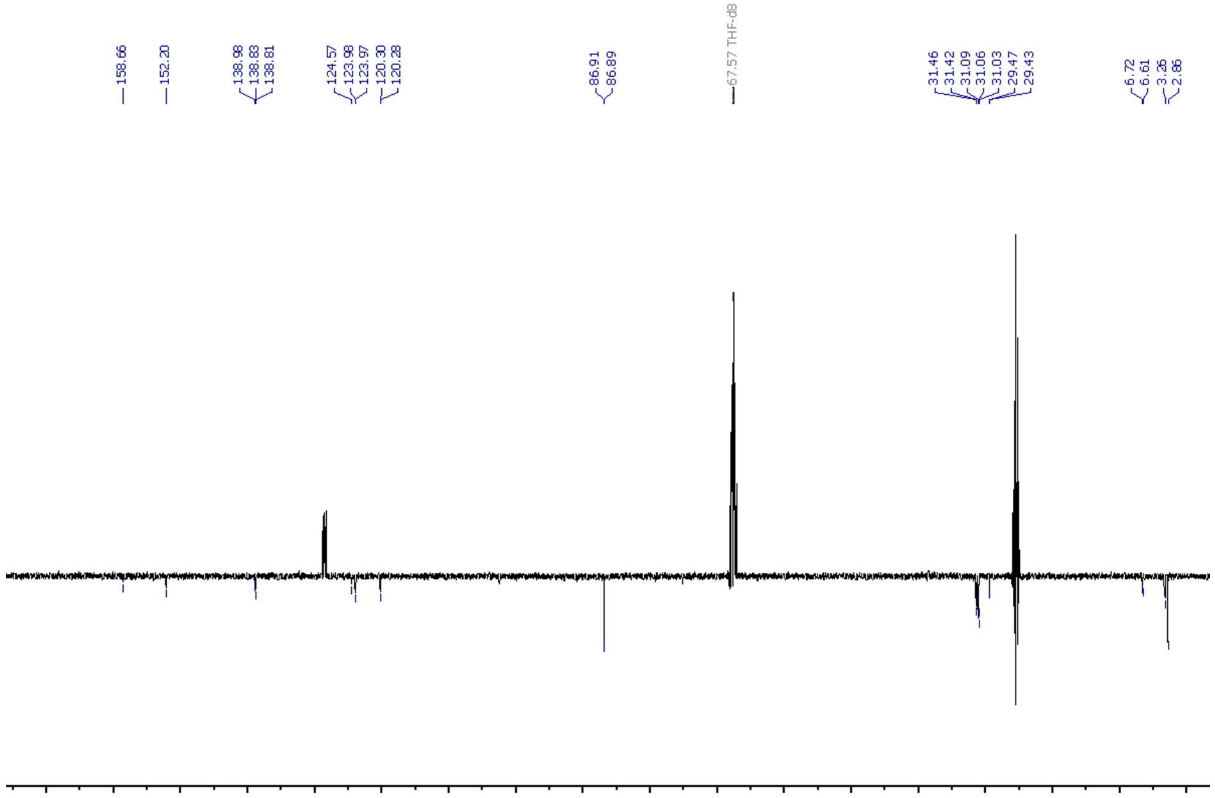

Figure S14. The ^{13}C (APT) NMR spectrum of the mixture of **2-K** and **3** in THF-d_8 at 298 K.

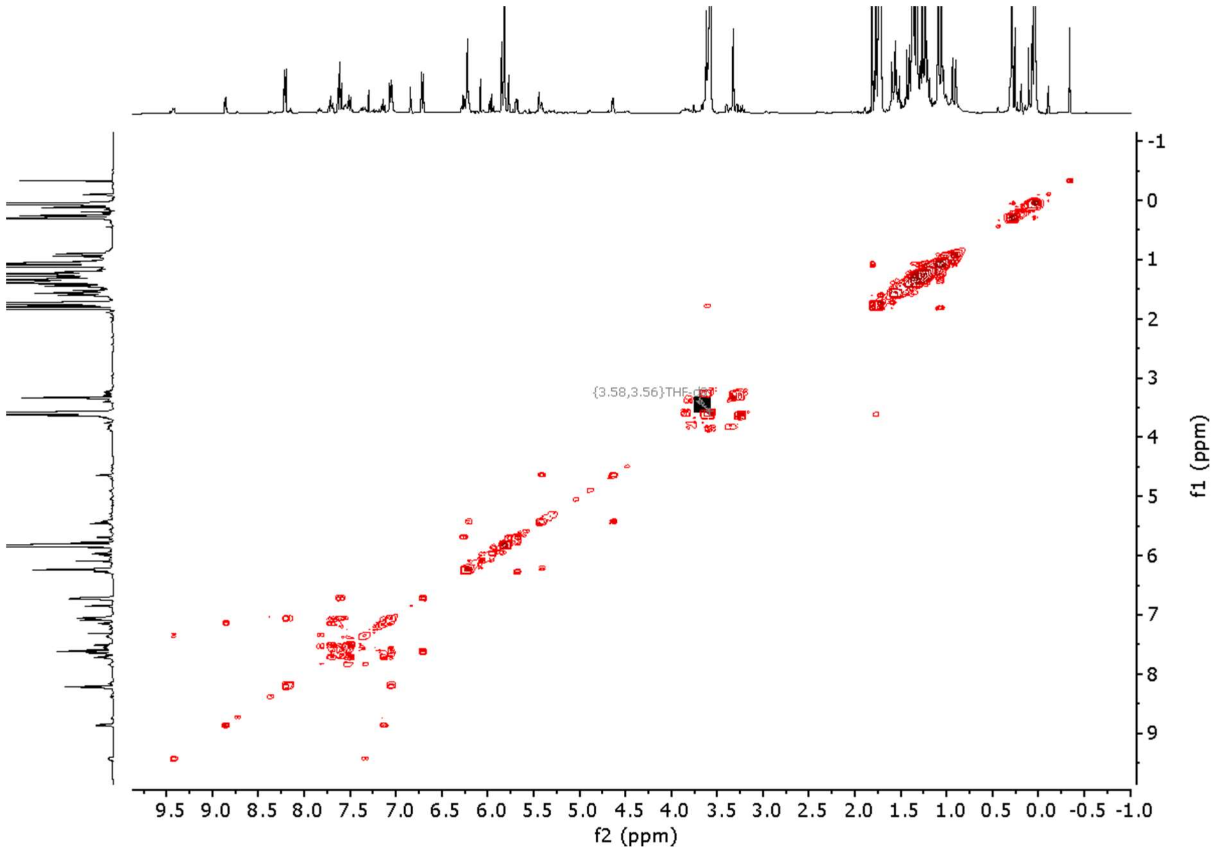

Figure S15. The ^{1}H-^{1}H COSY NMR spectrum of the mixture of **2-K** and **3** in THF-d_8 at 298 K.

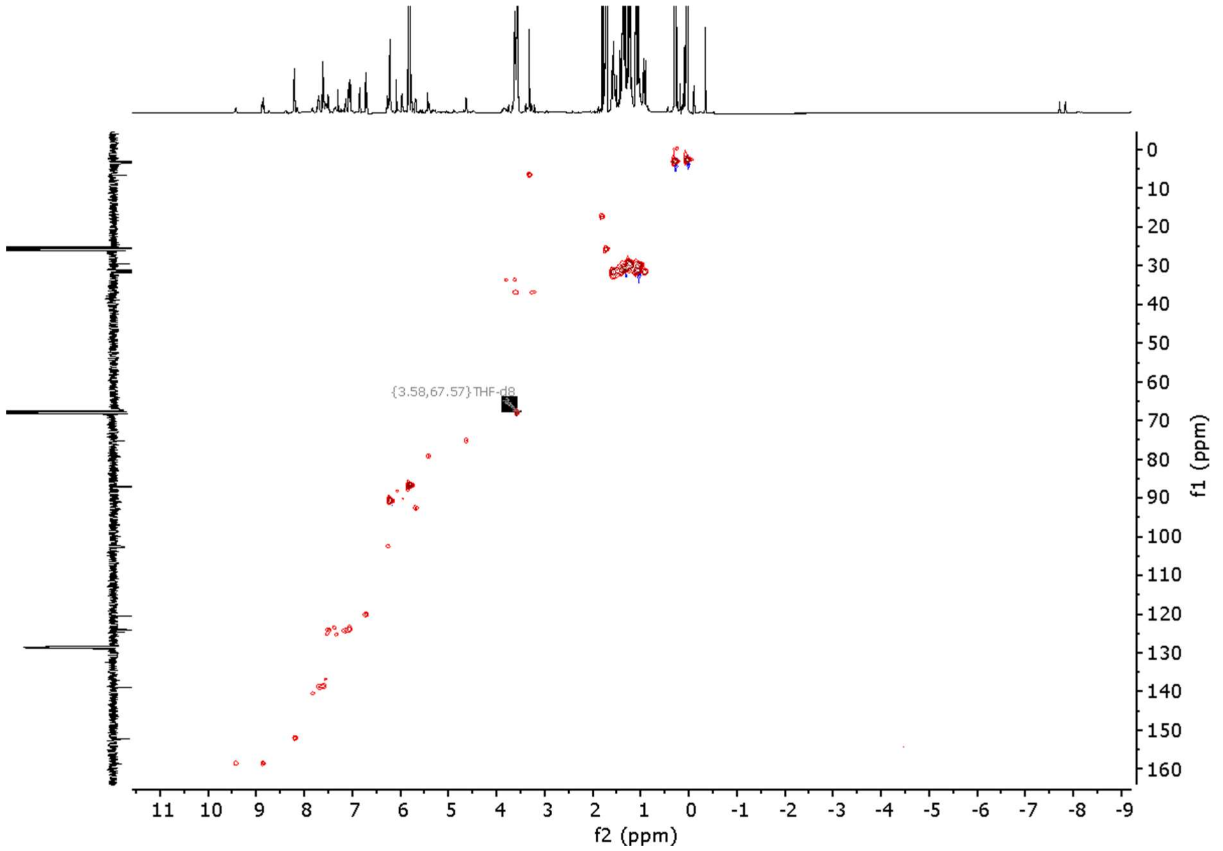

Figure S16. The ^{1}H-^{13}C ASAP-HMQC NMR spectrum of the mixture of **2-K** and **3** in THF-d_8 at 298 K.

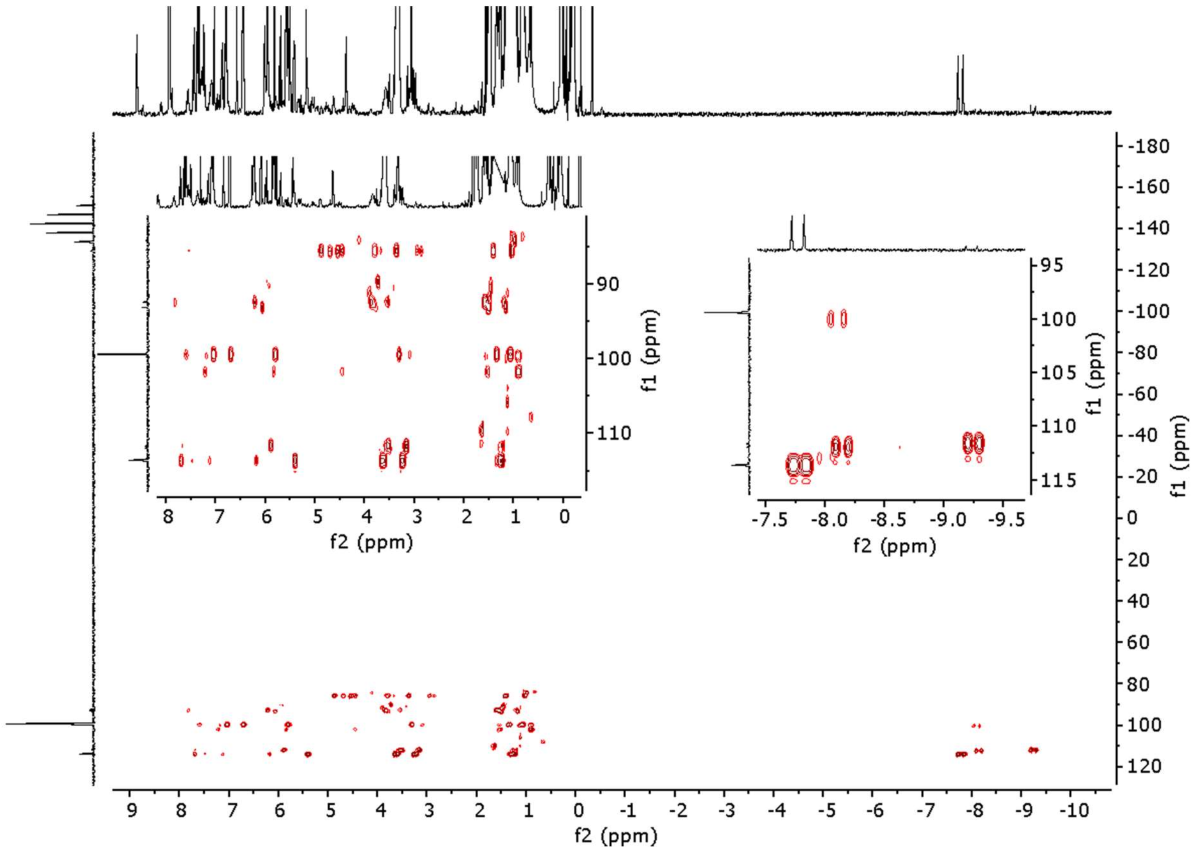

Figure S17. The ^{1}H-^{31}P HMBC NMR spectrum of the mixture of **2-K** and **3** in THF-d_8 at 298 K.

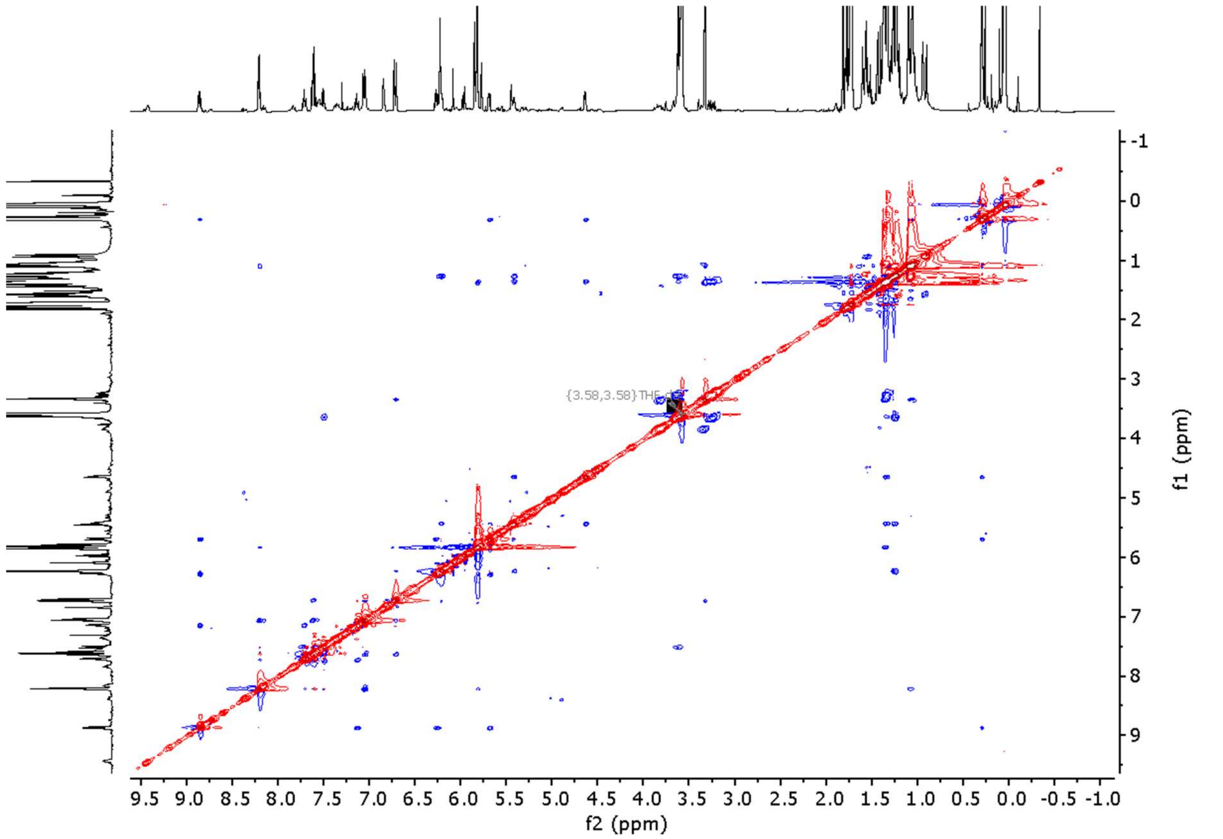

Figure S18. The ^{1}H-^{1}H NOESY NMR spectrum of the mixture of **2-K** and **3** in THF-d_8 at 298 K.

(^{tBu}PN*)RuH(PhN(TMS)$_2$) (4)

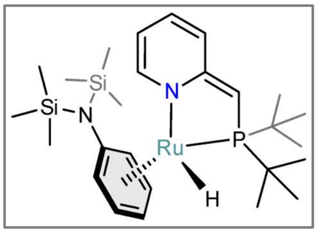

A yellow suspension of complex **1** (96.8 mg, 0.16 mmol) in THF (6.0 mL) was added dropwise to a colorless solution of KN(TMS)$_2$ (64.7 mg, 0.32 mmol) in THF (4.0 mL). The starting complex instantly dissolved upon the addition, resulting in a color change to dark brown. After stirring for 0.5 h the mixture was dried under a dynamic vacuum to give a dark brown solid. The residue was extracted with pentane (5.0 mL), and the extracts were dried under a dynamic vacuum to give a dark brown sticky solid (90.3 mg, 97%). Crystals suitable for X-ray diffraction analysis were grown by keeping a concentrated solution of **4** in pentane at –40 °C.

^{1}H NMR (400 MHz, C$_6$D$_6$, 298 K): δ = 7.33 (dd, $^3J_{H,H}$ = 6.3, $^4J_{H,H}$ = 0.9 Hz, 1H), 6.53 (dddd, $^3J_{H,H}$ = 9.0, $^3J_{H,H}$ = 6.3, $^5J_{H,P}$ = 2.1, $^4J_{H,H}$ = 1.4 Hz, 1H), 6.41 (d, $^3J_{H,H}$ = 8.8 Hz, 1H), 5.38 (ddd, $^3J_{H,H}$ = 7.1, $^3J_{H,H}$ = 6.3, $^4J_{H,H}$ = 1.4 Hz, 1H), 5.11 (dd, $^3J_{H,H}$ = 6.2, $^3J_{H,H}$ = 5.9 Hz, 1H), 4.94 (dd, $^3J_{H,H}$ = 6.2, $^3J_{H,H}$ = 5.2 Hz, 1H), 4.77 (dd, $^3J_{H,H}$ = 6.0, $^3J_{H,H}$ = 4.9 Hz, 1H), 4.67 (dd, $^3J_{H,H}$ = 6.0, $^3J_{H,H}$ = 1.9 Hz, 1H), 4.14 (ddd, $^3J_{H,H}$ = 5.7, $^4J_{H,H}$ = 2.2, $^4J_{H,H}$ = 1.8 Hz, 1H), 3.49 (d, $^2J_{H,P}$ = 2.6 Hz, 1H), 1.31 (d, $^3J_{H,P}$ = 12.4 Hz, 9H), 1.27 (d, $^3J_{H,P}$ = 13.2 Hz, 9H), 0.25 (s, 18H), –7.76 (d, $^2J_{H,P}$ = 43.4 Hz, 1H).

^{13}C{^{1}H} NMR (100 MHz, C$_6$D$_6$, 298 K) δ = 170.9 (d, $^2J_{C,P}$ = 15.6 Hz), 154.4 (s), 130.8 (d, $^4J_{C,P}$ = 2.3 Hz), 130.7 (s), 115.2 (d, $^3J_{C,P}$ = 17.2 Hz), 101.4 (s), 92.4 (s), 90.6 (d, $^2J_{C,P}$ = 5.7 Hz), 83.4 (s), 77.4 (s), 74.1 (d, $^2J_{C,P}$ = 3.1 Hz), 62.3 (d, $^1J_{C,P}$ = 60.3 Hz), 38.3 (d, $^1J_{C,P}$ = 14.5 Hz), 36.2 (d, $^1J_{C,P}$ = 34.3 Hz), 31.1 (d, $^2J_{C,P}$ = 3.4 Hz), 30.2 (d, $^2J_{C,P}$ = 5.0 Hz), 3.3 (s).

^{31}P{^{1}H} NMR (162 MHz, C$_6$D$_6$, 298 K): δ = 98.8 (s).

ATR-IR (film, N$_2$ flow): ν = 3045 (m), 2954 (s), 2864 (s), 2893 (m), 2034 (w, br), 1604 (s), 1535 (w), 1511 (w), 1488 (s), 1446 (s), 1381 (w), 1359 (w), 1358 (w), 1285 (m), 1253 (m), 1225 (m), 1205 (m), 1179 (w), 1146 (w), 1101 (w), 1017 (w), 1000 (m), 933 (m), 892 (s), 840 (m), 810 (m), 758 (w), 726 (w), 687 (w), 667 (w), 616 (w), 503 (w), 463 (w) cm^{-1}.

*Despite several attempts using spectroscopically pure samples, the reactive nature of **4** precluded obtaining a satisfactory elemental analysis.*

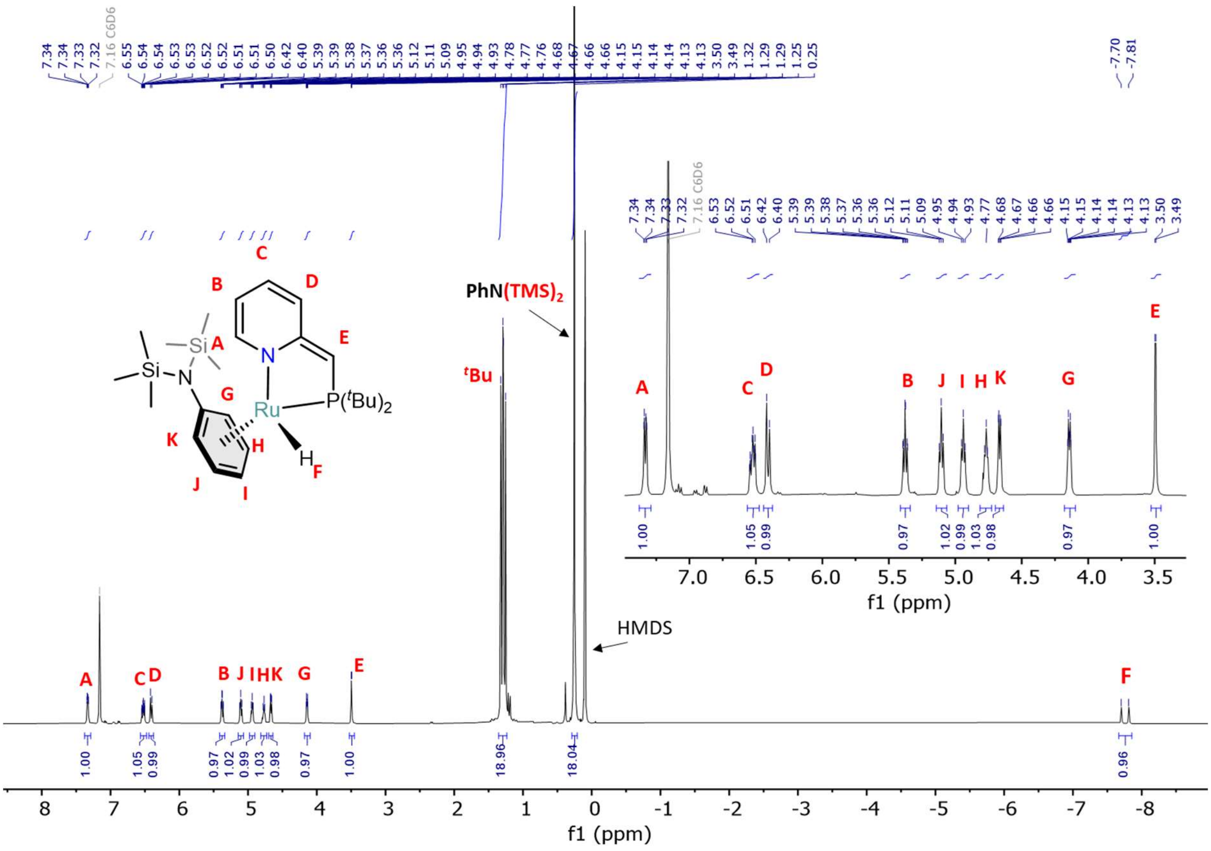

Figure S19. The ^{1}H NMR spectrum of complex **4** in C_6D_6 at 298 K.

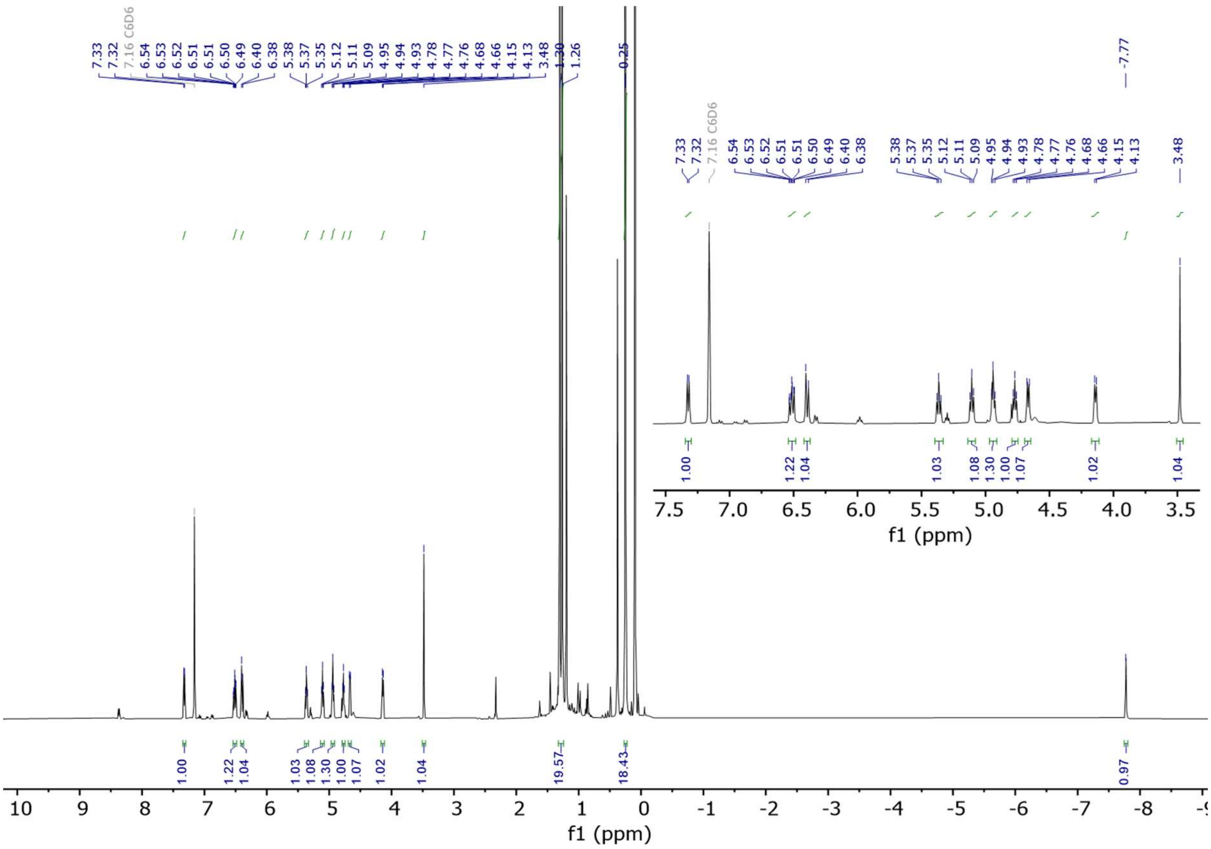

Figure S20. The $^{1}H\{^{31}P\}$ NMR spectrum of complex **4** in C_6D_6 at 298 K.

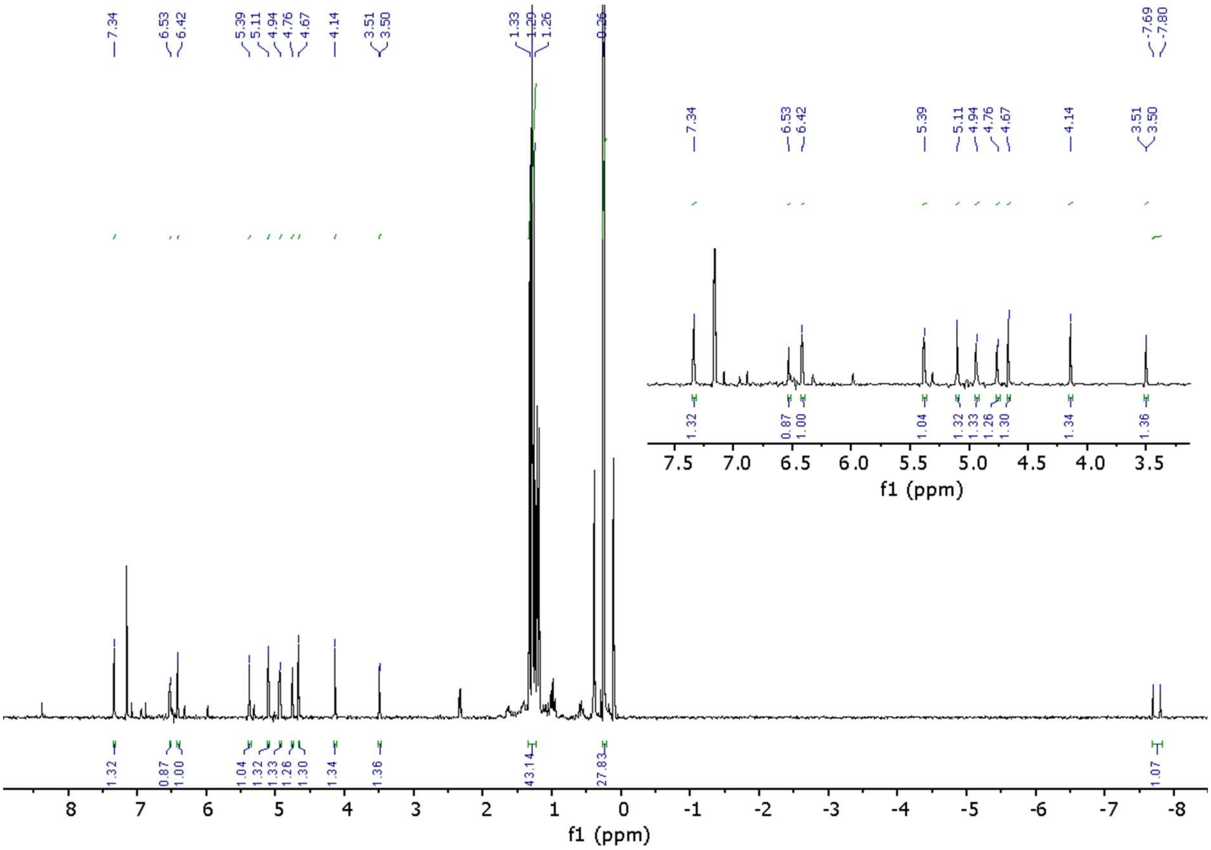

Figure S21. The PSYCHE NMR spectrum of complex **4** in C_6D_6 at 298 K.

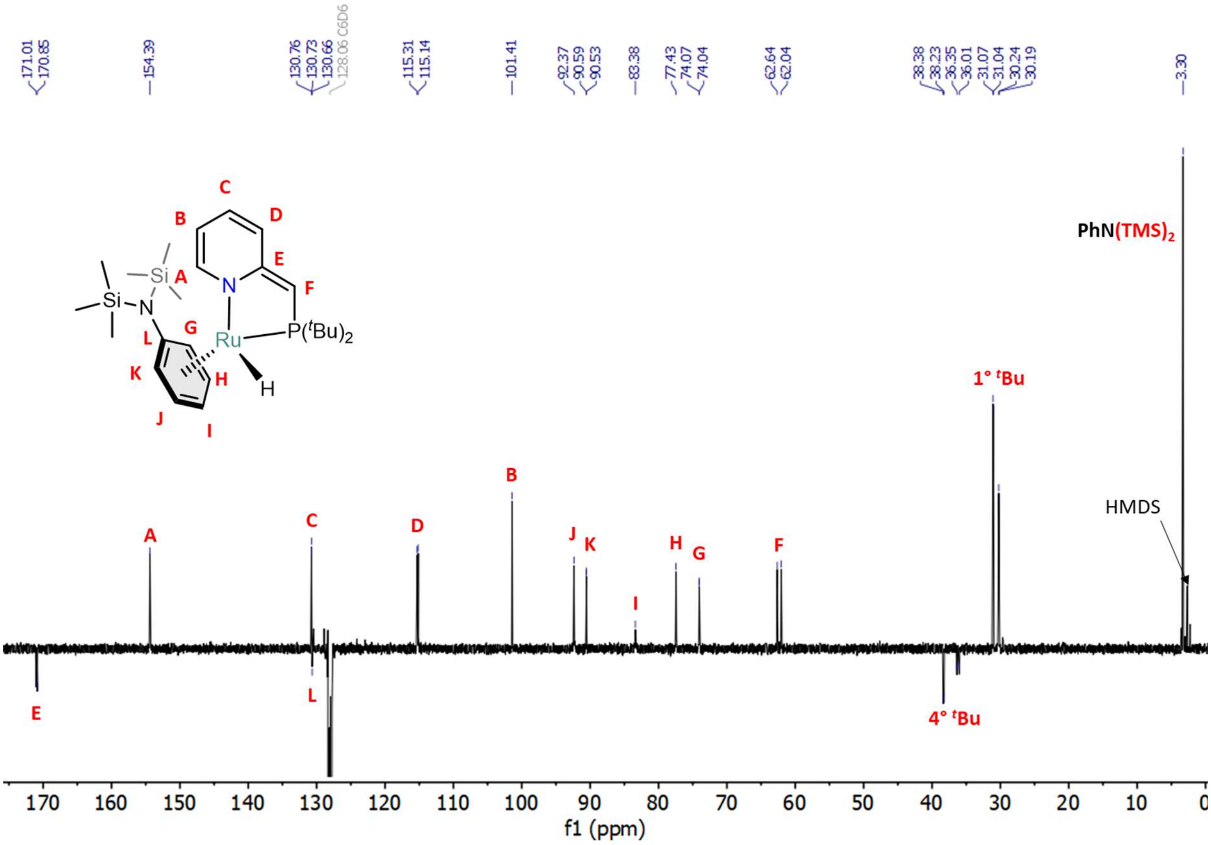

Figure S22. The ^{13}C-APT NMR spectrum of complex **4** in C_6D_6 at 298 K.

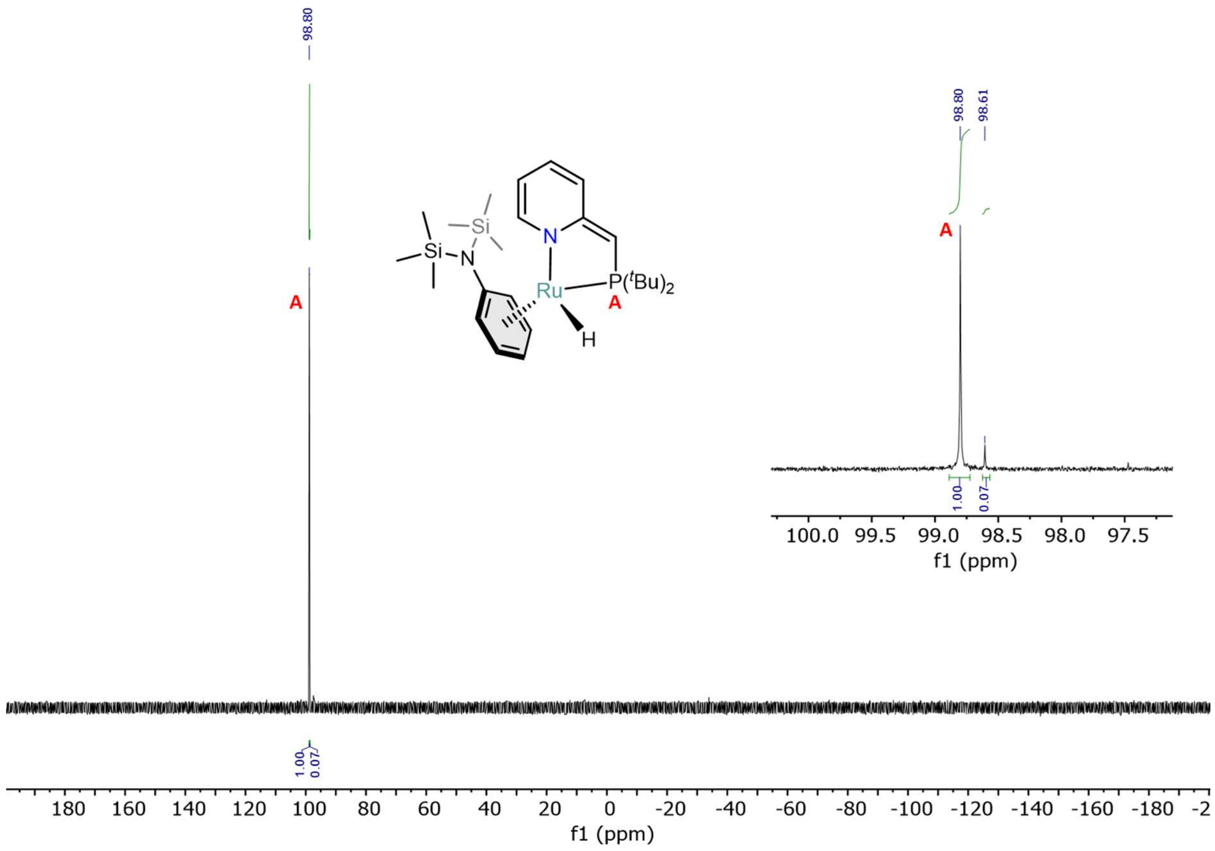

Figure S23. The $^{31}P\{^{1}H\}$ NMR spectrum of complex **4** in C_6D_6 at 298 K.

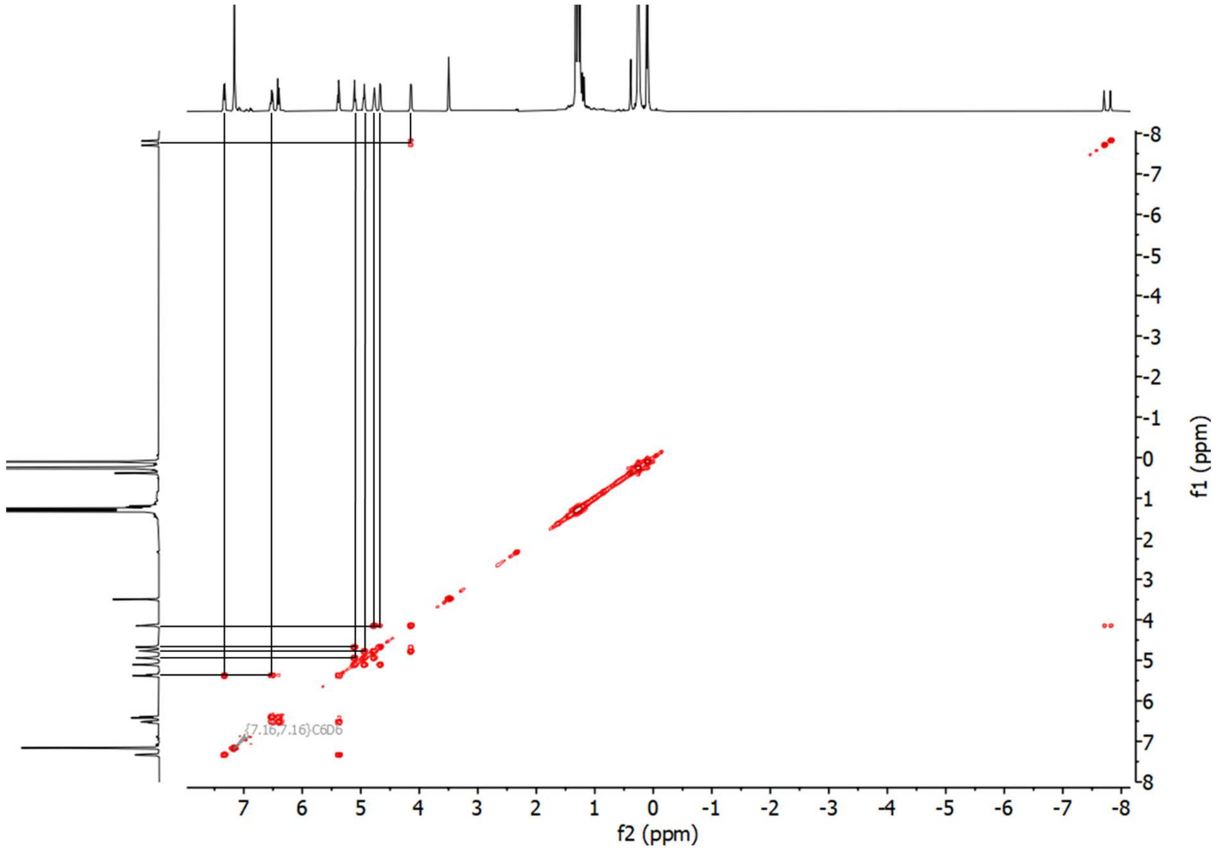

Figure S24. The ^{1}H-^{1}H COSY NMR spectrum of complex **4** in C_6D_6 at 298 K.

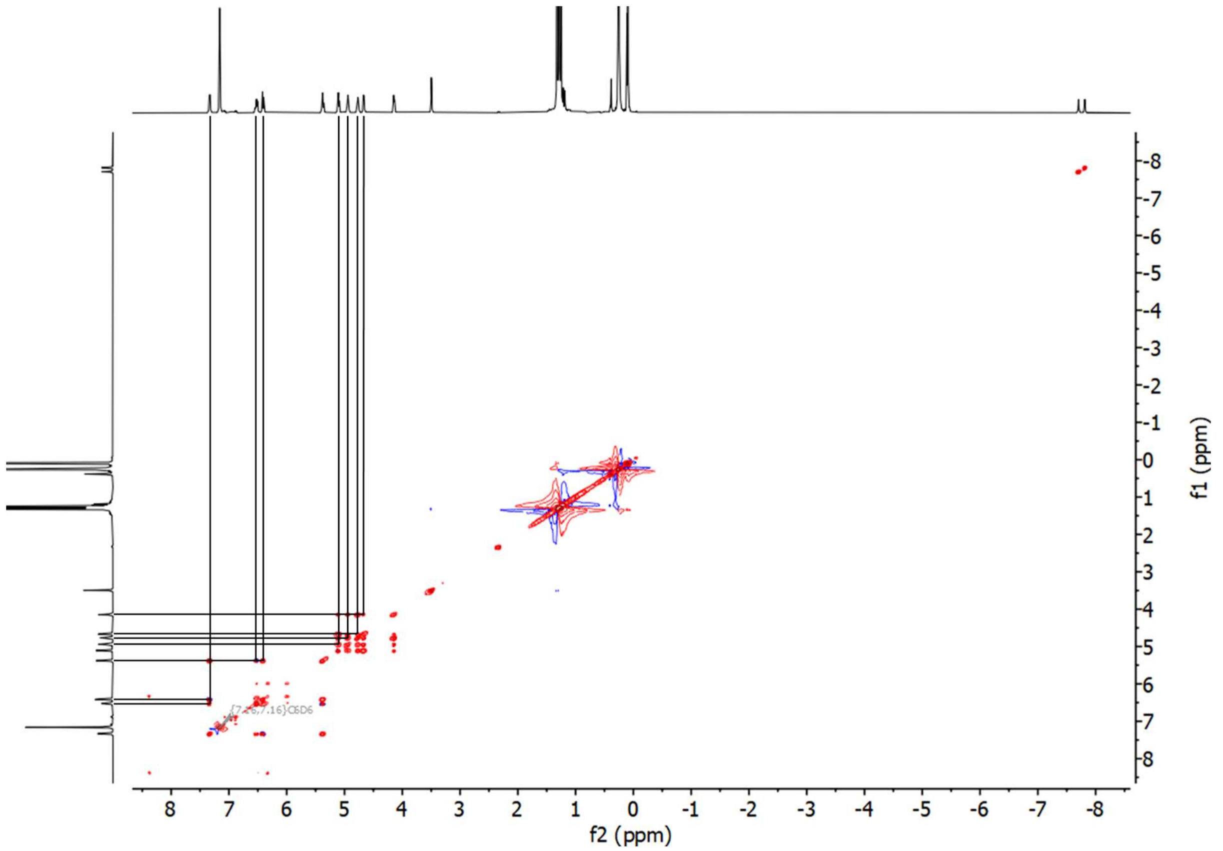

Figure S25. The ^{1}H-^{1}H TOCSY NMR spectrum of complex **4** in C_6D_6 at 298 K.

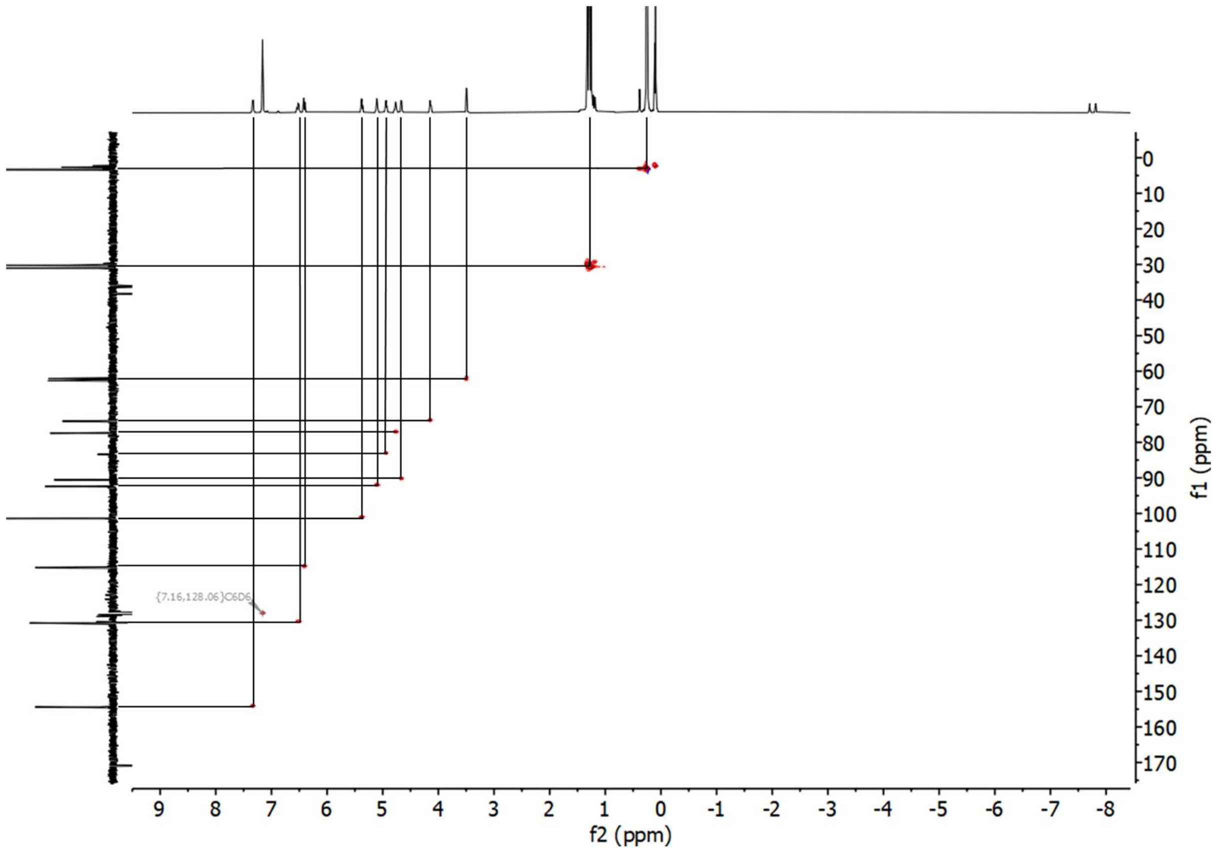

Figure S26. The ^{1}H-^{13}C ASAP-HMQC NMR spectrum of complex **4** in C_6D_6 at 298 K.

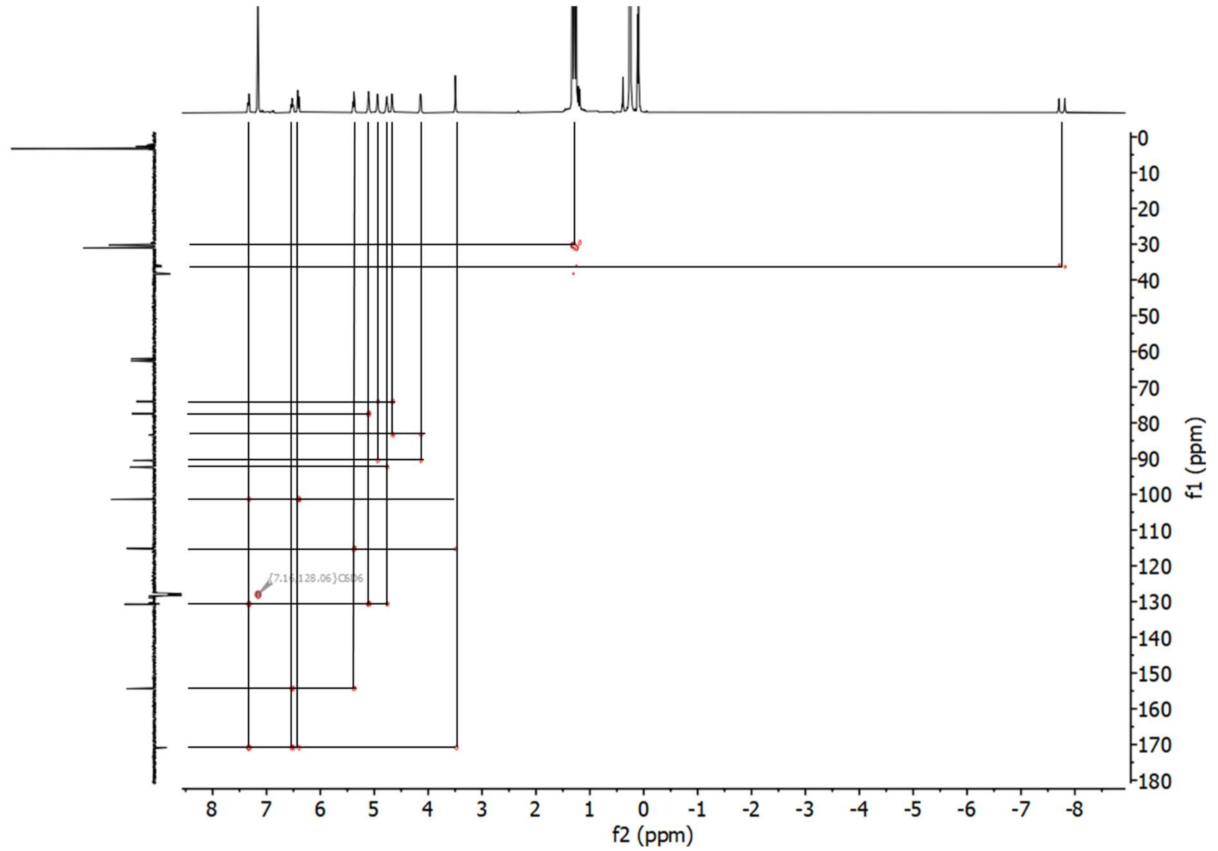

Figure S27. The ^{1}H-^{13}C HMBC NMR spectrum of complex **4** in C_6D_6 at 298 K.

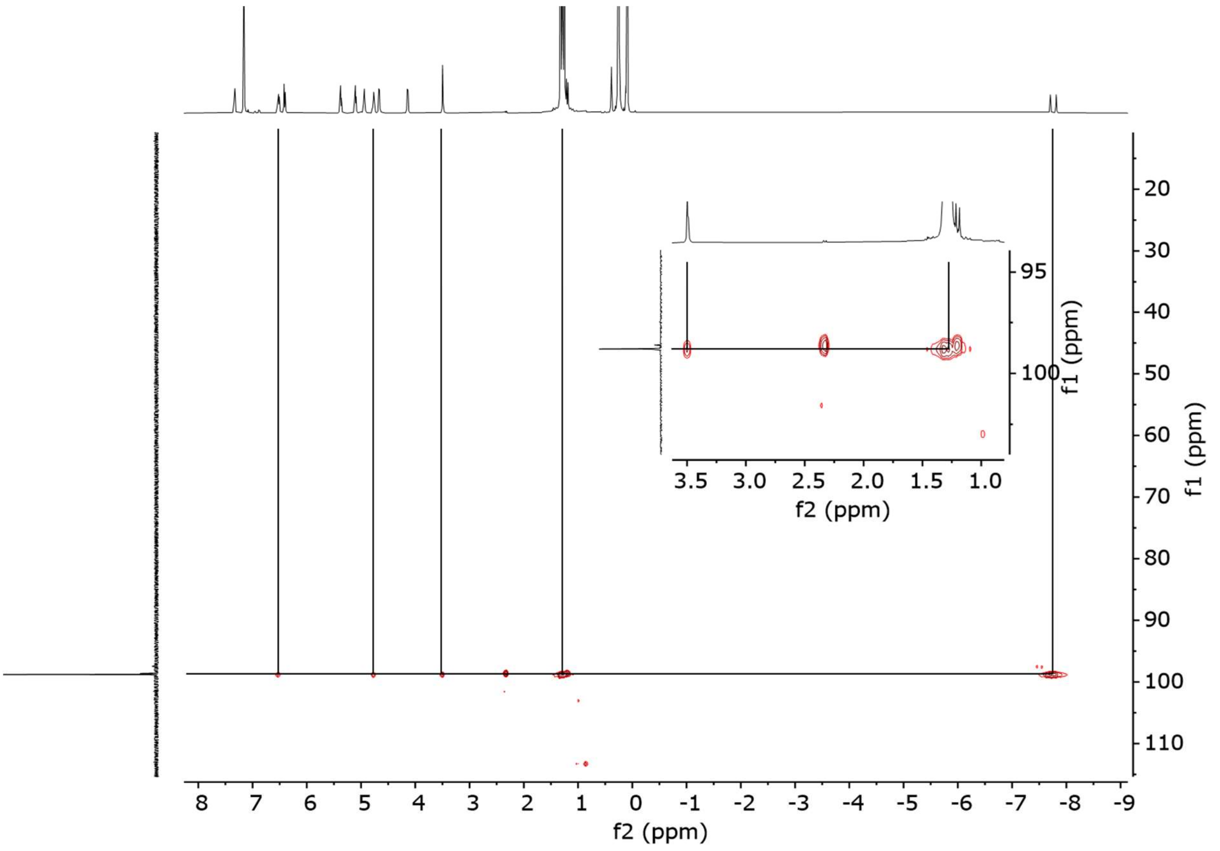

Figure S28. The ^{1}H-^{31}P HMBC NMR spectrum of complex **4** in C_6D_6 at 298 K.

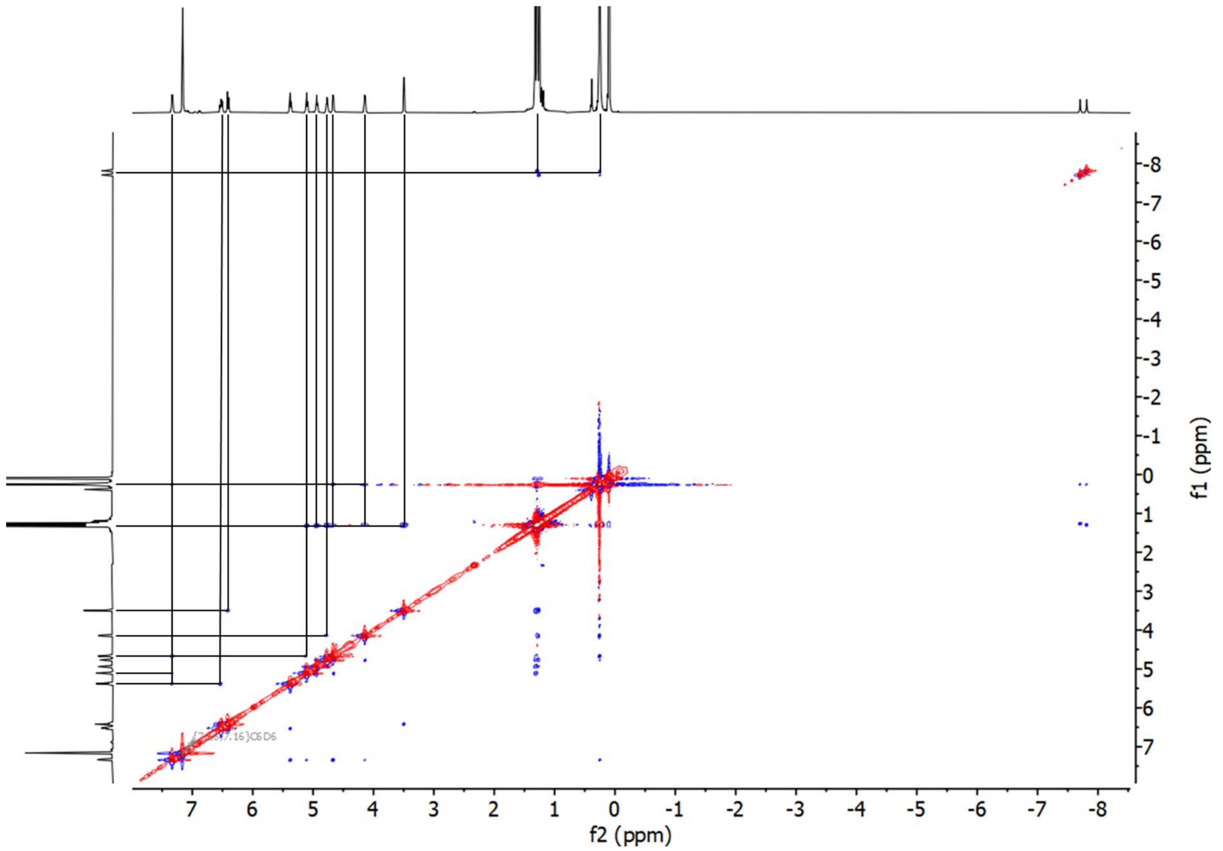

Figure S29. The NOESY NMR spectrum of complex **4** in C_6D_6 at 298 K.

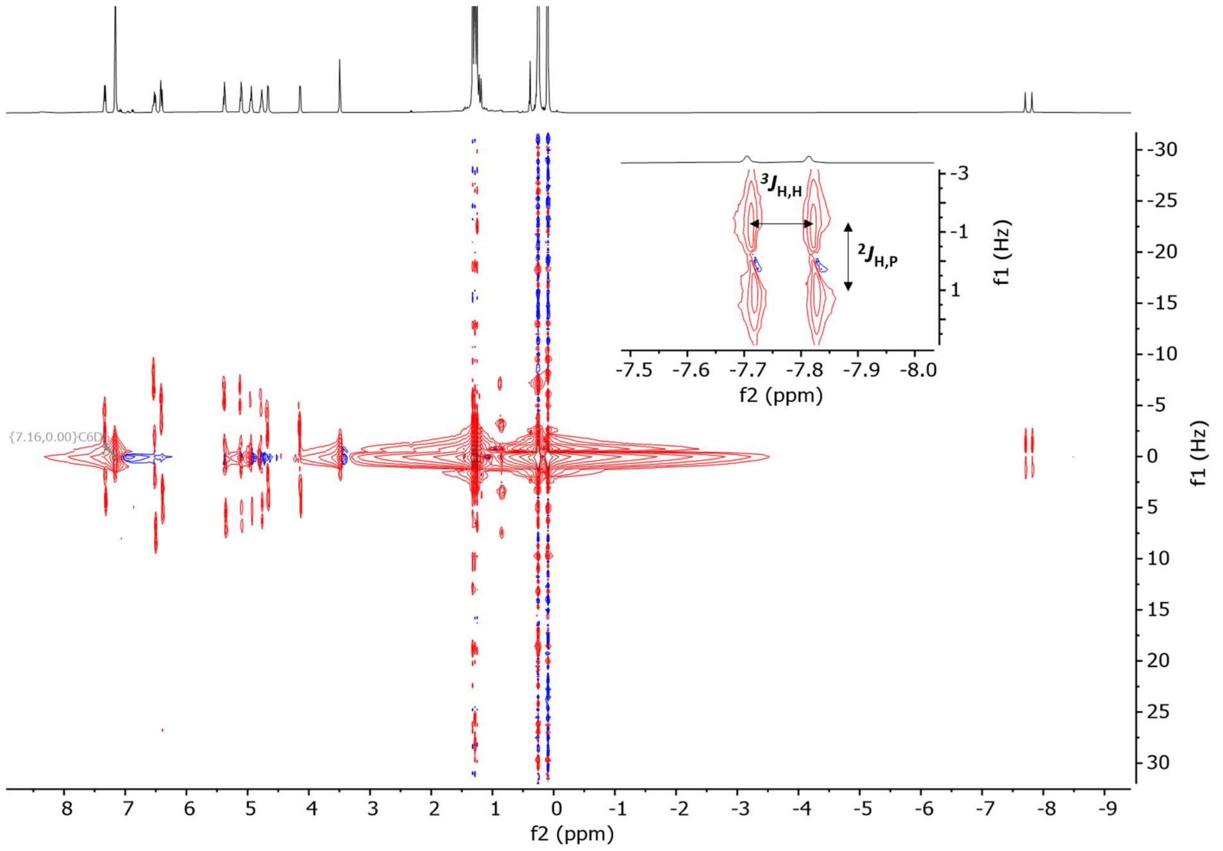

Figure S30. The J-resolved NMR spectrum of complex **4** in C_6D_6 at 298 K.

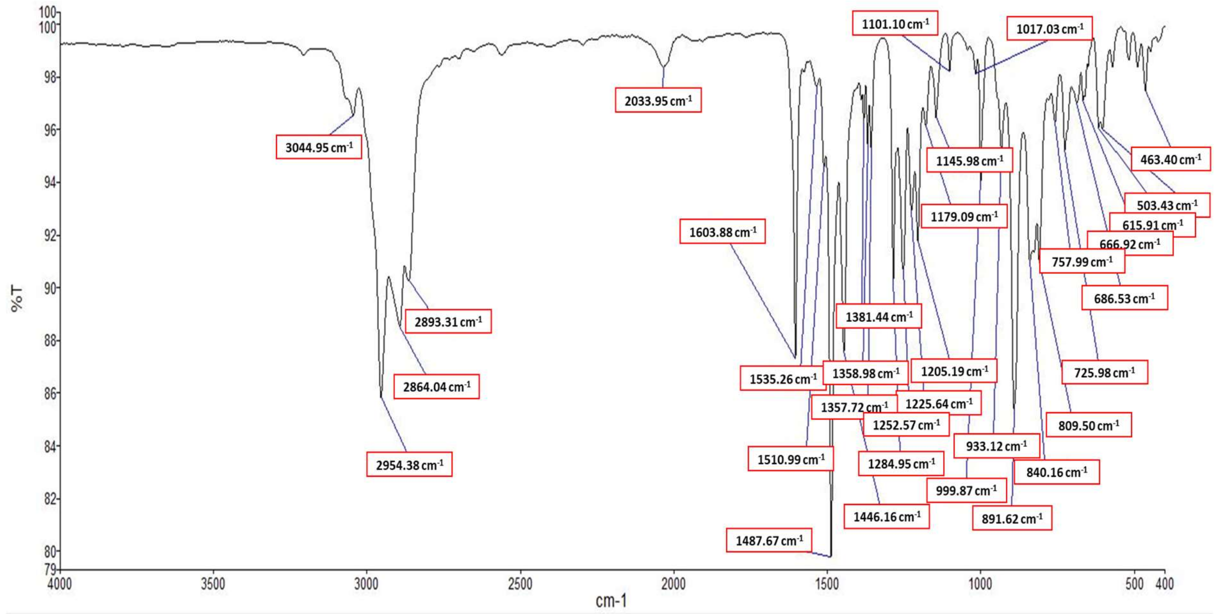

Figure S31. The ATR-IR spectrum of complex **4** measured as a film under N_2 flow at 298 K.

Hexadeuterocyclohexadienes (CHDs)

The modified procedure is based on the ammonia-free method described by the group of Koide.[1]

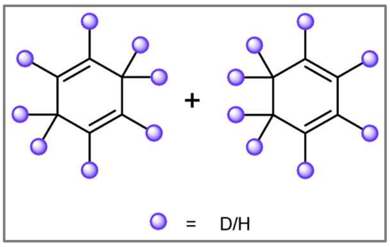

A 250 mL 3-necked round bottom flack equipped with a large stirring bar (vigorous stirring is important after the addition of Li) was charged with C_6D_6 (10.5 ml, 0.12 mol), Et_2O (80.0 mL), EtOD (10.4 mL, 0.18 mol) and ethylenediamine (47.6 mL, 0.71 mol). The reaction mixture was cooled down to 4 °C (external temperature) using an ice bath and the flask was connected to N_2 flow. Next, a block of Li metal (2.47 g, 0.36 mol) was wiped with filter paper to remove the protective oil layer, after which it was cut into 3 small pieces that were added to the reaction mixture against an N_2 flow. The mixture was vigorously stirred for 1 h and 15 min. After 25 min the color changed to brown (Li pieces took time to fully dissolve) and after 45 min a significant amount of white precipitate formed. The reaction was carefully quenched by slow dropwise addition of D_2O (20.0 mL) keeping the flask in the ice bath and stirring for 15 min (**CAUTION**: the quenching releases a large volume of hydrogen gas). The color changed to white, and a large quantity of precipitate formed. H_2O (20.0 mL) was added, and the product was extracted with Et_2O (3 x 15.0 mL). The organic fractions were combined and washed with a brine:water mixture (1:1) (2 x 15.0 mL), and subsequently the majority of Et_2O was distilled off with a long Vigreux column. Triglyme (7.0 mL) was added, and the distillation was continued with a short Vigreux column to give a colorless mixture of deuterated 1,2- and 1,4-cyclohexadienes and cyclohexene at 70-75 °C (1.2 mL).

The mixture was used further without extra purification, nor a more detailed analysis of deuteration.

NB: *The previously reported method*[2] *with the use of liquid ammonia for octadeutero-1,4-cyclohexadiene in our hands gave also an incomplete deuteration as a sole product (hexadeutero-1,4-cyclohexadiene). This result is consistent with the findings of the group of Holland*[3] *that show that complete deuteration can be achieved via using* ND_3.

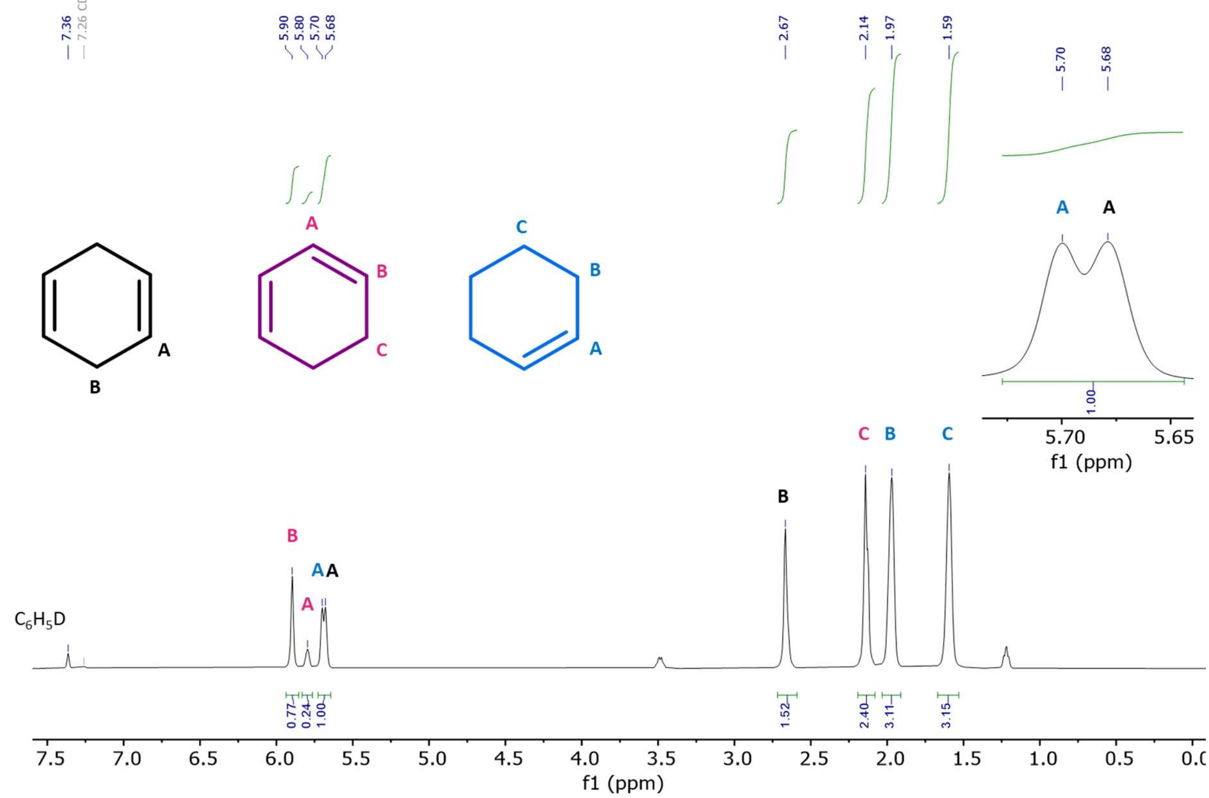

Figure S32. The ^{1}H NMR spectrum of the mixture of **CHDs** at 298 K.

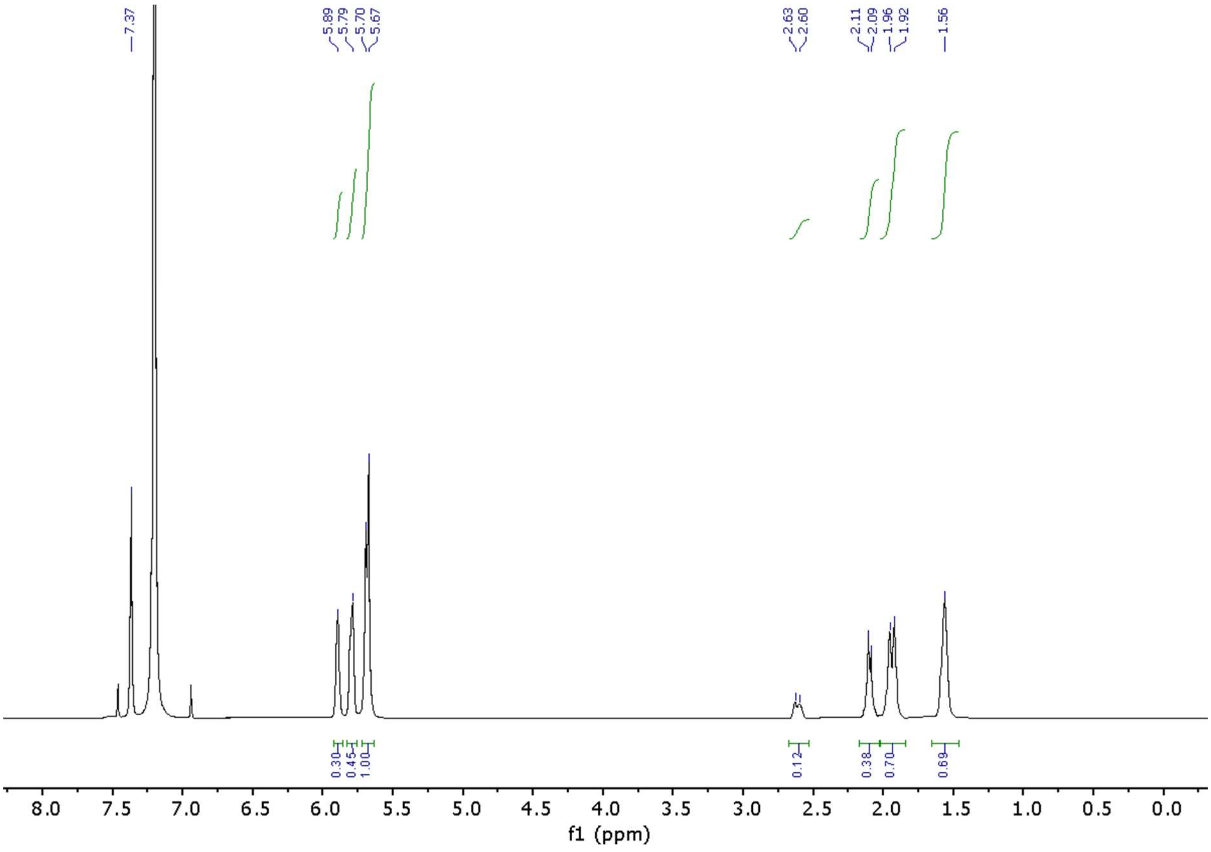

Figure S33. The ^{2}H NMR spectrum of the mixture of **CHDs** at 298 K.

$Ru_2Cl_4(\eta^6-C_6D_6)_2$ (5)

*The compound was synthesized using a slightly modified literature procedure[4] for the non-deuterated analog. The mixture of **CHDs** was used without any additional purifications.*

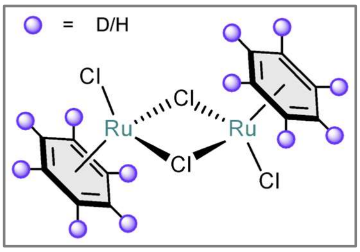

Hydrated ruthenium trichloride $RuCl_3{\cdot}xH_2O$ (500.0 mg) was dissolved in 15.0 mL EtOD to give a black solution. Then the mixture of deuterated cyclohexadienes (**CHDs**) was added (1.2 mL) and the resulting mixture was refluxed for 4 hours. The black precipitate was filtered off, washed with MeOH (5 x 10.0 mL), and dried under a dynamic vacuum to give 368.0 mg of dark brown/black powder (76 % taking into account 61% deuteration of **1-D** (for deuteration see qNMR spectra Fig. S38) and approximating the ruthenium starting material to $RuCl_3{\cdot}3H_2O$).

^{1}H NMR (400 MHz, $(CD_3)_2SO$, 298K): δ = 5.96 (s).

^{13}C{^{1}H} NMR (100 MHz, $(CD_3)_2SO$, 298K): δ = 87.7 – 87.4 (m)

^{2}H NMR (61 MHz, $(CD_3)_2SO$, 298K): δ = 5.97 (s).

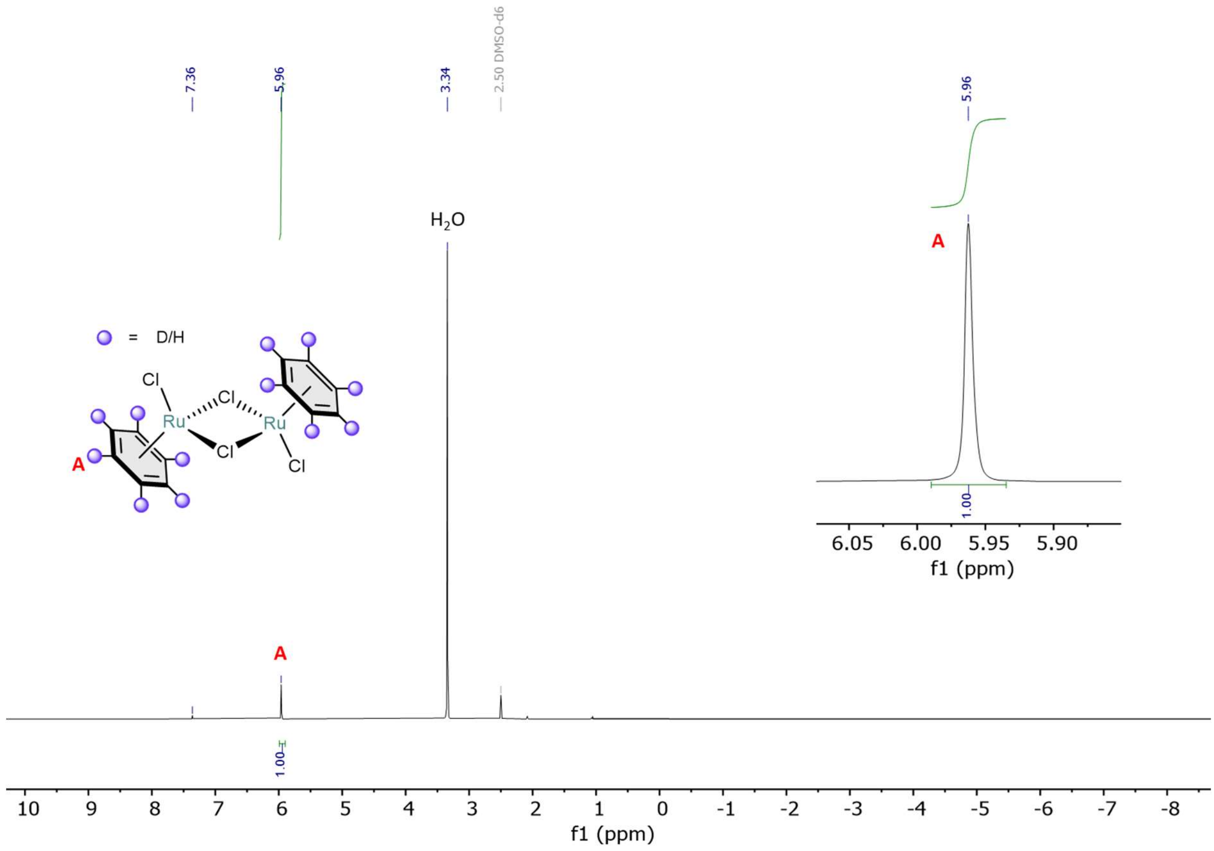

Figure S34. The ^{1}H NMR spectrum of compound **5** in $(CD_3)_2SO$ at 298 K.

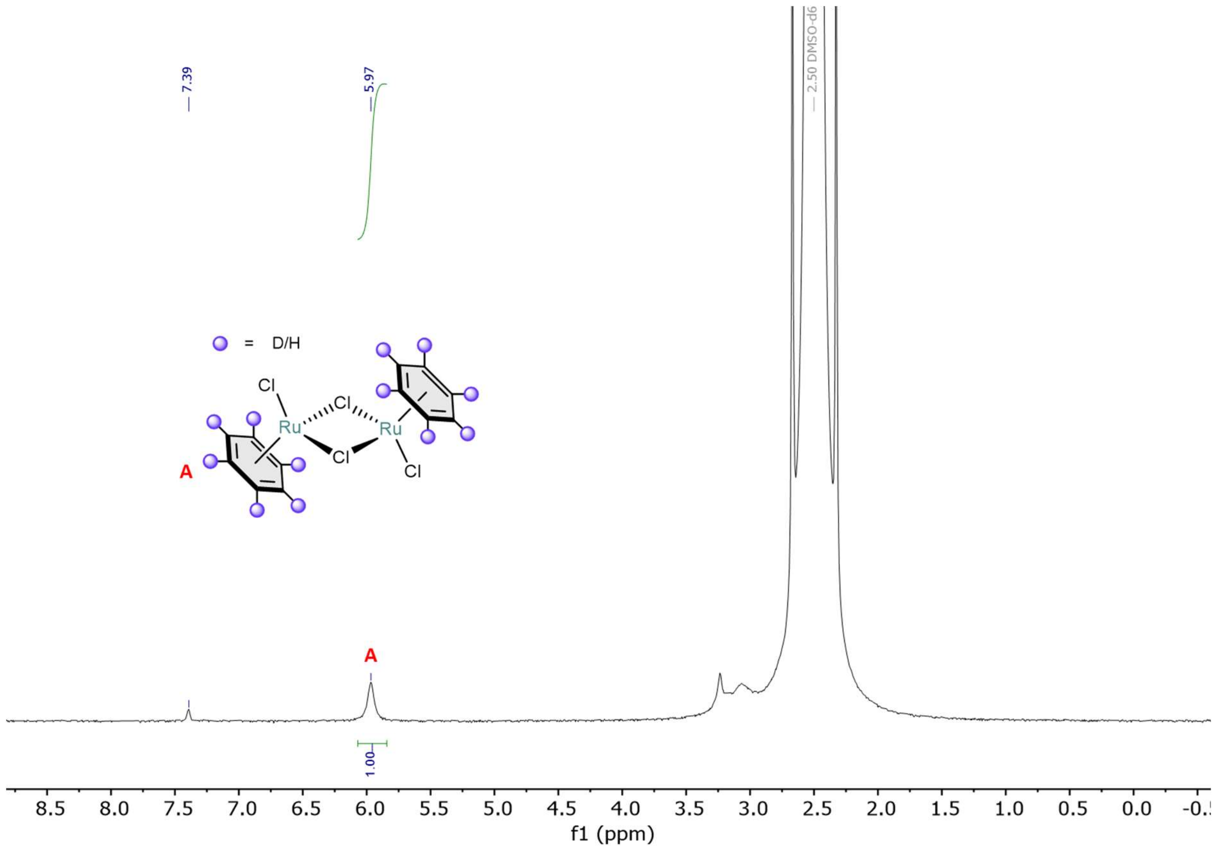

Figure S35. The ^{2}H NMR spectrum of compound **5** in $(CD_3)_2SO$ at 298 K.

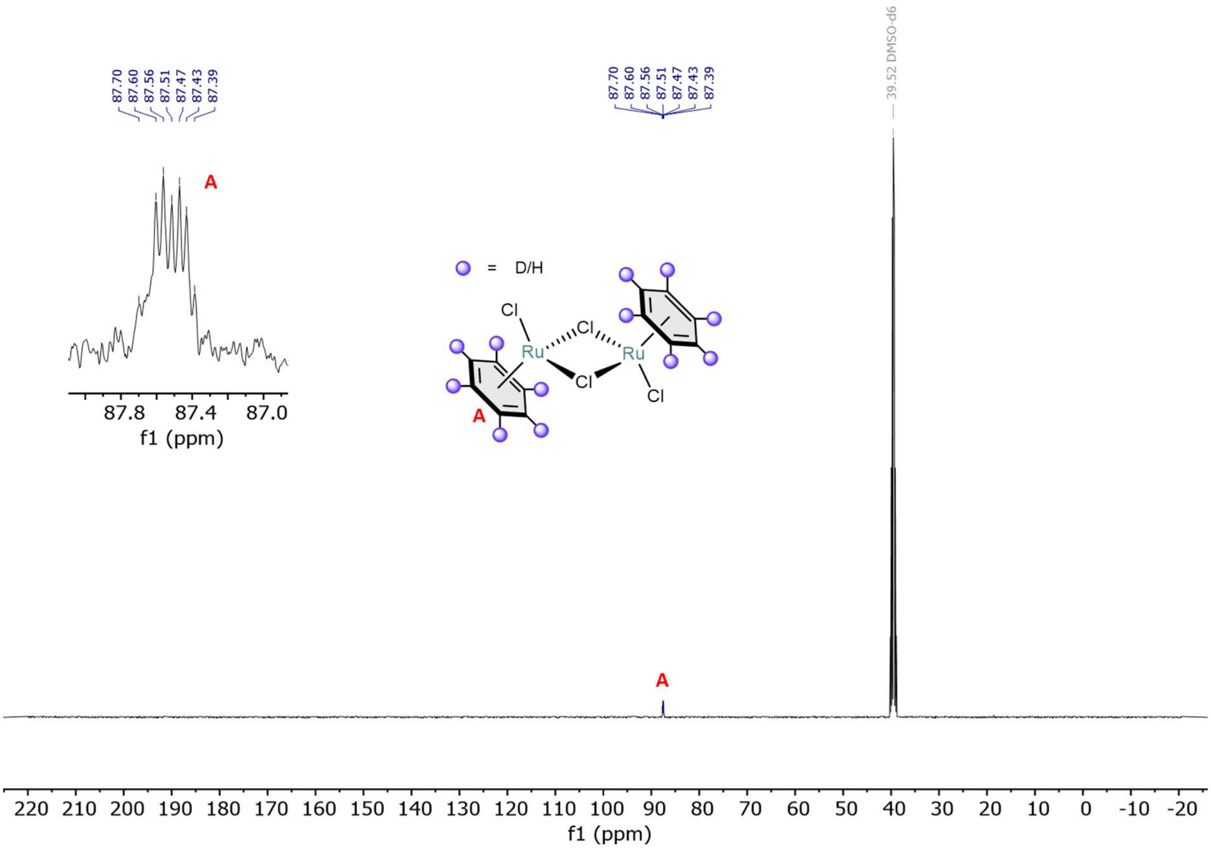

Figure S36. The ^{13}C NMR spectrum of compound **5** in $(CD_3)_2SO$ at 298 K.

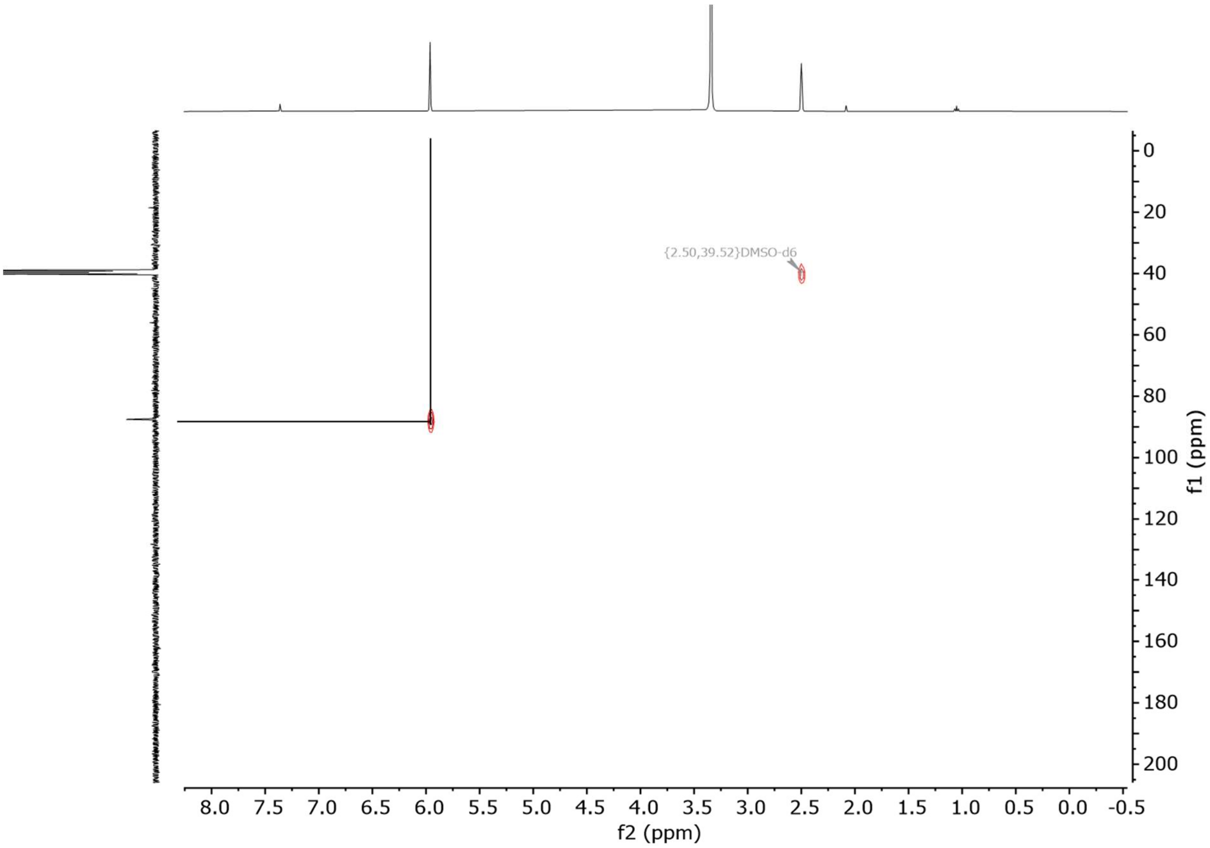

Figure S37. The ^{1}H-^{13}C ASAP-HMQC NMR spectrum of compound **5** in $(CD_3)_2SO$ at 298 K.

[(^{tBu}PN)RuCl(C_6D_6)][PF_6] (1-D)

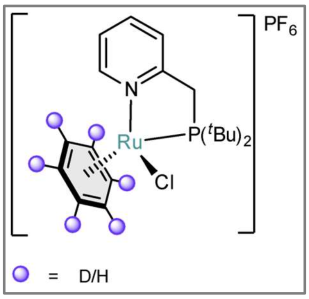

A 20 mL vial was charged with ^{tBu}PN (19.3 mg, 0.08 mmol), KPF_6 (18.7 mg, 0.10 mmol), and **5** (20.6 mg, 0.04 mmol). DCM (7 mL) was added, and the resulting orange suspension was stirred at RT for 18 h. The resulting dark brown mixture was filtered through a pipette filter to remove unreacted KPF_6 and KCl, to give a dark brown filtrate. After removing all volatiles under a dynamic vacuum, THF (1.5 mL) was added, and the suspension was stirred for 15 min. Next, the mixture was filtered to give a bright yellow powder as the product (32.0 mg (64%)).

Relative integration of the ^{1}H NMR spectrum gives 61% deuteration of the benzene ligand. See Fig. S38 for detailed deuterium distribution.

^{1}H NMR (400 MHz, CD_2Cl_2, 298K): δ = 9.23 (d, $^3J_{H,H}$ = 6.4 Hz, 1H), 7.88 – 7.79 (m, 1H), 7.44 (d, $^3J_{H,H}$ = 7.8 Hz, 1H), 7.40 – 7.33 (m, 1H), 6.11 (s, 2.4H)*, 3.89 (dd, $^2J_{H,H}$ = 16.4, $^2J_{H,P}$ = 8.7 Hz, 1H), 3.31 (dd, $^2J_{H,H}$ = 16.4, $^2J_{H,P}$ = 13.2 Hz, 1H), 1.57 (d, $^3J_{H,P}$ = 14.5 Hz, 9H), 1.21 (d, $^3J_{H,P}$ = 13.5 Hz, 9H).

** The integration shows fewer protons because of the partial deuteration of these positions.*

^{13}C{^{1}H} NMR (100 MHz, CD_2Cl_2, 298K): δ = 163.0 (d, $^4J_{C,P}$ = 2.4 Hz), 157.4 (s), 140.5 (d, $^4J_{C,P}$ = 3.1 Hz), 125.1, 125.0, 90.1 – 89.3 (m, a complicated multiplicity caused by deuteration), 39.7 (s), 39.5 (d, $^1J_{C,P}$ = 1.8 Hz), 33.5 (d, $^1J_{C,P}$ = 23.2 Hz), 31.6 (d, $^2J_{C,P}$ = 2.4 Hz), 30.0 (d, $^2J_{C,P}$ = 2.5 Hz).

^{31}P{^{1}H} NMR (162 MHz, CD_2Cl_2, 298K): δ = 90.8 (s, 1P), –144.5 (hept, $^1J_{P,F}$ = 712.3 Hz, 1P)

^{19}F NMR (376 MHz, CD_2Cl_2, 298K): δ = –72.9 (d, $^1J_{F,P}$ = 710.9 Hz).

^{2}H NMR (61 MHz, CD_2Cl_2, 298K): δ = 6.13 (s).

ATR-IR (film, N_2 flow): ν = 2958 (m), 2925 (s), 2855 (m), 1607 (w), 1474 (m), 1374 (w), 1264 (m), 1177 (m), 1024 (w), 835 (s), 734 (m), 556 (s), 493 (w), 459 (w) cm^{-1}.

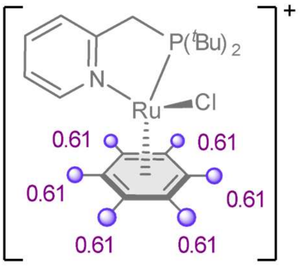

Figure S38. Detailed deuterium distribution in the complex **1-D**. The purple balls represent the positions where a significant amount of deuterium was detected in ^{1}H qNMR.

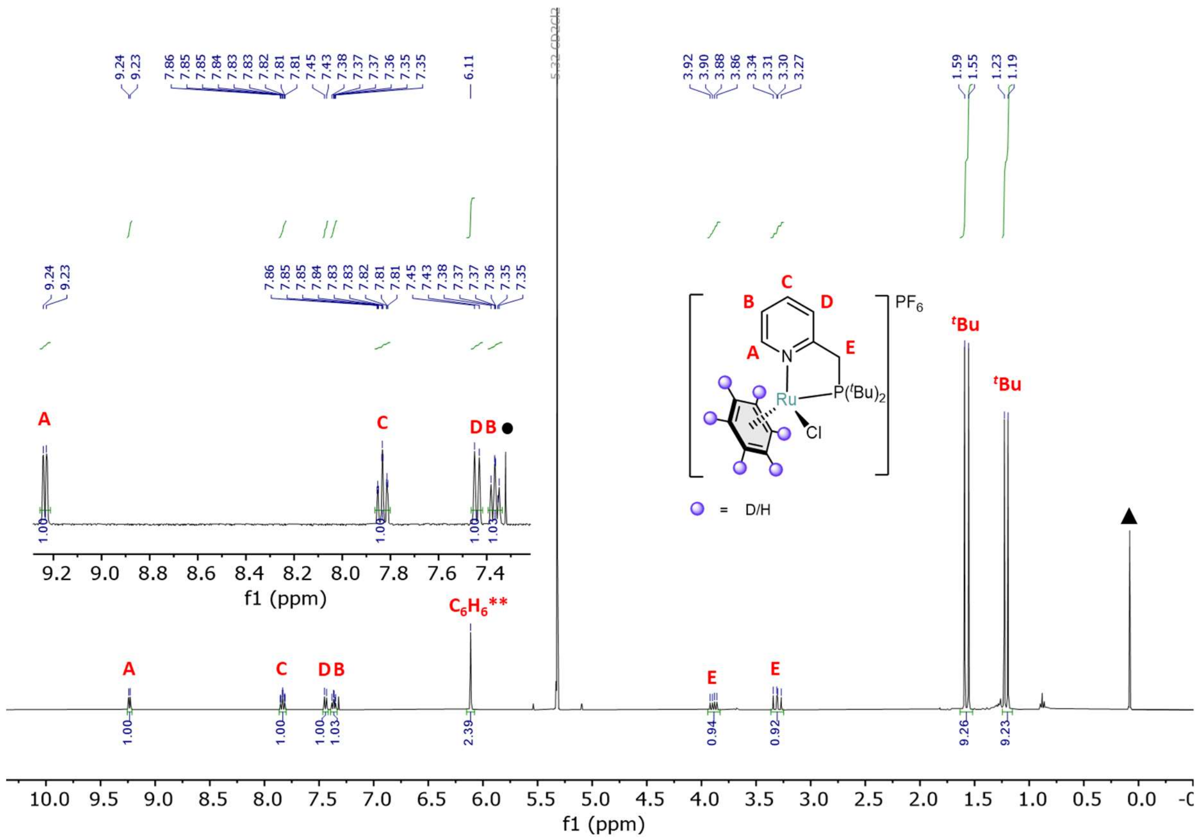

Figure S39. The ^{1}H NMR spectrum of complex **1-D** in CD_2Cl_2 at 298 K. ***The integration of the coordinated arene gives less than 6 protons because of the partial deuteration of this ligand (see above). The black circle and triangle represent the remaining free benzene and grease, respectively.*

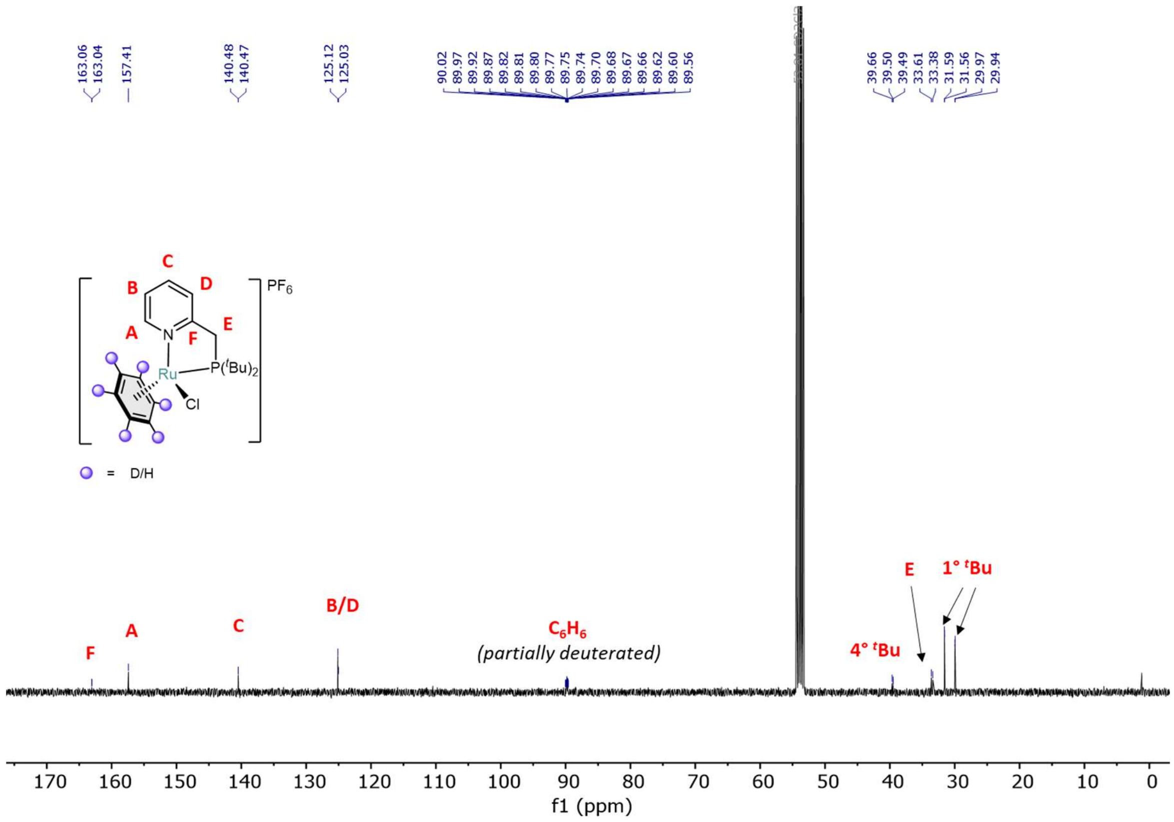

Figure S40. The $^{13}C\{^{1}H\}$ NMR spectrum of complex **1-D** in CD_2Cl_2 at 298 K.

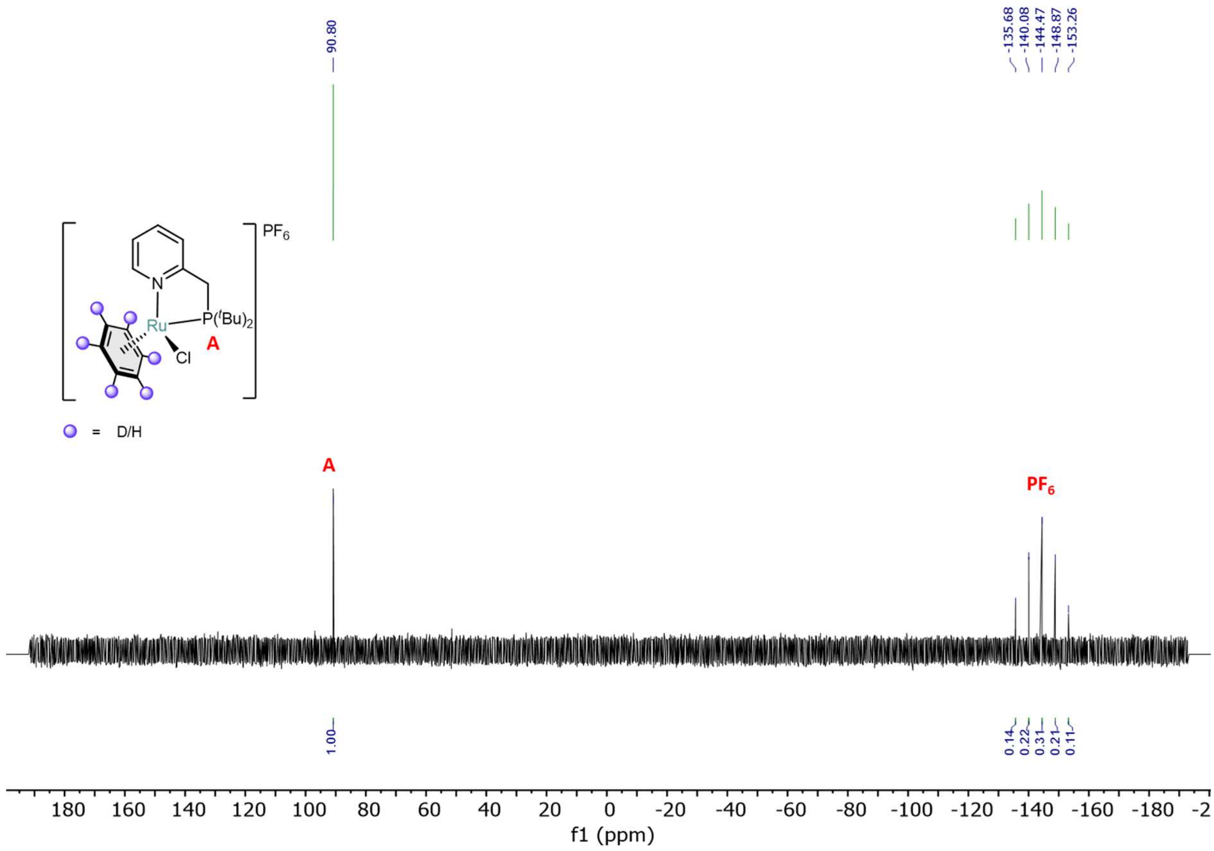

Figure S41. The $^{31}P\{^{1}H\}$ NMR spectrum of complex **1-D** in CD_2Cl_2 at 298 K.

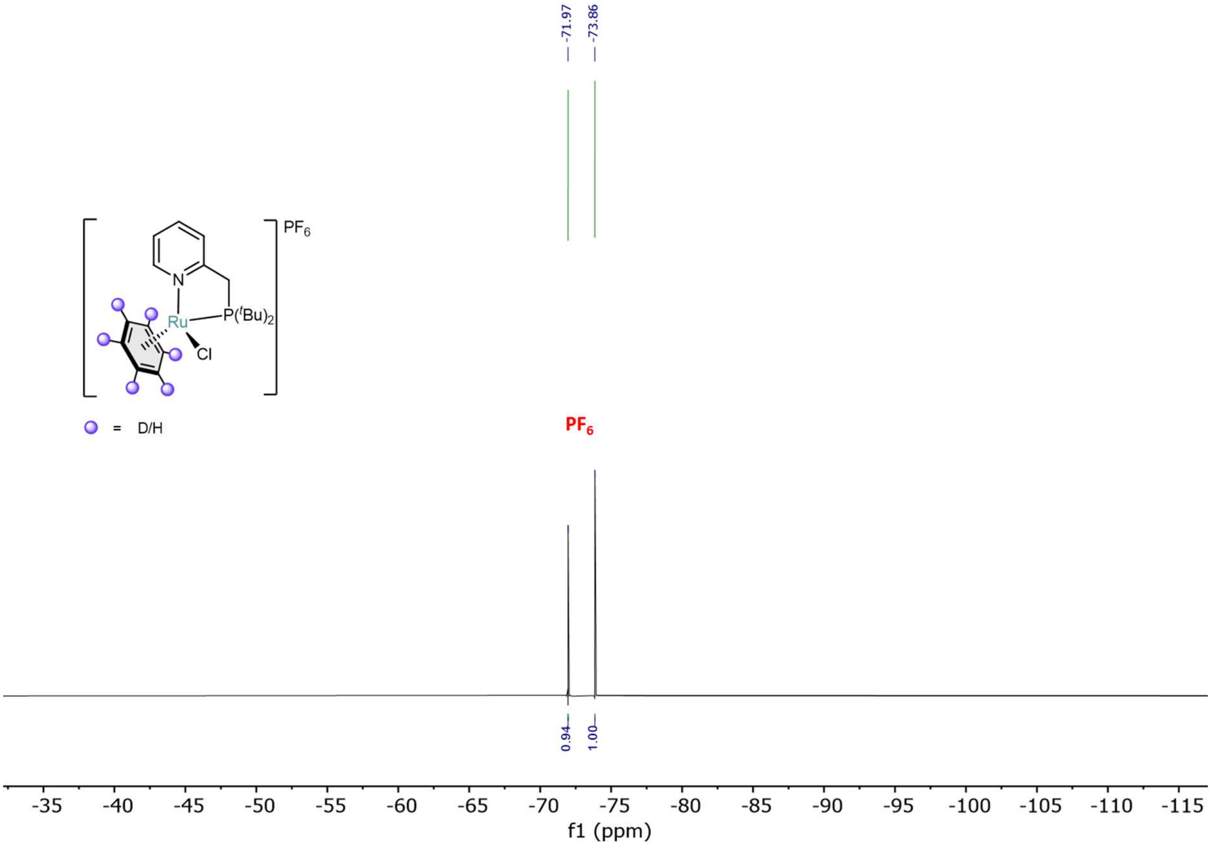

Figure S42. The ^{19}F NMR spectrum of complex **1-D** in CD_2Cl_2 at 298 K.

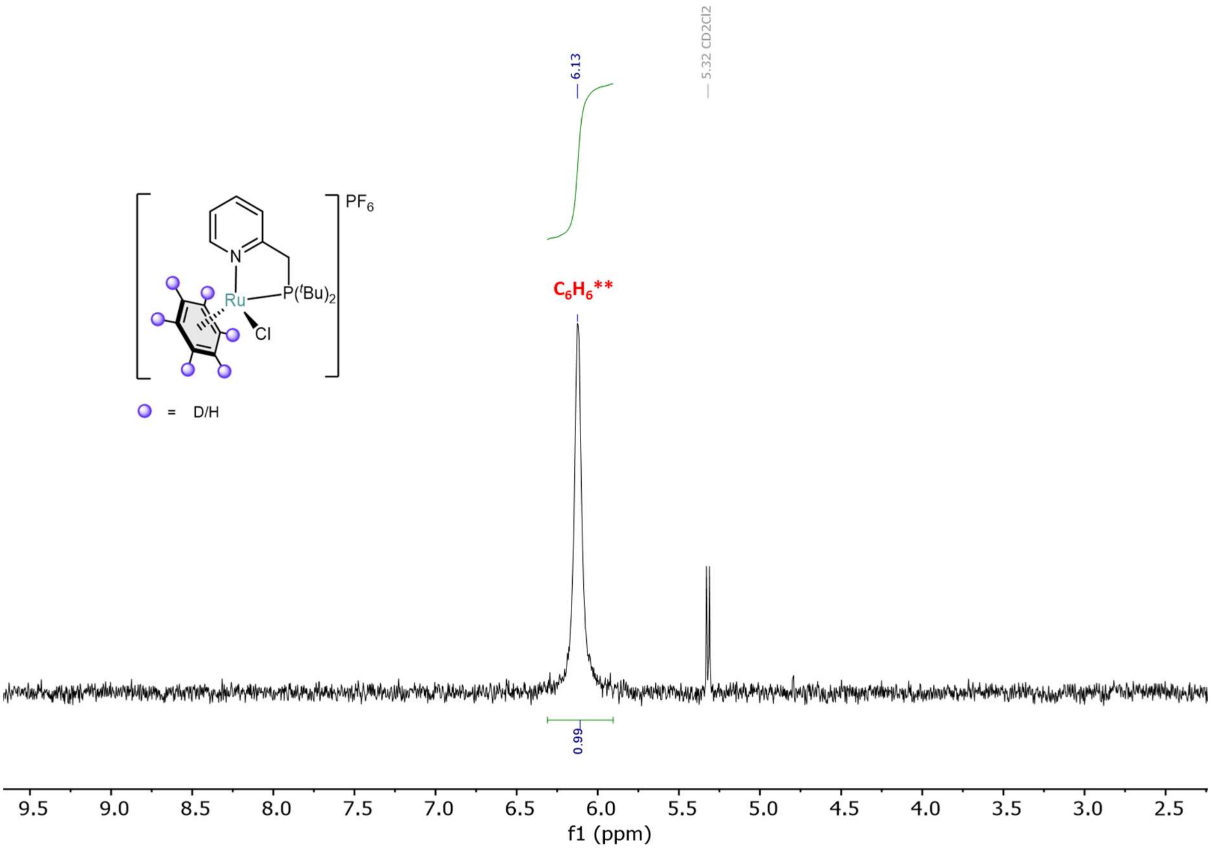

Figure S43. The ^{2}H NMR spectrum of complex **1-D** in CD_2Cl_2 at 298 K. **The coordinated arene appears very pronounced because of the partial deuteration of this ligand

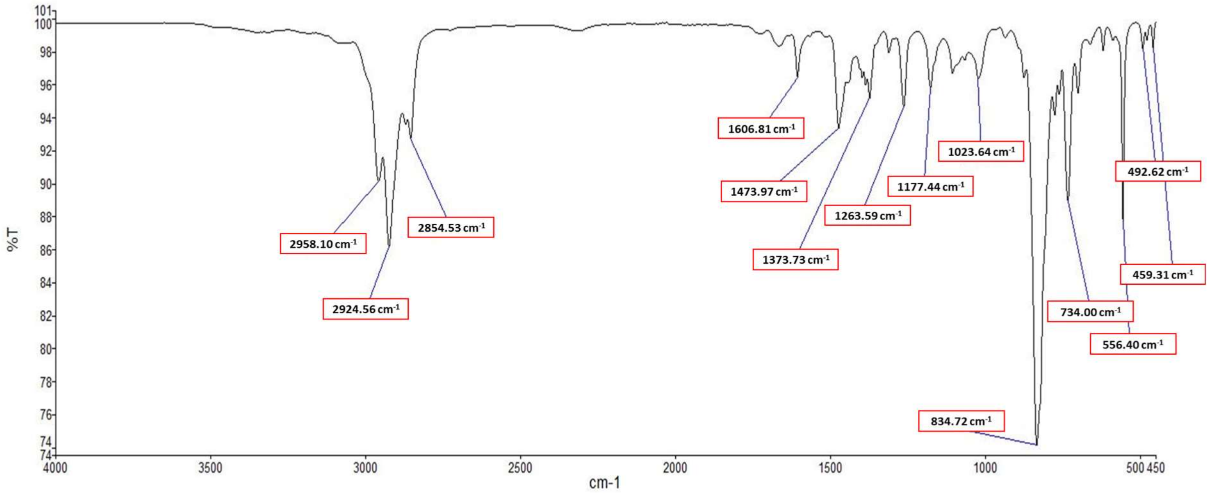

Figure S44. The ATR-IR spectrum of complex **1-D** measured as a film under N_2 flow at 298 K.

(^{tBu}PN*)RuD(PhN(TMS)$_2$-d_5) (4-D)

A yellow suspension of complex **1-D** (47.1 mg, 0.08 mmol) in THF (4.0 mL) was added dropwise to a colorless solution of KN(TMS)$_2$ (31.3 mg, 0.16 mmol) in THF (3.0 mL). The starting complex instantly dissolved upon the addition resulting in a colour change to dark brown. After stirring for 0.5 h at RT, the mixture was dried under a dynamic vacuum to give a dark brown solid. The residue was extracted with pentane (5.0 mL) and the extracts were dried under a dynamic vacuum to give a dark brown sticky solid (42.2 mg, 91%).

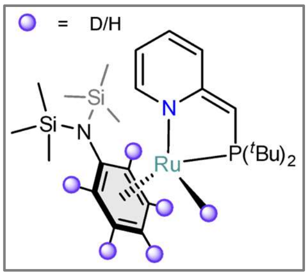

See Fig. S45 for detailed deuterium distribution.

^{1}H NMR (400 MHz, C_6D_6, 298 K): δ = 7.33 (d, J = 6.2 Hz, 1H), 6.53 (dddd, $^3J_{H,H}$ = 9.0, $^3J_{H,H}$ = 6.4, $^5J_{H,P}$ = 1.9, $^4J_{H,H}$ = 1.8 Hz, 1H), 6.41 (d, J = 8.5 Hz, 1H), 5.38 (ddd, $^3J_{H,H}$ = 6.6, $^3J_{H,H}$ = 6.3, $^4J_{H,H}$ = 1.6 Hz, 1H), 5.14 – 5.08 (m, 0.51H)*, 4.95 – 4.92 (m, 0.42H)*, 4.80 – 4.74 (m, 0.42H)*, 4.68 – 4.65 (m, 0.41H)*, 4.16 – 4.12 (m, 0.41H)*, 3.49 (d, $^2J_{H,P}$ = 2.6 Hz, 1H), 1.31 (d, $^3J_{H,P}$ = 12.3 Hz, 9H), 1.27 (d, $^3J_{H,P}$ = 13.2 Hz, 9H), 0.25 (s, 18H), –(7.69 – 7.86) (m, 0.45H)*.

** The integration shows fewer protons because of the partial deuteration of these positions.*

^{13}C{^{1}H} NMR (100 MHz, C_6D_6, 298 K) δ = 170.9 (d, $^2J_{C,P}$ = 15.9 Hz), 154.4 (s), 130.8 (d, $^4J_{C,P}$ = 1.0 Hz), 130.7 (d, $^2J_{C,P}$ = 0.7 Hz), 115.2 (d, $^3J_{C,P}$ = 17.0 Hz), 101.4 (s), 92.5 – 92.1 (m), 90.7 – 90.2 (m), 83.5 – 82.7 (m), 77.6 – 77.2 (m), 74.2 – 73.9 (m), 62.3 (d, $^1J_{C,P}$ = 60.5 Hz), 38.3 (d, $^1J_{C,P}$ = 14.5 Hz), 36.2 (d, $^1J_{C,P}$ = 34.7 Hz), 31.1 (d, $^2J_{C,P}$ = 3.4 Hz), 30.2 (d, $^2J_{C,P}$ = 5.1 Hz), 3.3 (s).

^{31}P{^{1}H} NMR (162 MHz, C_6D_6, 298 K): δ = 98.8 (s, 1P).

ATR-IR (film, N_2 flow): ν = 2952 (m), 2897 (m), 1665 (w), 1603 (m), 1487 (s), 1447 (s), 1385 (w), 1367 (w), 1285 (m), 1253 (m), 1179 (w), 1000 (m), 890 (s), 844 (s), 810 (s), 756 (w), 677 (m), 618 (w), 485 (w), 462 (w) cm^{-1}.

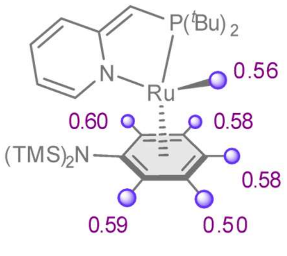

Figure S45. Detailed deuterium distribution in the complex **4-D**. The purple balls represent the positions where a significant amount of deuterium was detected in ^{1}H qNMR.

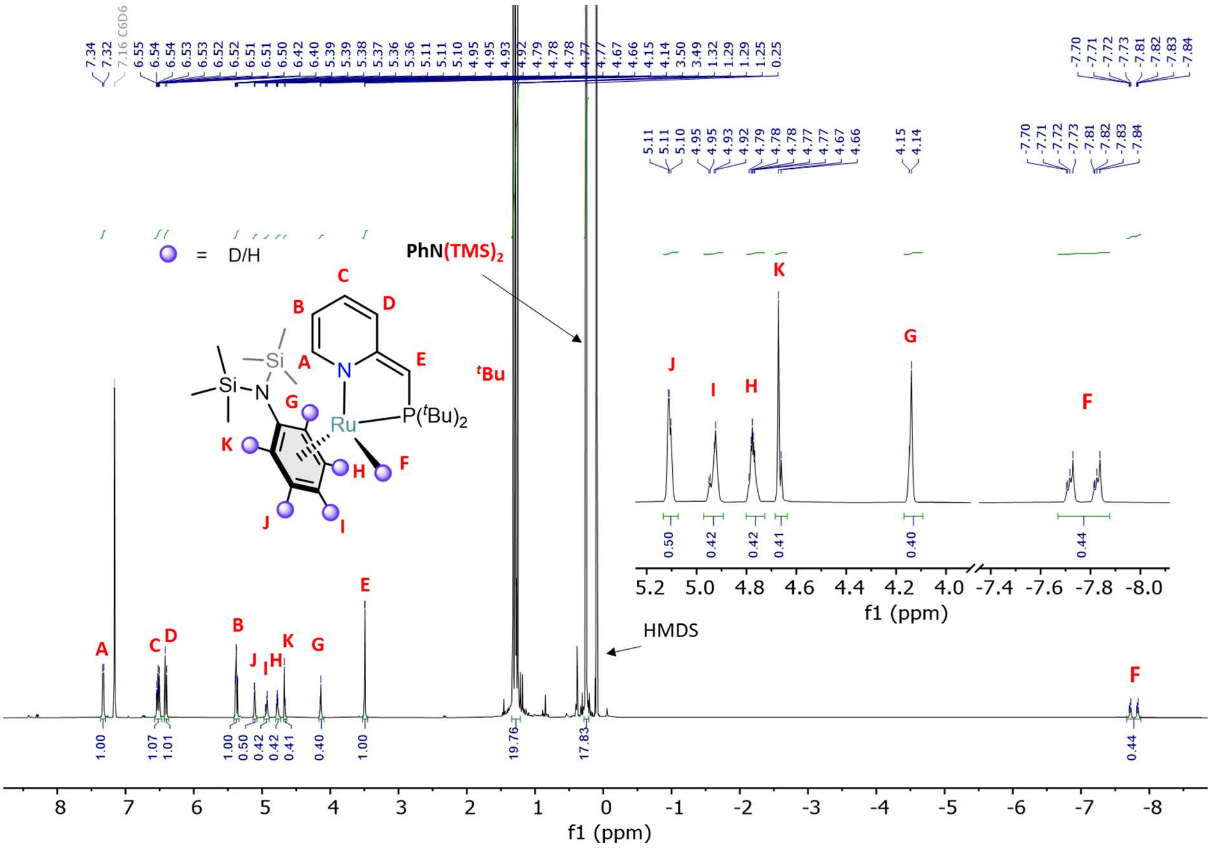

Figure S46. The ^{1}H NMR spectrum of complex **4-D** in C_6D_6 at 298 K. Additionally zoomed regions of aromatic aniline protons and hydride are shown.

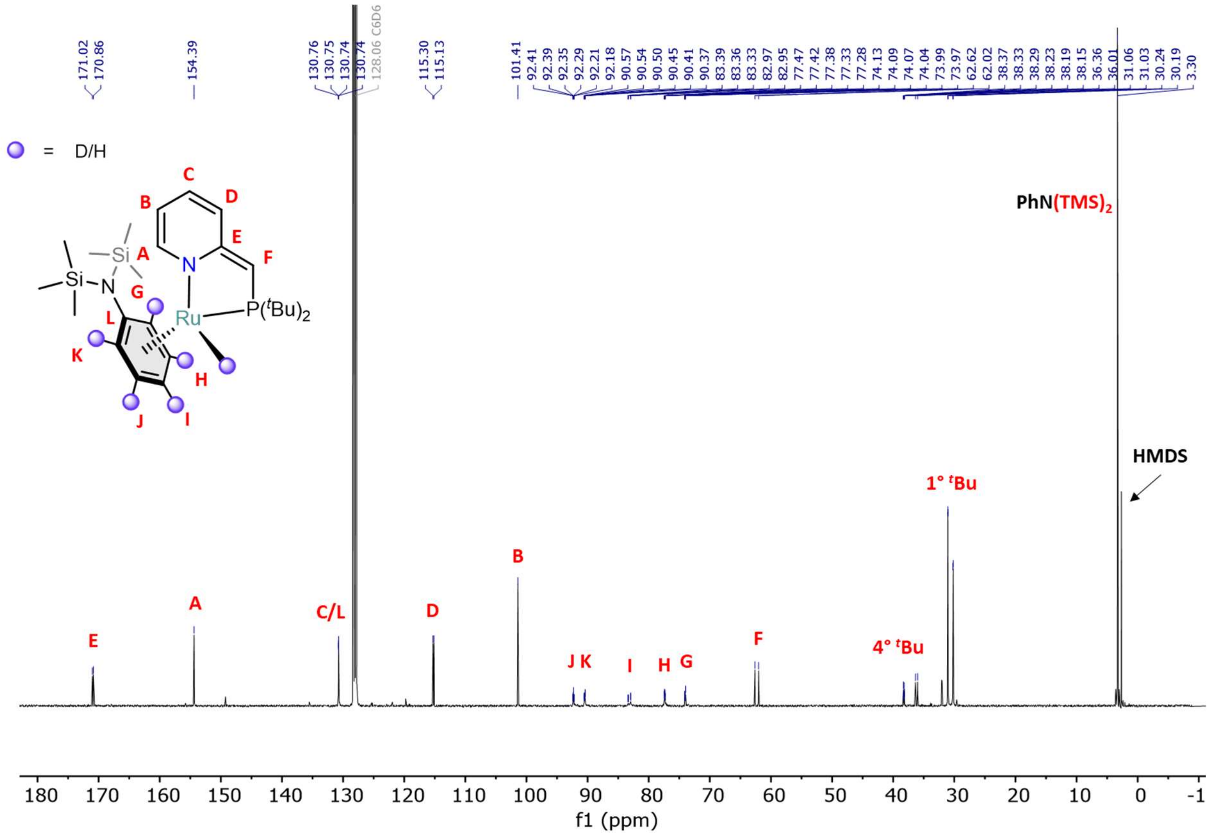

Figure S47. The $^{13}C\{^{1}H\}$ NMR spectrum of complex **4-D** in C_6D_6 at 298 K.

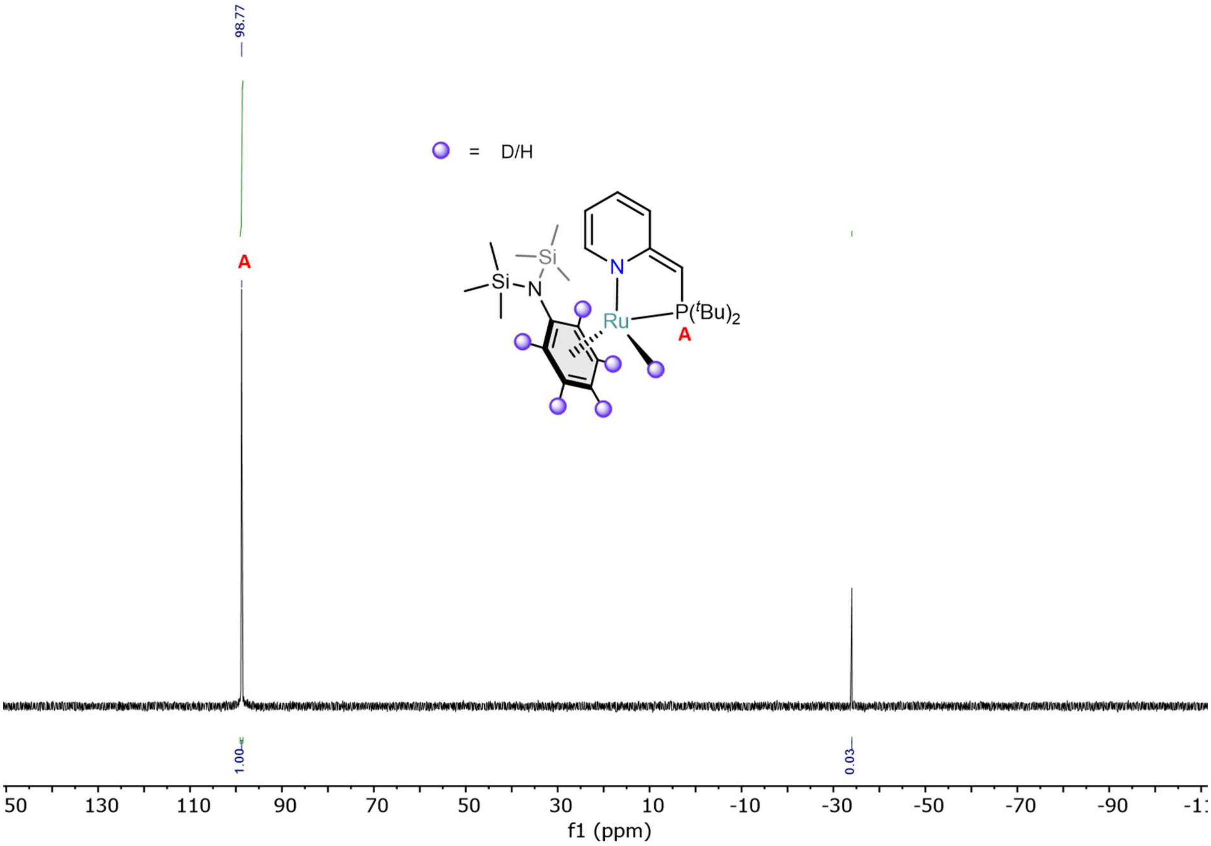

Figure S48. The $^{31}P\{^{1}H\}$ NMR spectrum of complex **4-D** in C_6D_6 at 298 K.

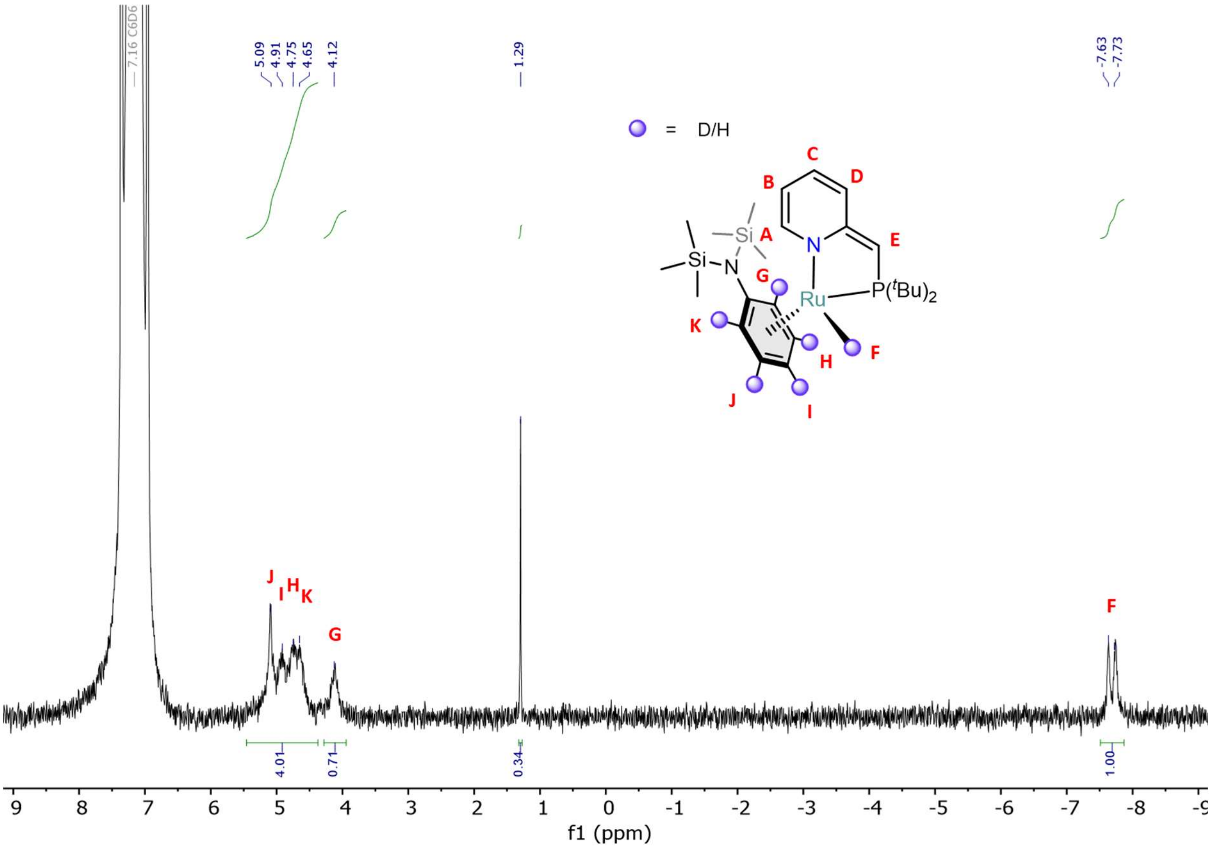

Figure S49. The ^2H NMR spectrum of complex **4-D** in C_6D_6 at 298 K.

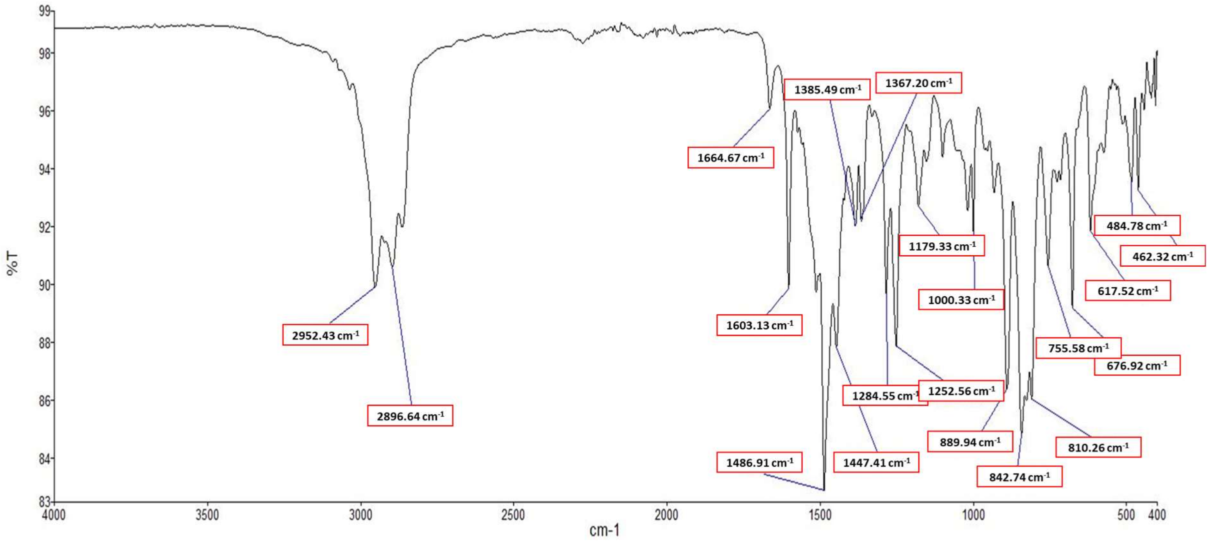

Figure S50. The ATR-IR spectrum of complex **4-D** measured as a film under N_2 flow at 298 K.

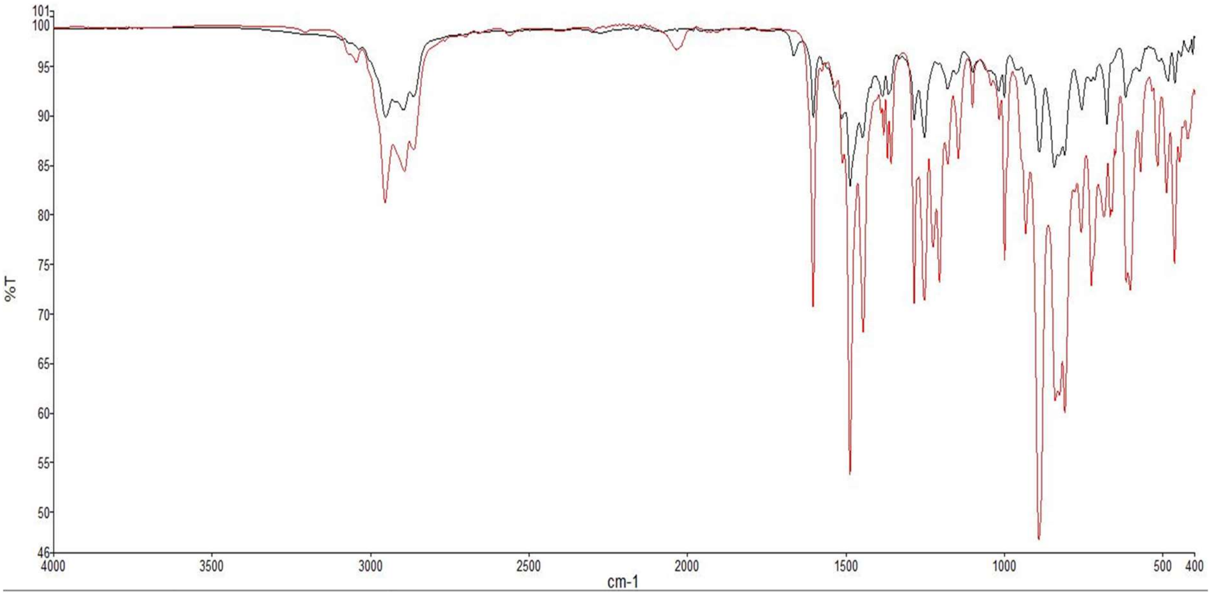

Figure S51. An overlap of the ATR-IR spectra of complexes **4** (the red line) and **4-D** (the black line) measured as films under N_2 flow at 298 K.

S2 Mechanistic Studies

S2.1 Room-Temperature Experiments

Nature of Complex 2-K

To get more insights into the role of the potassium cation, we performed a reaction between **1** and 1 equiv of $KN(TMS)_2$ in the presence of 1.1 equiv of [2.2.2]-cryptand (Fig. S52 (*top*)). In contrast to the analogous reaction in the absence of the cryptand (Fig. S52 (*bottom*)), NMR analysis showed no resonances associated with complex **3,** and exclusive formation of deprotonated complex **2** was observed. Interestingly, **2** displays the same set of ^{1}H and ^{31}P NMR signals as **2-K** but at considerably shifted positions. The addition of the excess of KPF_6 to a solution of **2** in THF results in the fast formation of the -*onium* complex **2-K** (see Fig. S73 for stacked spectra of **2** and **2** + KPF_6).

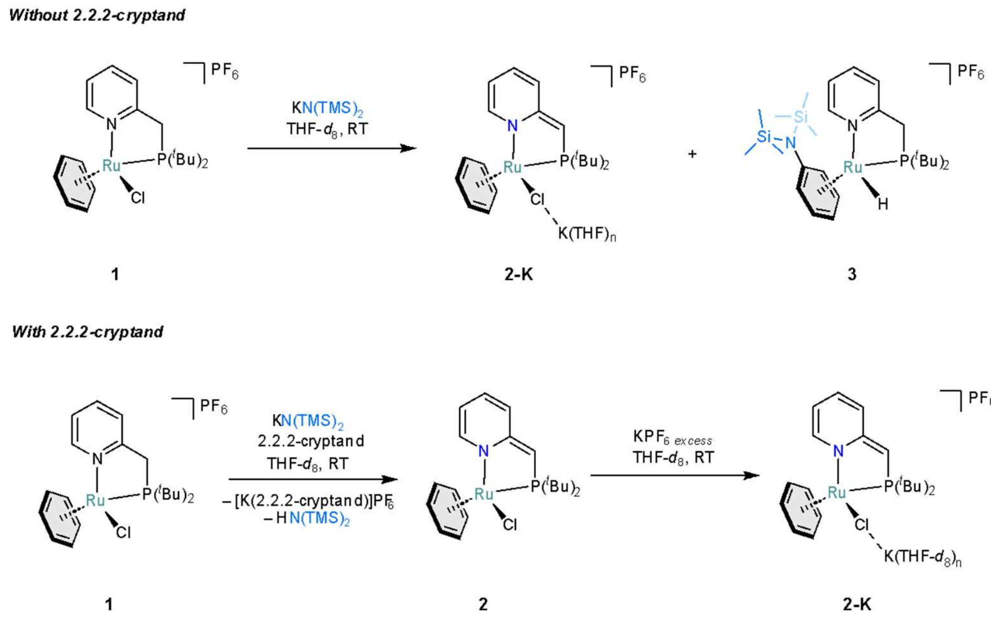

Figure S52. Reactions between **1** and 1 equiv of $KN(TMS)_2$ without (*top*) in the presence of [2.2.2]-cryptand (*bottom*).

On the origin of the formation of 3

The formation of complexes **2-K** and **3** can be envisioned via two possible pathways:

1) Route A: two competing independent reactions (Route A, Fig. S53) – deprotonation and nucleophilic addition. Direct hydride migration leading to complex **3** with KCl extrusion (see Supplementary Section S3.1 for computational studies of this process).

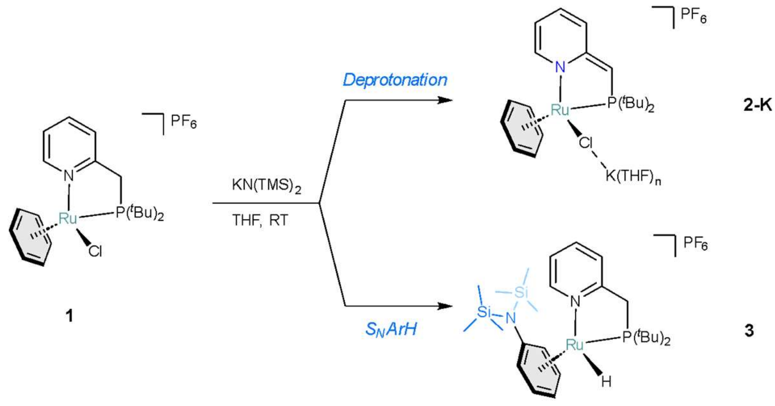

Figure S53. Origin of the formation of complex 3. Route A: complex **3** forms via direct nucleophilic substitution of complex **1**.

2) A sequence of two dependent reactions (Route B, Fig. S54) – complex **1** partially reacts with the base to yield hydride complex **4** which subsequently gets protonated by the rest of complex **1**. The presence of potassium cation can theoretically also decrease the pKa of **1** and propel the deprotonation.

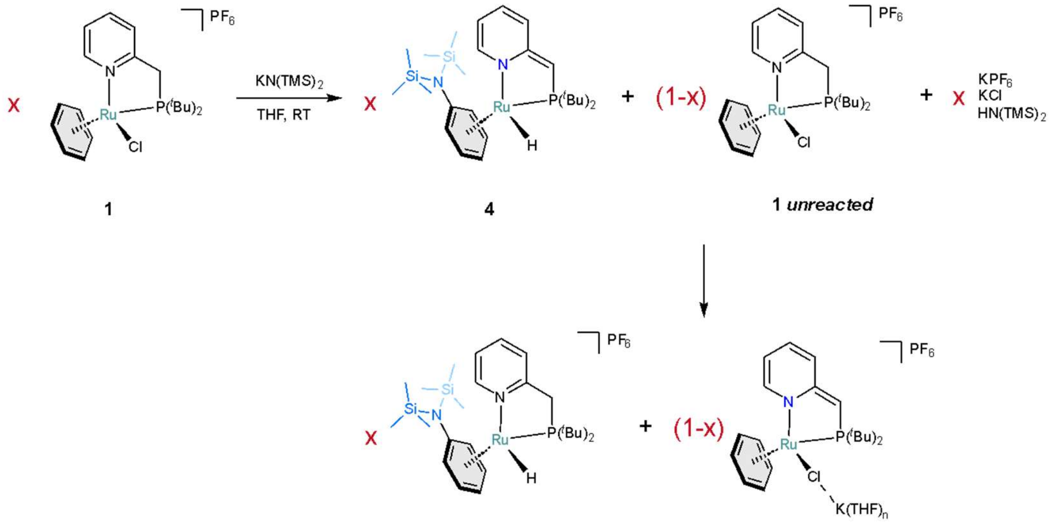

Figure S54. Origin of the formation of complex 3. Route B: complex **3** forms via protonation of complex **4** by residuals of starting complex **1**.

Given the considerably high energy barrier for hydride migration for route A (see Supplementary Section S3.1 for computational studies) we probed if the complexes **1** and **4** can react with each other in the presence of potassium ions (Route B, Fig. S54).

To verify this hypothesis of the reaction between **1** and **4** (Route B, Fig. S54) we performed a reaction between 1 equiv of **1** and 1 equiv of **4** in THF at RT in the presence of KPF_6 (Supplementary Figs. 56-59). Indeed, this reaction was found to be relatively fast and led to the clean formation of a mixture of compounds **2-K** and **3** identically to the reaction between **1** and 1 equiv of $KN(TMS)_2$ (see Methods and Supplementary Section S1 for details). This experiment shows that the formation of **3** can be related to simply a higher basicity of **4** compared to **2-K**.

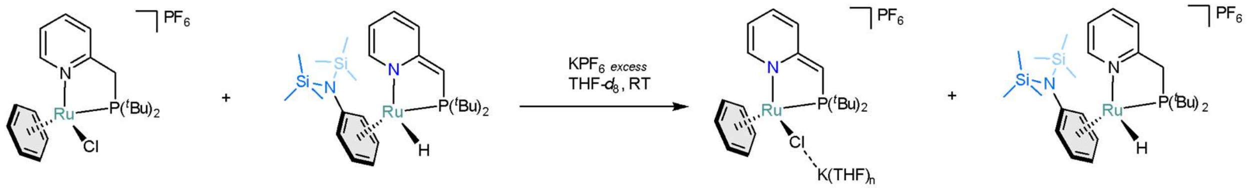

Figure S55. Reactions between **1** and **4** in the presence of KPF_6.

Procedure: A colorless solution of **4** (5.7 mg, 0.01 mmol) in THF (0.5 mL) was added dropwise to a yellow suspension of complex **1** (6.0 mg, 0.01 mmol) and KPF_6 (1.8 mg, 0.01 mmol) in THF (2.5 mL). The resulting dark brown suspension was stirred for 30 min at RT to give a clear orange solution. A sample of the mixture was transferred into a J. Young tube and the mixture was analyzed by NMR spectroscopy.

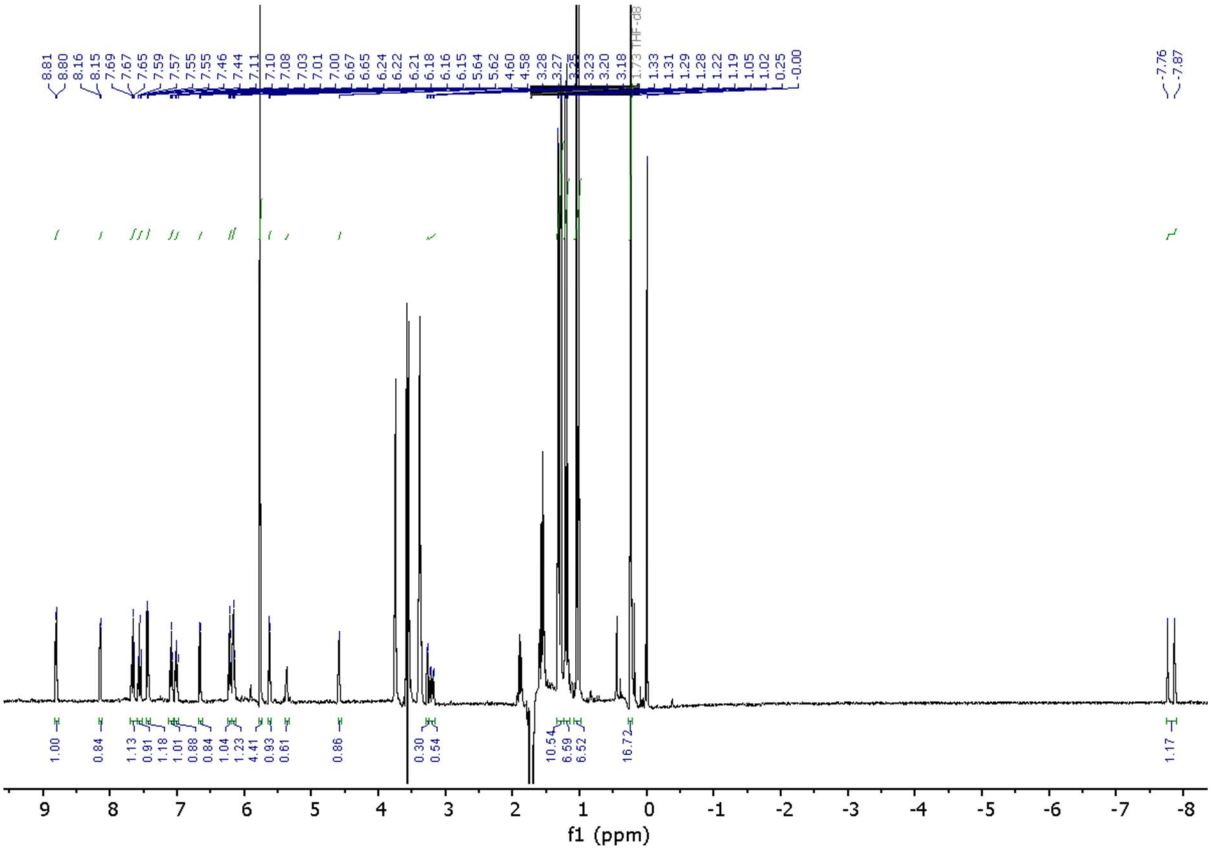

Figure S56. The ^{1}H NMR spectrum of the reaction between **1**, **4**, and KPF_6 in THF (PRESAT option) at 298 K.

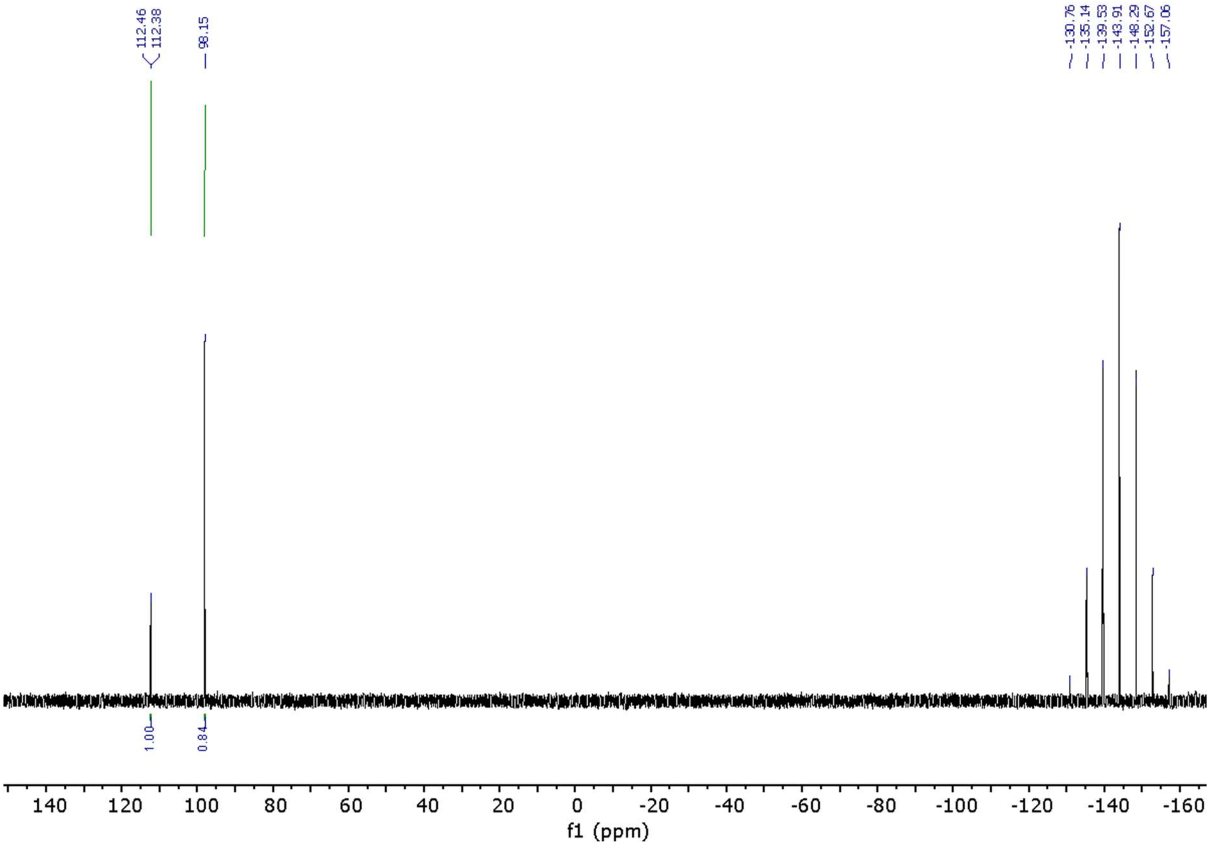

Figure S57. The ^{31}P NMR spectrum of the reaction between **1**, **4**, and KPF_6 in THF at 298 K.

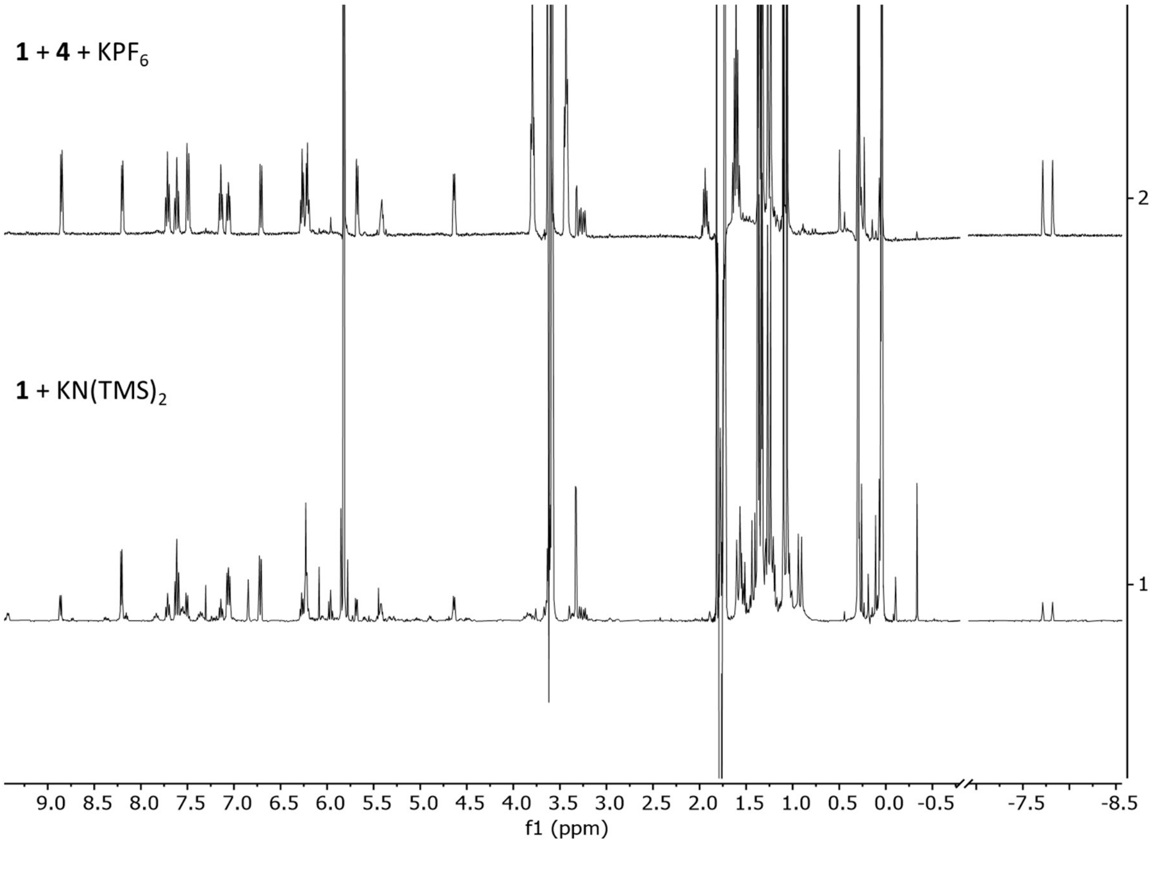

Figure S58. The stacked ^{1}H NMR spectra of the reaction between **1**, **4**, and KPF_6 (*top*, in THF (PRESAT)) and the reaction between **1** and 1 equiv of $KN(TMS)_2$ (*bottom*, in THF-d_8).

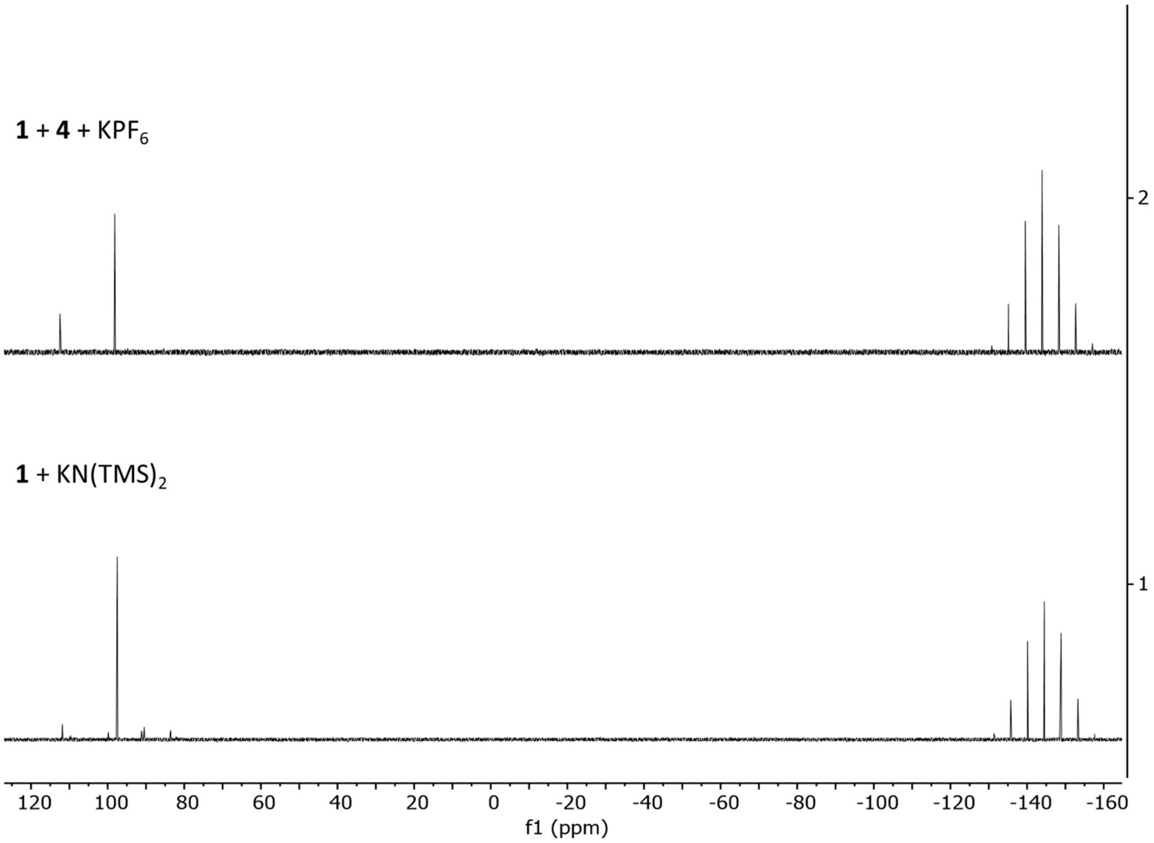

Figure S59. The stacked ^{31}P NMR spectra of the reaction between **1**, **4**, and KPF_6 (*top*, in THF) and the reaction between **1** and 1 equiv of $KN(TMS)_2$ (*bottom*, in THF-d_8).

(^{tBu}PN*)RuCl(C_6H_6) (2)

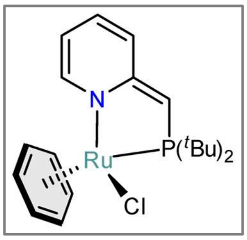

A colorless solution of KN(TMS)$_2$ (4.0 mg, 0.02 mmol) and Kryptofix® 2.2.2 (8.3 mg, 0.02 mmol) in THF-d_8 (0.5 mL) was added dropwise to a yellow suspension of complex **1** (11.9 mg, 0.02 mmol) in THF-d_8 (0.5 mL). The starting complex instantly dissolved upon the addition resulting in a colour change to dark brown. After stirring the reaction mixture for 0.5 h at RT an aliquot of the mixture was transferred into a J. Young tube, 111.0 µL of 9 mM solution of hexamethylbenzene (in THF-d_8) was added as an internal standard, and the mixture was analyzed by NMR spectroscopy.

^{1}H NMR (400 MHz, THF-d_8, 296K): δ = 7.95 (dd, J = 6.5, 1.0 Hz, 1H), 6.26 (dddd, J = 8.9, 6.6, 2.1, 1.9 Hz, 1H), 5.96 – 5.90 (m, 1H), 5.72 (s, 6H), 5.20 (ddd, J = 6.8, 6.4, 1.5 Hz, 1H), 2.99 (d, $^2J_{H,P}$ = 0.7 Hz, 1H), 1.44 (d, $^3J_{H,P}$ = 13.6 Hz, 9H), 1.32 (d, broad, $^3J_{H,P}$ = 11.9 Hz, 9H).

^{31}P{^{1}H} NMR (162 MHz, THF-d_8, 298K): 90.2, –144.6 (hept, $^1J_{P,F}$ = 710.2 Hz).

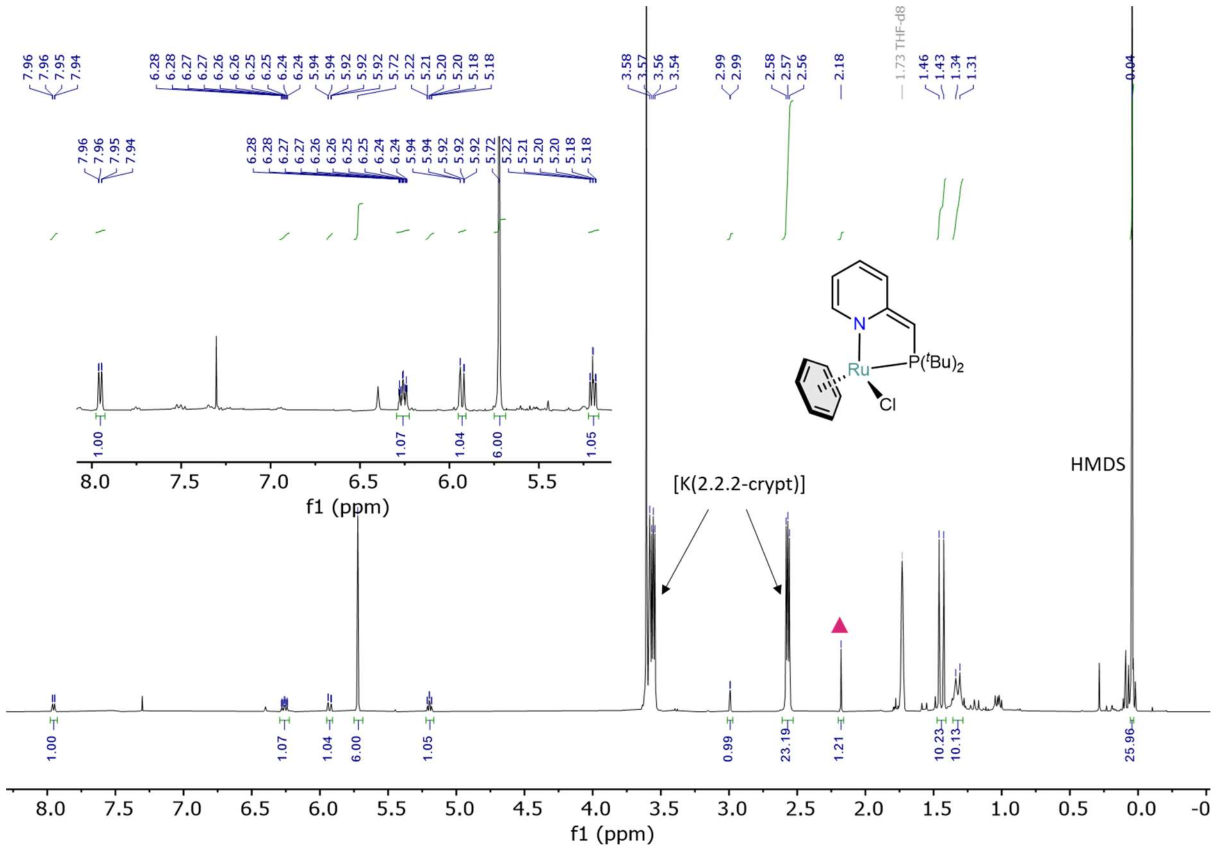

Figure S60. The ^{1}H NMR spectrum of complex **2** in THF-d_8 at 296 K. The internal standard (hexamethylbenzene) is highlighted with the triangle.

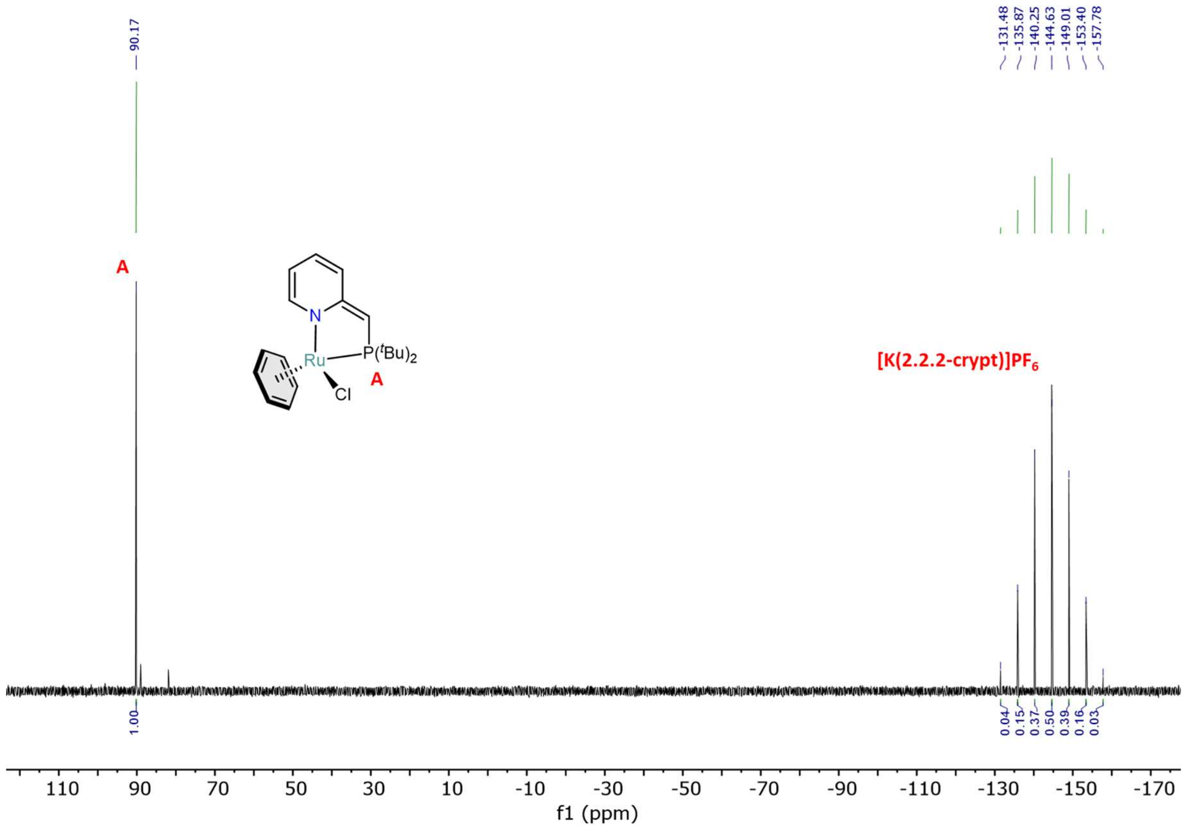

Figure S61. The ^{31}P NMR spectrum of complex **2** in THF-d_8 at 296 K.

Direct synthesis of 2-K from 2

The NMR tube with freshly prepared complex **2** (previous experiment) was placed back in a glove box, and KPF_6 (7.6 mg, 0.04 mmol) was added. The tube was shaken for 10 min resulting in a change from an opaque dark brown solution to a clear light red solution. NMR analysis of the mixture showed full conversion into species **2-K**.

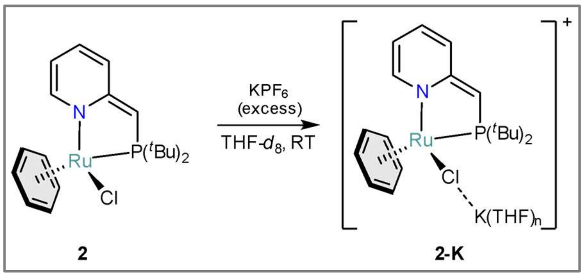

^{1}H NMR (400 MHz, THF-d_8, 296K): δ = 8.21 (d, J = 5.2 Hz, 1H), 7.62 (ddd, J = 8.2, 7.7, 1.4 Hz, 1H), 7.06 (dddd, J = 8.0, 5.4, 1.8, 1.2 Hz, 1H), 6.75 – 6.71 (m, 1H), 5.82 (s, 6H), 3.34 (d, $^2J_{H,P}$ = 3.0 Hz, 1H), 1.36 (d, $^3J_{H,P}$ = 14.9 Hz, 9H), 1.08 (d, $^3J_{H,P}$ = 15.4 Hz, 9H).

^{31}P{^{1}H} NMR (162 MHz, THF-d_8, 298K): δ = 97.5, –144.6 (hept, $^1J_{P,F}$ = 709.8 Hz).

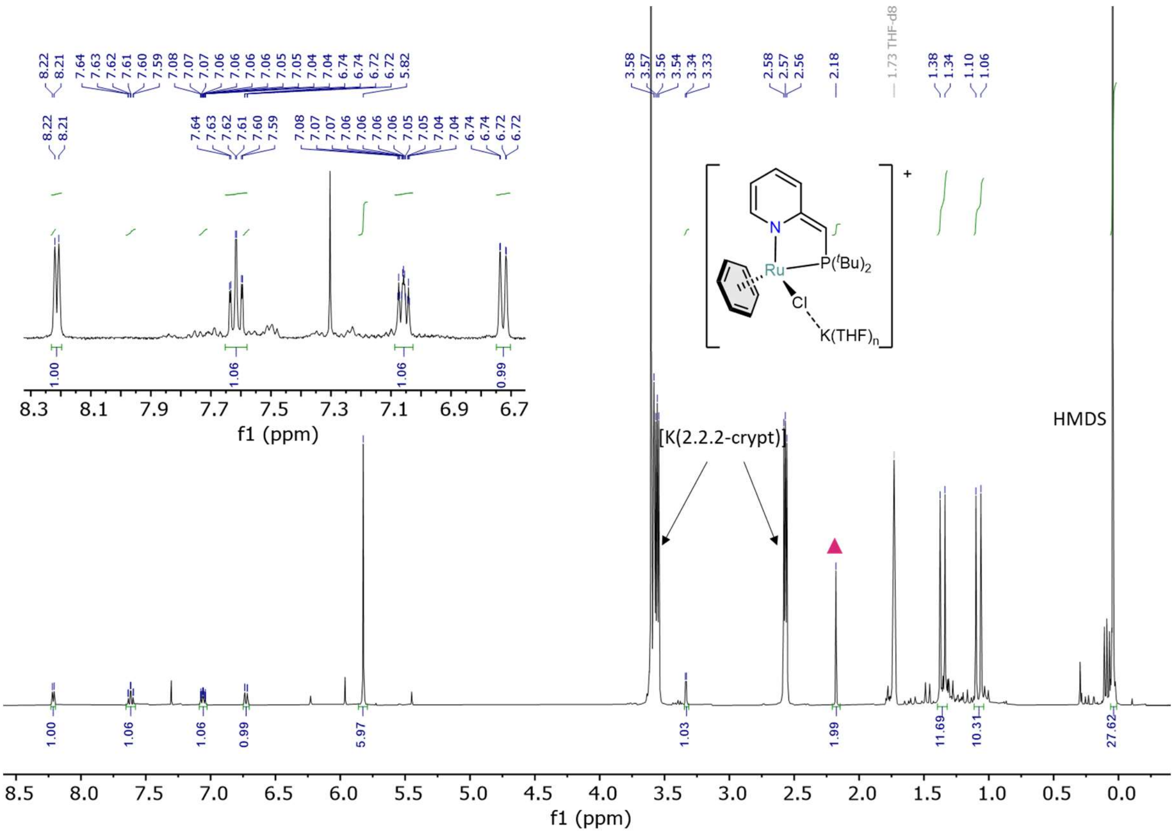

Figure S62. The crude ^{1}H NMR spectrum of complex **2-K** in THF-d_8 at 296 K. The internal standard (hexamethylbenzene) is highlighted with the triangle.

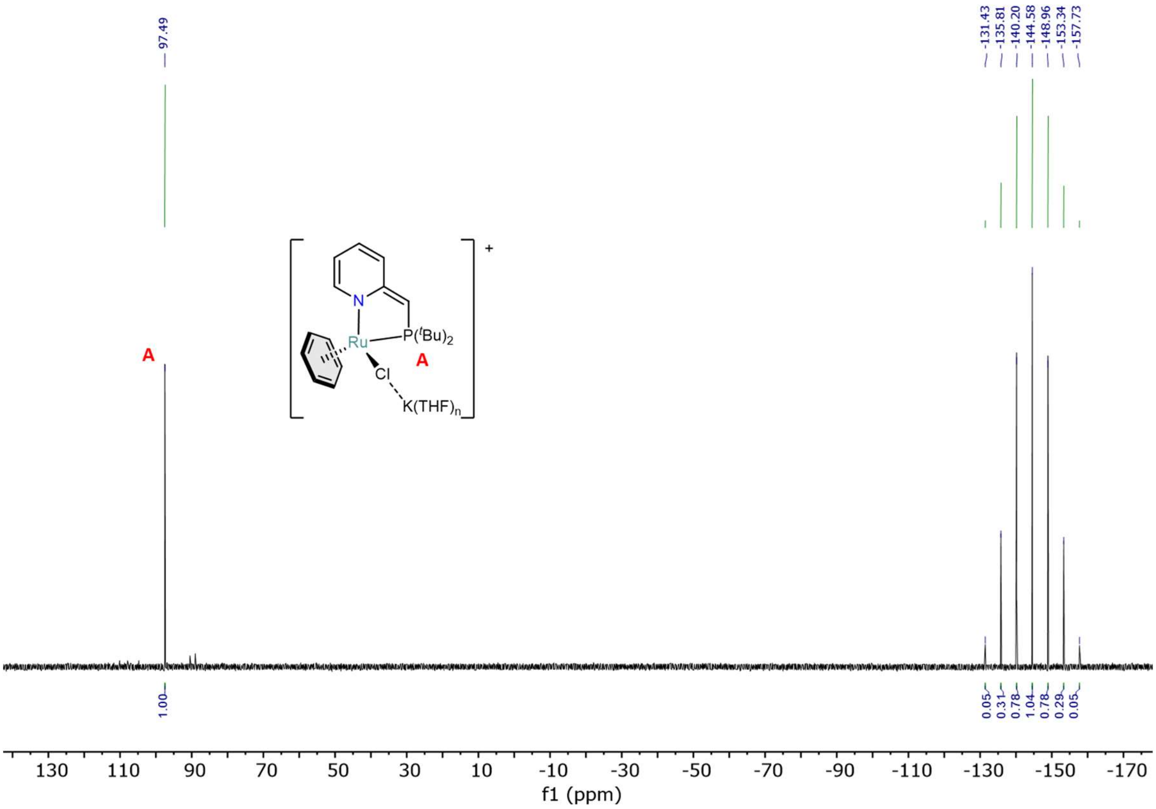

Figure S63. The crude ^{31}P NMR spectrum of complex **2-K** in THF-d_8 at 296 K.

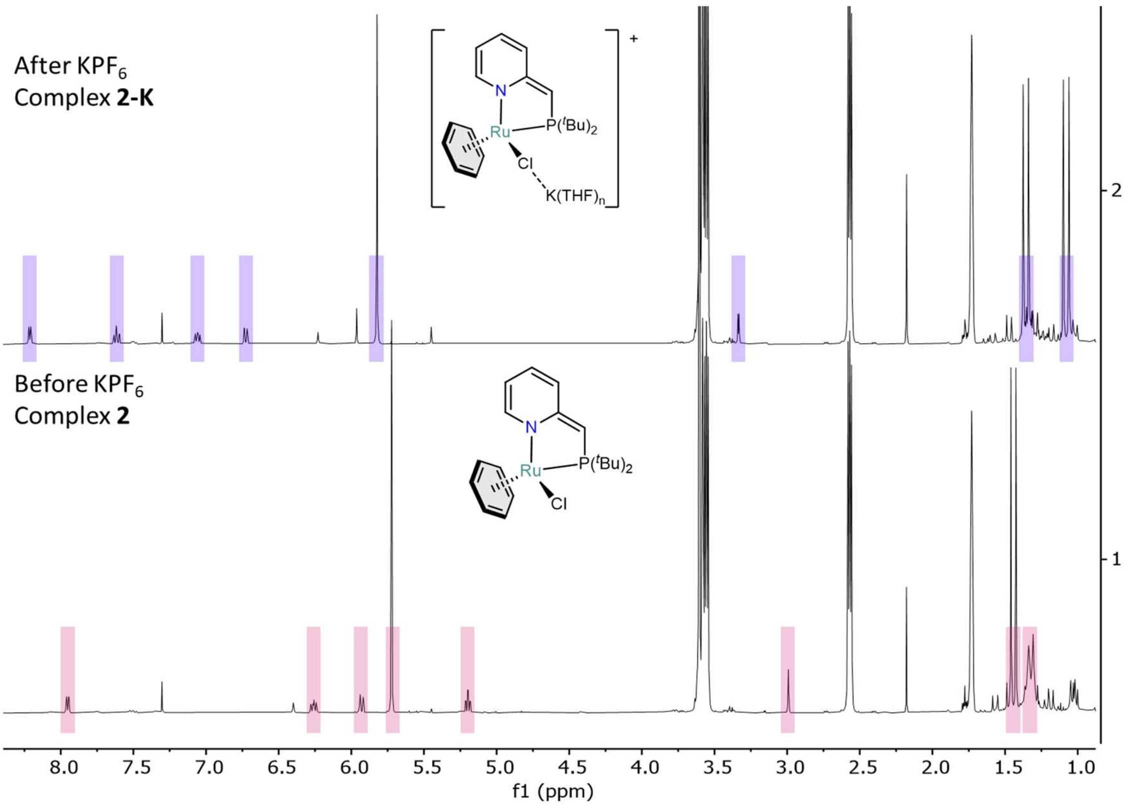

Figure S64. Overlap of the crude ^{1}H NMR spectra before and after the addition of KPF_6 (THF-d_8) at 296 K.

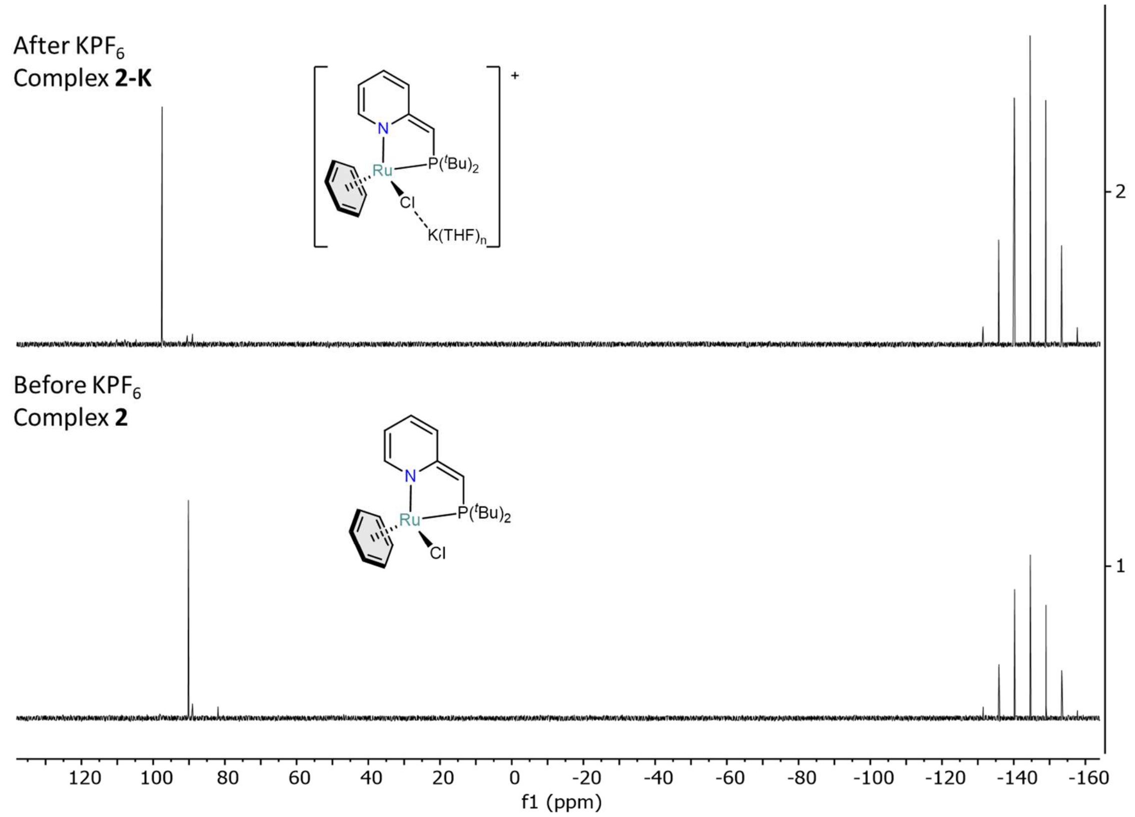

Figure S65. Overlap of the crude ^{31}P NMR spectra before and after the addition of KPF_6 (THF-d_8) at 296 K.

S2.2 Low-temperature NMR experiments

(^{tBu}PN)RuCl(η^5-(6-N,N-bis(trimethylsilyl)aminocyclohexadienyl) (Int1)

(Reaction between **1** and 1 equiv $KN(TMS)_2$)

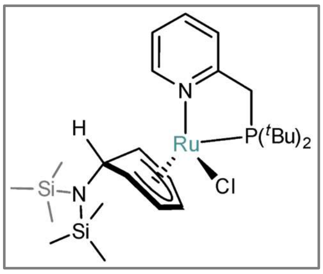

A yellow suspension of complex **1** (23.9 mg, 0.04 mmol) in THF-d_8 (1.5 mL) and a solution of $KN(TMS)_2$ (8.0 mg, 0.04 mmol) in THF-d_8 (1.5 mL) were cooled down in a cold well of a glove box (with an acetone/dry ice bath). After 10 minutes, the $KN(TMS)_2$ solution was added dropwise to the suspension of **1** using a pipette (precooled in the cold well) resulting in an instant color change to dark greenish/brown. The reaction mixture was stirred for 15 minutes at –78°C (external temperature) in the cold well. An aliquot (~ 0.8 mL) was transferred to a precooled J. Young NMR inside a precooled aluminum holder. Directly after taking the sample out of the glove box, it was submerged in an acetone/dry ice bath from which it was transferred into a precooled (–60°C) NMR machine to record the spectra.

^{1}H NMR (400 MHz, THF-d_8, 213K): δ = 8.37 (d, $^3J_{H,H}$ = 5.5 Hz, 1H), 7.60 (dd, $^3J_{H,H}$ = 9.2, $^3J_{H,H}$ = 7.3 Hz, 1H), 7.39 (d, $^3J_{H,H}$ = 7.3 Hz, 1H), 7.00 (dd, $^3J_{H,H}$ = 8.4, $^3J_{H,H}$ = 6.0 Hz, 1H), 5.10 (s, br, 1H), 4.88 (dd, $^3J_{H,H}$ = 7.6, $^3J_{H,H}$ = 5.2 Hz, 1H), 4.79 (dd, $^3J_{H,H}$ = 7.4, $^3J_{H,H}$ = 5.0 Hz, 1H), 4.70 (dd, $^3J_{H,H}$ = 11.4, $^3J_{H,H}$ = 5.0 Hz, 1H), 3.68 – 3.57 (dd, partially overlapped with THF, 2H), 3.40 (dd, $^2J_{H,P}$ = 15.6, $^2J_{H,H}$ = 11.2 Hz, 1H), 2.76 (dd, $^3J_{H,H}$ = 7.7, $^3J_{H,H}$ = 5.8 Hz, 1H), 1.37 (d, $^3J_{H,P}$ = 12.2 Hz, 9H), 1.02 (d, br, $^3J_{H,P}$ = 9.9 Hz, 9H), 0.12 (s, 18H).

^{31}P{^{1}H} NMR (162 MHz, THF-d_8, 213K): δ = 83.0 (s, 1P), –144.8 (hept, $^1J_{P,F}$ = 710.1 Hz, 1P).

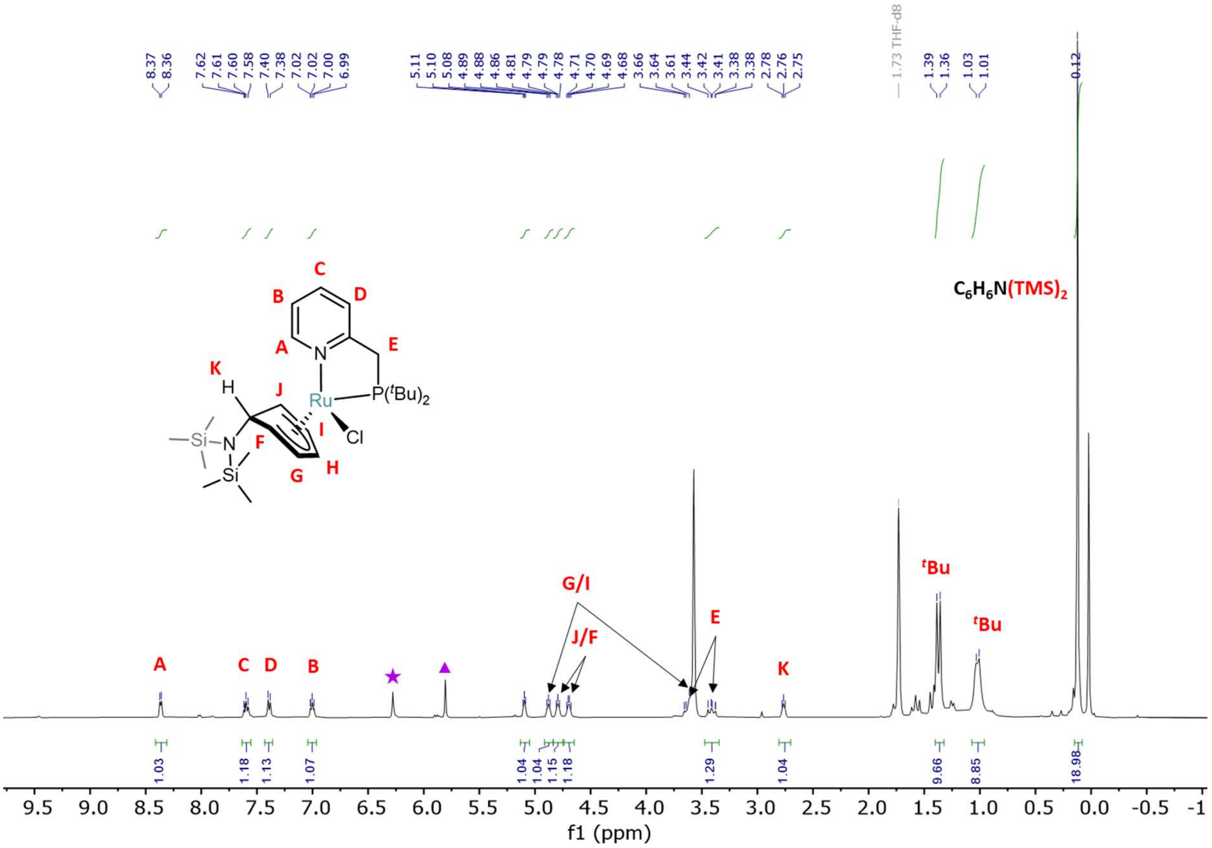

Figure S66. The ^{1}H NMR spectrum of complex **Int1** in THF-d_8 at 213 K. A small amount of unreacted compound **1** is highlighted with the star, the triangle highlights **2-K**.

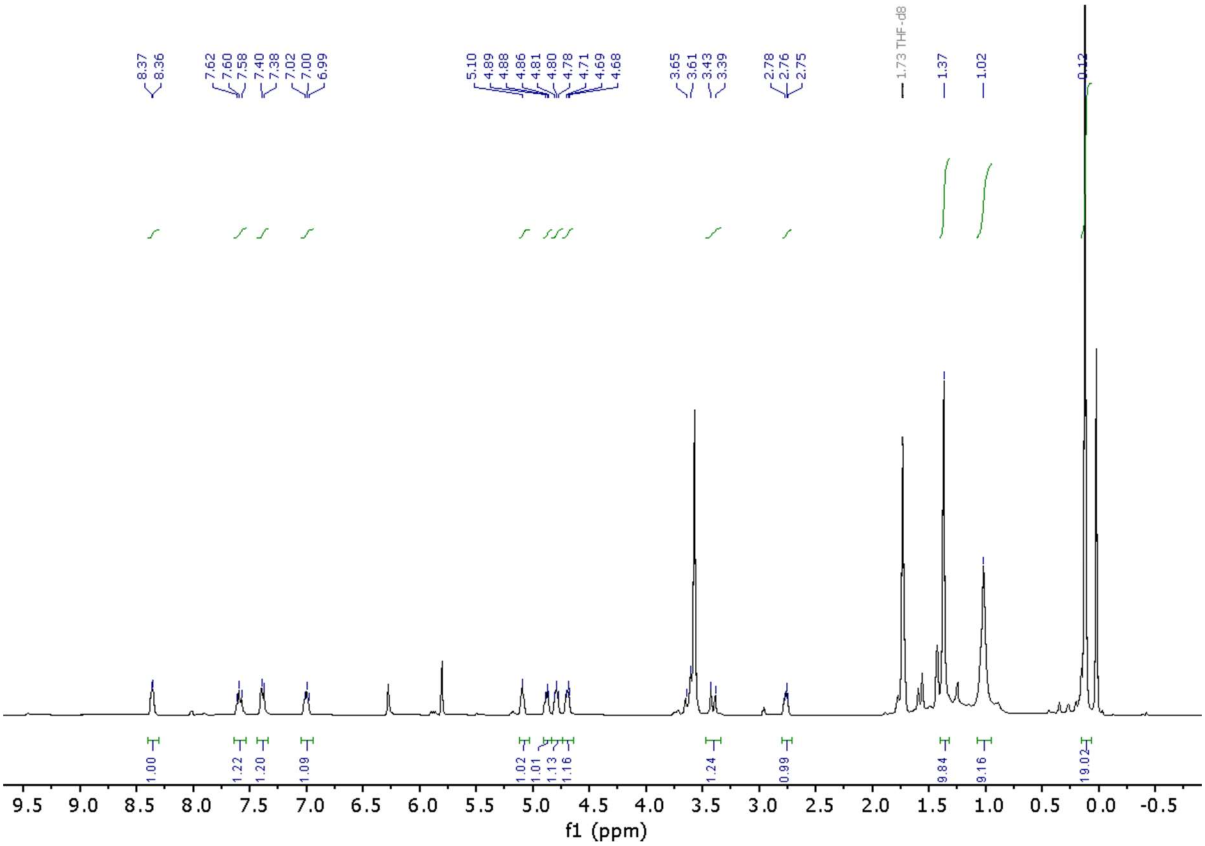

Figure S67. The $^{1}H\{^{31}P\}$ NMR spectrum of complex **Int1** in THF-d_8 at 213 K.

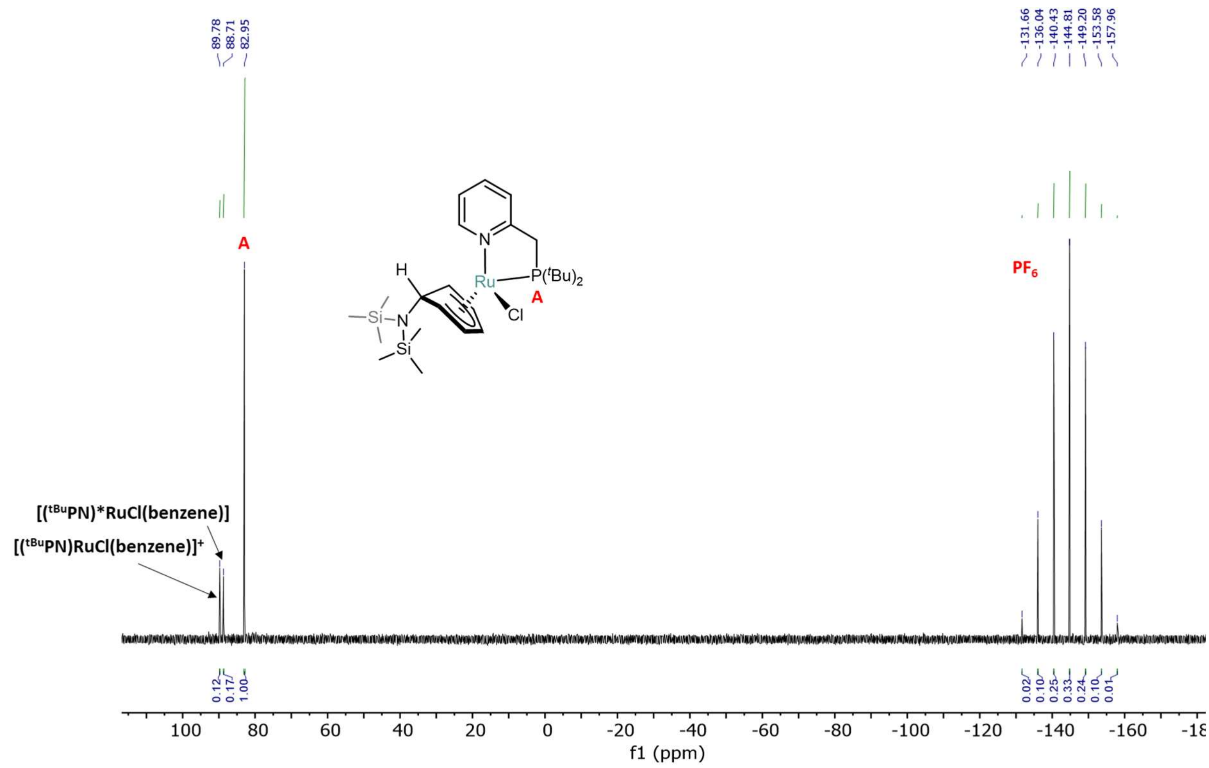

Figure S68. The $^{31}P\{^{1}H\}$ NMR spectrum of complex **Int1** in THF-d_8 at 213 K.

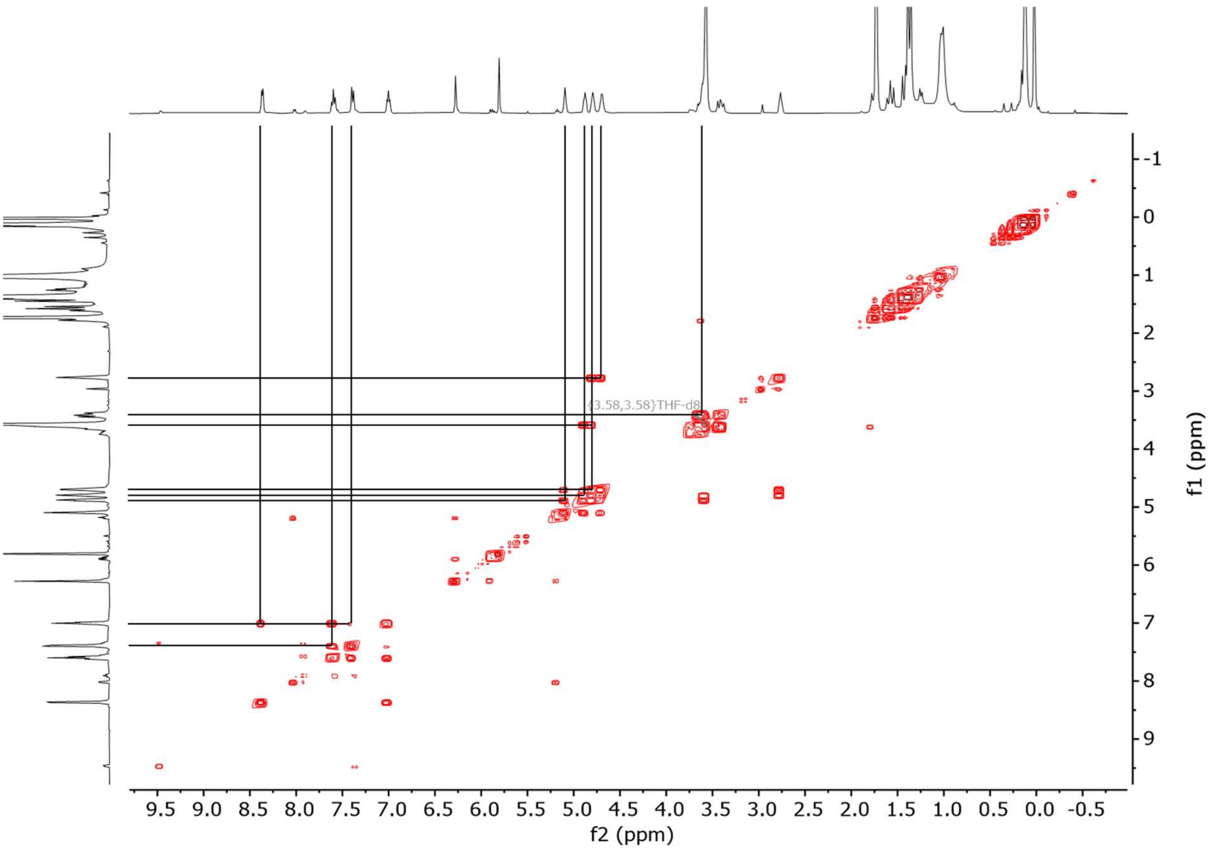

Figure S69. The ^{1}H-^{1}H COSY NMR spectrum of complex **Int1** in THF-d_8 at 213 K.

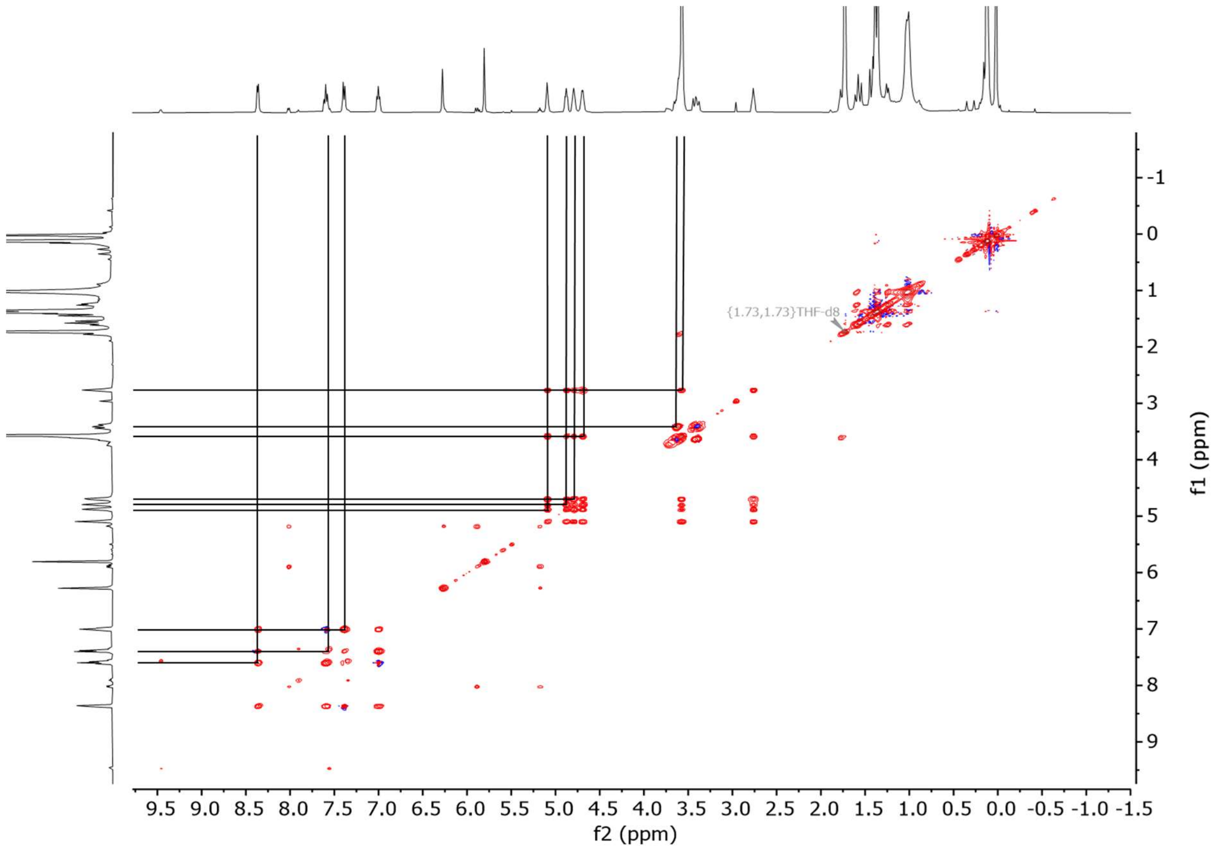

Figure S70. The ^{1}H-^{1}H TOCSY NMR spectrum of complex **Int1** in THF-d_8 at 213 K.

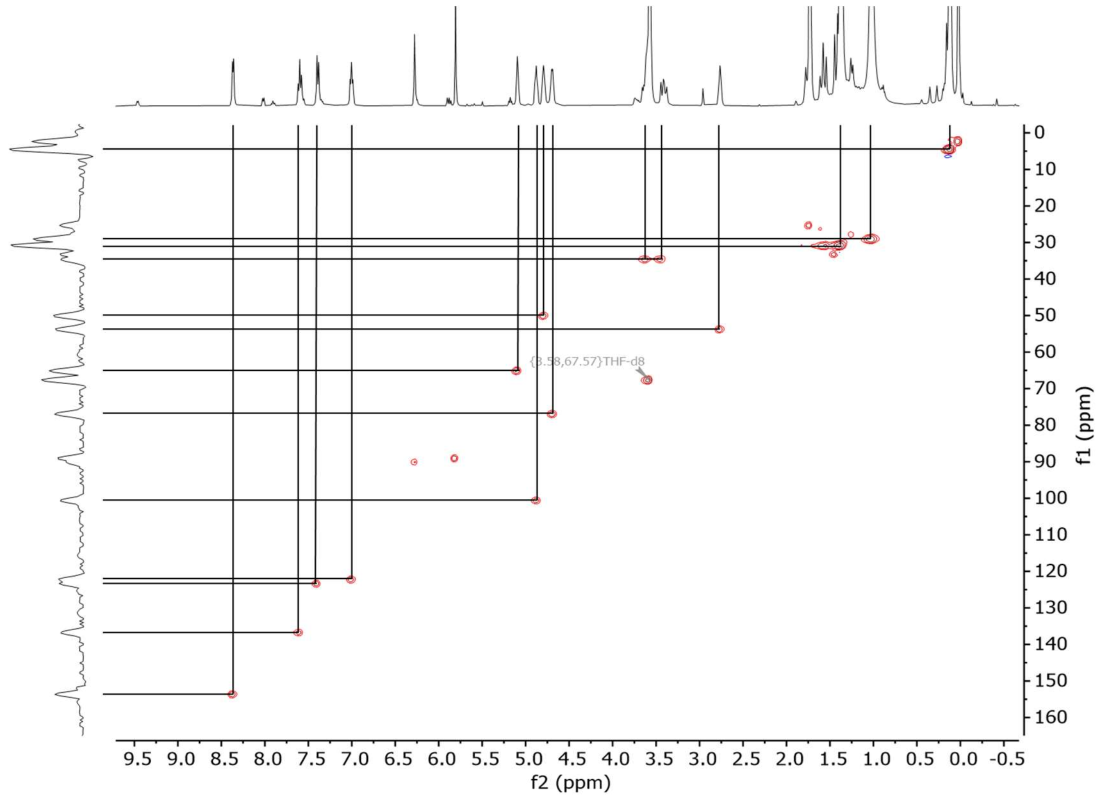

Figure S71. The ^{1}H-^{13}C ASAP-HMQC NMR spectrum of complex **Int1** in THF-d_8 at 213 K.
NOTE f1 axis is depicted as the projection of the HMQC cross peaks.

Warming up the reaction mixture to room temperature.

Upon warming up the mixture to room temperature, the transformation of compound **Int1** to compound **2-K** was observed. NMR spectrum recorded after 16 hours after the low-temperature experiments showed almost only the deprotonated species **2-K**.

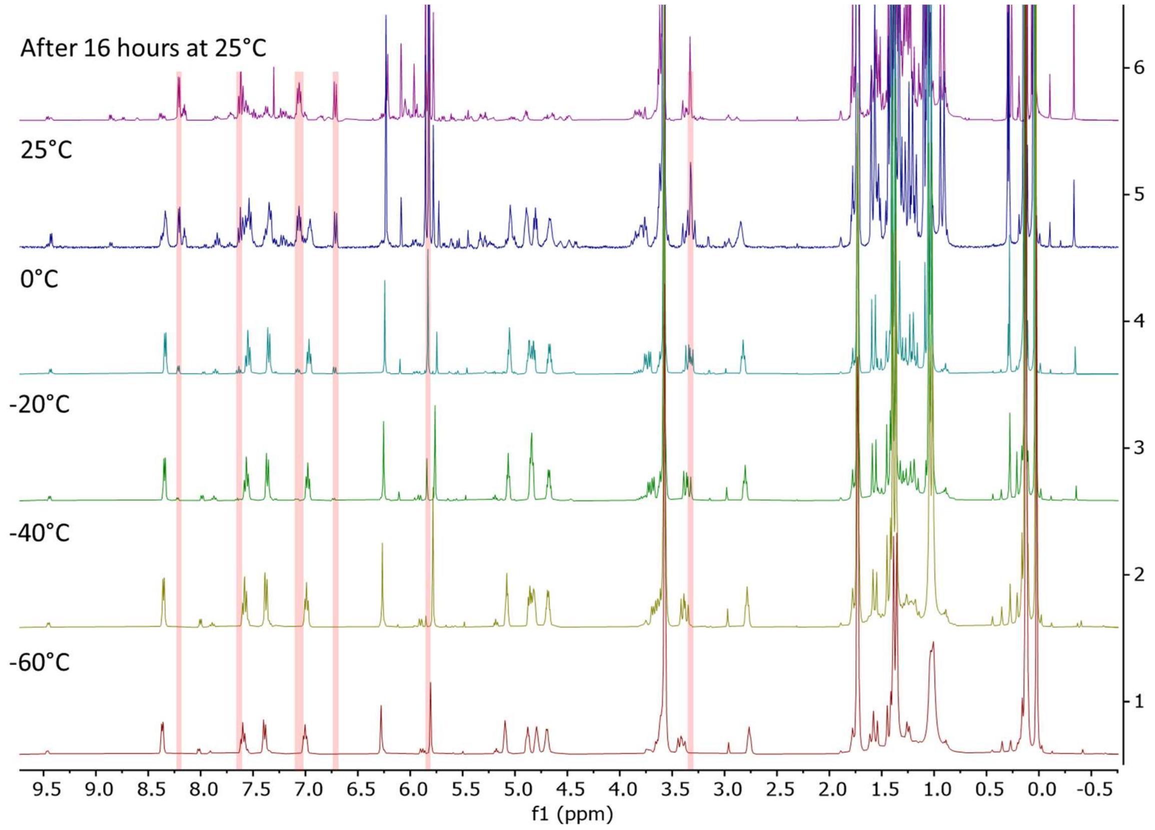

Figure S72. The ^{1}H NMR spectra of complex **Int1** upon warming it up from −60°C to 25°C. The signals of complex **2-K** are highlighted in red.

(^{tBu}PN*)Ru(η^5-(6-N,N-bis(trimethylsilyl)aminocyclohexadienyl) (Int2)
(Reaction between **1** and 2 equiv KN(TMS)$_2$)

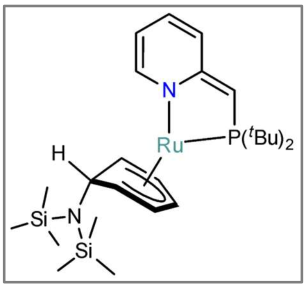

A yellow suspension of complex **1** (23.9 mg, 0.04 mmol) in THF-d_8 (1.5 mL) and a solution of KN(TMS)$_2$ (16.0 mg, 0.04 mmol) in THF-d_8 (1.5 mL) were cooled down in a cold well of a glove box (with an acetone/dry ice bath). After 10 minutes, the KN(TMS)$_2$ solution was added dropwise to the suspension of **1** using a pipette (precooled in the cold well) resulting in an instant color change to dark green. The reaction mixture was stirred for 15 minutes at –78 °C (external temperature) in the cold well. An aliquot (~ 0.8 mL) was transferred in a precooled J. Young NMR inside a precooled aluminum holder. Directly after taking the sample out of the glove box ,it was submerged in an acetone/dry ice bath from which it was transferred into a precooled (–60°C) NMR machine to record the spectra.

Note: It was noticed that the color of the reaction mixture is orange at –78 °C (i.e. being submerged in a dry ice/acetone bath). When the temperature rises to –60 °C, the solution becomes dark green (almost black) and further warming up causes a rapid color change to bright orange/brown within a minute.

*Due to the exceptionally unstable nature of the compound, our attempts did not lead to cleaner NMR spectra (for the high reactivity of **Int2** see the spectra at different temperatures).*

^{1}H NMR (400 MHz, THF-d_8, 213K): δ 6.76 (s, br, 1H), 6.52 – 5.42 (m, br, 1H), 6.29 – 6.13 (m, br, overlapped with compound **2**, 3H), 5.37 (s, br, 1H), 5.31 – 5.24 (m, br, 1H), 5.05 – 5.92 (m, overlapped with compound **2,** 1H), 4.78 (s, br, 1H), 4.11 (s, br, 1H), 3.67 (s, br, partially overlapped with THF, 1H), 3.24 (s, br, 1H), 3.09 (s, overlapped with compound **2**, 1H), 1.27 (d, br, overlapped with compound **2**,$^3J_{H,P}$ = 12.6 Hz, 9H), 1.15 (d, br, overlapped with compound **2**, $^3J_{H,P}$ = 12.9 Hz, 9H), 0.18 (s, 18H).

^{31}P{^{1}H} NMR (162 MHz, THF-d_8, 213K): δ = 82.3 (s, 1P), –143.0 (hept, $^1J_{P,F}$ = 710.5 Hz, 1P).

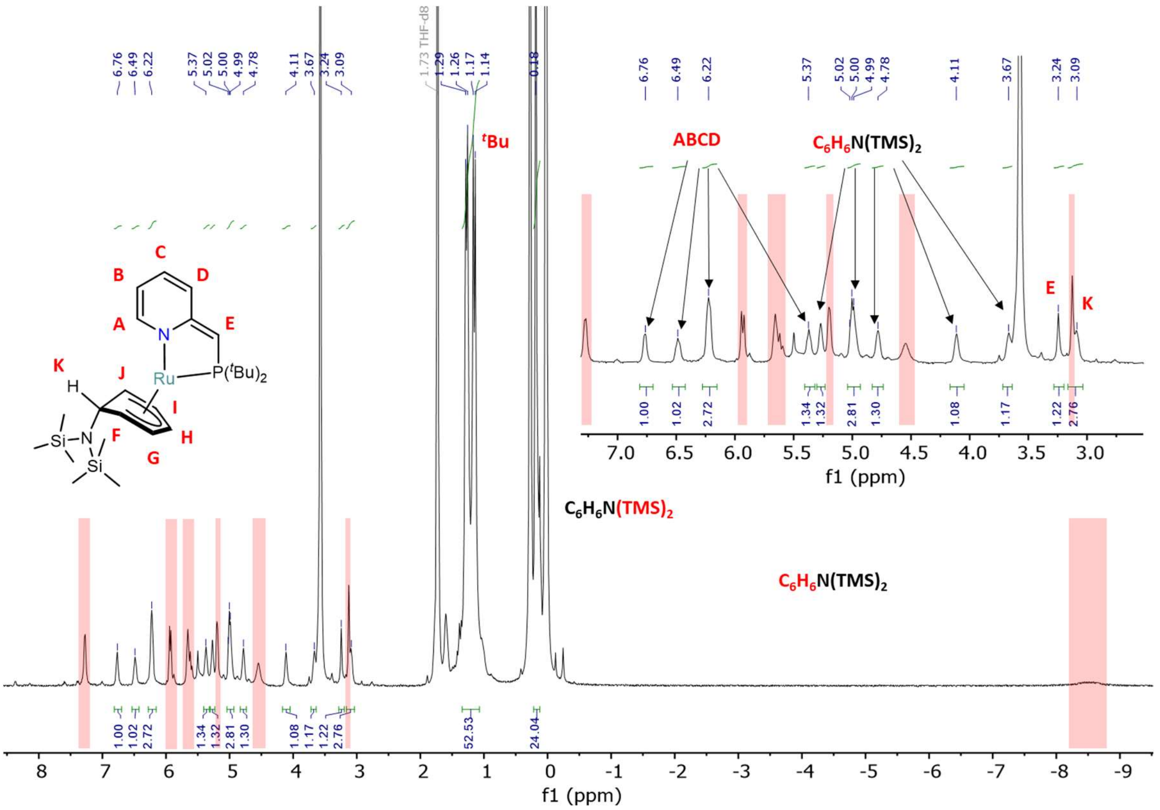

Figure S73. The ^{1}H NMR spectrum of complex **Int2** in THF-d_8 at 213 K. The red boxes highlight the presence of compound **4**.

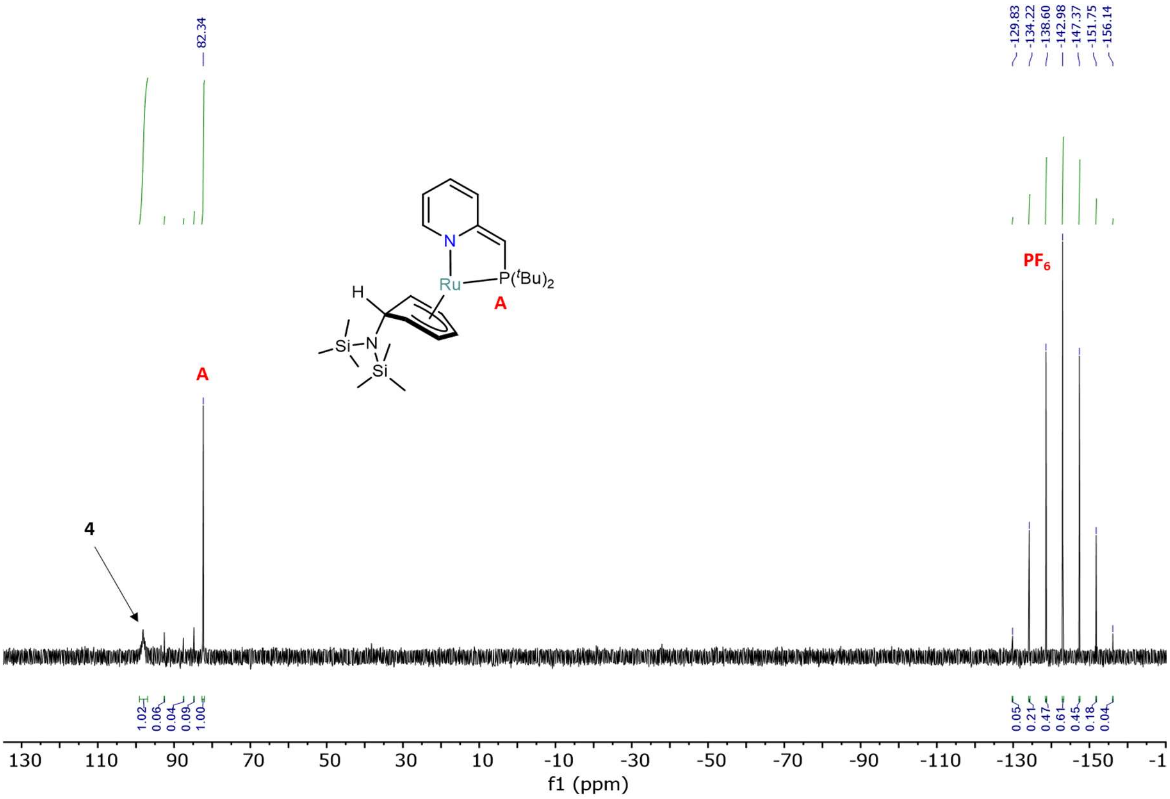

Figure S74. The $^{31}P\{^{1}H\}$ NMR spectrum of complex **Int2** in THF-d_8 at 213 K.

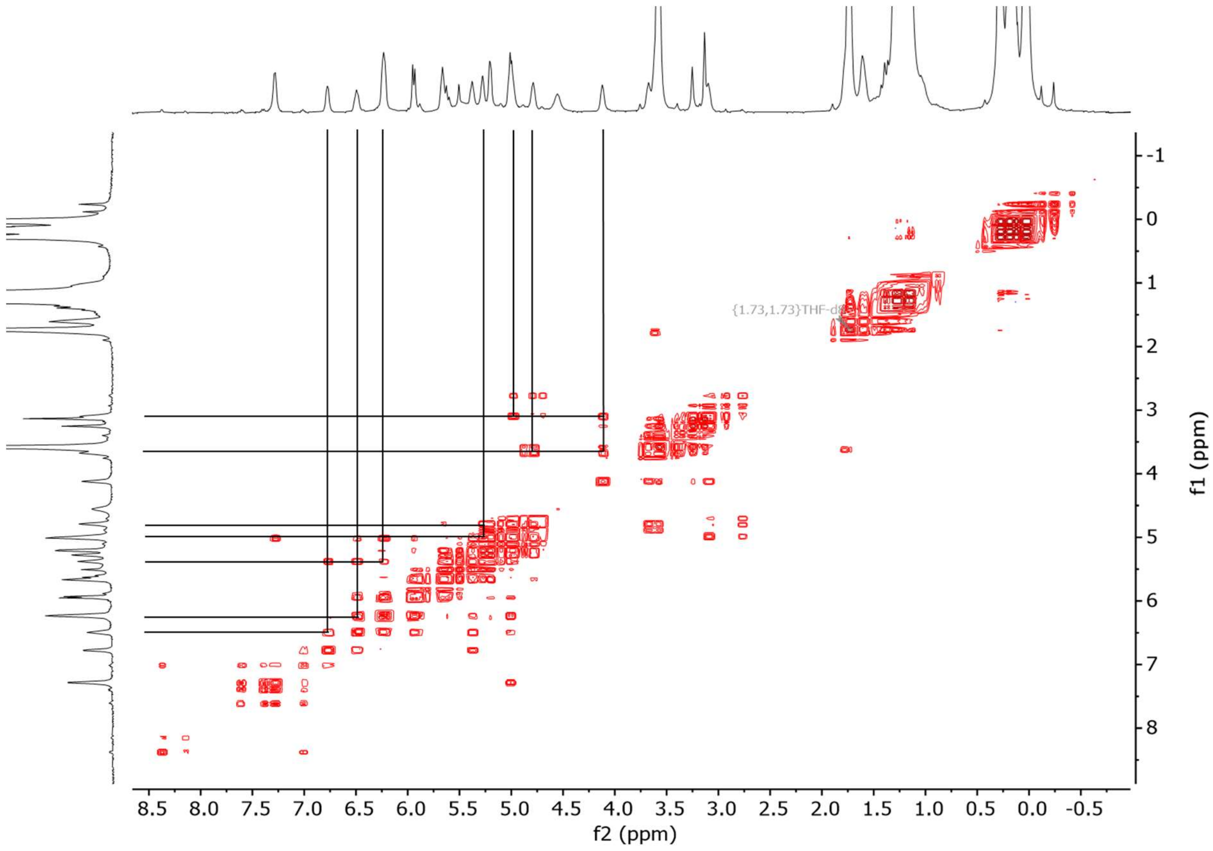

Figure S75. The ^{1}H-^{1}H gCOSY NMR spectrum of complex **Int2** in THF-d_8 at 213 K.

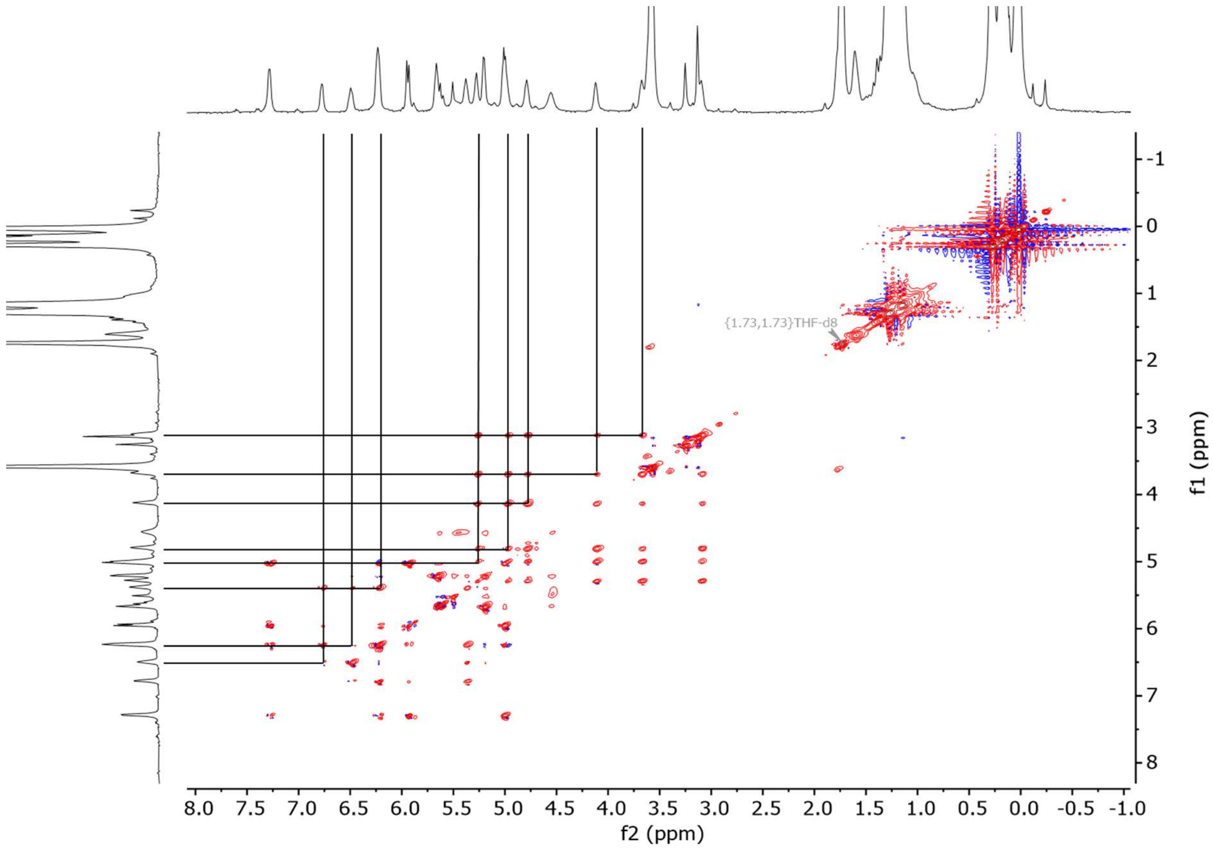

Figure S76. The ^{1}H-^{1}H TOCSY NMR spectrum of complex **Int2** in THF-d_8 at 213 K.

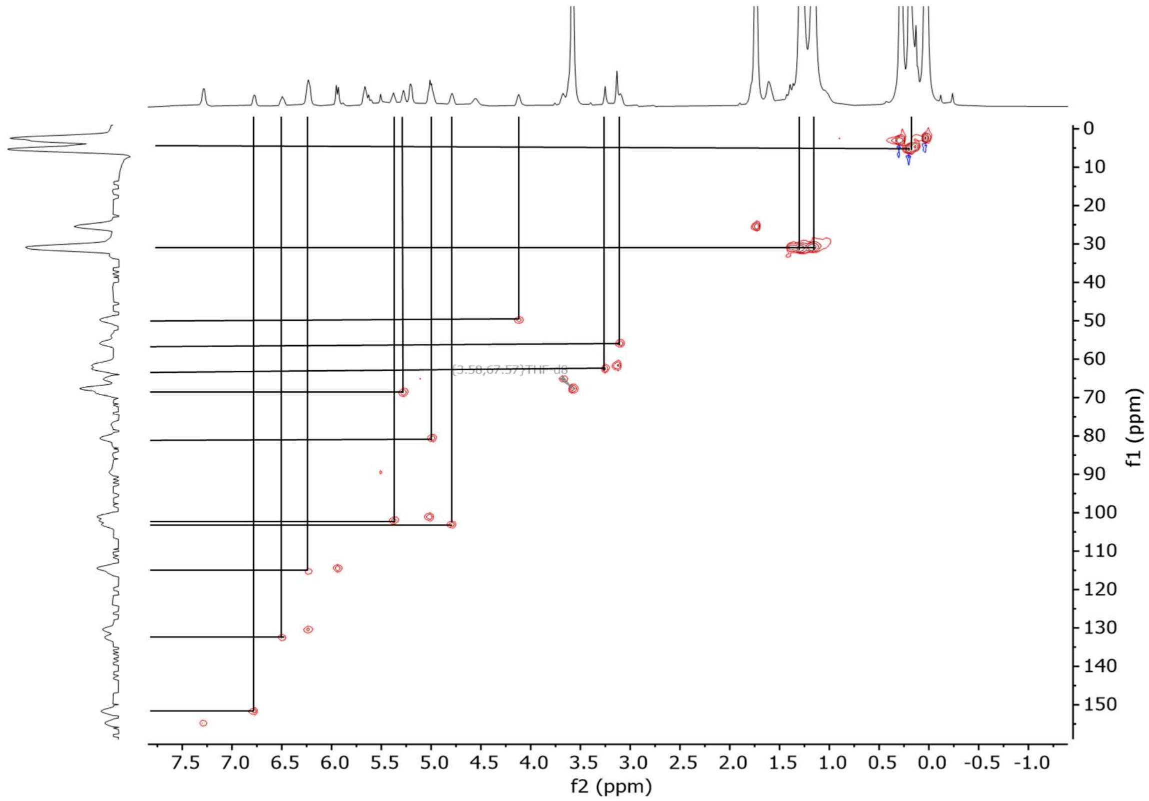

Figure S77. The ^{1}H-^{13}C ASAP-HMQC NMR spectrum of complex **Int2** in THF-d_8 at 213 K. *NOTE: f1 axis is depicted as the projection of the HMQC cross peaks.*

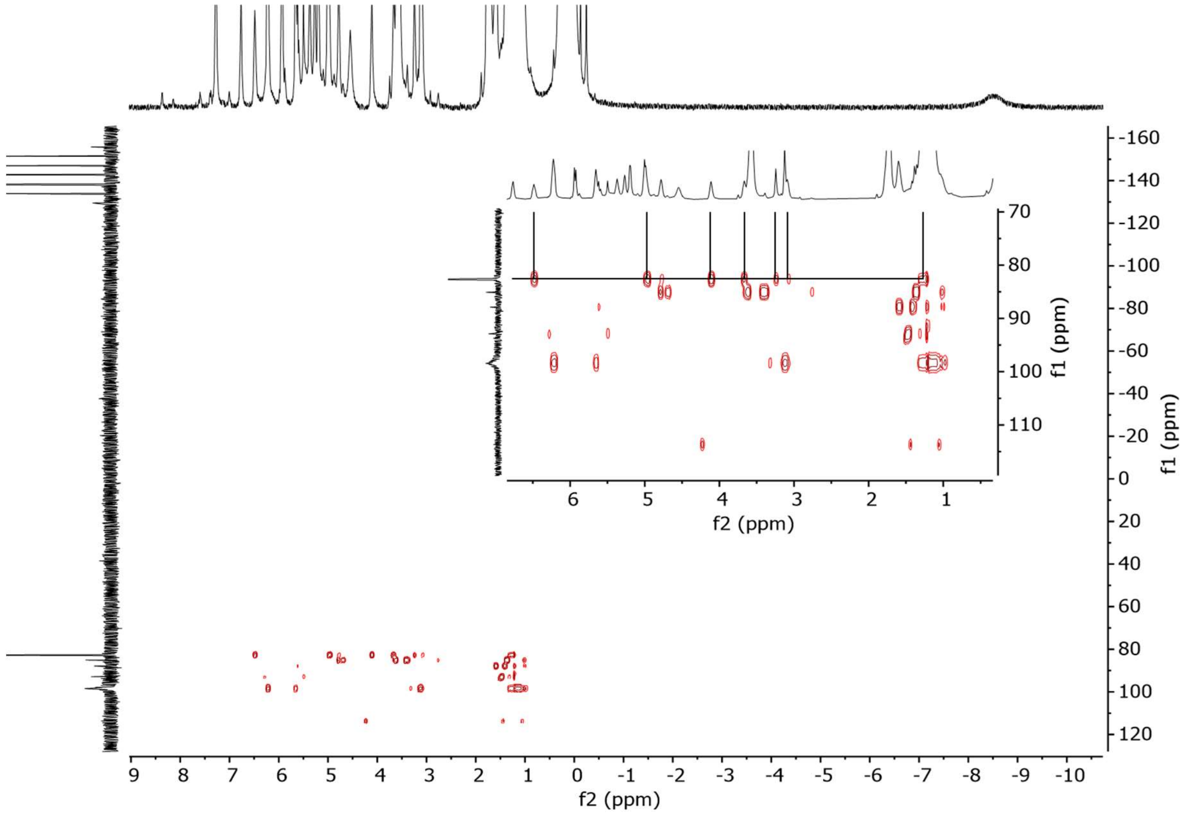

Figure S78. The ^{1}H-^{31}P HMBC NMR spectrum of complex **Int2** in THF-d_8 at 213 K.

Warming up the reaction mixture to room temperature.

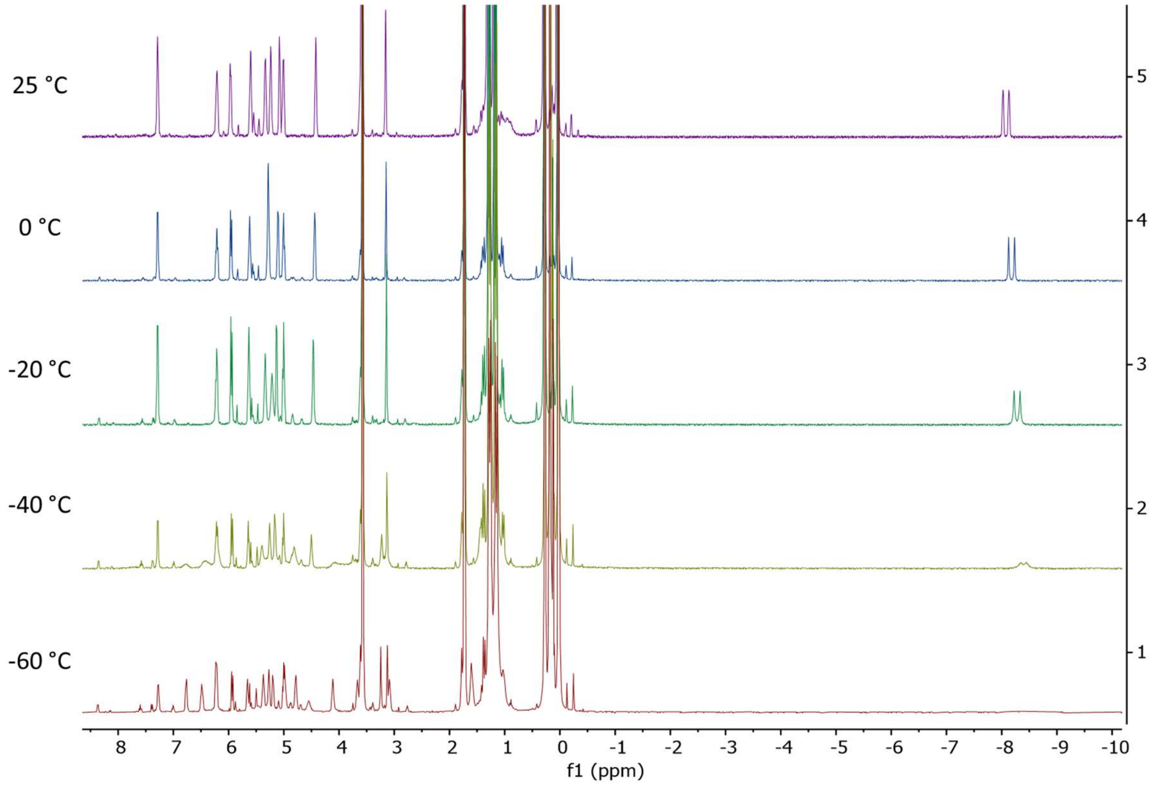

Figure S79. The ^{1}H NMR spectrum of complex **Int2** in THF-d_8 upon warming up from 213 K to RT. *It is noticeable that already at 233 K a considerable part of the complex* ***Int2*** *transformed into species* ***4.***

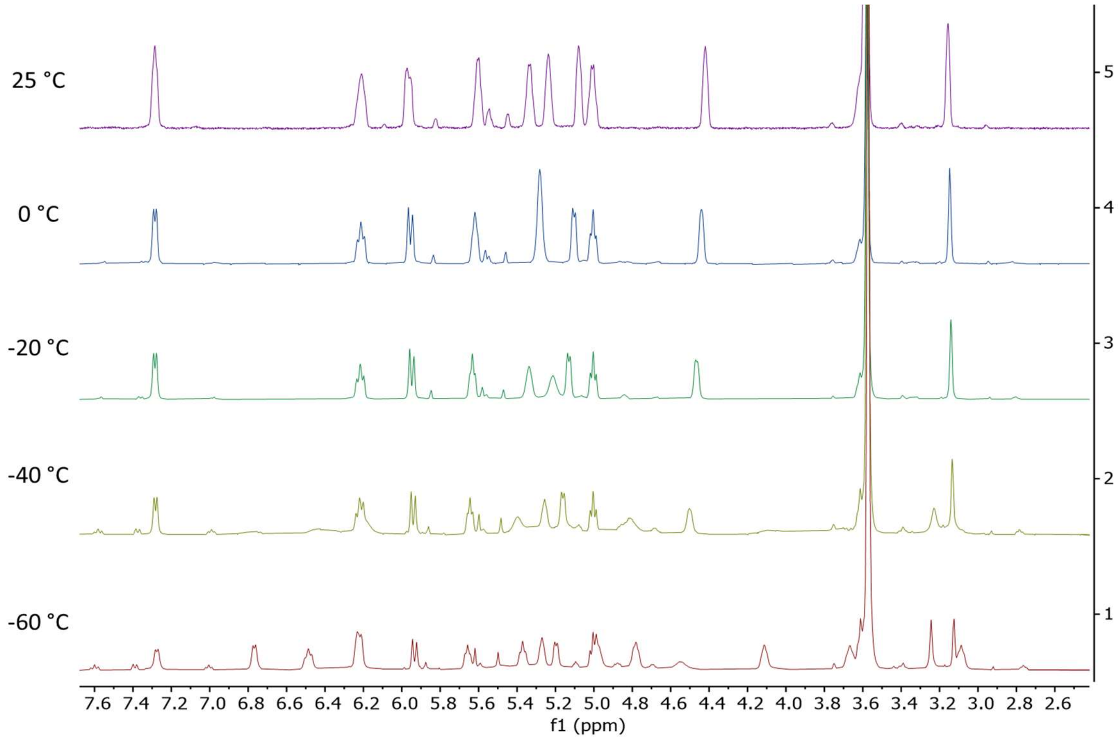

Figure S80. The ^{1}H NMR spectrum of complex **Int2** in THF-d_8 upon warming up from 213 K to RT (zoomed aromatic region).

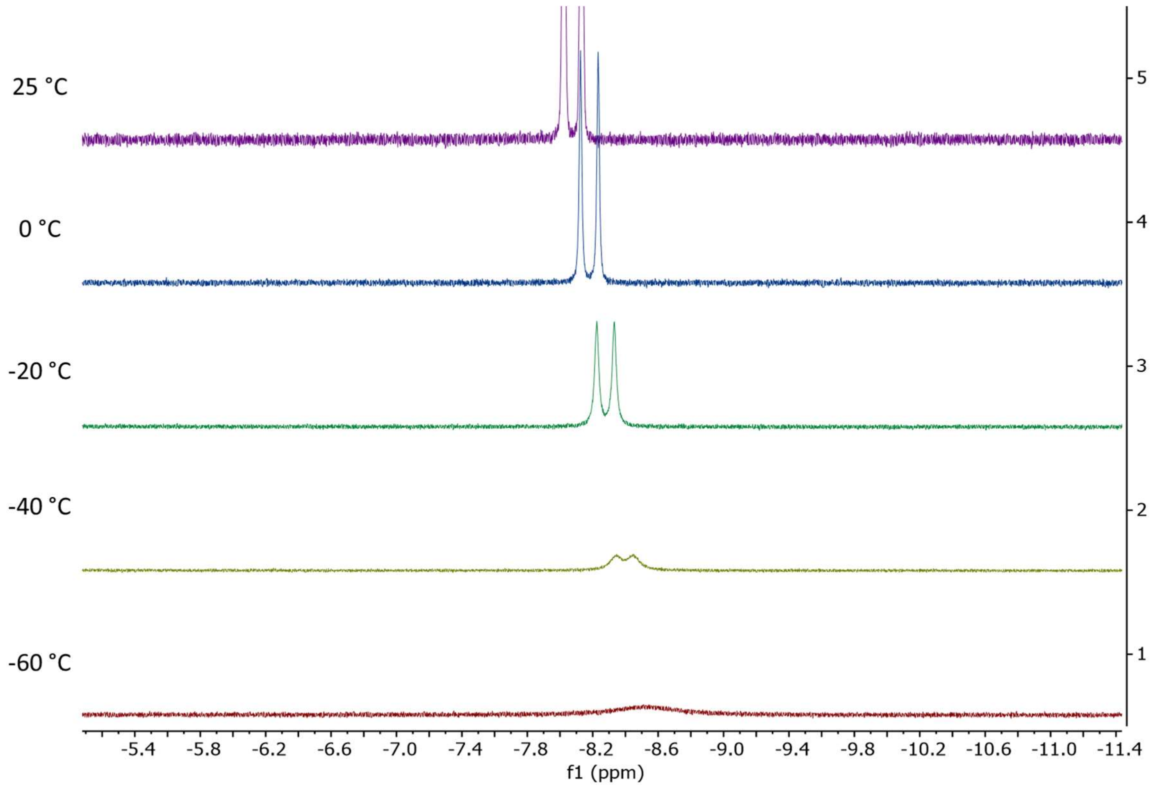

Figure S81. The ^{1}H NMR spectrum of complex **Int2** in THF-d_8 upon warming up from 213 K to RT (zoomed hydride region).

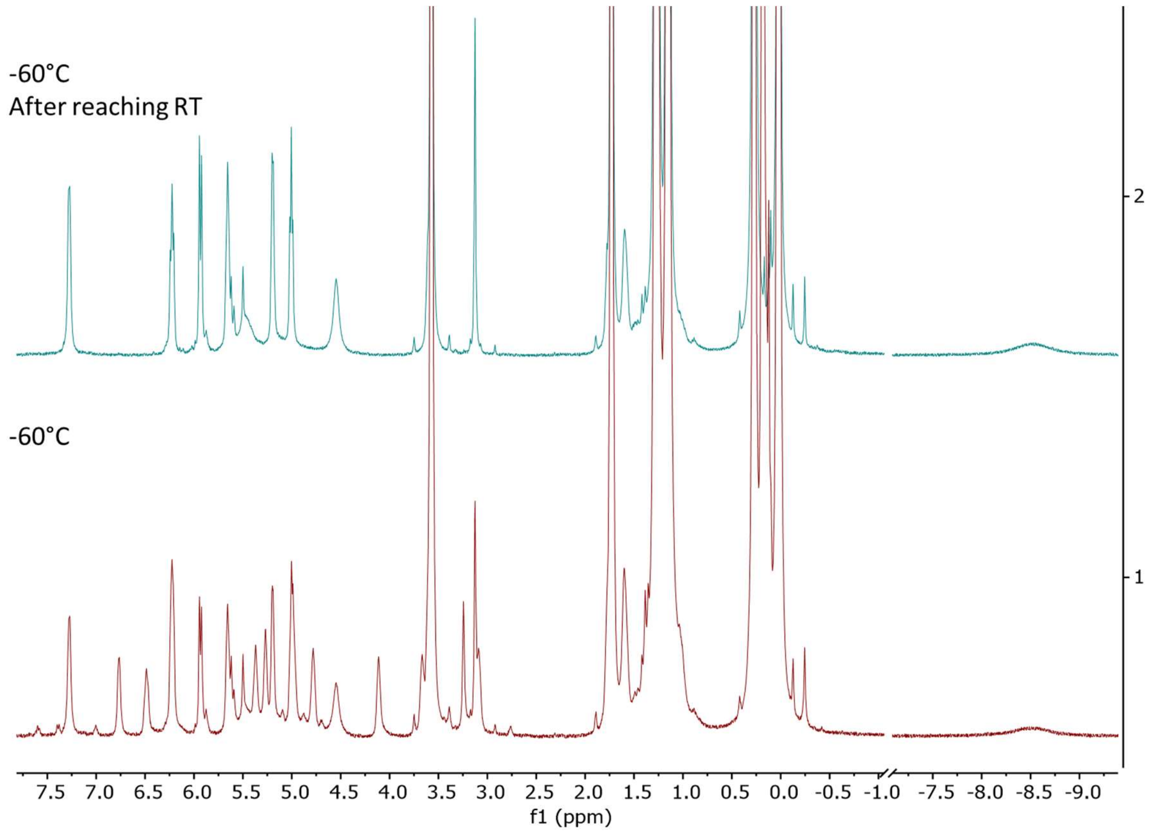

Figure S82. The ^{1}H NMR spectrum of the reaction mixture of **Int2** in THF-d_8 at 213 K right after the reaction (bottom) and after reaching RT (top).

Synthesis of 4-D at low temperature

To see whether performing the reaction between compound **1-D** and 2 equiv of $KN(TMS)_2$ leads to different H/D ratio of the hydride moiety in complex **4-D**, we repeated the synthesis of **4-D** –78 °C inside a glovebox cold-well. , ^1H qNMR analysis of the reaction product shows that the H/D ratio does not change significantly for the hydride ligand (56% "D" for **4-D** made at RT *vs* 60% "D" for **4-D** made at –78 °C), which is within the experimental error of the measurement.

Procedure: A yellow suspension of complex **1-D** (30.0 mg, 0.05 mmol) in THF (1.5 mL) and a solution of $KN(TMS)_2$ (19.9 mg, 0.1 mmol) in THF (1.5 mL) were cooled down in a cold well of a glove box (with an acetone/dry ice bath). After 10 minutes, the $KN(TMS)_2$ solution was added dropwise to the suspension of **1** using a pipette (precooled in the cold well) resulting in an instant color change to dark green. The reaction mixture was stirred for 15 minutes at –78 °C (external temperature) in the cold well. The acetone/dry ice bath was removed and the vial was allowed to gradually warm to room temperature in the cold well over 2 hours. An aliquot (~ 0.7 mL) of formed dark brown solution was transferred into a J. Young tube for NMR analysis.

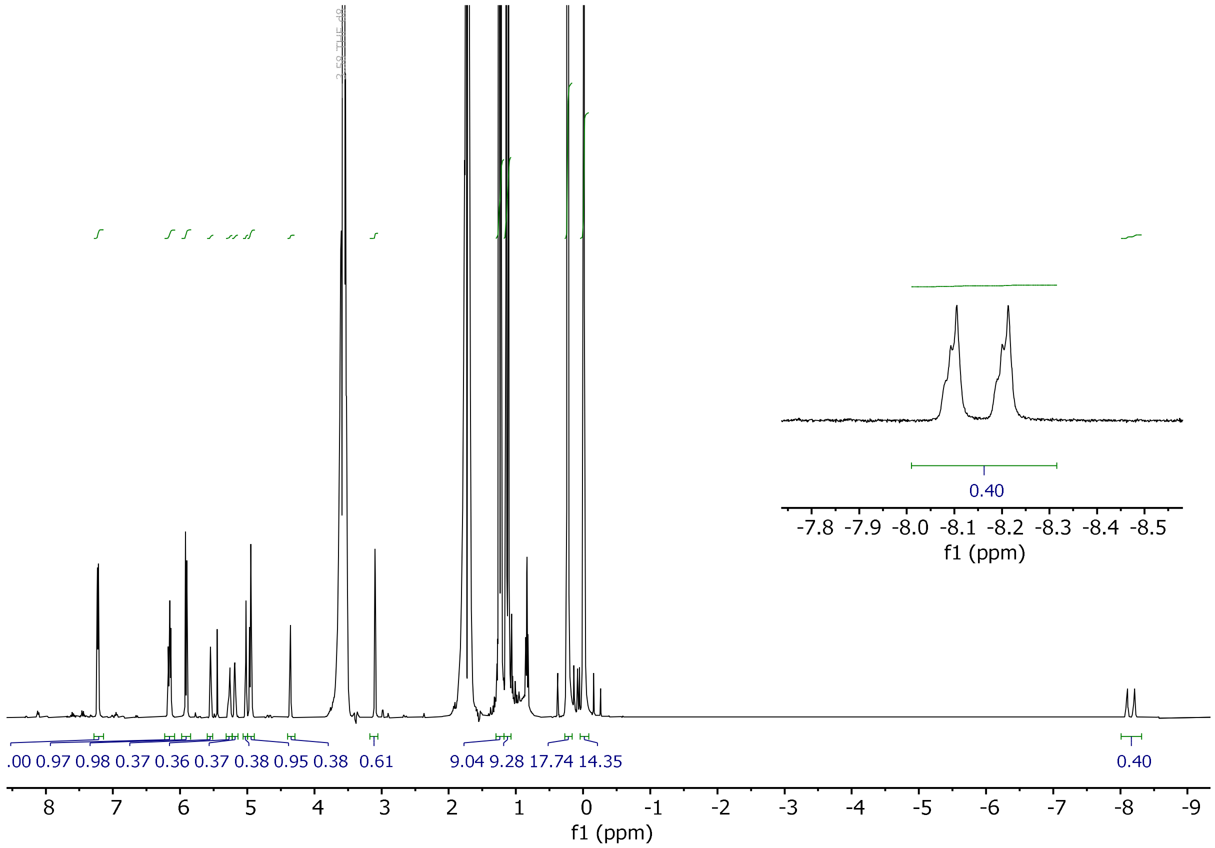

Figure S83. The ^1H qNMR spectrum of complex **4-D** synthesized at low-temperature measured at 298 K in THF (WET function was used to suppress the solvent signals)

S2.3 Experiments with other ligands

To get more insights if the found S_NArH reactivity is limited to the PN ligand or can be also extended to other ligands, we performed S_NArH reactions with five more complexes featuring different ligands (Fig. S83).

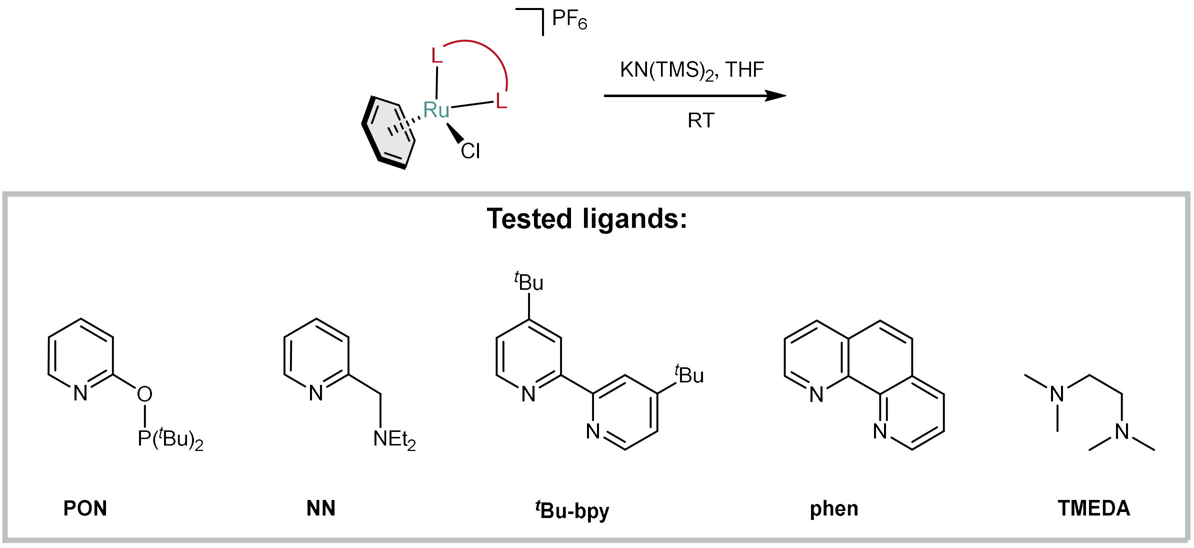

Figure S84. S_NArH experiments with other ligands.

[(PON)RuCl(C_6H_6)]PF_6

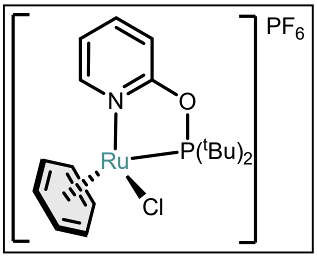

A round-bottom flask was charged with [Ru(C_6H_6)Cl_2]$_2$ (673.5 mg, 1.35 mmol) DCM (30.0 mL), followed by the PON ligand (640.0 mg, 2.68 mmol). The resulting brown suspension was stirred for 2 hours at room temperature and most of the solids dissolved. After 2 hours of stirring, KPF_6 (746.8 mg, 4.06 mmol) was added to the reaction mixture, which was stirred at room temperature for another two days. The reaction mixture was subsequently filtered over a glass filter to remove any KCl, unreacted KPF_6 and [Ru(C_6H_6)Cl_2]$_2$, which led to a dark green filtrate. Removing the volatiles under dynamic vacuum gave green and yellow solids, which were suspended in 12.0 mL of THF. The suspension was filtered over a glass filter and the residue was washed with 10.0 mL *n*-pentane and 5.0 mL THF and was subsequently dried under dynamic vacuum to obtain 713.0 mg of a bright orange powder (44.2%).

^{1}H NMR (400 MHz, CD_3CN, 298K): δ = 8.92 (ddt, *J* = 6.0, 1.2, 0.6 Hz, 1H), 7.94 (dddd, *J* = 8.3, 7.3, 1.8, 0.8 Hz, 1H), 7.20 – 7.12 (m, 2H), 6.20 (d, $^3J_{H,P}$ = 0.6 Hz, 6H), 1.63 (d, $^3J_{H,P}$ = 16.2 Hz, 9H), 1.31 (d, $^3J_{H,P}$ = 14.8 Hz, 9H).

^{13}C{^{1}H} NMR (100 MHz, CD_3CN, 298K): δ = 165.6 (s), 156.0 (d, $^4J_{C,P}$ = 2.5 Hz), 144.7 (s), 121.6 (s), 113.1 (d, $^3J_{C,P}$ = 4.5 Hz), 92.5 (d, $^2J_{C,P}$ = 3.0 Hz), 45.3 (d, $^1J_{C,P}$ = 12.5 Hz), 43.4 (d, $^1J_{C,P}$ = 13.3 Hz), 31.2 (d, $^2J_{C,P}$ = 4.8 Hz), 27.8 (d, $^2J_{C,P}$ = 4.5 Hz).

^{31}P{^{1}H} NMR (162 MHz, CD_3CN, 298K): δ = 222.4 (s, 1P), –144.6 (hept, $^1J_{P,F}$ = 707.3 Hz, 1P).

^{19}F NMR (376 MHz, CD_3CN, 298K): δ = –72.7 (d, $^1J_{F,P}$ = 706.4 Hz).

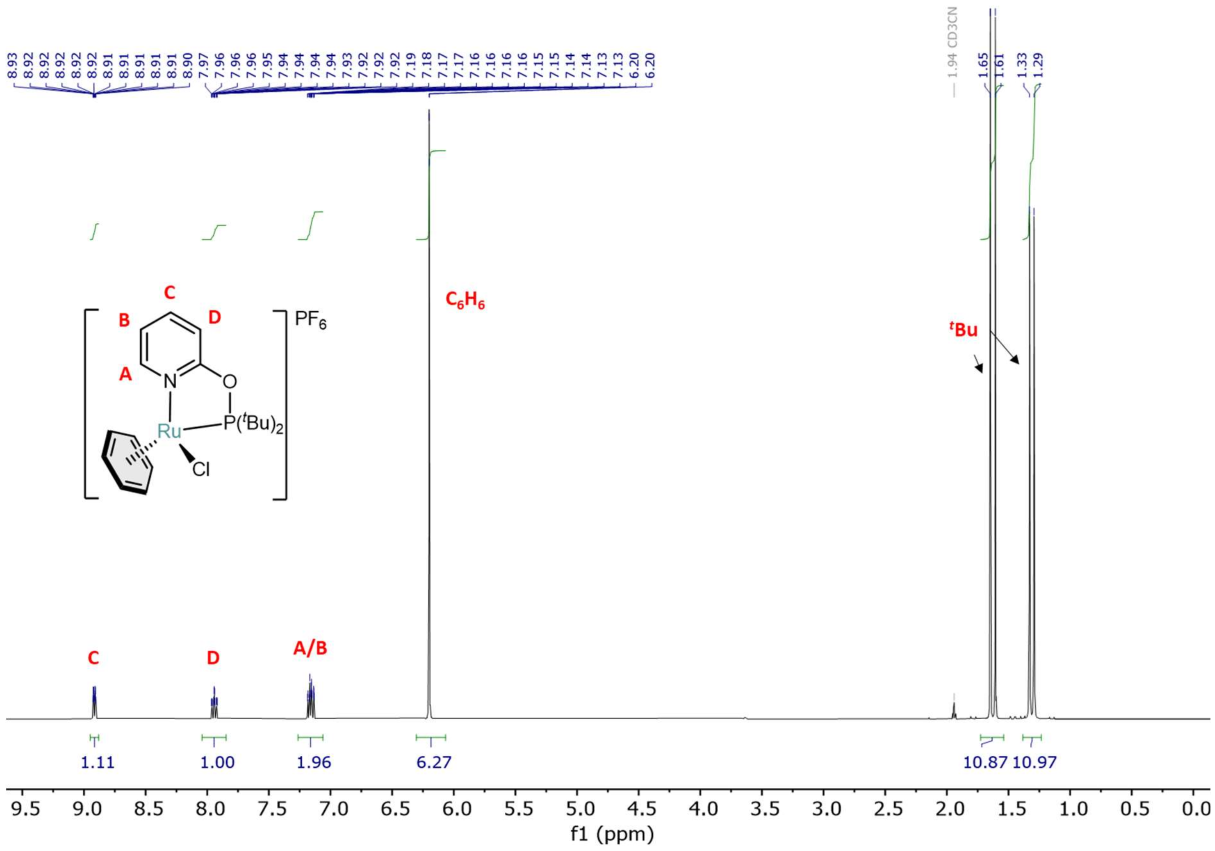

Figure S85. The ^{1}H NMR spectrum of complex **[(PON)RuCl(C_6H_6)]PF_6** in CD_3CN at 298 K.

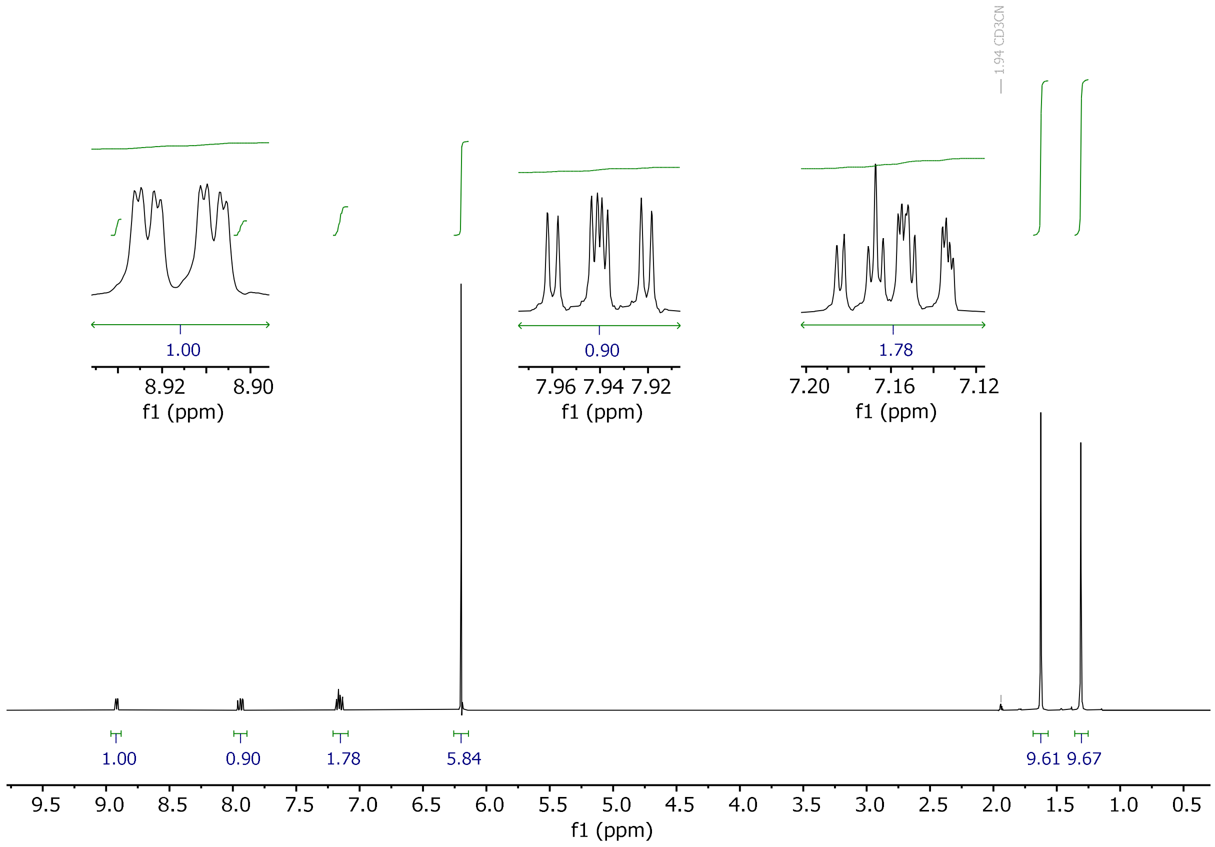

Figure S86. The $^{1}H\{^{31}P\}$ NMR spectrum of complex **[(PON)RuCl(C_6H_6)]PF_6** in CD_3CN at 298 K.

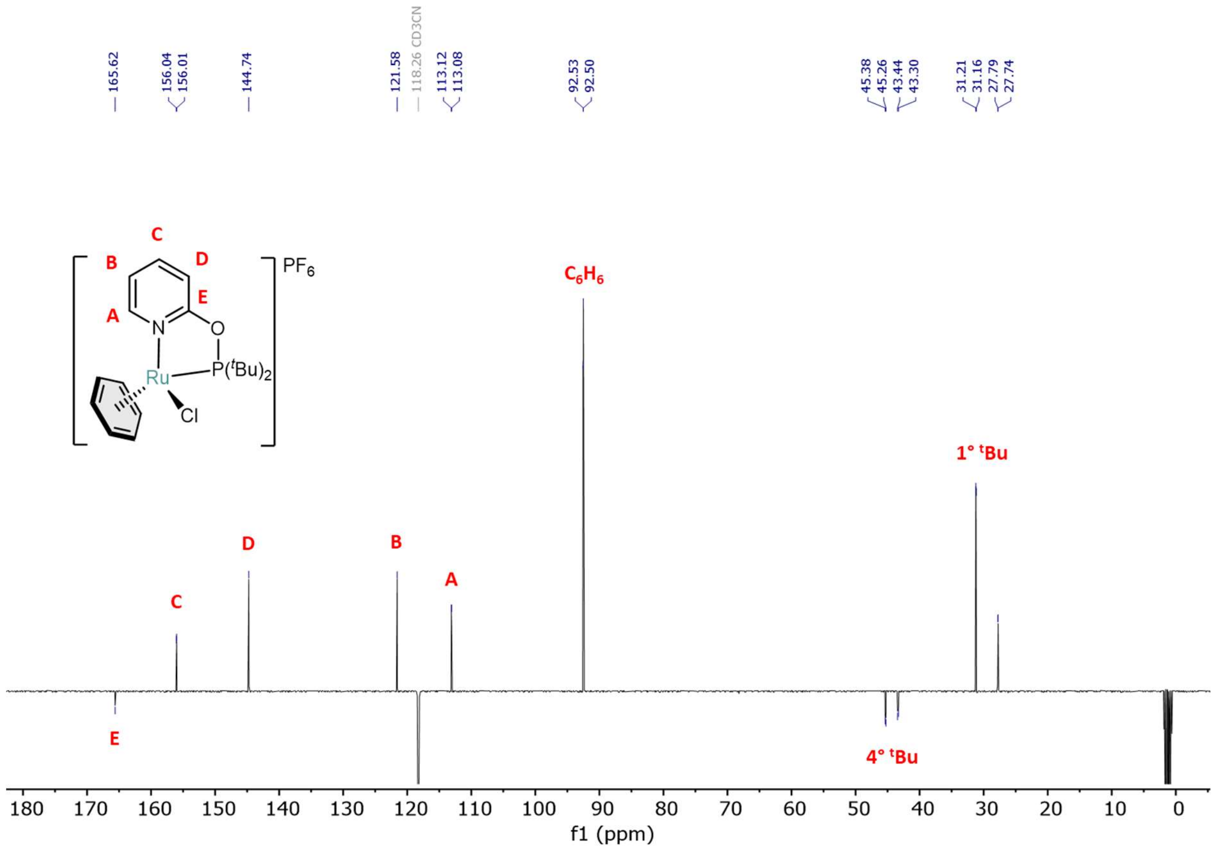

Figure S87. The ^{13}C (APT) NMR spectrum of complex **$[(PON)RuCl(C_6H_6)]PF_6$** in CD_3CN at 298 K.

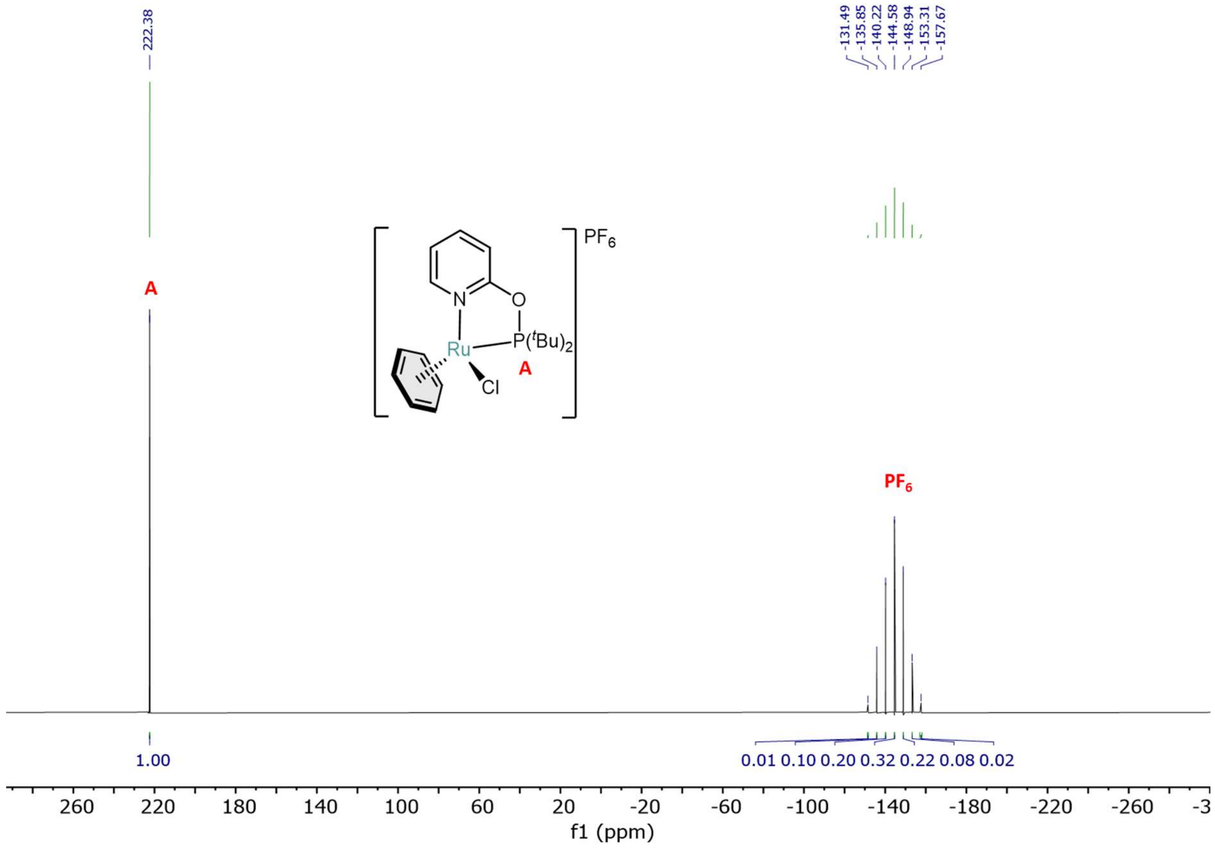

Figure S88. The $^{31}P\{^1H\}$ NMR spectrum of complex **$[(PON)RuCl(C_6H_6)]PF_6$** in CD_3CN at 298 K.

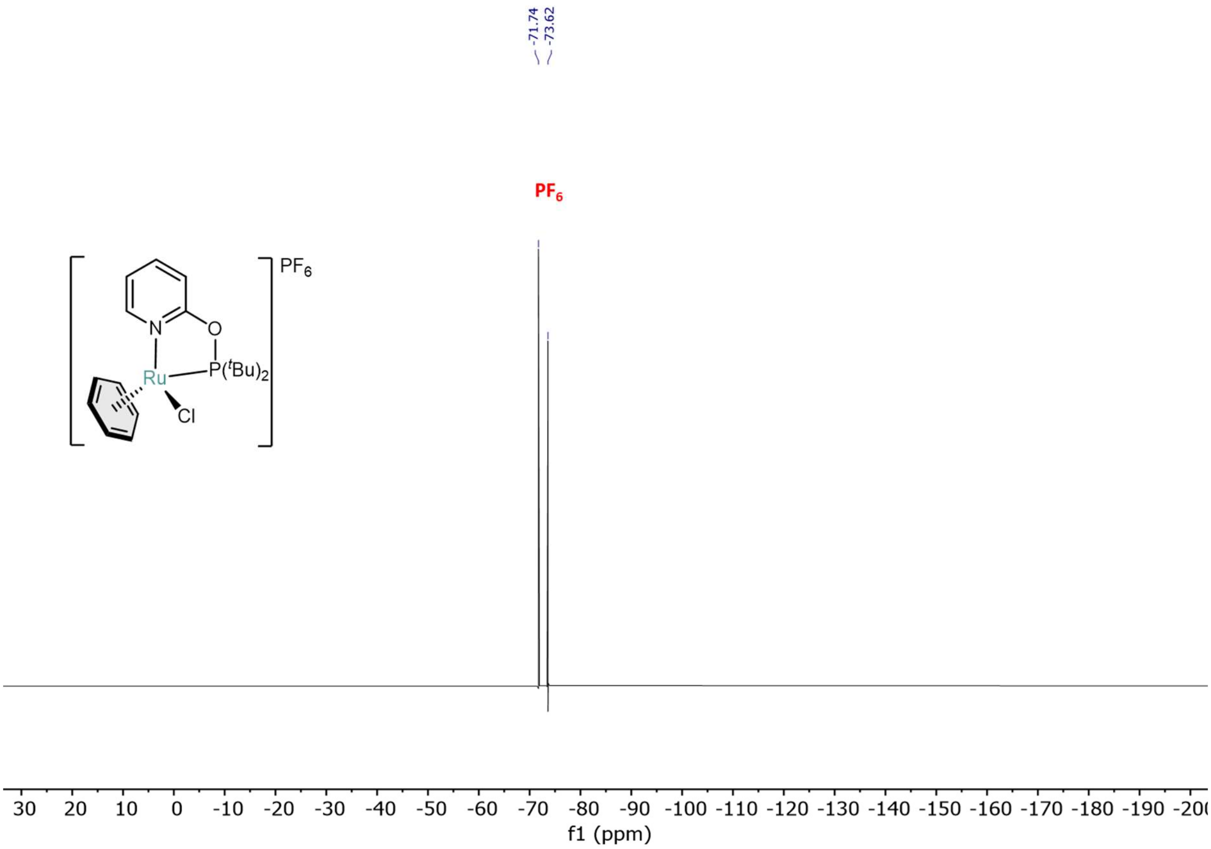

Figure S89. The $^{19}F\{^{1}H\}$ NMR spectrum of complex **[(PON)RuCl(C_6H_6)]PF_6** in CD_3CN at 298 K.

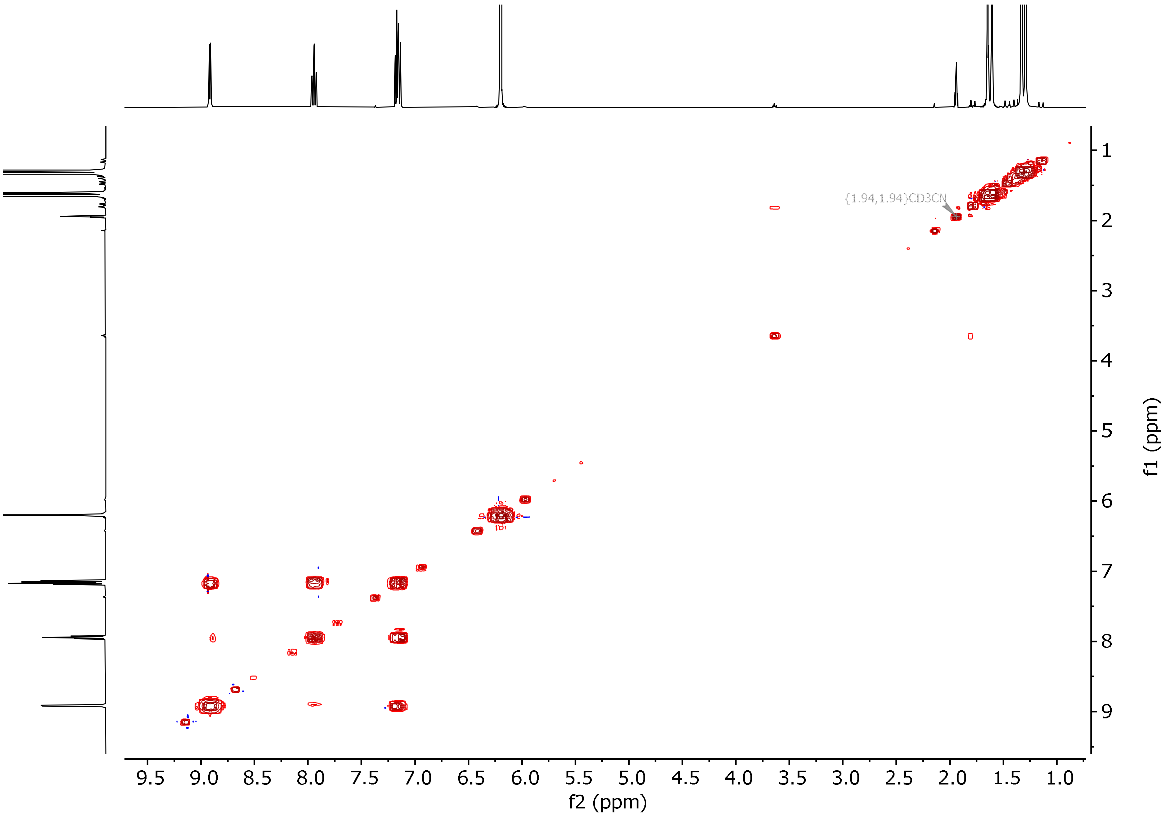

Figure S90. The ^{1}H-^{1}H COSY NMR spectrum of complex **[(PON)RuCl(C_6H_6)]PF_6** in CD_3CN at 298 K.

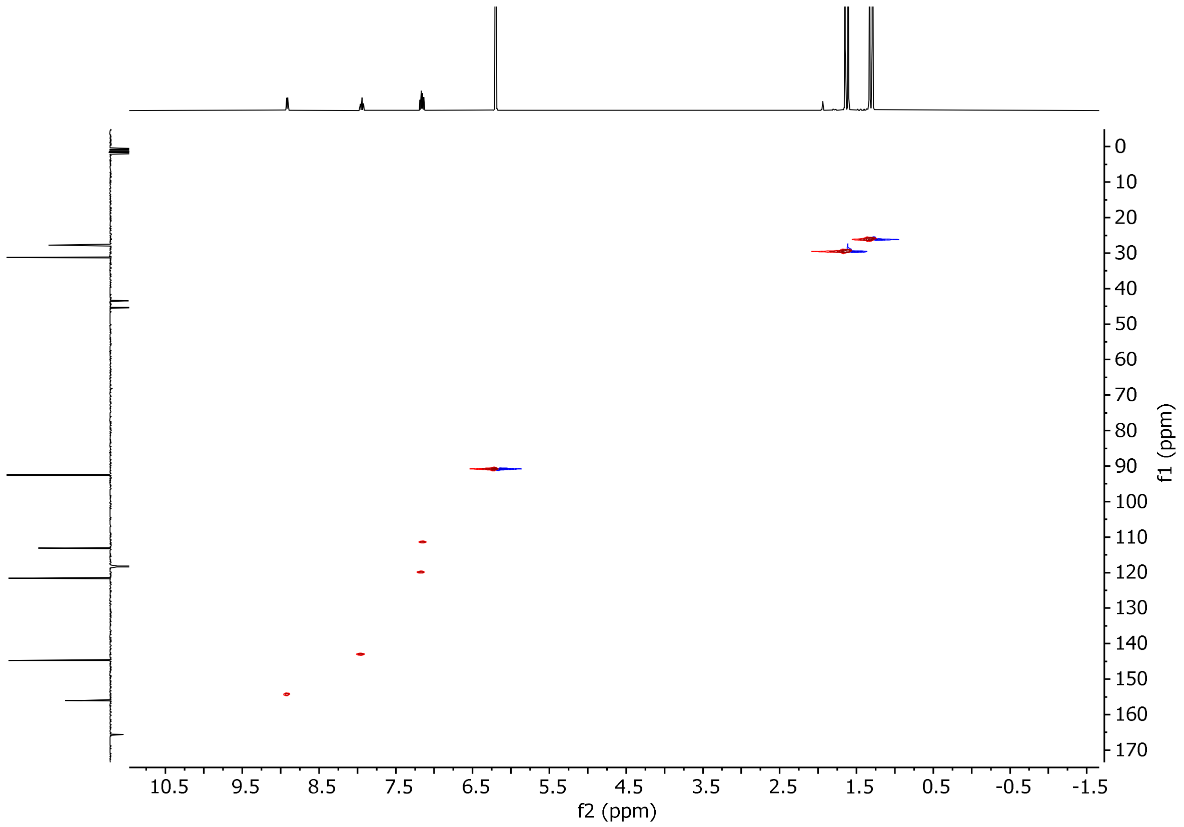

Figure S91. The ^{1}H-^{13}C ASAP-HMQC NMR spectrum of complex **[(PON)RuCl(C_6H_6)]PF_6** in CD_3CN at 298 K.

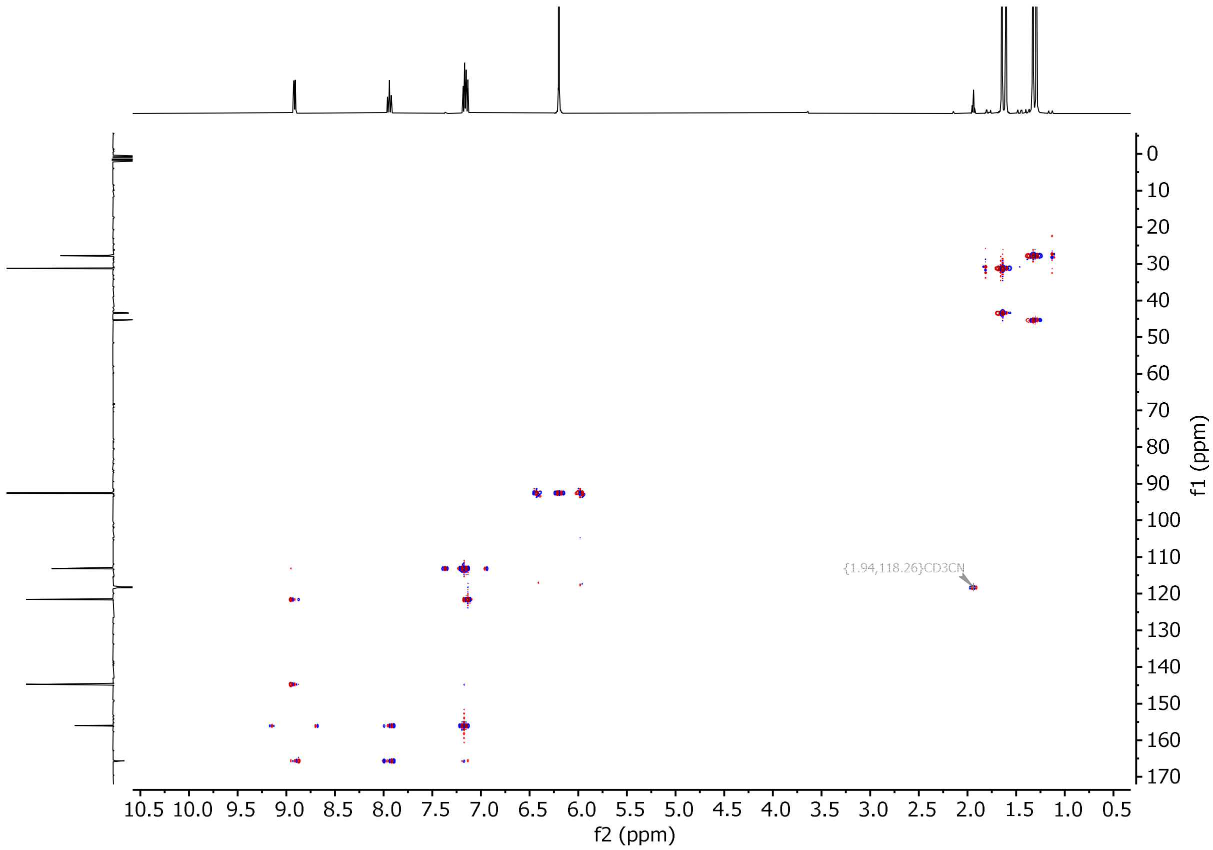

Figure S92. The ^{1}H-^{13}C HMBC NMR spectrum of complex **[(PON)RuCl(C_6H_6)]PF_6** in CD_3CN at 298 K.

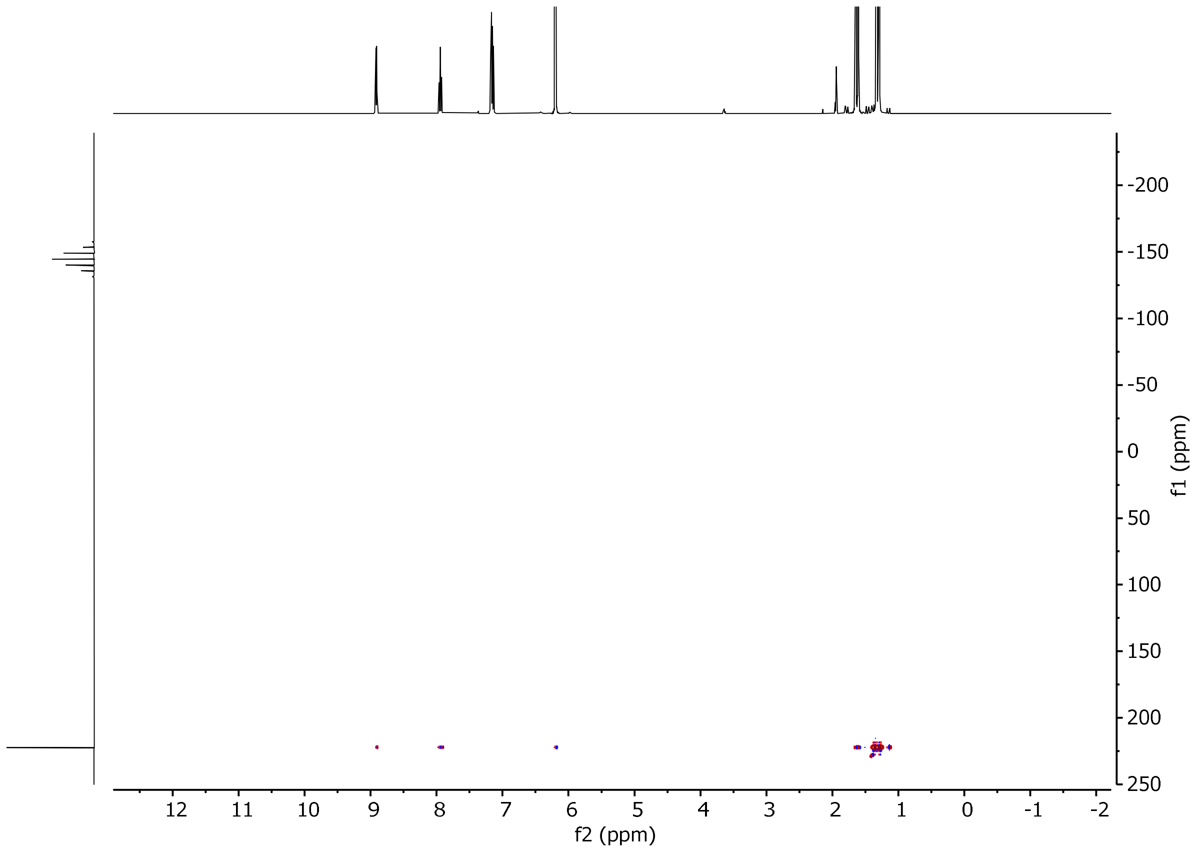

Figure S93. The ^{1}H-^{31}P HMBC NMR spectrum of complex **[(PON)RuCl(C_6H_6)]PF_6** in CD_3CN at 298 K.

[(NN)RuCl(C_6H_6)]PF_6

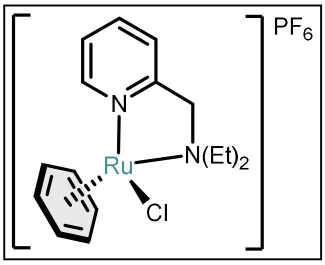

A round-bottom flask was charged with [Ru(C_6H_6)Cl_2]$_2$ (304.4 mg, 0.61 mmol), MeCN (20.0 mL), and the NN ligand (200.2 mg, 1.22 mmol) under air. The resulting brown suspension was stirred at room temperature for 1h and the volatiles were removed *in vacuo* to obtain a dark brown solid. A suspension of NH_4PF_6 (401.5 mg, 2.46 mmol) in EtOH (10.0 mL) was added to the brown solid and the resulting brownish yellow suspension was stirred for 30 min. 10.0 mL of H_2O was added to the reaction mixture, which was subsequently filtered over a paper filter. the residue was washed with 5.0 mL H_2O and 20.0 mL Et_2O, and was dried under a dynamic vacuum to obtain 516.0 mg of a brownish green powder (80.9%).

^{1}H NMR (400 MHz, CD_3CN, 298K): δ = 9.23 (ddd, $J_{H,H}$ = 5.7, 1.4, 0.8 Hz, 1H), 7.93 (td, $J_{H,H}$ = 7.7, 1.5 Hz, 1H), 7.46 (ddt, $J_{H,H}$ = 7.3, 6.0, 1.2 Hz, 1H), 7.38 (d, $J_{H,H}$ = 7.9 Hz, 1H), 5.91 (s, 6H), 4.07 (d, $^2J_{H,H}$ = 16.0 Hz, 1H), 3.79 – 3.37 (m, 5H), 1.24 (t, $^3J_{H,H}$ = 7.2 Hz, 3H), 0.93 (t, $^3J_{H,H}$ = 7.2 Hz, 3H).

^{13}C{^{1}H} NMR (100 MHz, CD_3CN, 298K): δ = 163.0 (s), 155.0 (s), 141.2 (s), 125.7 (s), 122.9 (s), 87.0 (s), 62.5 (s), 59.7 (s), 59.4 (s), 12.8 (s), 10.5 (s).

^{31}P{^{1}H} NMR (162 MHz, CD_3CN, 298K): δ = –144.0 (hept, $^1J_{P,F}$ = 706.5 Hz, 1P).

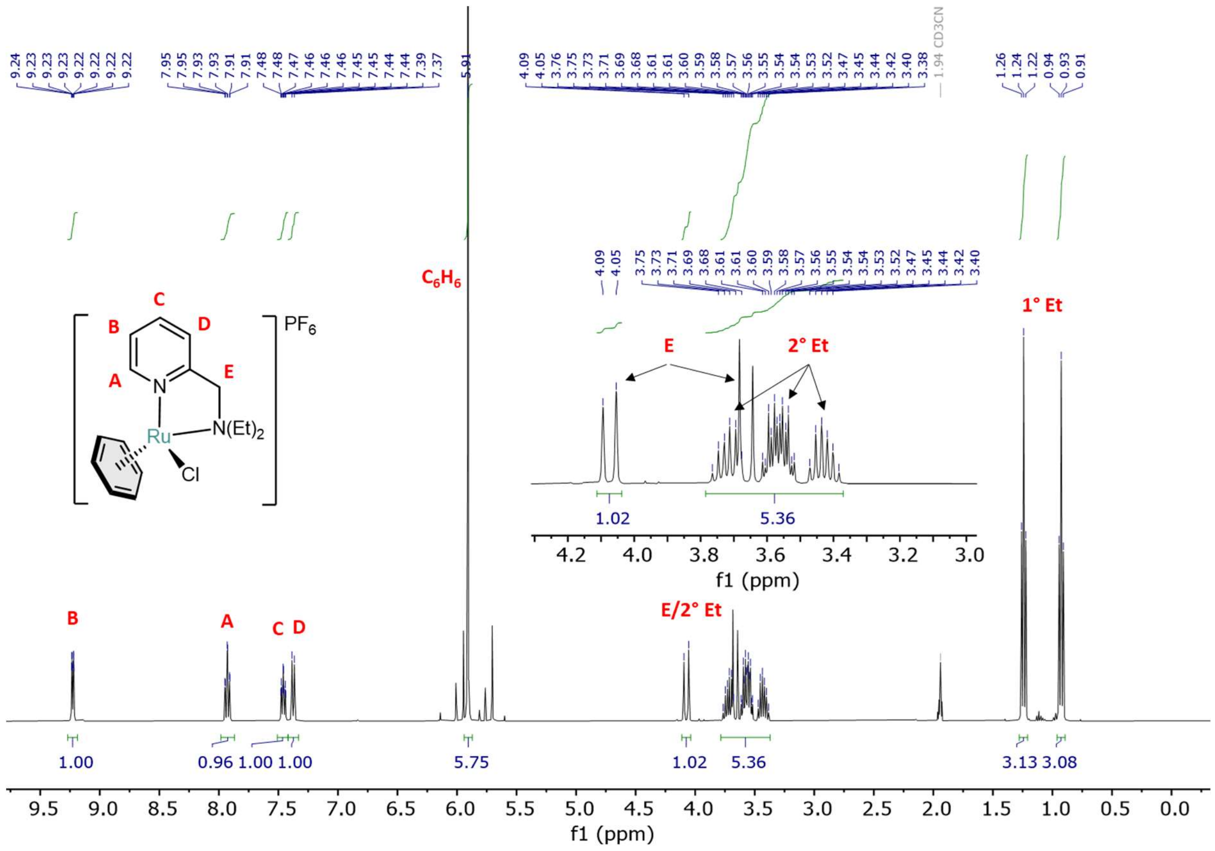

Figure S94. The ^{1}H NMR spectrum of complex **[(NN)RuCl(C_6H_6)]PF_6** in CD_3CN at 298 K.

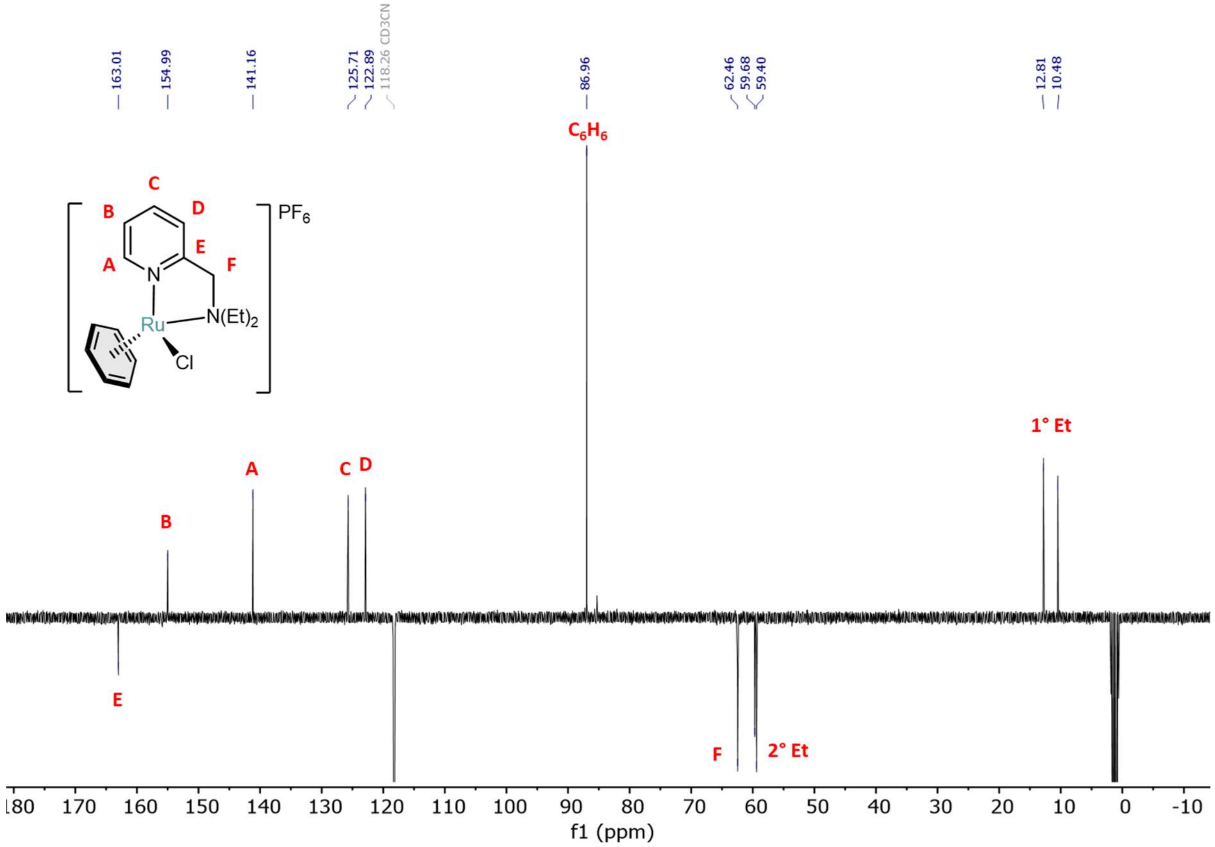

Figure S95. The ^{13}C (APT) NMR spectrum of complex **[(NN)RuCl(C_6H_6)]PF_6** in CD_3CN at 298 K.

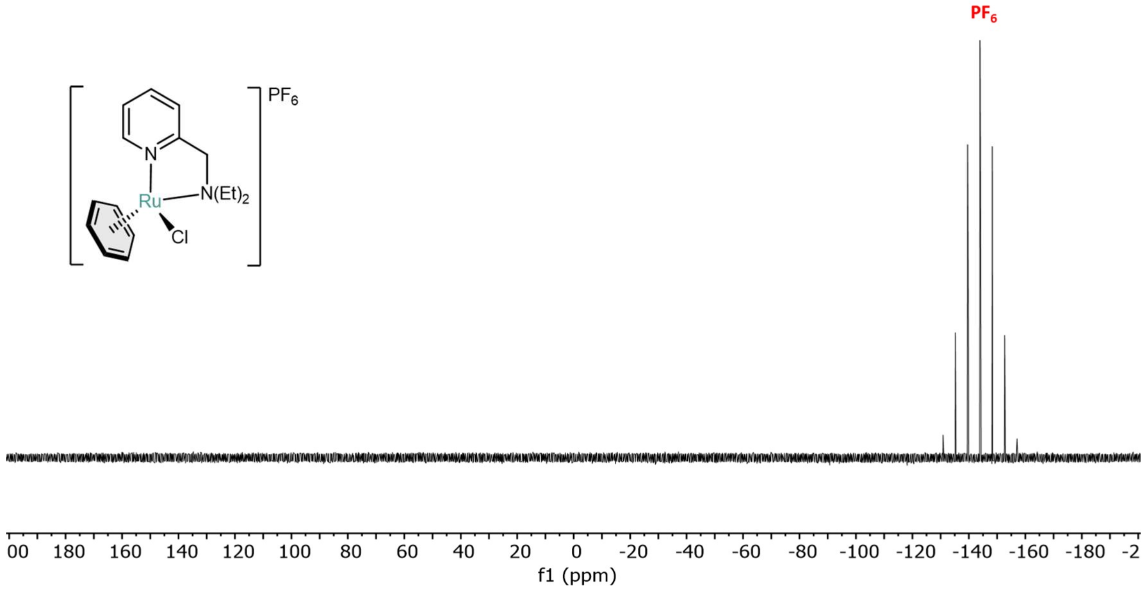

Figure S96. The $^{31}P\{^{1}H\}$ NMR spectrum of complex **[(NN)RuCl(C_6H_6)]PF_6** in CD_3CN at 298 K.

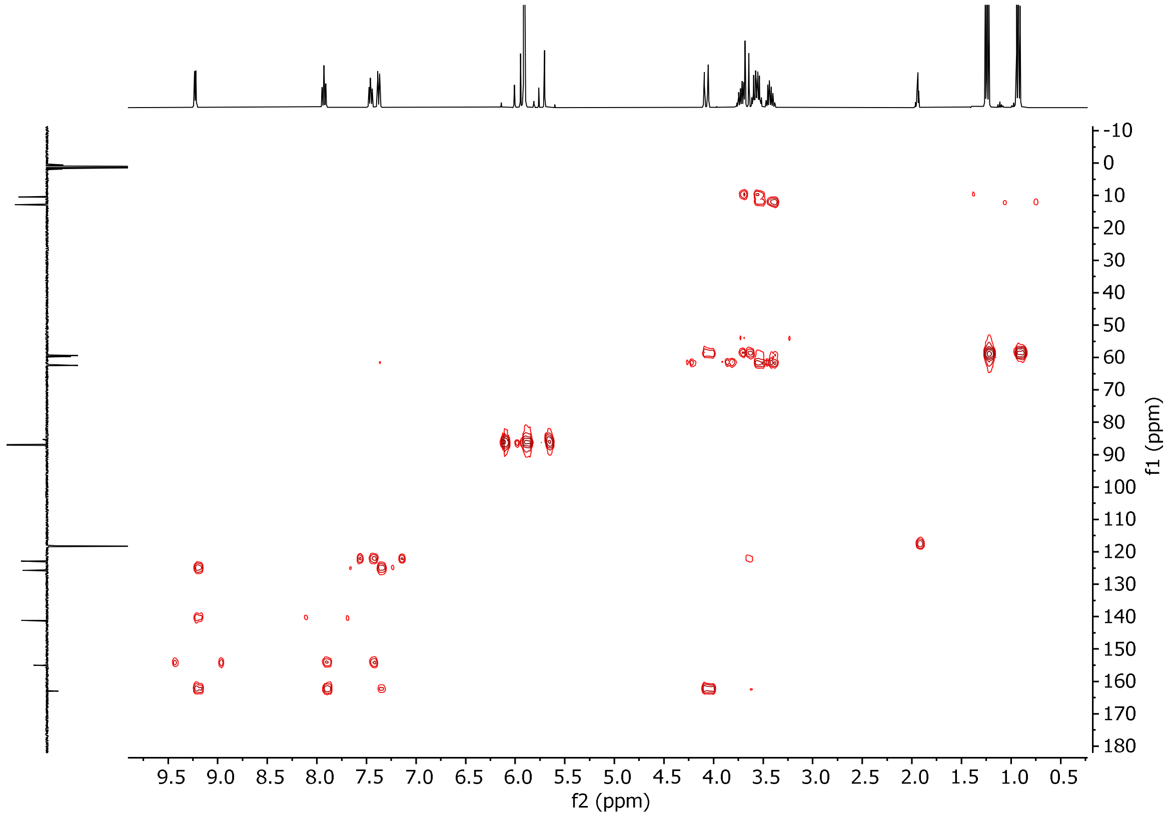

Figure S97. The ^{1}H-^{13}C ASAP-HMQC NMR spectrum of complex **[(NN)RuCl(C_6H_6)]PF_6** in CD_3CN at 298 K.

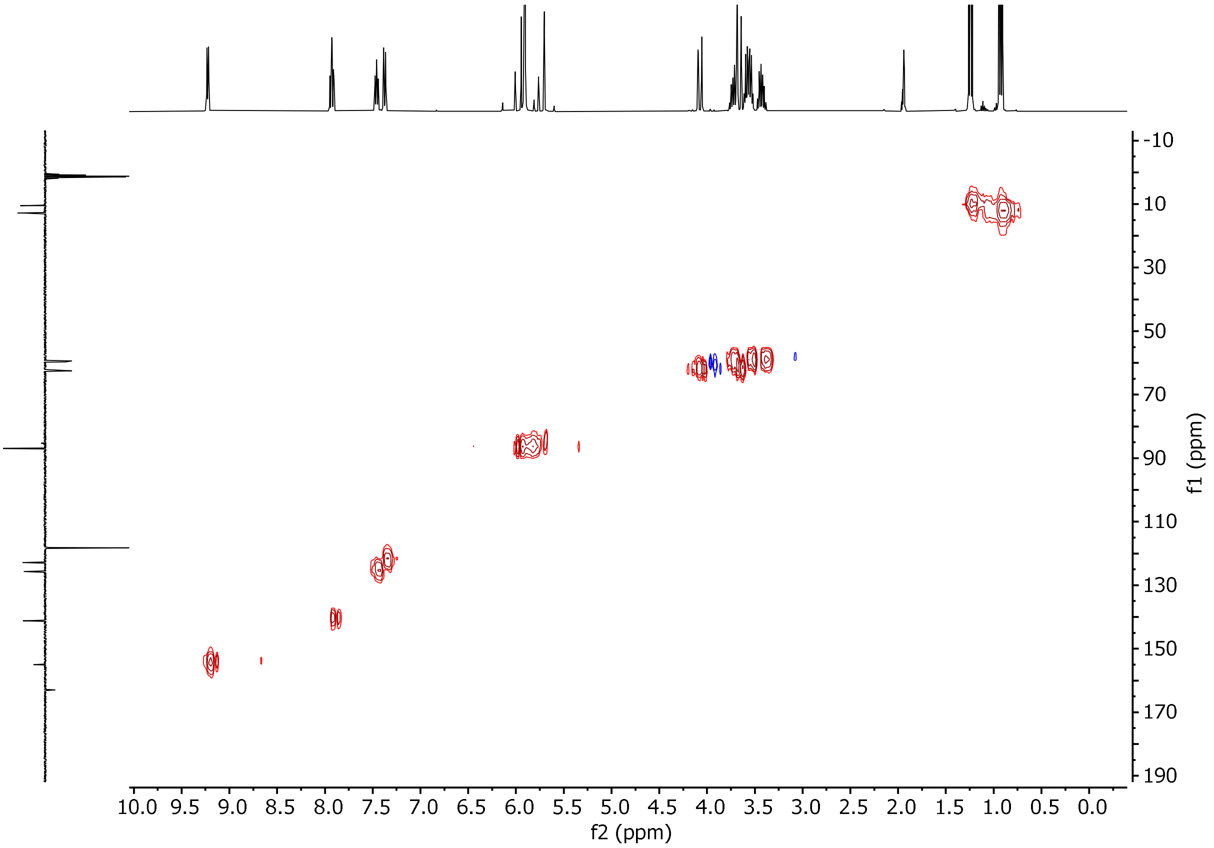

Figure S98. The ^{1}H-^{13}C HMBC NMR spectrum of complex **[(NN)RuCl(C_6H_6)]PF_6** in CD_3CN at 298 K.

[(^{t}Bu-bpy)RuCl(C_6H_6)]PF_6

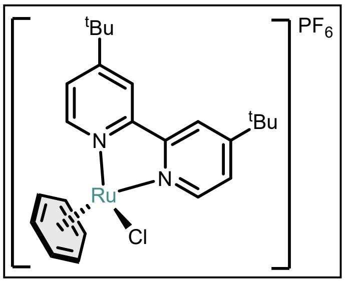

A round-bottom flask was charged with [Ru(C_6H_6)Cl_2]$_2$ (250.0 mg, 0.50 mmol), DCM (20.0 mL), and the ^{tBu}Bipy ligand (268.2 mg, 1.00 mmol) under air. The resulting brown suspension was stirred for 1h at room temperature, yielding a brown solution. A solution of NH_4PF_6 (251.8 mg, 1.55 mmol) in EtOH (20.0 mL) was added to the brown reaction mixture, which was stirred for another 20 min. *n*-hexane (200 mL) was added to the reaction mixture and the resulting yellow-blackish suspension was filtered over a paper filter. The residue was extracted with DCM (30.0 mL)to obtain an orange/yellow filtrate. Volatiles were removed under a dynamic vacuum giving 568.0 mg of an orange/yellow solid (90.4%).

^{1}H NMR (400 MHz, CD_3CN, 298K): δ = 9.28 (dd, $J_{H,H}$ = 6.1, 0.6 Hz, 2H), 8.30 (dd, $J_{H,H}$ = 2.2, 0.6 Hz, 2H), 7.68 (dd, $J_{H,H}$ = 6.1, 2.1 Hz, 2H), 5.98 (s, 6H), 1.44 (s, 18H).

^{13}C{^{1}H} NMR (100 MHz, CD_3CN, 298K): δ = 165.8 (s), 156.1 (s), 155.7 (s), 125.5 (s), 122.0 (s), 87.9 (s), 36.5 (s), 30.4 (s).

^{31}P{^{1}H} NMR (162 MHz, CD_3CN, 298K): δ = –144.0 (hept, $^1J_{P,F}$ = 707.3 Hz, 1P).

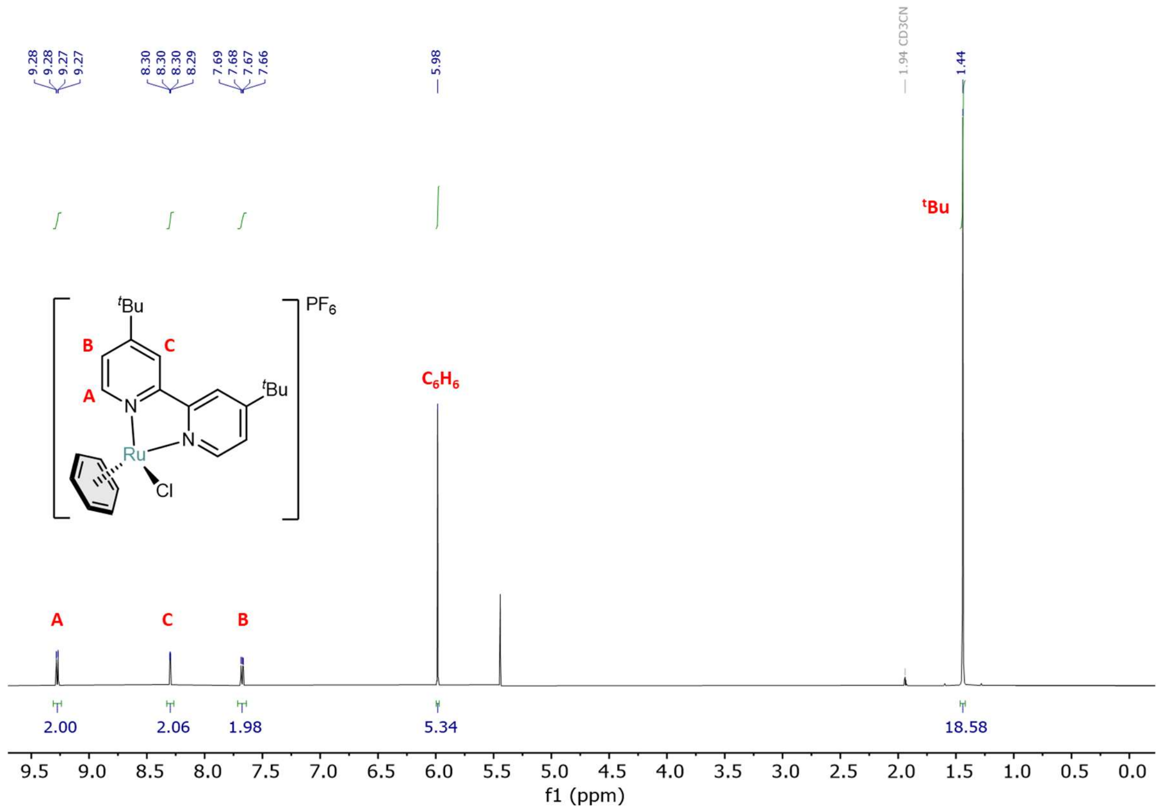

Figure S99. The ^{1}H NMR spectrum of complex **[(ᵗBu-bpy)RuCl(C_6H_6)]PF_6** in CD_3CN at 298 K.

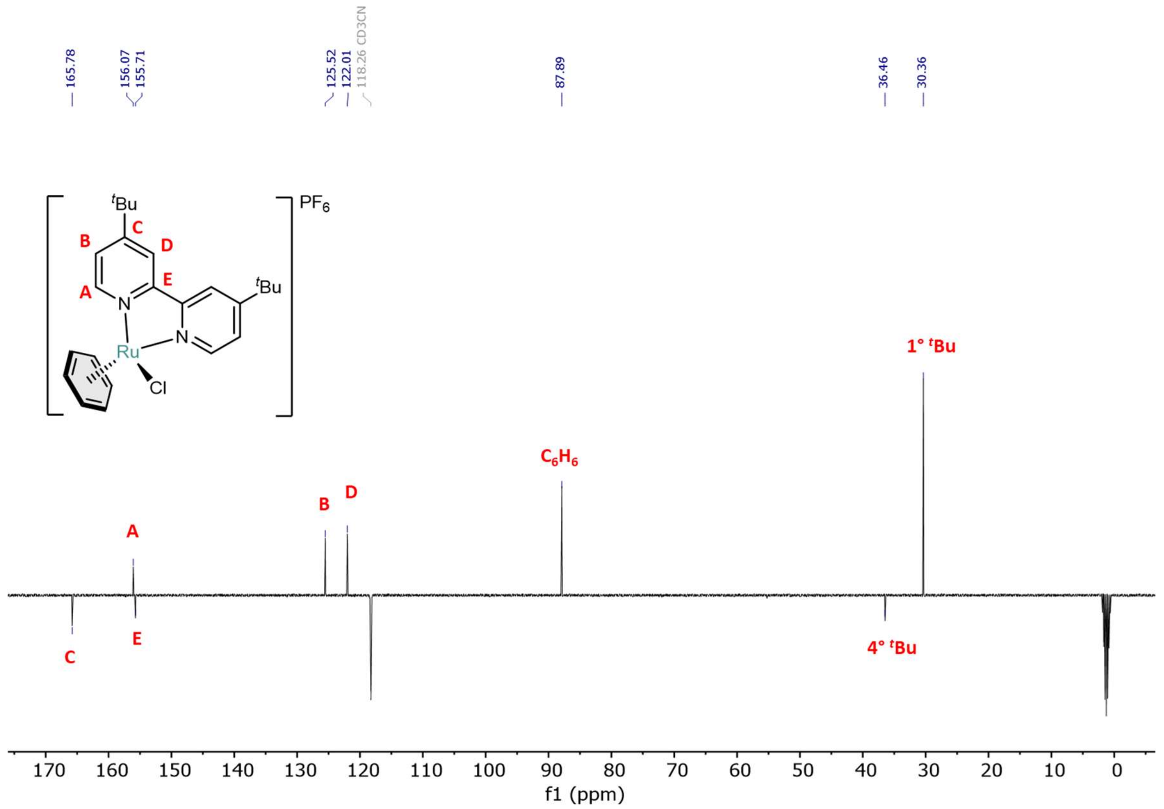

Figure S100. The ^{13}C (APT) NMR spectrum of complex **[(ᵗBu-bpy)RuCl(C_6H_6)]PF_6** in CD_3CN at 298 K.

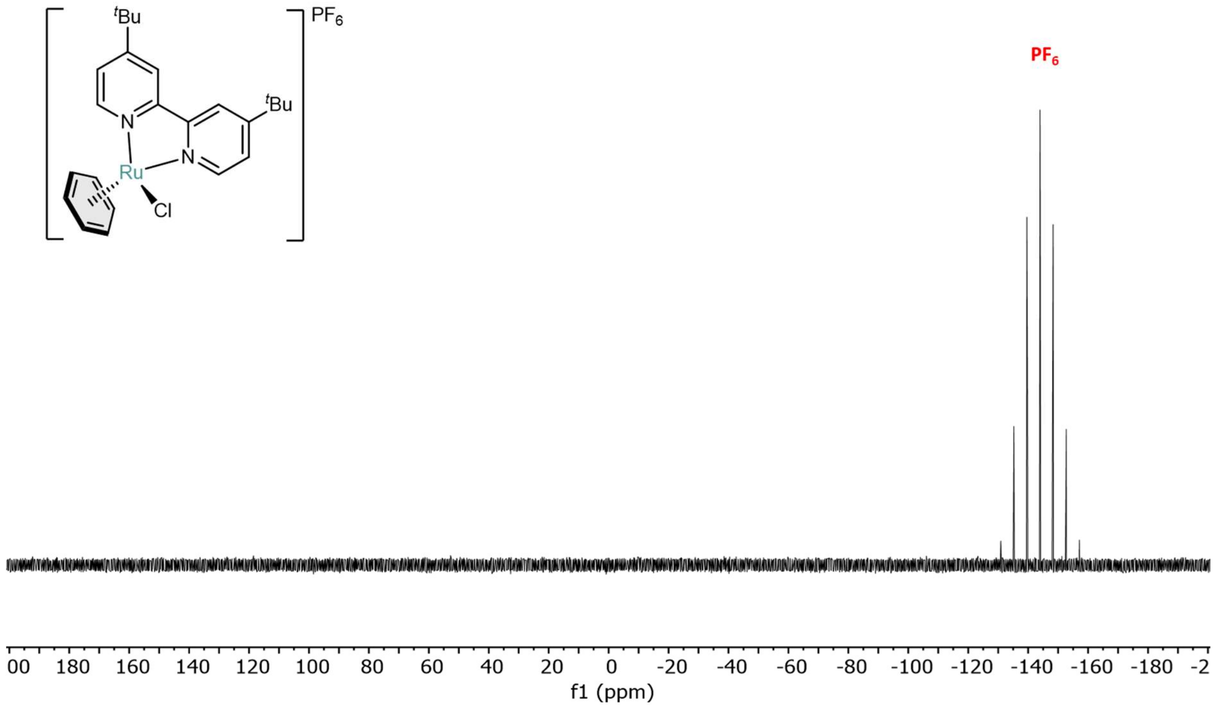

Figure S101. The $^{31}P\{^{1}H\}$ NMR spectrum of complex **[(^{t}Bu-bpy)RuCl(C_6H_6)]PF_6** in CD_3CN at 298 K.

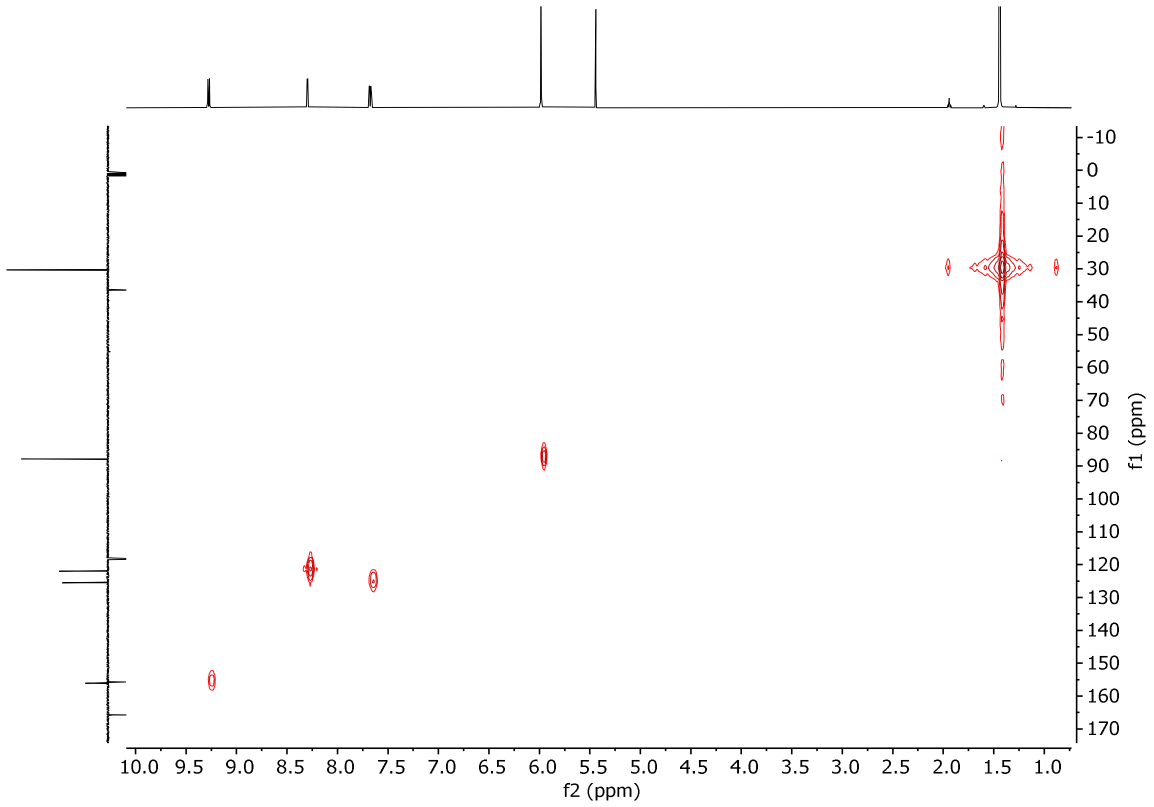

Figure S102. The ^{1}H-^{13}C ASAP-HMQC NMR spectrum of complex **[(^{t}Bu-bpy)RuCl(C_6H_6)]PF_6** in CD_3CN at 298 K.

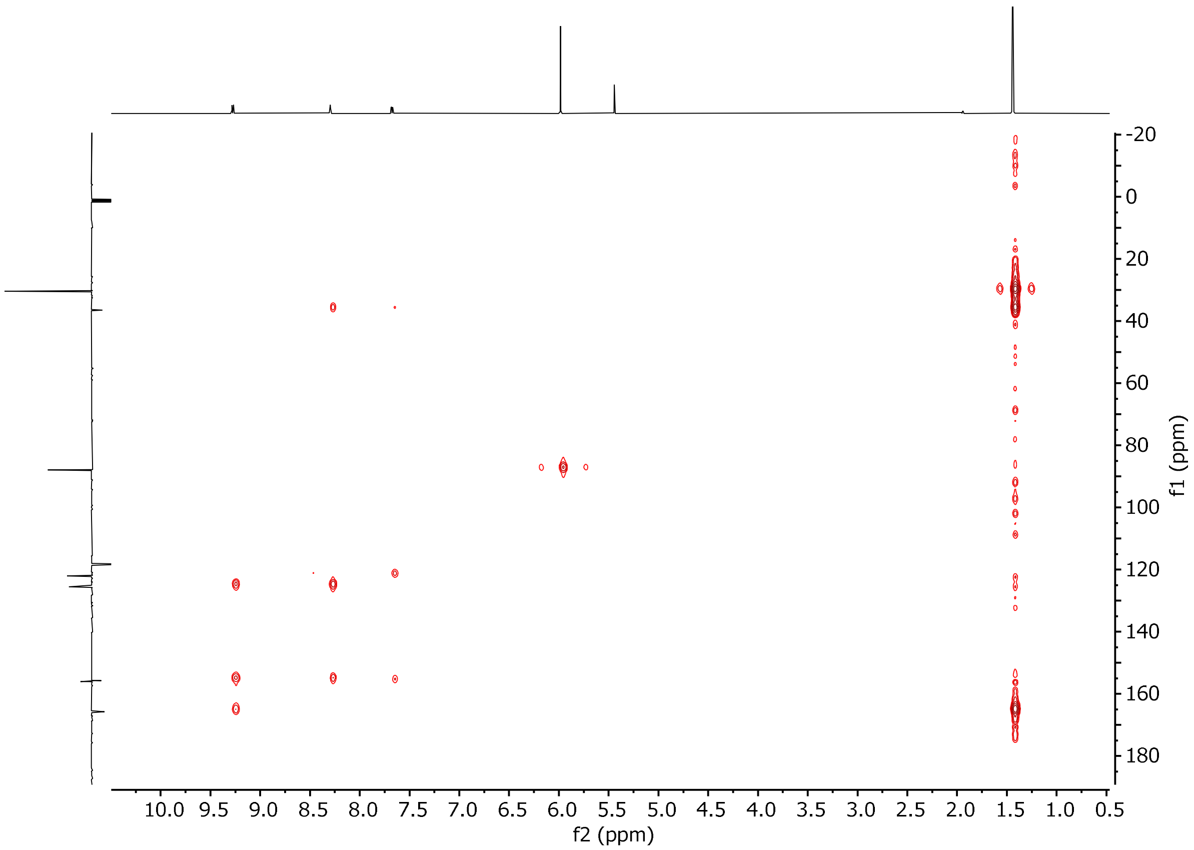

Figure S103. The ^{1}H-^{13}C HMBC NMR spectrum of complex **[(^{t}Bu-bpy)RuCl(C_6H_6)]PF_6** in CD_3CN at 298 K.

[(phen)RuCl(C_6H_6)]PF_6

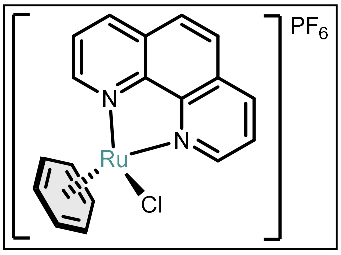

A round-bottom flask was charged with [Ru(C_6H_6)Cl_2]$_2$ (25 mg, 0.05 mmol), DCM (15.0 mL), and the phen ligand (18.0 mg, 0.10 mmol) under air. The resulting brown suspension was stirred for 3 h at room temperature, leading to a greenish yellow suspension. A solution of NH_4PF_6 (20.0 mg, 0.122 mmol) in 6.0 mL MeOH was added to the reaction mixture, that changed the color to yellow upon addition. The reaction mixture was filtered over a paper filter, yielding a yellow filtrate. Next, *n*-hexane (50.0 mL) was added to form a yellow suspension, which was then filtered over a paper filter. The obtained yellow residue was extracted with 30.0 mL MeCN and dried under dynamic vacuum to give 34.0 mg of a yellow solid (62.9%).

^{1}H NMR (400 MHz, CD_3CN, 298K): δ = 9.74 (dd, $J_{H,H}$ = 5.3, 1.3 Hz, 1H), 8.74 (ddd, $J_{H,H}$ = 8.3, 1.3, 0.5 Hz, 1H), 8.14 (d, $J_{H,H}$ = 0.6 Hz, 1H), 8.02 (ddd, $J_{H,H}$ = 8.3, 5.3, 0.4 Hz, 1H), 6.09 (s, 2H).

^{13}C{^{1}H} NMR (100 MHz, CD_3CN, 298K): δ = 156.5 (s), 146.8 (s), 139.8 (s), 131.5 (s), 128.4 (s), 127.1 (s), 87.6 (s).

^{31}P{^{1}H} NMR (162 MHz, CD_3CN, 298K): δ = –144.6 (hept, $^1J_{P,F}$ = 707.8 Hz, 1P).

^{19}F NMR (376 MHz, CD_3CN, 298K): δ = –72.9 (d, $^1J_{F,P}$ = 706.4 Hz).

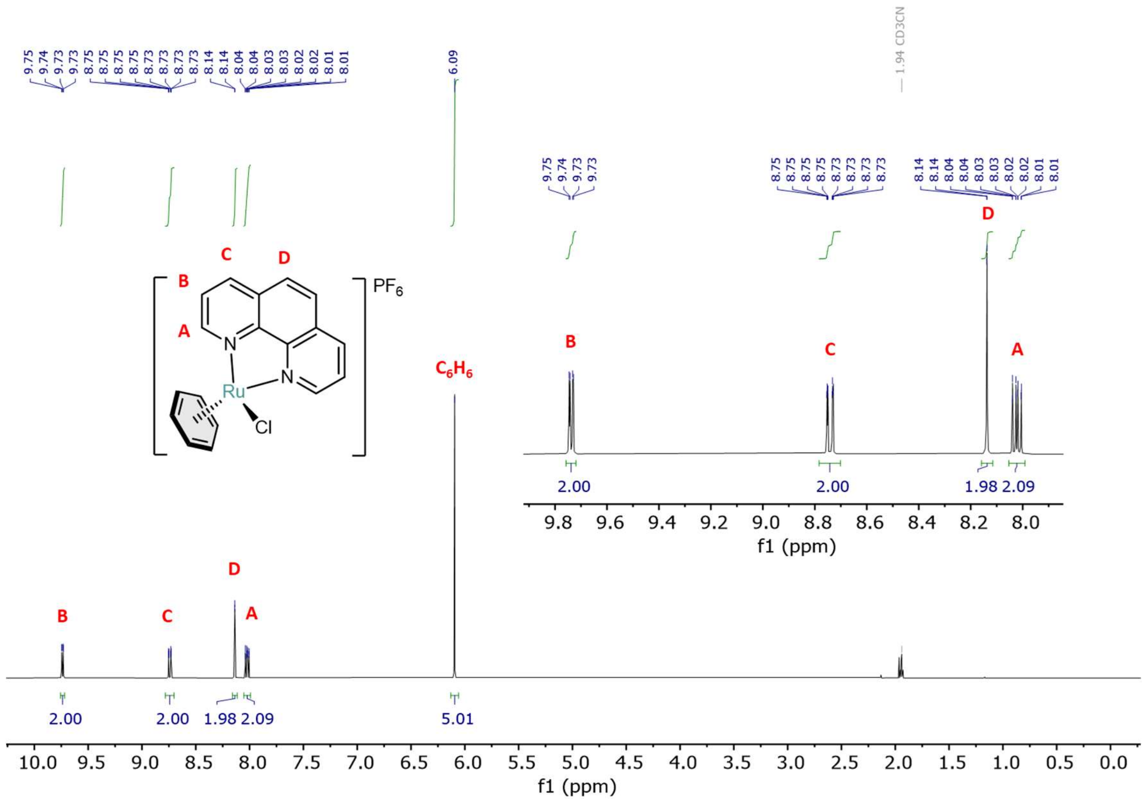

Figure S104. The ^{1}H NMR spectrum of complex **[(phen)RuCl(C_6H_6)]PF_6** in CD_3CN at 298 K.

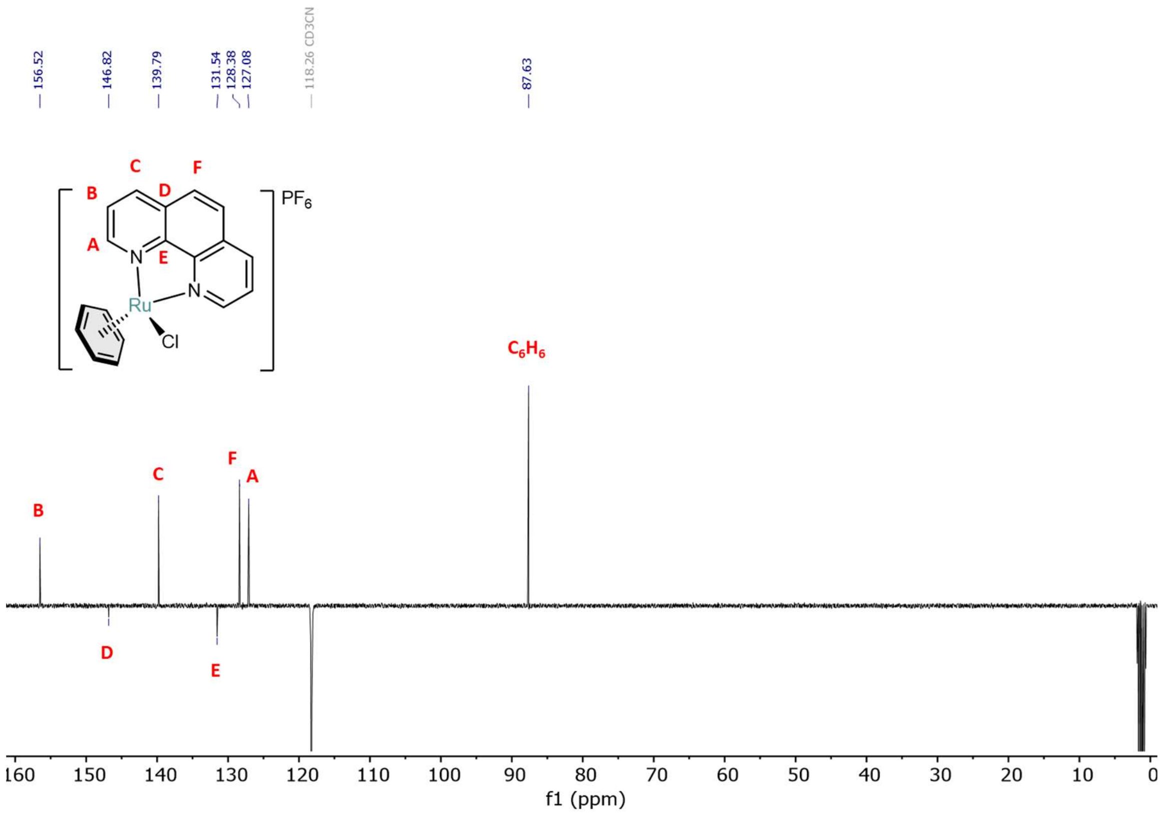

Figure S105. The ^{13}C (APT) NMR spectrum of complex **[(phen)RuCl(C_6H_6)]PF_6** in CD_3CN at 298 K.

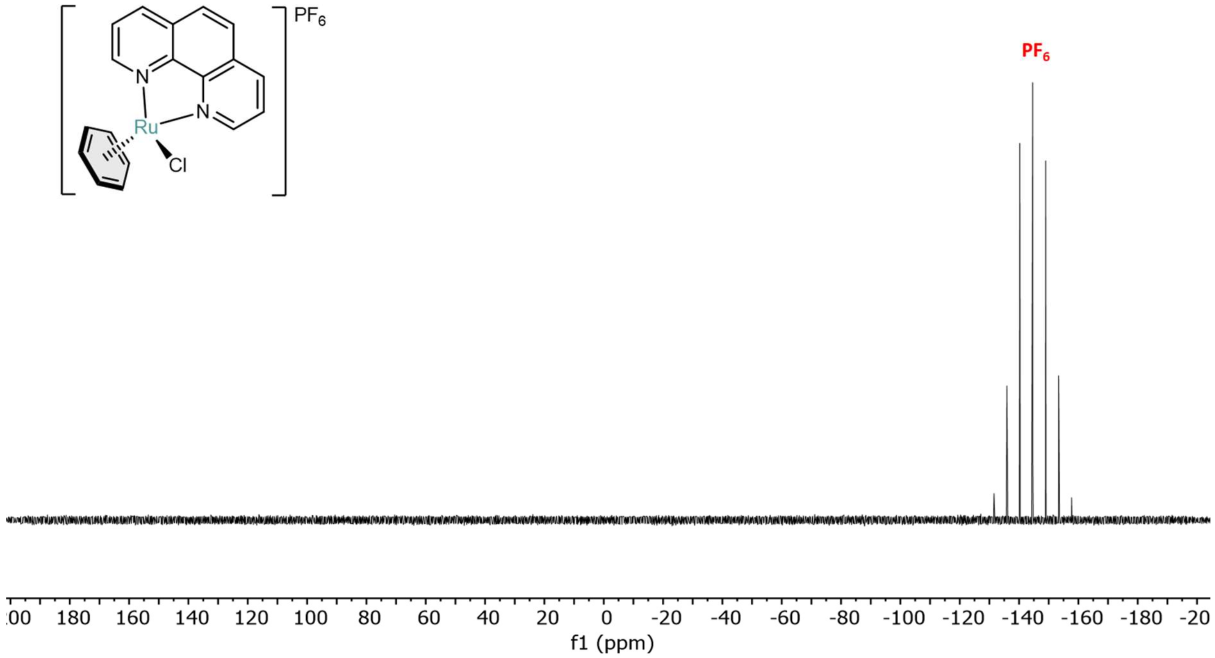

Figure S106. The $^{31}P\{^{1}H\}$ NMR spectrum of complex **[(phen)RuCl(C_6H_6)]PF_6** in CD_3CN at 298 K.

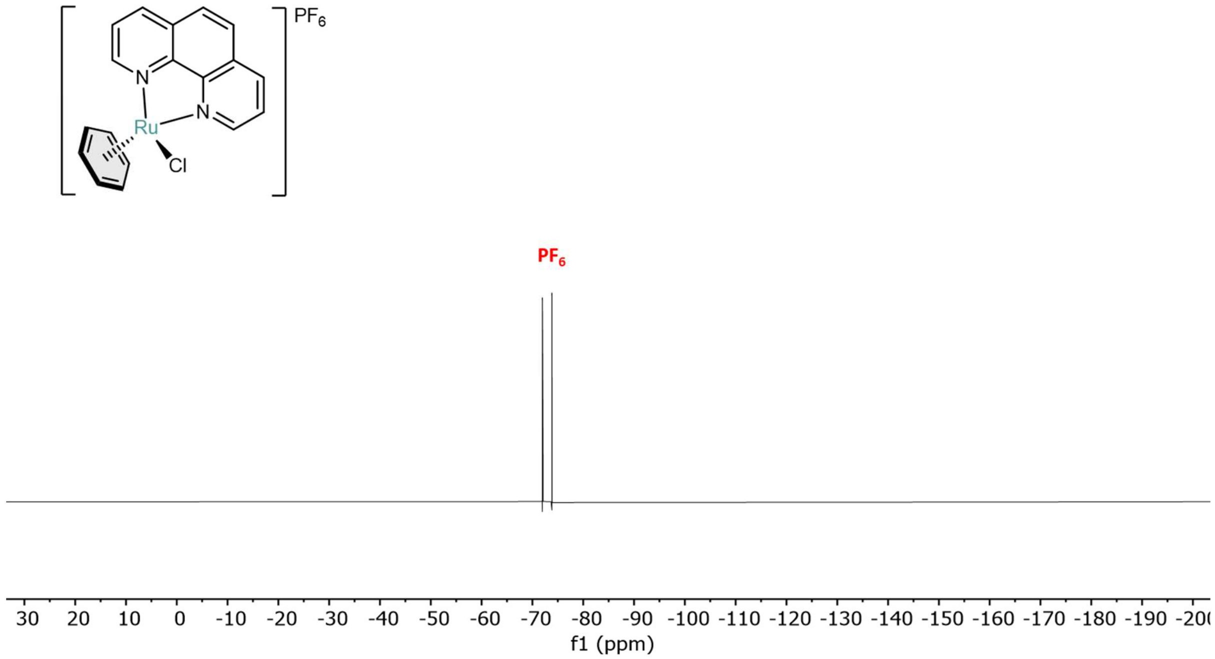

Figure S107. The $^{19}F\{^{1}H\}$ NMR spectrum of complex **[(phen)RuCl(C_6H_6)]PF_6** in CD_3CN at 298 K.

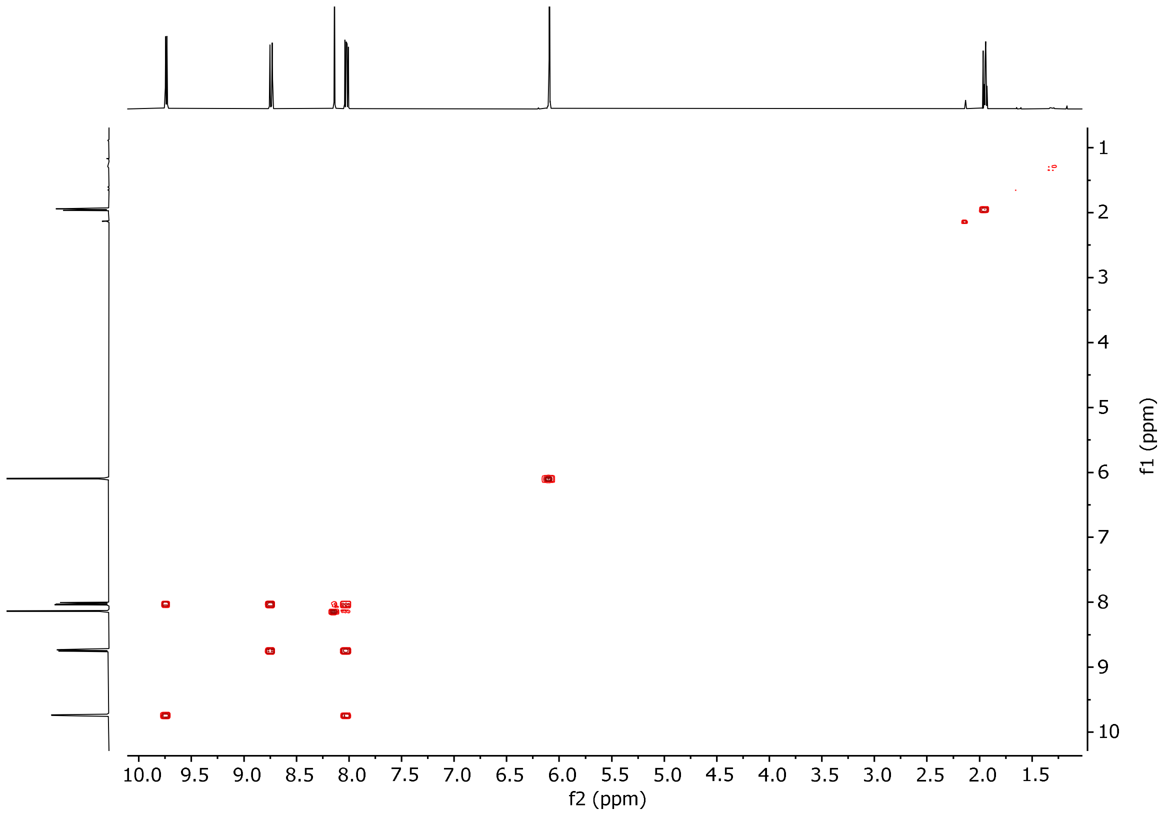

Figure S108. The ^{1}H-^{1}H COSY NMR spectrum of complex **[(phen)RuCl(C_6H_6)]PF_6** in CD_3CN at 298 K.

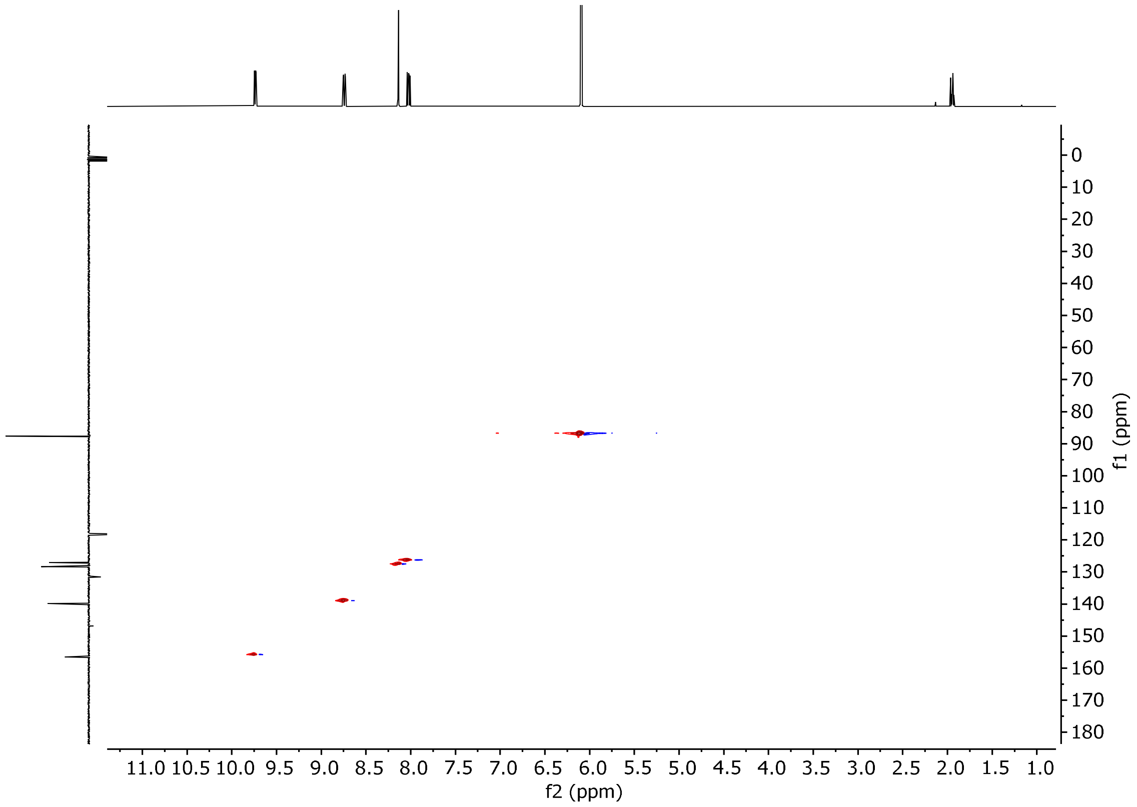

Figure S109. The ^{1}H-^{13}C ASAP-HMQC NMR spectrum of complex **[(phen)RuCl(C_6H_6)]PF_6** in CD_3CN at 298 K.

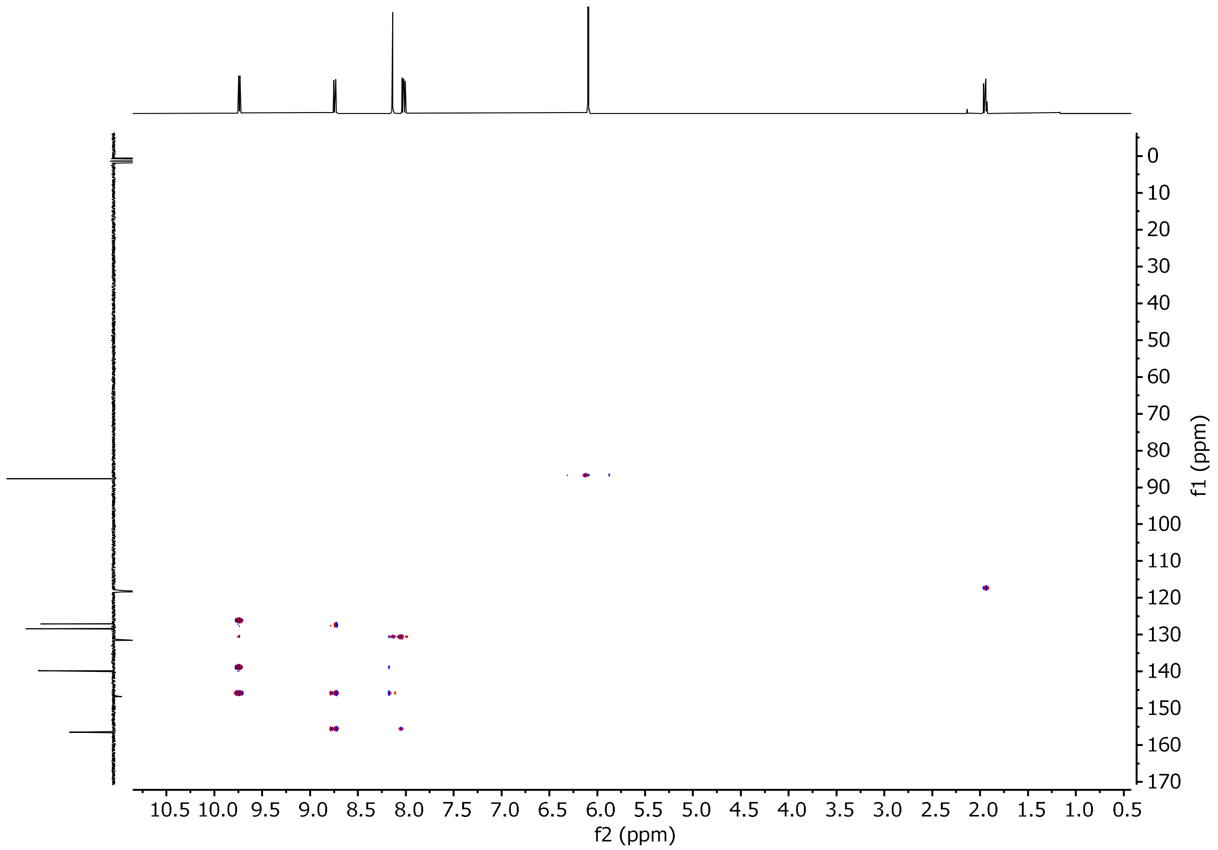

Figure S110. The ^{1}H-^{13}C HMBC NMR spectrum of complex **[(phen)RuCl(C_6H_6)]PF_6** in CD_3CN at 298 K.

[(TMEDA)RuCl(C_6H_6)]PF_6

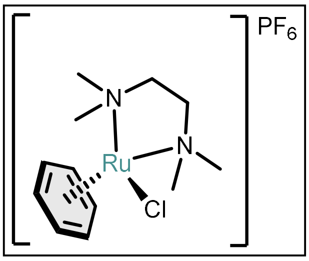

A round-bottom flask was charged with [Ru(C_6H_6)Cl_2]$_2$ (200.1 mg, 0.40 mmol), MeOH (10.0 mL) andthe TMEDA ligand (0.13 mL, 100.1 mg, 0.86 mmol) under air. The resulting brown suspension was stirred for 2 h at room temperature after which a brown yellow solution had formed. A solution of NH_4PF_6 (652.5 mg, 4.00 mmol) in MeOH (3.0 mL) was added to the reaction mixture. After stirring for 15 min, the reaction mixture was filtered over a paper filter. The residue was washed with a 10.0 mL MeOH and 20.0 mL *n*-hexane and was dried under dynamic vacuum to obtain 323.0 mg of a bright yellow powder (84.9%).

^{1}H NMR (400 MHz, CD_3CN, 298K): δ = 5.77 (s, 6H), 3.34 (s, 6H), 2.81 (s, 6H), 2.50 – 2.34 (m, 4H).

^{13}C{^{1}H} NMR (100 MHz, CD_3CN, 298K): δ = 85.8 (s), 62.4 (s), 60.1 (s), 55.7 (s).

^{31}P{^{1}H} NMR (162 MHz, CD_3CN, 298K): δ = –144.0 (hept, $^1J_{P,F}$ = 707.2 Hz, 1P).

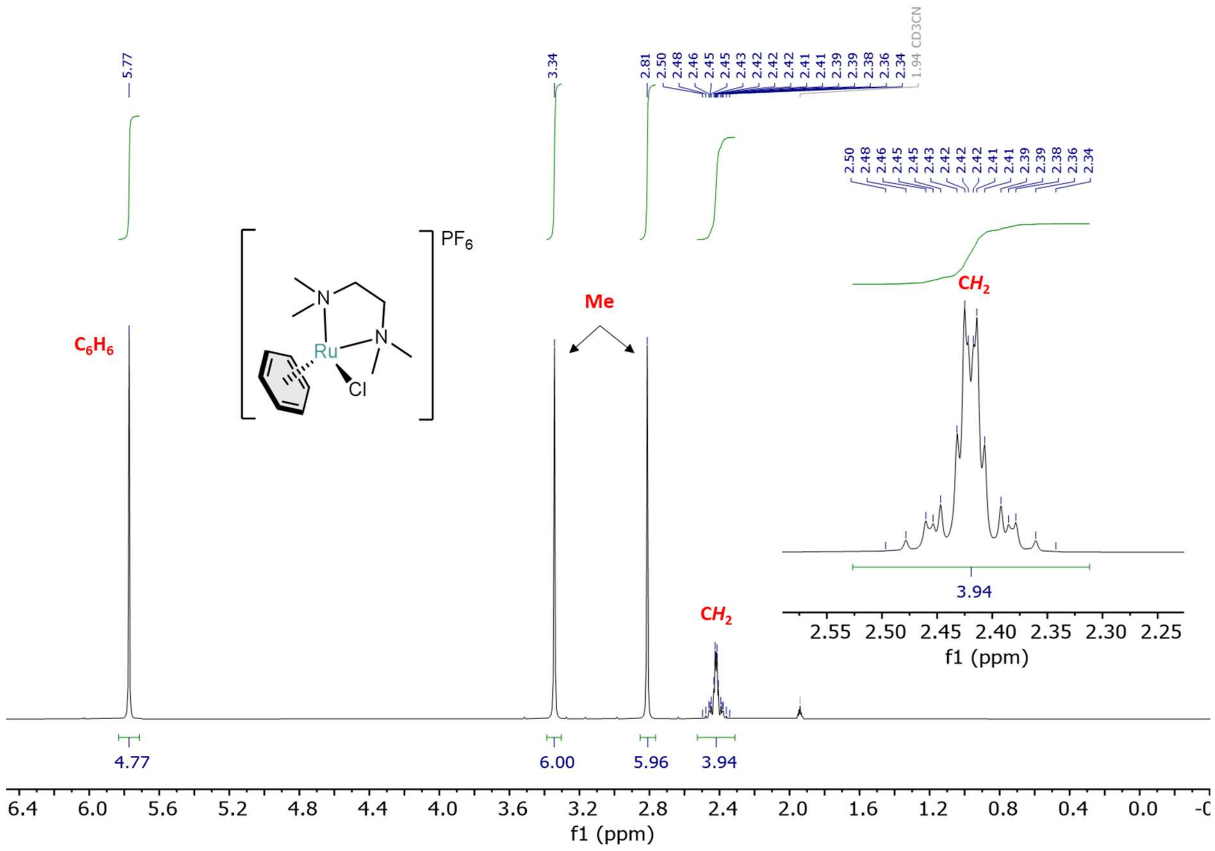

Figure S111. The ^{1}H NMR spectrum of complex **[(TMEDA)RuCl(C_6H_6)]PF_6** in CD_3CN at 298 K.

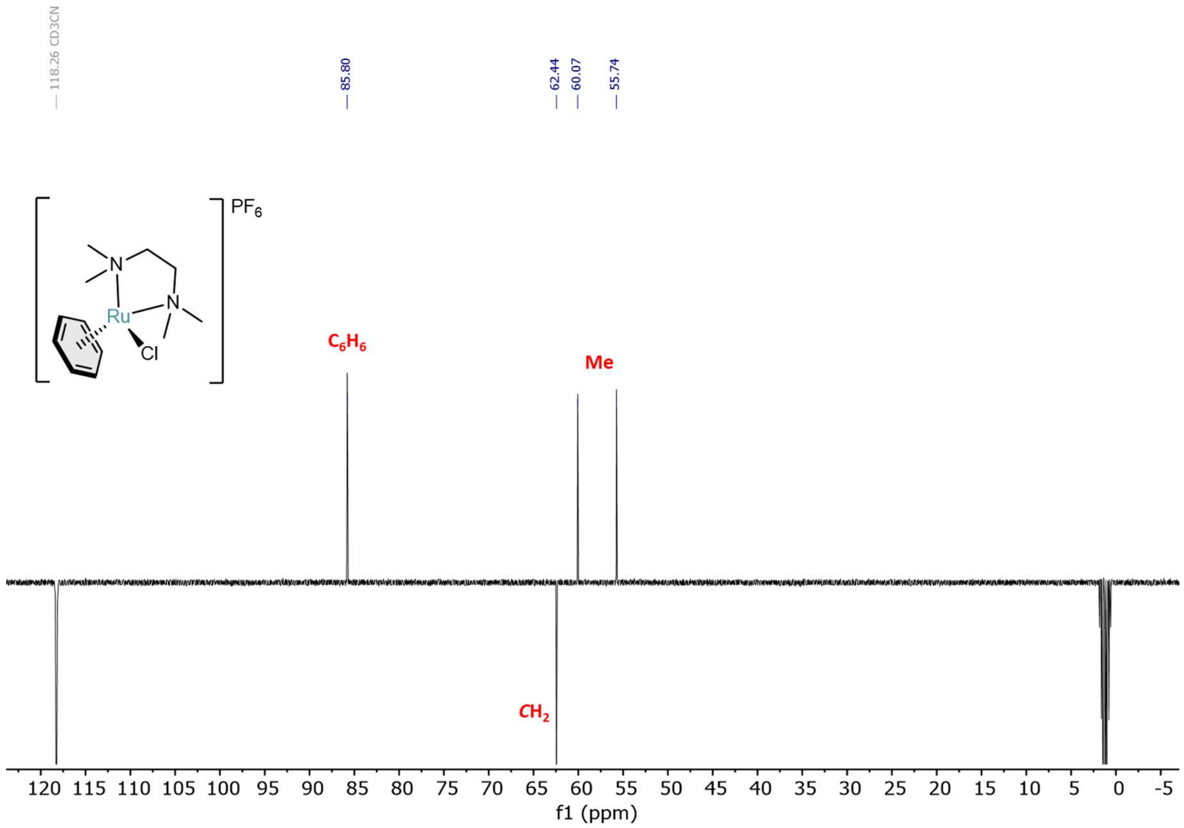

Figure S112. The ^{13}C (APT) NMR spectrum of complex **[(TMEDA)RuCl(C_6H_6)]PF_6** in CD_3CN at 298 K.

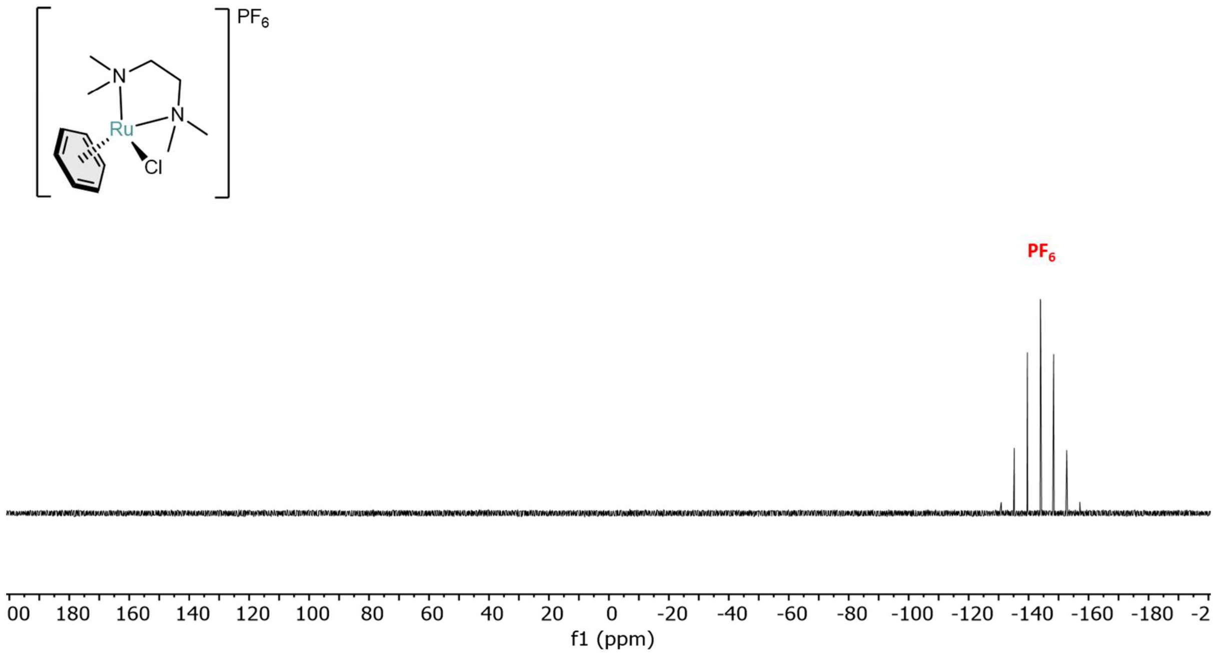

Figure S113. The $^{31}P\{^{1}H\}$ NMR spectrum of complex **[(TMEDA)RuCl(C_6H_6)]PF_6** in CD_3CN at 298 K.

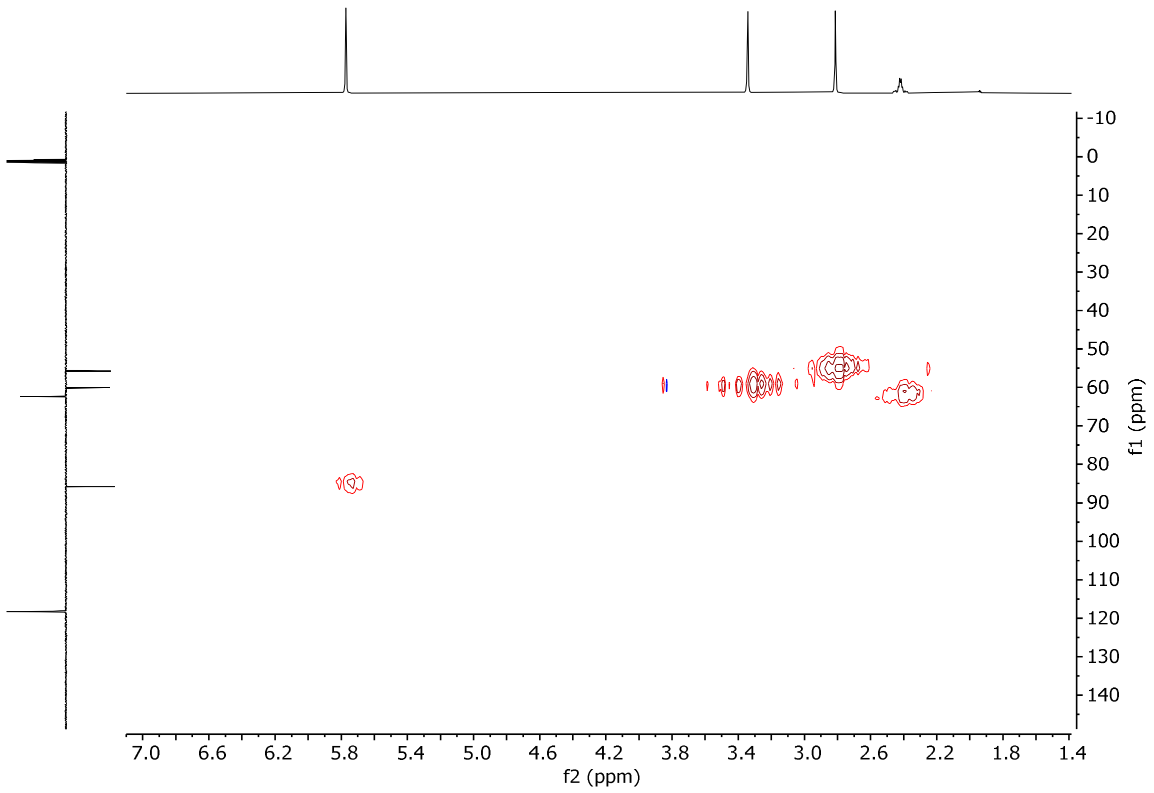

Figure S114. The ^{1}H-^{13}C ASAP-HMQC NMR spectrum of complex **[(TMEDA)RuCl(C_6H_6)]PF_6** in CD_3CN at 298 K.

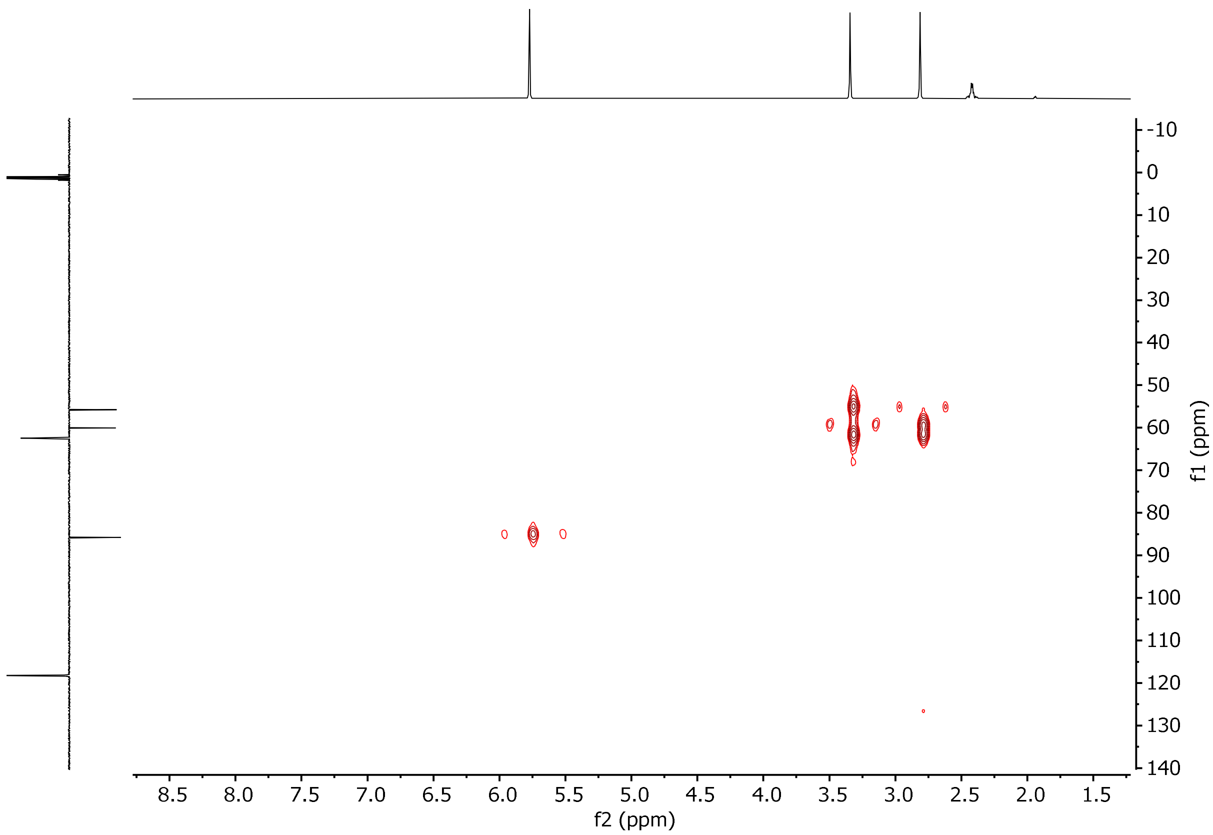

Figure S115. The ^{1}H-^{13}C HMBC NMR spectrum of complex **[(TMEDA)RuCl(C_6H_6)]PF_6** in CD_3CN at 298 K.

Reactions of the various metal complexes with KN(TMS)$_2$

General procedure for nucleophile addition

A colorless solution of KN(TMS)$_2$ (1 or 2 equiv, see Table in THF (1.5 mL) was added dropwise to an orange/yellow suspension of the complex in THF (1.5 mL). The vial with the reaction mixture was kept stirring for 30 min at RT, after which an aliquot was transferred into a J. Young tube for NMR analysis.

Complex	Equivalents of KN(TMS)$_2$	Observations upon addition	NMR results
**[(PON)Ru(C_6H_6)Cl]PF_6**	1	Color change to orange/brown	S_NArH observed
**[(NN)Ru(C_6H_6)Cl]PF_6**	1	Color change to dark brown/almost black	No hydride observed, nor characteristic signals related to formation of S_NArH product.
	2	Color change to dark brown/almost black	No hydride observed, nor characteristic signals related to formation of S_NArH product.
**[(phen)RuCl(C_6H_6)]PF_6**	1	Color change to dark brown	No hydride observed, nor characteristic signals related to formation of S_NArH product.
**[(TMEDA)RuCl(C_6H_6)]PF_6**	1	Color change to reddish purple	No hydride observed, nor characteristic signals related to formation of S_NArH product.
**[(^{t}Bu-bpy)RuCl(C_6H_6)]PF_6**	1	Color change to reddish purple	No hydride observed, nor characteristic signals related to formation of S_NArH product.

Table S1. Summary of the experiments between synthesized complexes and KN(TMS)$_2$.

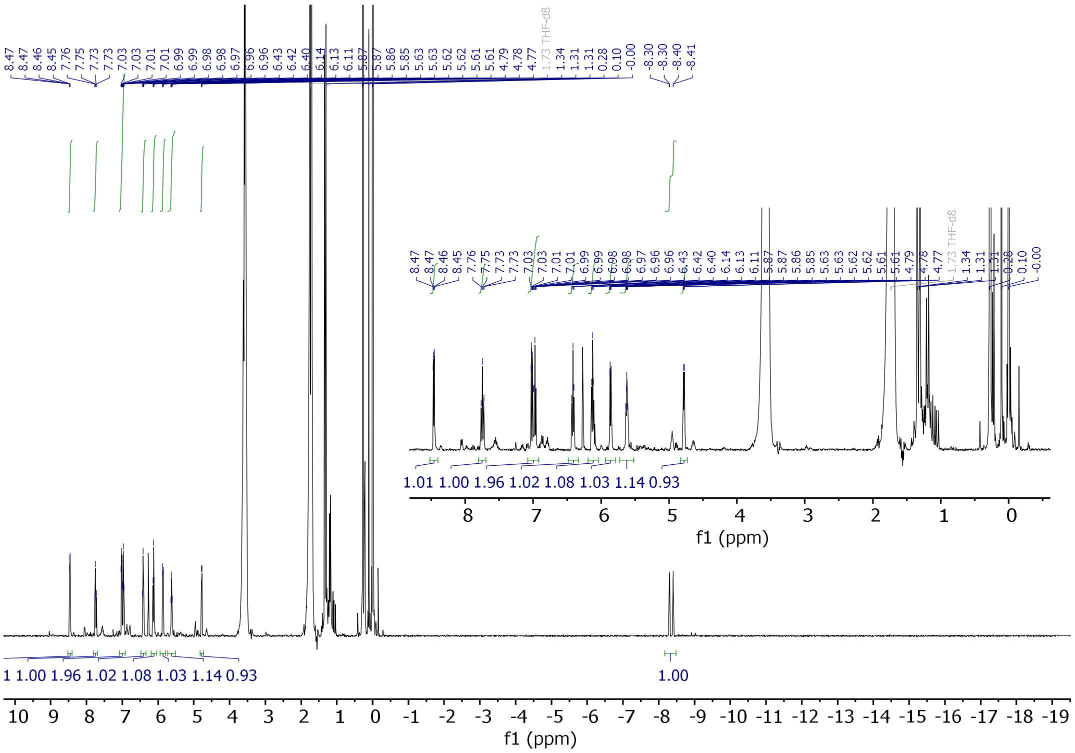

Figure S116. The ^{1}H NMR spectrum of crude mixture of the reaction between **[(PON)RuCl(C_6H_6)]PF_6** and 1 equiv $KN(TMS)_2$ in THF (WET solvent suppression was used).

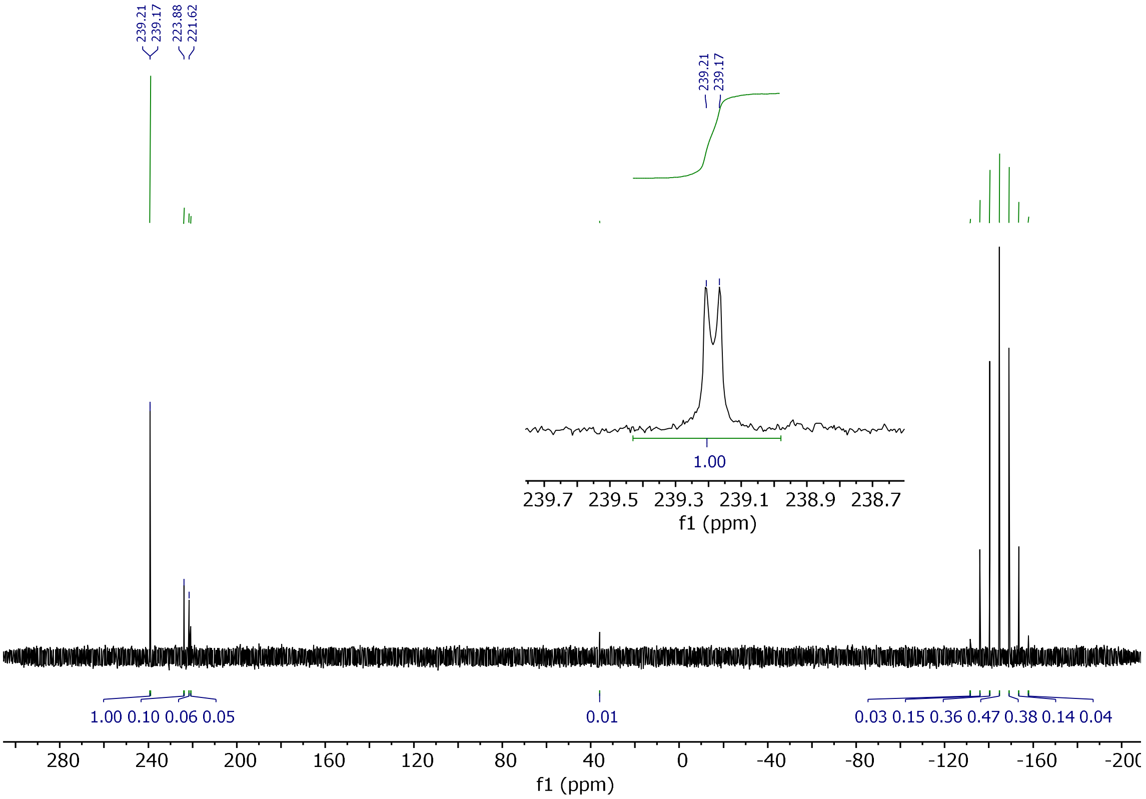

Figure S117. The $^{31}P\{^1H\}$ NMR spectrum of crude mixture of the reaction between **[(PON)RuCl(C_6H_6)]PF_6** and 1 equiv $KN(TMS)_2$ in THF (WET solvent suppression was used). *Hydride is not fully decoupled.

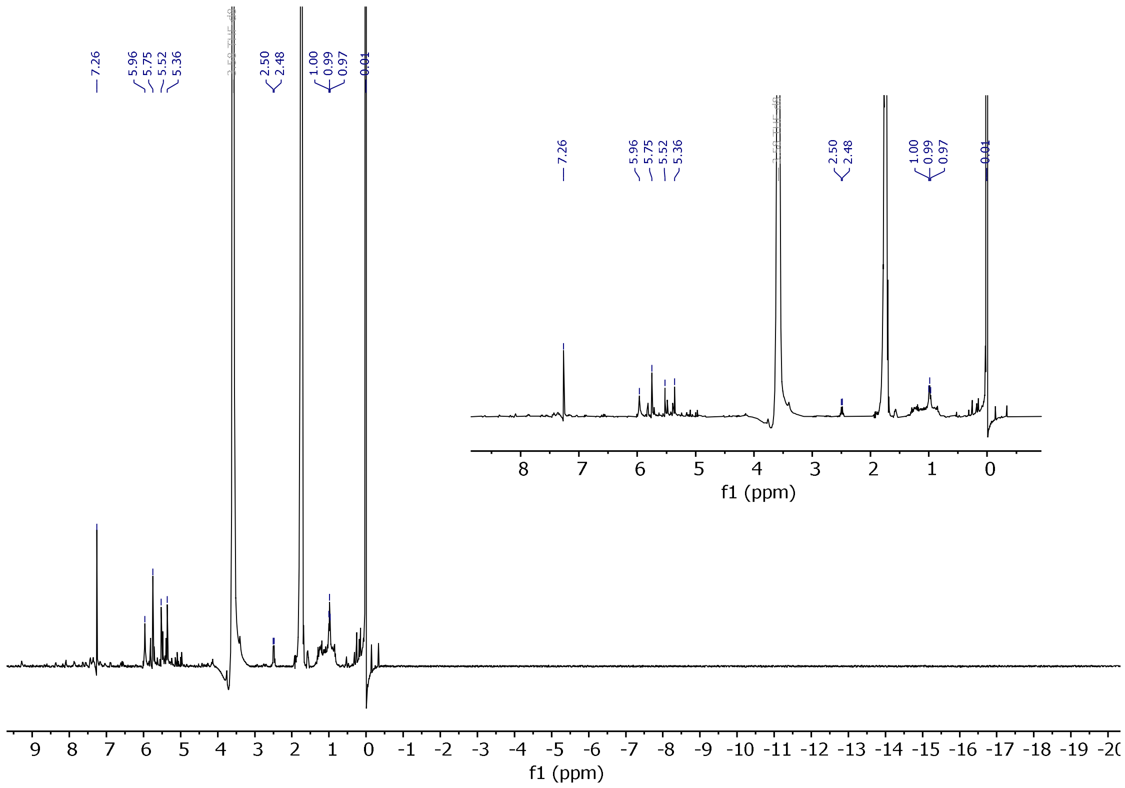

Figure S118. The ^{1}H NMR spectrum of crude mixture of the reaction between **[(NN)RuCl(C_6H_6)]PF_6** and 1 equiv KN(TMS)$_2$ in THF (WET solvent suppression was used).

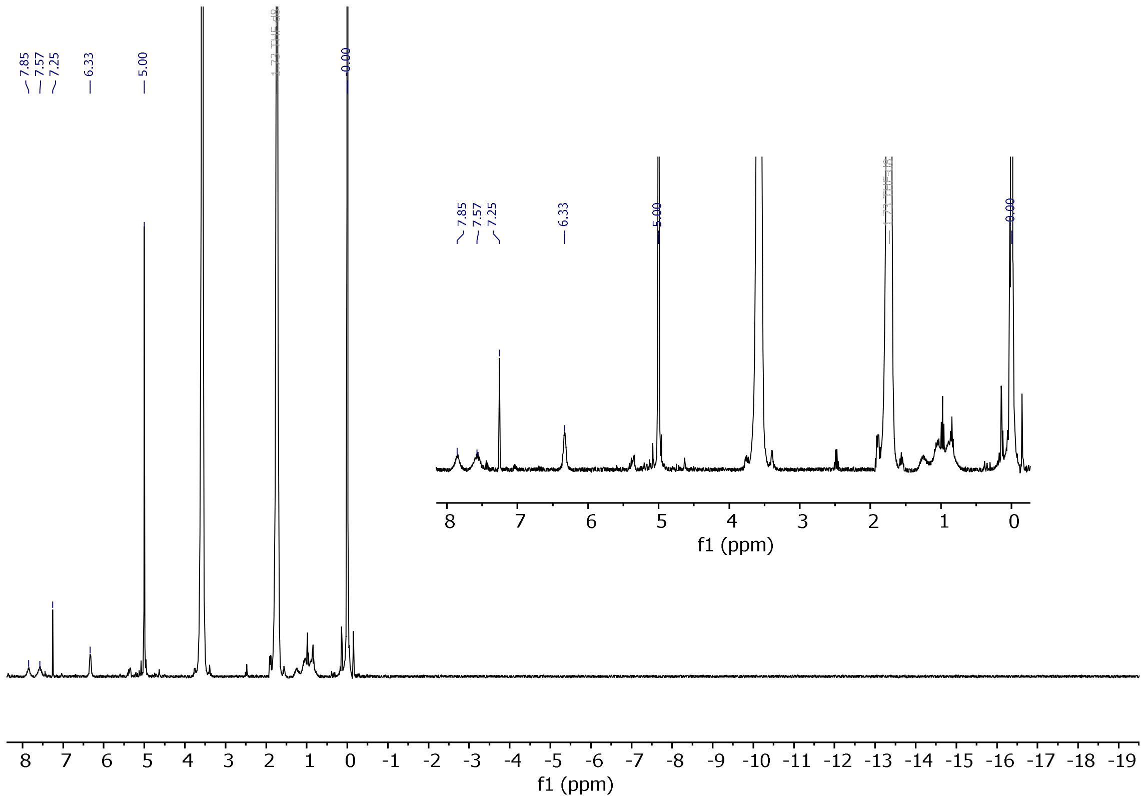

Figure S119. The ^{1}H NMR spectrum of crude mixture of the reaction between **[(NN)RuCl(C_6H_6)]PF_6** and 2 equiv KN(TMS)$_2$ in THF (WET solvent suppression was used).

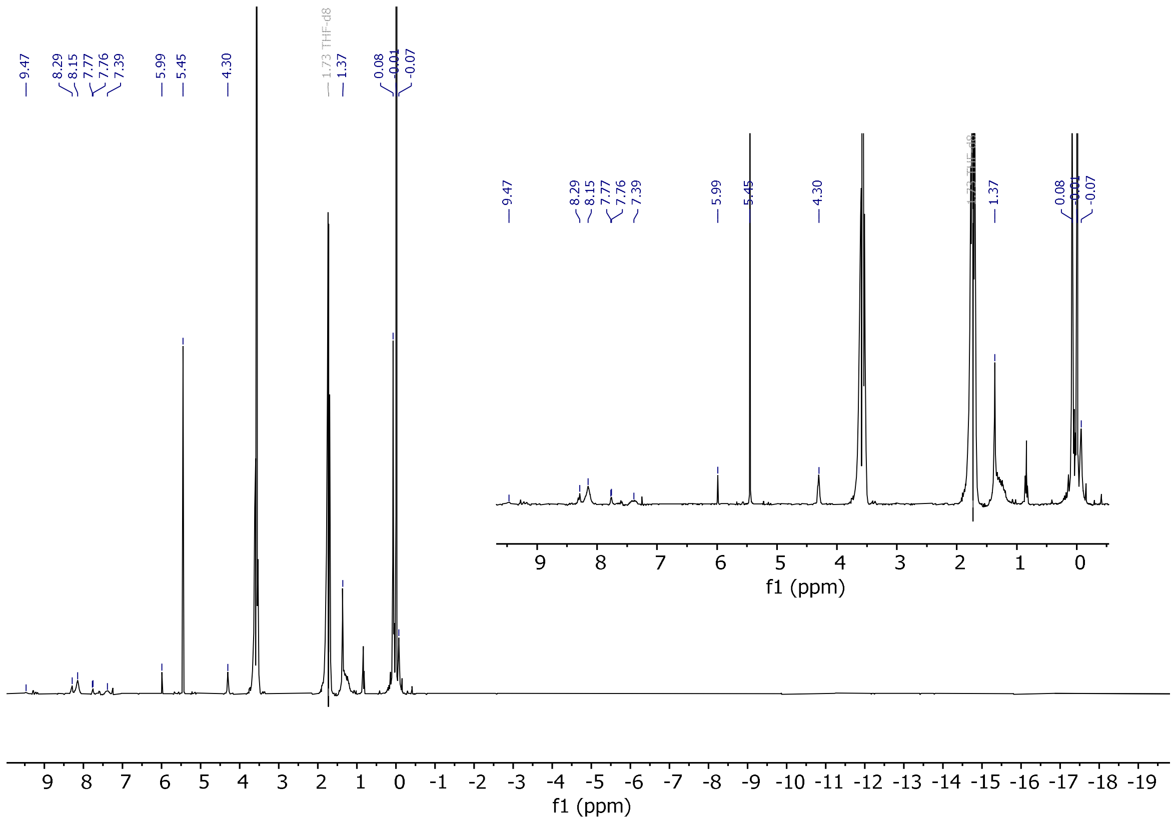

Figure S120. The ^1H NMR spectrum of crude mixture of the reaction between **[(^{t}Bu-bpy)RuCl(C_6H_6)]PF_6** and 1 equiv $KN(TMS)_2$ in THF (WET solvent suppression was used).

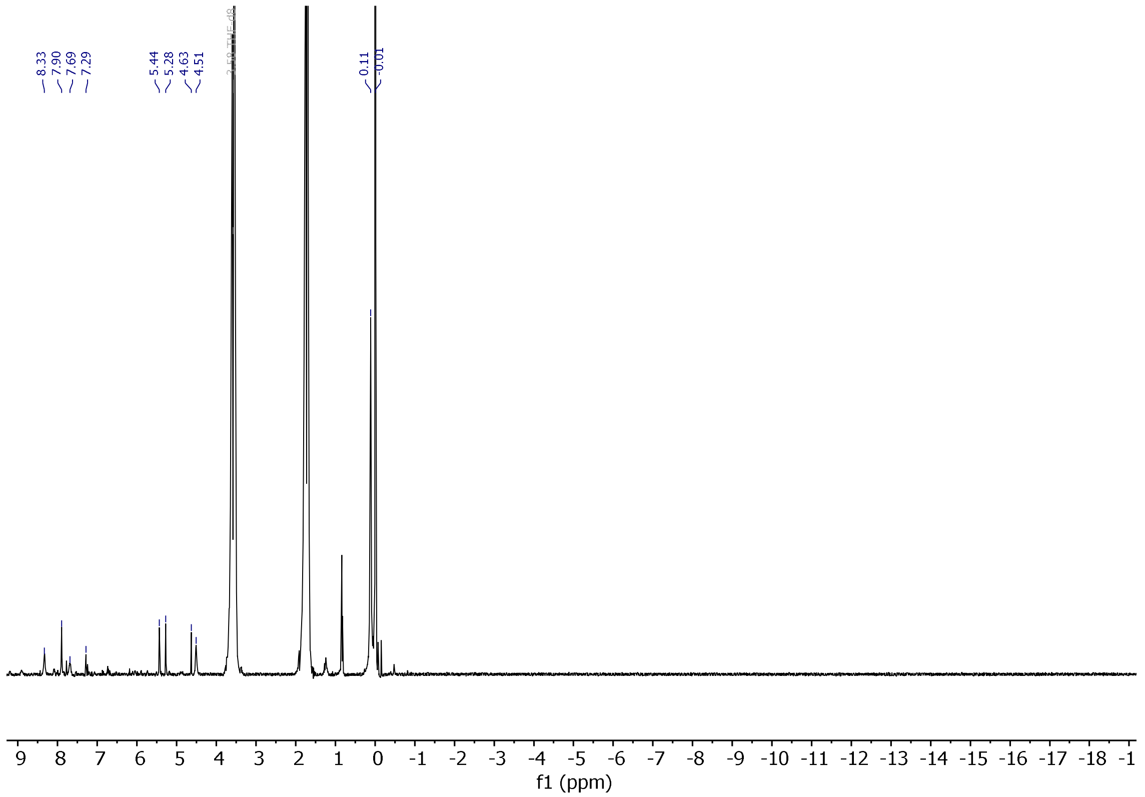

Figure S121. The ^1H NMR spectrum of crude mixture of the reaction between **[(phen)RuCl(C_6H_6)]PF_6** and 1 equiv $KN(TMS)_2$ in THF (WET solvent suppression was used).

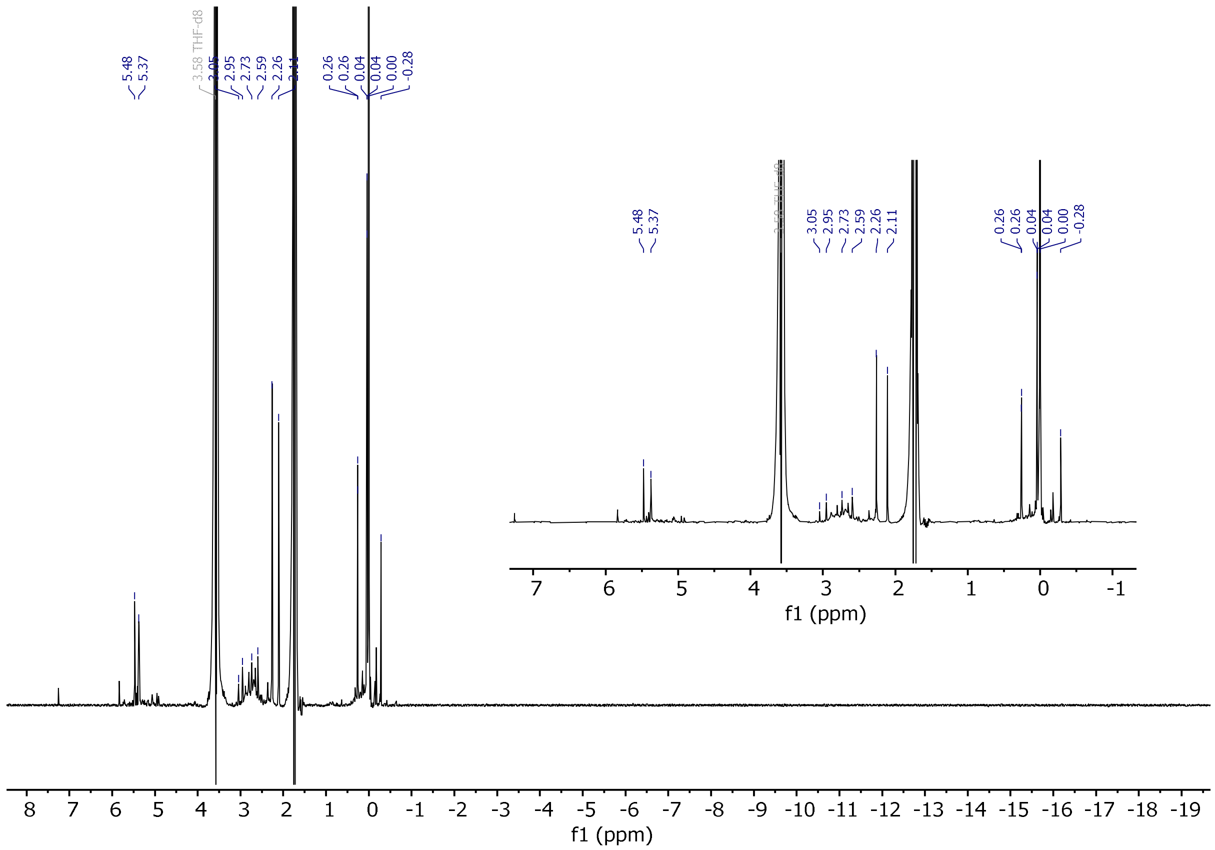

Figure S122. The ^{1}H NMR spectrum of crude mixture of the reaction between **[(TMEDA)RuCl(C_6H_6)]PF_6** and 1 equiv KN(TMS)$_2$ in THF (WET solvent suppression was used).

S3 Computational details

Computational details

All calculations were carried out using DFT[5] as implemented in ORCA 5.0.3[6–8] with the B3LYP,[9,10] including Grimme's D3 dispersion correction with Becke-Johnson Damping.[11–16] Geometry optimizations and analytical vibrational frequency calculations were carried out with the def2-SVP basis set[17] with def2-ECP for Ru.[18] For all optimized structures, the intermediates were confirmed with no imaginary vibrational frequency, while transition states showed a single imaginary frequency with a motion corresponding to the proper transitions. The solvated energies of optimized structures were re-evaluated by additional single-point calculations on each optimized geometry using the def2-TZVPD basis set.[17] For all calculations, the RIJCOSX approximation[19,20] was utilized with the auxiliary basis set def2/J.[21] To model the solution environment for tetrahydrofuran, the solvation model based on density (SMD)[22] was utilized with parameters that have been implemented in ORCA. TD-DFT calculations for modeling excited states were conducted as implemented in Q-Chem 5.4 software.[23] Geometries from the optimized geometry with ORCA were utilized for the calculations of excited states. Single Excitation Configuration Interaction (CIS)[24] and Tamm-Dancoff approximation[25] were utilized to reduce the computation cost without damage to the quality of the results. The functional and basis set for the calculations of the excited state are identical to those for DFT calculations.

The energy components have been computed with the following protocol. The free energy in solution-phase, $G^0(sol)$, has been calculated as follows, with T = 298.15 K to match the experimental conditions.

$$H(sol) = E(SCF, sol) + ZPE + Total\ thermal\ correction$$

$$G^0(sol) = H(sol) - TS(gas) + G^0(solv)$$

$$\Delta G^0(sol) = \Sigma\ G^0(sol)\ for\ products - \Sigma\ G^0(sol)\ for\ reactants$$

Literature values were utilized for the solvation energy of Cl^- (–61.76 kcal/mol)[26,27] in tetrahydrofuran. The translational entropy of Cl^- was calculated using the Sackur-Tetrode equation and its value was 36.63 cal/mol·K.

S3.1 Alternative Pathways

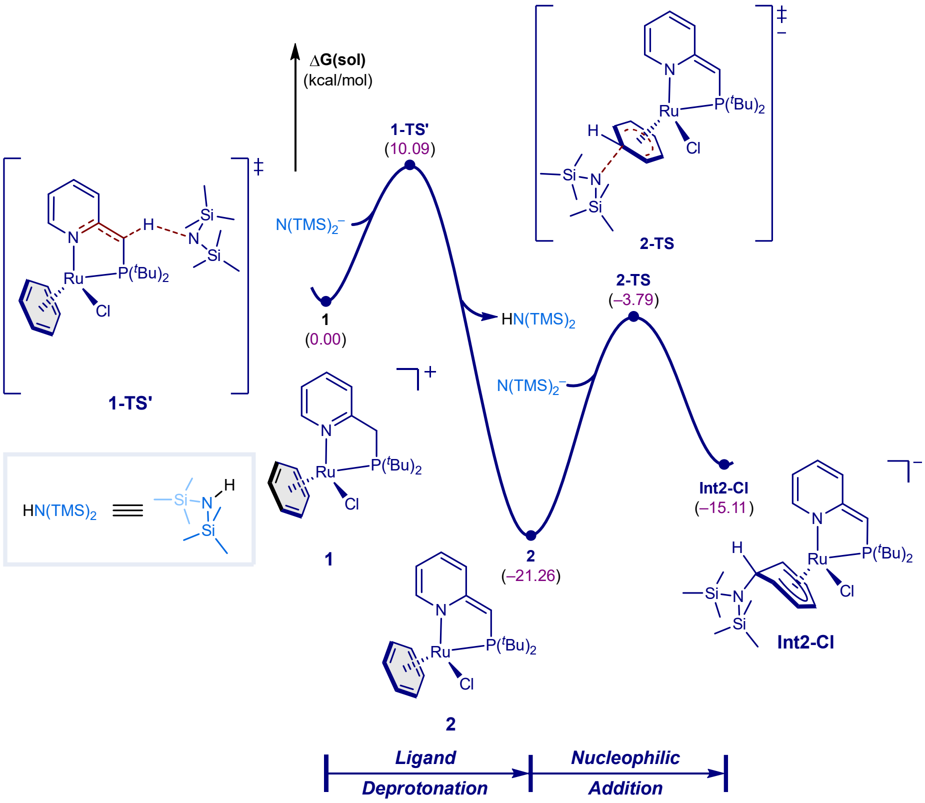

Figure S123. Computed reaction profiles where the deprotonation precedes the nucleophilic addition.

In the main text, we described the mechanistic scenario where the nucleophilic addition of the $N(TMS)_2$ anion occurs prior to PN ligand deprotonation. Fig. S123 depicts an alternative pathway where the deprotonation of the PN ligand takes place first. In this alternative mechanism, $N(TMS)_2$ anion deprotonates the methylene proton in **1** with a step barrier of 10.1 kcal/mol, which is 3.0 kcal/mol higher than the deprotonation via **1-TS**. This step generates the neutral complex **2** lying 12.9 kcal/mol lower than **Int1** (Fig. 5). Subsequently, the benzene in **2** also can be attacked by the second equivalent of $N(TMS)_2$ anion, leading to the formation of the Jackson-Meisenheimer-like intermediate, **Int2-Cl**. In this pathway, the barrier for nucleophilic addition to **2** is calculated to be 17.5 kcal/mol, which is also accessible at room temperature. These results align with the experimental observation of complex **2** after warming **Int1** from the low temperature, supporting computational results that the nucleophilic addition is reversible, with complex **2** representing the thermodynamic product while **Int1** can be regarded as the kinetic product when only 1 equivalent of base is added. (see Supplementary Section S2.2 for Low-temperature NMR studies).

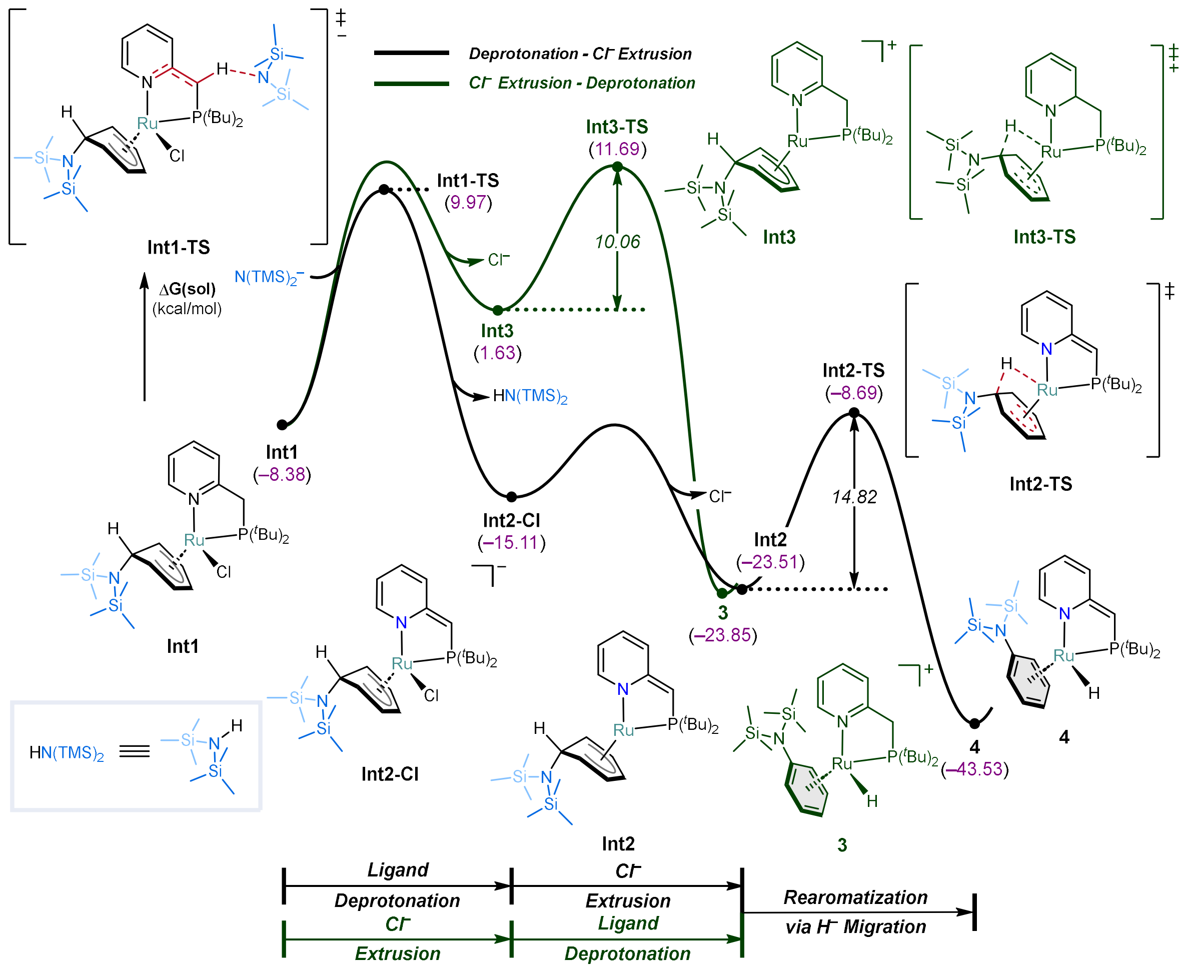

Figure S124. Computed reaction profiles where extrusion of Cl⁻ precedes the deprotonation of the ligand.

In the main pathway (Fig. 5) the methylene linker of the PN ligand in **Int1** is deprotonated to give **Int2-Cl** from which Cl⁻ extrusion gives **3**. Alternatively, Cl⁻ can dissociate first from **Int1** to give **Int3** (solid green line in Fig S124), and this step is calculated to be endergonic with a free energy change of 12.3 kcal/mol. Subsequent hydride migration can then occur via **Int3-TS** with a step barrier of 10.1 kcal/mol to give **3,** which is lower than the barrier in the main pathway. This lower step barrier arises because dearomatization of the PN ligand enhances its electron donation to the ruthenium center, which as a result becomes a less potent hydride acceptor. On the other hand, when deprotonation occurs prior to the Cl⁻ extrusion, the extrusion of chloride becomes exergonic due to the anionic nature of **Int2-Cl** and the increased electron density at the ruthenium center. Although the barrier for intramolecular hydride migration is higher in this scenario, the deprotonation step makes the extrusion of Cl⁻ exergonic, providing the driving force for Cl⁻ release. These results underscore the role of the deprotonation in facilitating Cl⁻ extrusion. Based on these calculations and experimental data, the scenario where Cl⁻ extrusion occurs before PN ligand deprotonation appears less plausible than the main pathway.

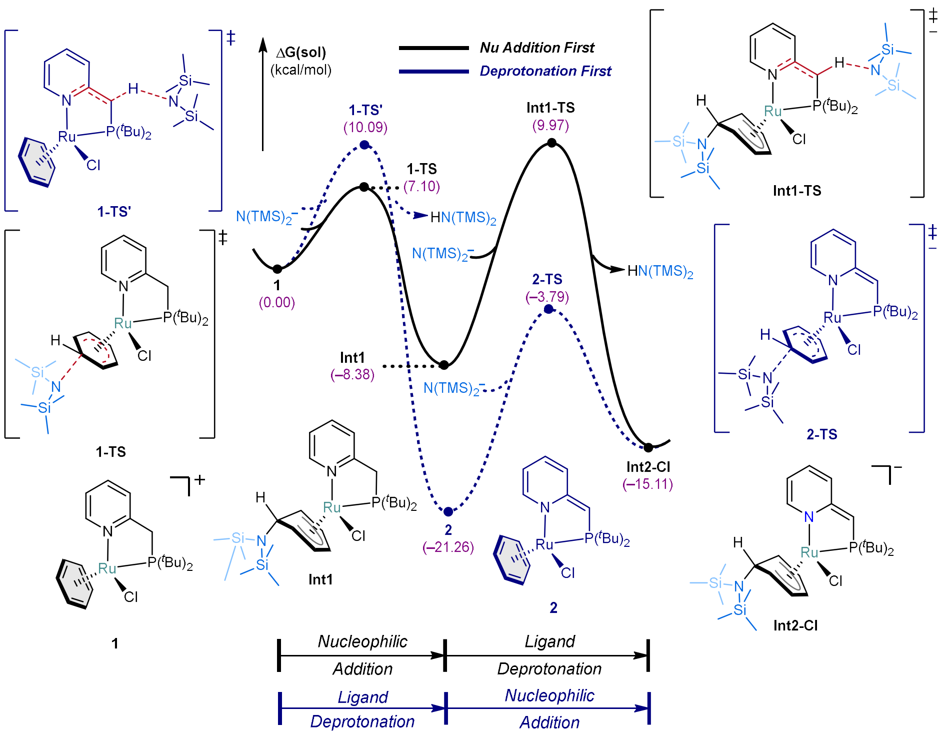

Figure S125. Computed reaction profiles which are depicted in Figure 5 and Figure S123 at 298.15 K.

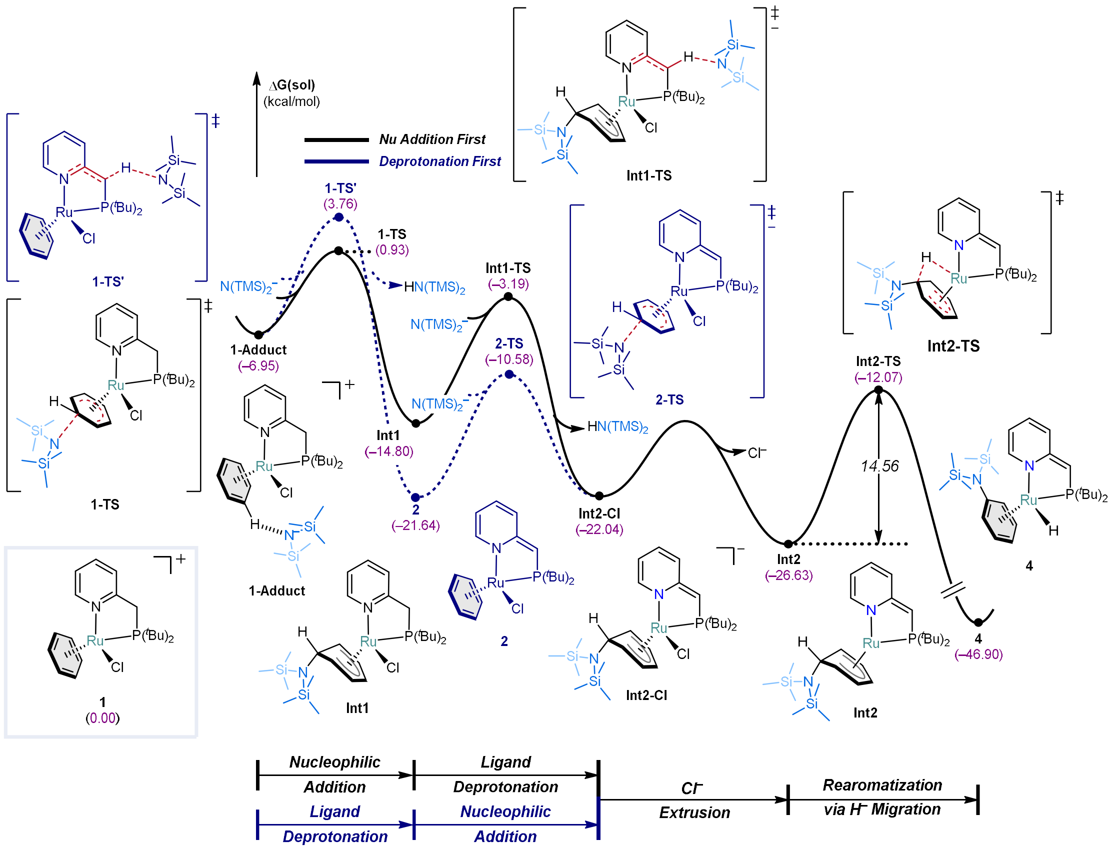

Figure S126. Computed reaction profiles which are depicted in Figure 5 and Figure S123 at 195.15 K.

To simulate the low-temperature experiment, we re-evaluated the free Gibbs energies at 195.15 K (Fig. S126). The formation of an adduct of complex **1** with $N(TMS)_2$ anion (**1-Adduct**) before the initiation of the reaction is thermodynamically favorable by –7.0 kcal/mol at this low temperature. This is the direct consequence of the reduced entropic penalty resulting from lowering temperature. Considering this favorable adduct formation, barriers for both nucleophilic addition and deprotonation were calculated to be 7.9 kcal/mol and 10.7 kcal/mol, respectively. Similar to the reaction at room temperature, the nucleophilic addition is expected to be more facile than the deprotonation of the ligand, with a barrier difference of 2.8 kcal/mol. This supports the observation of the metal-stabilized Jackson-Meisenheimer intermediate **Int1** as the major product when only one equivalent of amide base was added to the reaction mixture. Notably, the barrier for the deprotonation from **Int1** decreased to 11.6 kcal/mol at 195.15 K, which is 3.0 kcal/mol lower than the barrier for the hydride migration at this temperature. This change makes the final hydride migration have the highest barrier at low temperatures. These computation results strongly support the observation of **Int2** and the formation of **4** when the reaction mixture is warmed up. In conclusion, a combination of both experimental and computational investigations supports that the main pathway we suggest is the most plausible scenario.

S3.2 Analysis of Fukui Functions

Condensed Fukui indices[28,29] were calculated by using the equations below.

For Nucleophilic Attack:

$$f^{+} = q^{i}{}_{N} - q^{i}{}_{N+1}$$

The $q^{i}{}_{N}$ is the partial charge of atom i in the complex with N electrons, while $q^{i}{}_{N+1}$ is the partial charge of atom i in the molecule with N+1 electrons. For the calculation of $q^{i}{}_{N}$, the Hirshfeld charges[30] were calculated on each optimized geometry using the def2-TZVPD basis set and $q^{i}{}_{N+1}$ were calculated from the same geometry corresponding to the N-electron counterpart.

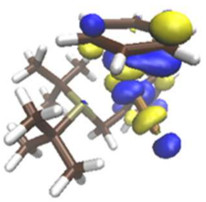

LUMO
(–4.904 eV)

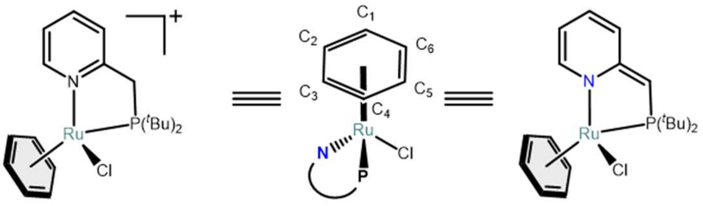

1 2

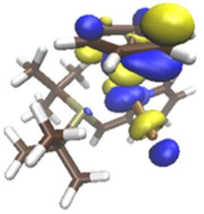

LUMO
(–1.565 eV)

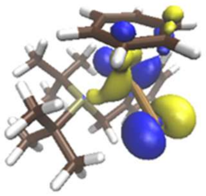

HOMO
(–8.966 eV)

	1			2		
Atom	$q_N(r)$	$q_N(r)$	$f^+(r)$	$q_N(r)$	$q_{N+1}(r)$	$f^+(r)$
**C_1**	–0.014	–0.048	0.034	–0.029	–0.069	0.040
**C_2**	–0.022	–0.048	0.026	–0.037	–0.065	0.028
**C_3**	–0.012	–0.045	0.033	–0.029	–0.066	0.037
**C_4**	–0.024	–0.055	0.031	–0.040	–0.074	0.034
**C_5**	0.010	–0.053	0.063	–0.008	–0.084	0.076
**C_6**	–0.020	–0.051	0.031	–0.033	–0.069	0.036

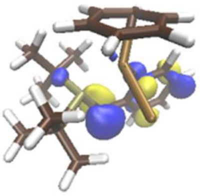

HOMO
(–4.229 eV)

Figure S127. Calculated condensed Fukui functions and the frontier orbitals of intermediates 1 and 2. The images of the molecular orbital were generated with Visual Molecular Dynamics (VMD) software.[31] The contour value was set to 0.05 a.u.

The condensed Fukui function of both complex **1** and **2** for nucleophilic addition were calculated, to assess the effect of PN ligand protonation state (Fig. S127). The condensed Fukui function predicts C_5, which is the proximal carbon to the chloride, as the most likely site for the nucleophilic in both intermediates. Similarly, analysis of the frontier orbitals (HOMO and LUMO) shows the largest LUMO coefficient on C_5 for both complexes. Based on these results, we modeled the nucleophilic addition at C_5.

S3.3 TD-DFT studies

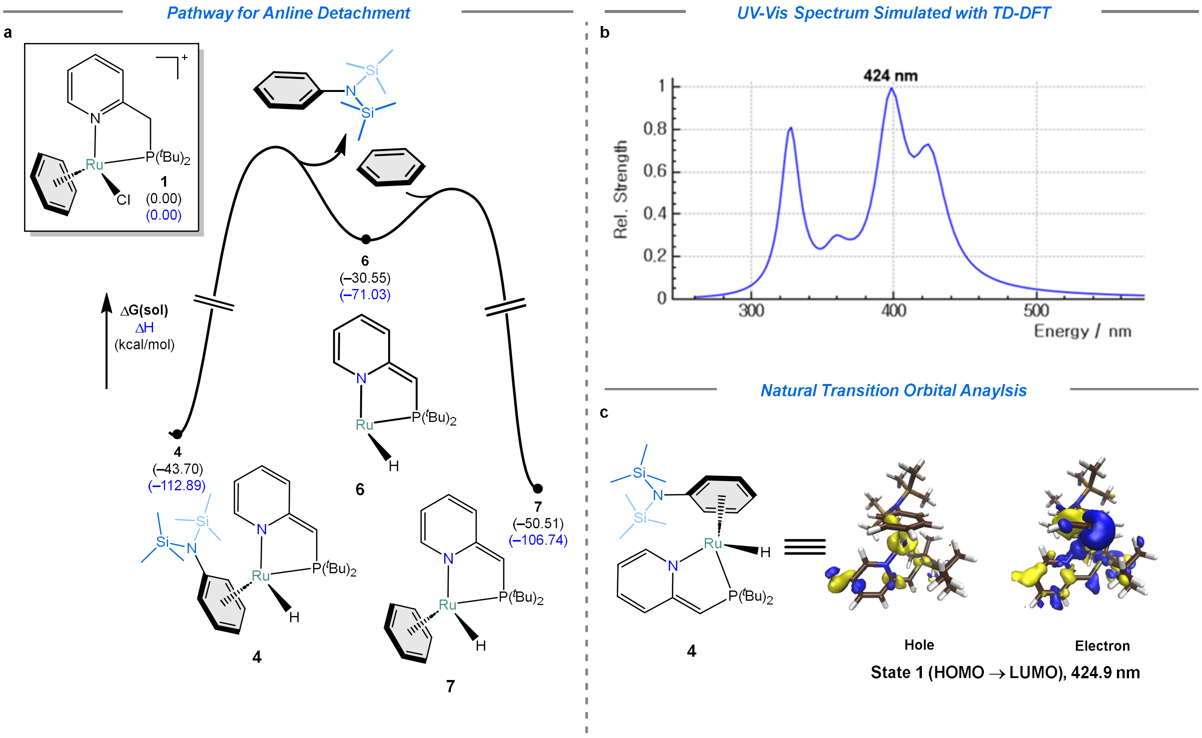

Figure S128. Analysis for aniline detachment step (a) Computed profile for the exchange of benzene with the product. (b) UV-Vis spectrum simulated with TD-DFT calculation. The image was generated from IQmol software. (c) The images of the natural transition orbital for the transition with the highest oscillator strength were generated with Visual Molecular Dynamics (VMD) software.[21] The contour value was set to 0.05 a.u.

To gain insight into the underlying reason why arene exchange in complex **4** is challenging, we further investigated the step of detachment of the aniline product (Fig. S128a). The liberation of the product was calculated to be endergonic by 13.5 kcal/mol, yielding a neutral 12-electron intermediate **6**. Additionally, this process involves 41.9 kcal/mol of enthalpy change, which reflects a strong binding affinity between the ruthenium center and the product. Due to this strong binding, the product can only be liberated when **4** is irradiated with 365 nm UV light (Fig. S128b). From the TD-DFT calculations, the transition where an electron moves from HOMO to LUMO+1 was expected to have the largest probability of occurrence (Table S2). In addition to this, the HOMO to LUMO transition was calculated to have a significant transition probability. With the natural transition orbital (NTO) analysis, those transitions were attributed to the electron transfer from the metal center to the product, causing the liberation of the product (Fig. S128c).

State	Energy (eV)	Energy (nm)	*f* (Oscillator strength)	Contribution
1	2.918	424.9	0.02662	HOMO → LUMO (80.6%)
2	3.120	397.4	0.04166	HOMO → LUMO+1 (80.6%)
3	3.361	368.9	0.00164	HOMO–1 → LUMO (89.3%)
4	3.458	358.5	0.00711	HOMO → LUMO+2 (86.7%)
5	3.599	344.5	0.00030	HOMO → LUMO+3 (70.8%)
6	3.689	336.1	0.00010	HOMO–2 → LUMO (83.1%)
7	3.796	326.6	0.03744	HOMO–1 → LUMO+1 (83.4%)

Table S2. TD-DFT calculated singlet excited states for **7**. Only orbital contributions > 15% are shown.

S4 Scope of nucleophiles

S4.1 Optimisation of the arene decoordination protocol

TMB = trimethoxybenzene, PP = 1,2-Bis(dicyclohexylphosphino)ethane. For the UV light irradiation experiments, a set-up was used that consisted of a double-walled quartz tube and a UV light source[32]. The UV light source consists of flexible Waveform Lighting realUVTM 365 nm LED strip lights (2.46 W per 1 meter) wrapped around a brass rod. This type of LED has a single sharp peak at 365 nm in the spectrum. The rod with the LED lights is placed inside the double-walled quartz tube, which is actively cooled with water during irradiation experiments ("cold" UV). When the water cooling is not used ("hot" UV), the quartz tube gets warm (~45 °C). J. Young valved NMR tubes containing the solutions of complexes were then placed around the quartz tube. The standard distance of 1-3 mm between the lamp and an NMR tube was used unless different is stated (see Supplementary Fig. S129).

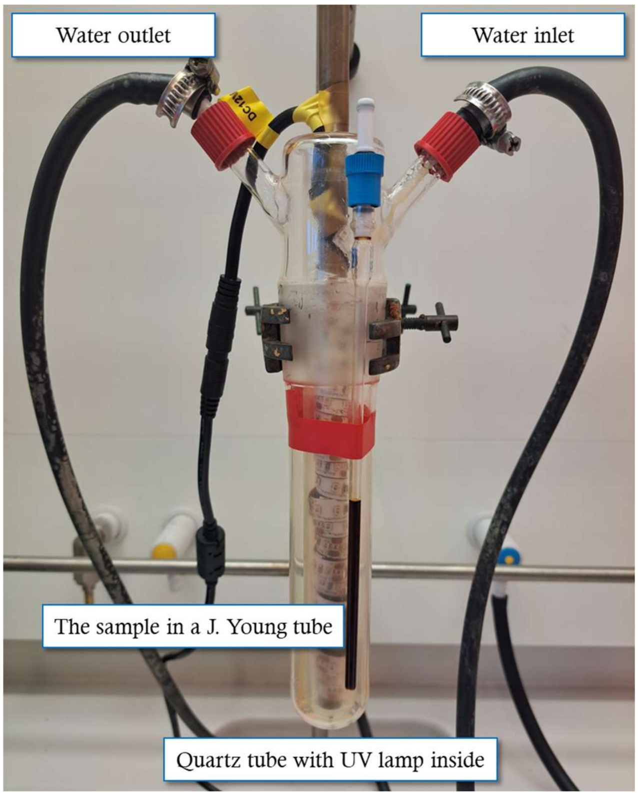

Figure S129. A standard set-up used for the photochemical experiments.

Diverse conditions for getting metal-free substitution product were tested. A summary of the screened conditions is shown in Table S3. All the experiments were performed on freshly synthesized complex **4**.

Entry	Abbreviation	Protocol outline	Yield, % (GC)
**1**	TMB in C_6H_6, 71h "hot" UV	The residue after THF removal from the synthesis of **4** was dissolved in C_6H_6 (~ 1.0 mL) and an excess of TMB was added. The solution was placed in a J.Young tube and irradiated with UV (standard conditions, without any external cooling, resulting in lukewarm heating) for 71 hours. A filter pipette with ~7 cm of alumina was prepared and the solution was filtered with the pre-wet alumina plug (THF). The residues in the NMR tube were extracted with additional THF (3 x 2.0 mL) and filtered through the same alumina plug always keeping it wet. The solvent was removed under vacuum and the residue was redissolved in 1 mL of THF and placed in a GC vial. 0.5 mL of an internal standard solution (8.0 mM solution of nitrobenzene in THF) was added and the mixture was analyzed by GC analysis.	29
**2**	MeCN, 71h "cold" UV	The residue after THF removal from the synthesis of **4** was dissolved in MeCN (~ 1.0 mL). The solution was placed in a J.Young tube and irradiated with UV (with an external water cooling) for 71 hours. The same steps with alumina plug filtration and GC analysis as **entry 1** were performed.	8
**3**	MeCN, 15h "hot" UV	The same as **entry 2** but the irradiation was for 15 hours.	17
**4**	DMSO in MeCN, 15h "hot" UV	The same as **entry 3** but before the irradiation DMSO (~ 1.0 mL) was added.	0
**5**	*ᵗ*BuNC in THF, 15h "hot" UV	The same as **entry 1** but in THF instead of benzene and before the irradiation ^{t}BuNC (~ 0.1 mL) was added.	23
**6**	CO in THF, 15h "hot" UV	The same as **entry 5** but with 1.0 atm of CO instead of the addition of ^{t}BuNC.	7
**7**	PP in C_6H_6, 15h "hot" UV	The same as **entry 1** but with a small excess of 1,2-bis(dicyclohexylphosphino)ethane instead of TMB.	18
**8**	MeCN, 15h at 100 °C	The same as **entry 3** but with heating at 100 °C instead of UV irradiation.	2
**9**	MeCN, 15h at 100 °C and UV	The same as **entry 3** but with heating at 100 °C and UV irradiation (with UV lamp/J.Young tube distance of 100 mm)	6
**10**	TMB in C_6H_6, 15h "hot" UV	The same as **entry 1** but the irradiation was for 15 hours.	27
**11**	C_6H_6, 15h "hot" UV	The same as **entry 3** but in benzene.	14
**12**	C_6H_6, 15h at 100 °C	The same as **entry 8** but in benzene.	11
**13**	HCl in C_6H_6, 15h "hot" UV	The same as **entry 11** but before the irradiation step, HCl (~ 1.0 mL, 2M in Et_2O) was added.	0
**14**	HNO_3 workup	To the residue after THF removal from the synthesis of **4** 3.0 mL of HNO_3 (50%) was added. The solution was stirred for 1 hour and was neutralized by adding NaOH (2 M) solution until pH > 7. The solution was extracted with Et_2O (3.0 mL x 3) and dried over $MgSO_4$. All volatiles were removed under a dynamic vacuum, and the solid was redissolved in THF (~ 1.0 mL) and was then placed in a GC vial. 0.5 mL of the internal standard solution (8.0 mM solution of nitrobenzene in THF) was added and the mixture was analyzed by GC analysis.	0
**15**	HCl aq. workup	The same as **entry 14** but with 4M HCl.	0
**16**	HBArF, MeCN, 15h "hot" UV	The same as **entry 3** but 1.0 equiv of HBArF* was added before the irradiation.<br>(*The protonation was intended to ease the exchange rate, as it has been reported to be more facile for cationic complexes).[33]	34
**17**	C_6H_6, 72h "hot" UV	The same as **entry 11** but the irradiation was for 72 hours.	40

Table S3. Conditions tested for decoordination of the arene.

Fate of [Ru] after UV irradiation experiments

In order to get insights on what happens to complex **4** after UV irradiation, we performed experiments where we irradiated a J. Young NMR tube (equipped with a capillary with a C_6D_6 solution of hexamethylbenzene (0.012 mM) as internal standard) containing a C_6D_6 solution (0.6 mL) of freshly-made complex **4** (9.6 mg, 0.016 mmol) with UV light (the standard procedure). The mixture was analyzed at several time intervals (see Fig. S130-133).

According to the obtained results, a complex mixture of metal hydrides and possibly other Ru-containing species forms upon prolonged irradiation with strong UV light. Also it is noticeable that prolonged UV irradiation leads to higher yield of the "free" $PhN(TMS)_2$. ^2H NMR sheds more light on the potential formation of benzene-d_6 complexes displaying signals at ~5 ppm.

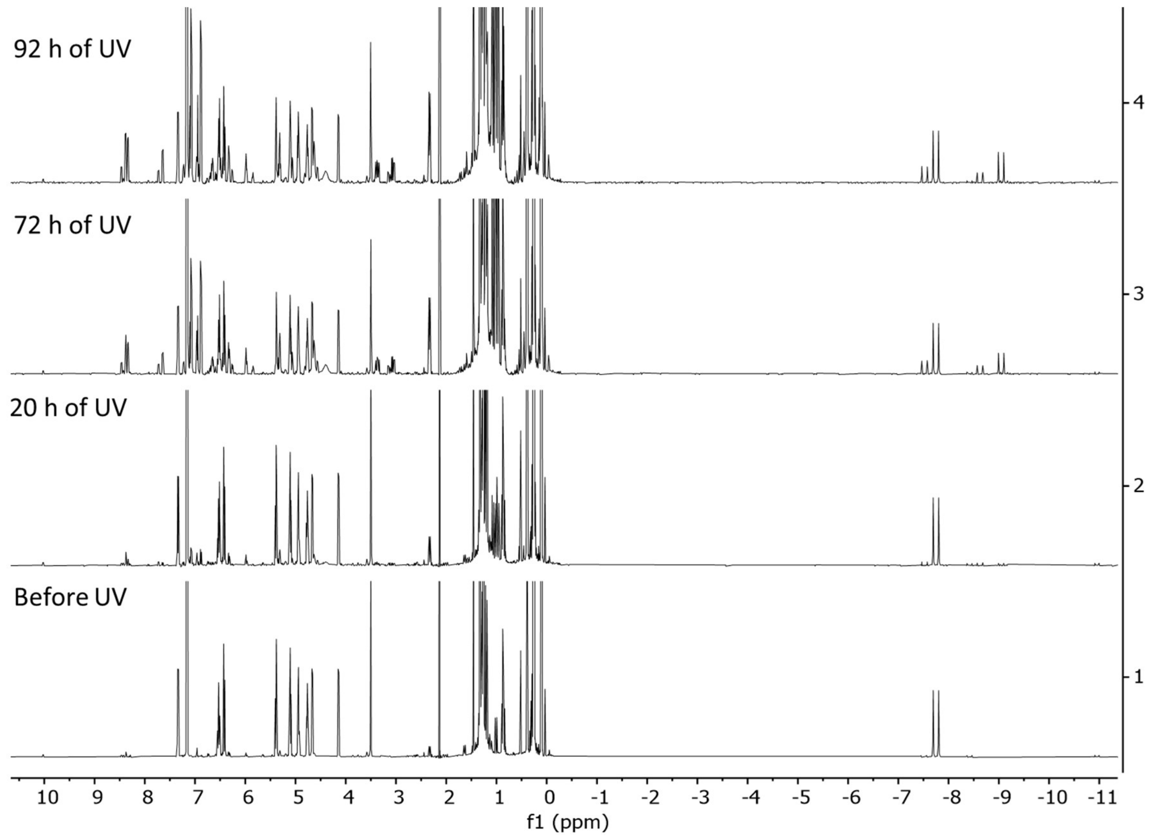

Figure S130. The ^1H NMR spectra of the solution of **4** (with hexamethylbenzene as an internal standard) in C_6D_6 at 298 K before and after UV irradiation over different amount time.

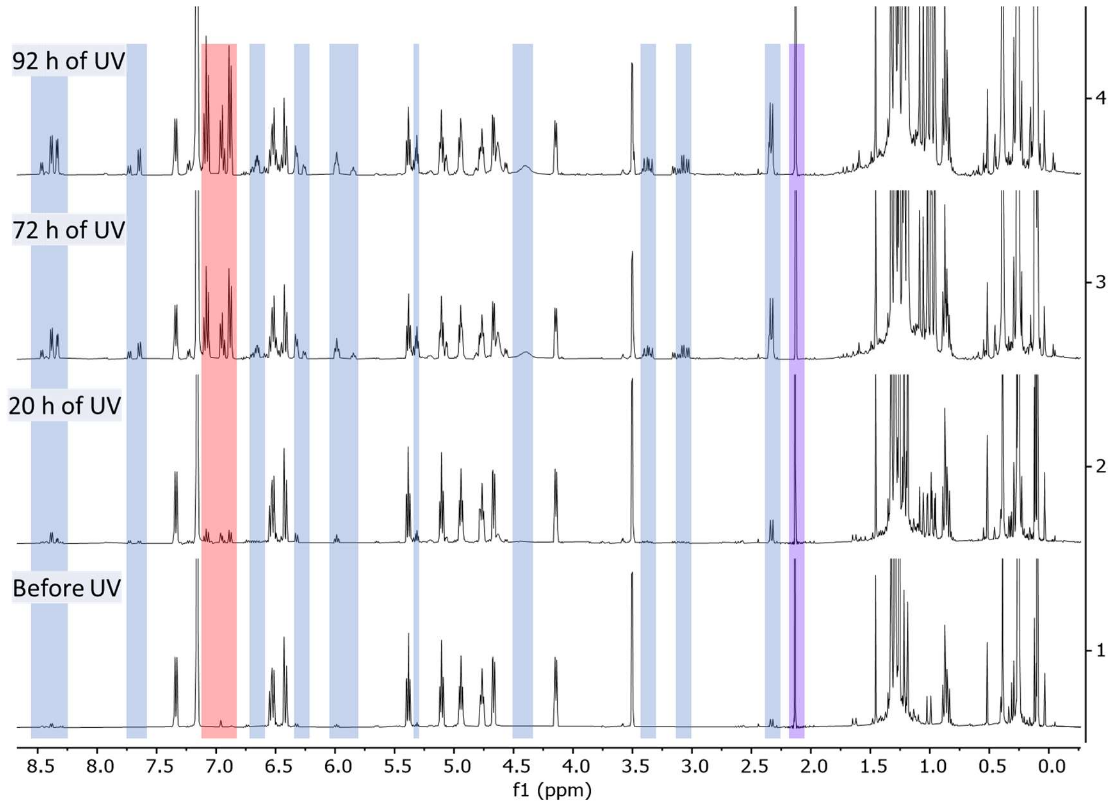

Figure S131. Zoomed-in Fig. S128. The new species are highlighted with blue, internal standard with purple and "free" $PhN(TMS)_2$ with red.

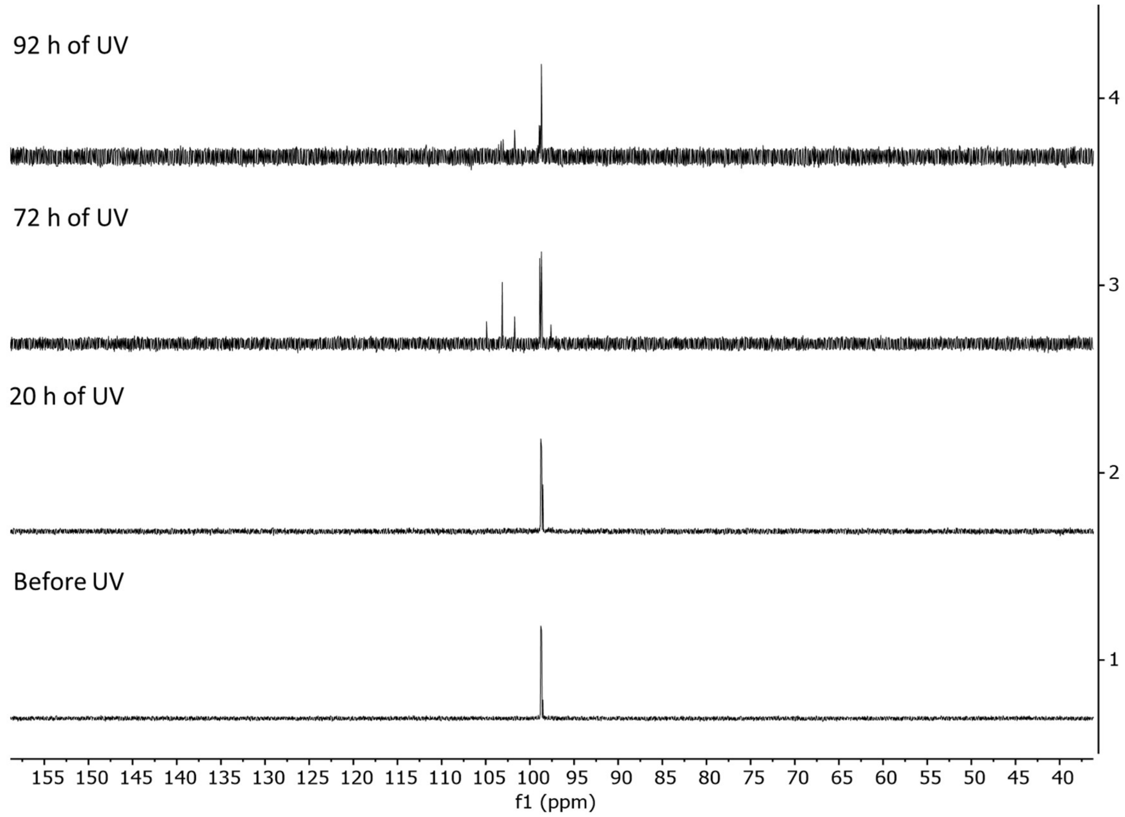

Figure S132. The ^{31}P NMR spectra of the solution of **4** (with hexamethylbenzene as an internal standard) in C_6D_6 at 298 K before and after UV irradiation over different amount time.

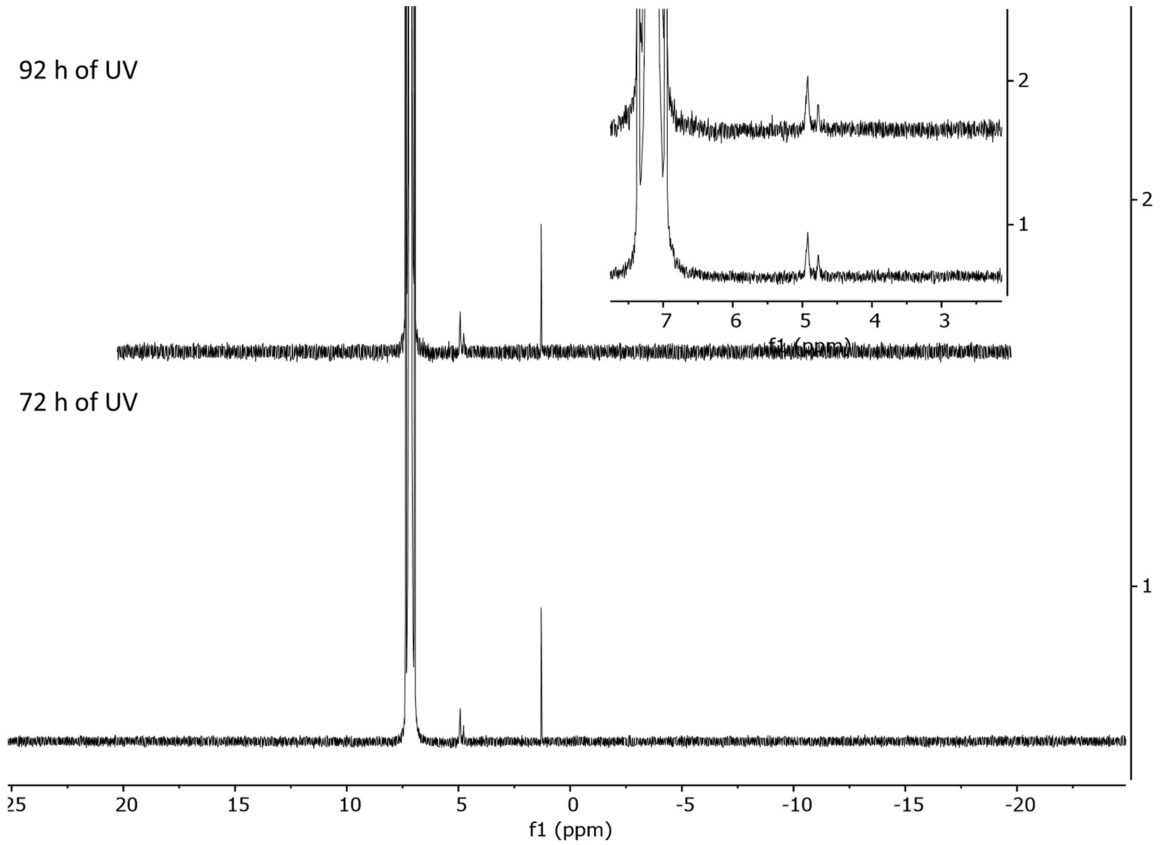

Figure S133. The ^{2}H NMR spectra of the solution of **4** (with hexamethylbenzene as an internal standard) in C_6D_6 at 298 K before and after UV irradiation over different amount time (the aromatic region is highlighted in the separate window).

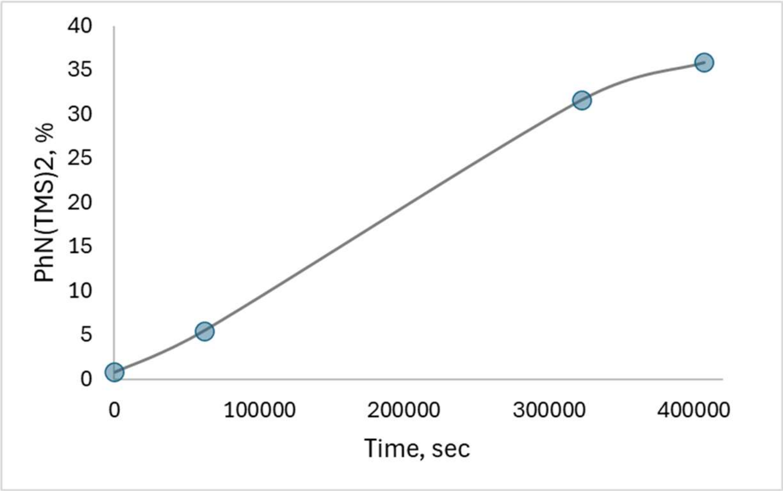

Figure S134. Dependence of the yield (based on ^{1}H qNMR) of $PhN(TMS)_2$ *vs* time of UV irradiation of a solution of complex **4** in C_6D_6.

S4.2 Experiments with other nucleophiles

Standard procedure for the workup after the S_NArH

The solid after a S_NArH reaction was dissolved in a mixture of benzene (1.5 mL) and THF (0.1 mL). The solution was placed in a J. Young tube and irradiated with UV for 72 hours in a fumehood to give a brown solution. A filter pipette with ~7 cm of alumina was prepared and the solution was filtered with the pre-wet alumina plug (THF). The residues in the NMR tube were extracted with additional THF (3 x 2.0 mL) and filtered through the same alumina plug keeping it always wet. The solvent was removed under vacuum and the residue was redissolved in THF (1.0 mL) and placed in a GC vial. 0.5 mL of the internal standard solution (8 mM solution of nitrobenzene in THF) was added and the mixture was analyzed by GC.

Reaction with potassium bis(trimethylsilyl)amide ($KN(TMS)_2$)

A yellow suspension of complex **1** (11.9 mg, 0.02 mmol) in THF (2.0 mL) was added dropwise to a colorless solution of $KN(TMS)_2$ (8.0 mg, 0.04 mmol) in THF (3.0 mL) at RT. The starting complex instantly dissolved upon the addition causing a change in the color of the resulting solution to dark brown. The vial with the reaction mixture was kept stirring for 0.5 h at RT in a glove box. The mixture was dried under a dynamic vacuum to give a dark brown glass-like solid. The standard procedure was applied to give ~0.008 mmol (40 %) of N,N-bis(trimethylsilyl)aniline according to GC analysis.

Reaction with benzyl potassium (BnK)

A yellow suspension of complex **1** (11.9 mg, 0.02 mmol) in THF (2.0 mL) and a red solution of BnK (5.2 mg, 0.04 mmol) in THF (3.0 mL) were cooled down in a cold well of a glove box (using an acetone/dry ice bath). After 10 minutes, the suspension of **1** was added dropwise to the solution of the BnK using a precooled (in the cold well) pipette, and the color instantly changed to dark brown. The acetone/dry ice bath was removed, and the mixture was allowed to warm up. After 1 hour of vigorous stirring, the THF was removed under a dynamic vacuum to give a brown solid. The standard procedure was applied to give ~0.006 mmol (31 %) of diphenylmethane according to GC analysis.

Reaction with *n*-butyl lithium (^{n}BuLi)

A yellow suspension of complex **1** (11.9 mg, 0.02 mmol) in THF (2.0 mL)and a colorless solution of ^{n}BuLi (25.0 mL, 0.04 mmol, 1.6 M) in THF (3.0 mL) were cooled down in a cold well of a glove box (using an acetone/dry ice bath). After 10 minutes, the suspension of **1** was added dropwise to the solution of the ^{n}BuLi a precooled (in the cold well) pipette and the color instantly changed to dark red. The acetone/dry ice bath was removed, and the mixture was allowed to warm up. After 1 hour of vigorous stirring, the THF was removed under a dynamic vacuum to give an orange solid. The standard procedure was applied to give ~0.002 mmol (9 %) of *n*-butylbenzene according to GC analysis.

Reaction with phenyl magnesium bromide (PhMgBr)

A yellow suspension of complex **1** (11.9 mg, 0.02 mmol) in THF (2.0 mL)and a greenish solution of PhMgBr (40.0 mL, 0.04 mmol, 1.0 M) in THF (3.0 mL) were cooled down in a cold well of a glove box (using an acetone/dry ice bath). After 10 minutes, the suspension of **1** was added dropwise to the solution of the PhMgBr using a precooled (in the cold well) pipette, and the color instantly changed to dark brown. The acetone/dry ice bath was removed, and the mixture was allowed to warm up. After 1 hour of vigorous stirring, the THF was removed under a dynamic vacuum to give a dark green solid. The solid was washed with 5.0 mL of pentane to get rid of biphenyl that could form via homocoupling

of phenyl magnesium bromide[34,35]. The standard procedure was applied to give ~0.007 mmol (35 %) of biphenyl according to GC analysis.

Reaction with lithium phenyl acetylide (PhC≡CLi)

A yellow suspension of complex **1** (11.9 mg, 0.02 mmol) in THF (2.0 mL) and a colorless solution of PhC≡CLi (4.3 mg, 0.04 mmol) in THF (3.0 mL) were cooled down in a cold well of a glove box (using an acetone/dry ice bath). After 10 minutes, the suspension of **1** was added dropwise to the solution of the PhC≡CLi using a precooled (in the cold well) pipette, and the color instantly to light brown after 40 min of stirring. The acetone/dry ice bath was removed, and the mixture was allowed to warm up. After 1 hour of vigorous stirring, the THF was removed under a dynamic vacuum to give a brown solid. To the residue 3 mL of water and 1.0 mL of NaOH (4M, aqueous solution) were added. The formed dark brown solution was stirred for 1 hour. The solution was extracted with Et_2O (3 x 3.0 mL) and dried over $MgSO_4$. All volatiles were removed under a dynamic vacuum, the solid was redissolved in 1.0 mL of THF, and was then placed in a GC vial. 0.5 mL of the internal standard solution (8.0 mM solution of nitrobenzene in THF) was added and a GC chromatogram was recorded to give ~0.001 mmol (6 %) of biphenyl according to GC analysis.

Reaction with potassium diphenyl phosphide ($KPPh_2$)

A yellow suspension of complex **1** (11.9 mg, 0.02 mmol) in THF (2.0 mL) and a dark red solution of $KPPh_2$ (80.0 mL, 0.04 mmol, 0.5 M) in THF (3.0 mL) was cooled down in a cold well of a glove box (using an acetone/dry ice bath). After 10 minutes, the suspension of **1** was added dropwise to the solution of the $KPPh_2$ in a precooled (in the cold well) pipette, and the color instantly changed to dark brown. The acetone/dry ice bath was removed, and the mixture was allowed to warm up. After 1 hour of vigorous stirring, the THF was removed under a dynamic vacuum to give a dark brown solid. To the residue 3 mL of water and 1.0 mL of H_2O_2 (30% aqueous solution) were added. The formed dark brown solution was stirred for 1 hour. The left H_2O_2 was ceased by the slow addition of an excess of $Na_2S_2O_3$ (aqueous aqueous solution) till the bubbling stopped. The mixture was stirred for 30 min more and the product was extracted with DCM (3 x 1.5 mL). The organic phases were collected, dried over $MgSO_4$, and concentrated. The solid was redissolved in 1 mL of DCM, 0.5 mL of the internal standard solution (8.0 mM solution of nitrobenzene in THF) was added and a GC chromatogram was recorded to give ~0.001 mmol (6 %) of triphenylphosphine oxide according to GC analysis.

Reaction with vinyl magnesium bromide (VinMgBr)

A yellow suspension of complex **1** (11.9 mg, 0.02 mmol) in THF (2.0 mL) was added dropwise to a colorless solution of VinMgBr (40.0 mL, 0.04 mmol, 1.0 M) in THF (3.0 mL) at RT. The starting complex instantly dissolved upon the addition causing the change of the color of the resulting solution to dark green. The vial with the reaction mixture was kept stirring for 1 h at RT in a glove box. The solution was concentrated to ~0.6 mL and 10 µL of a 111 mM internal standard solution was added (hexamethylbenzene in THF). The hydride products were quantified by qNMR using the internal standard as a reference for integration which gave a 21 % spectroscopic yield.

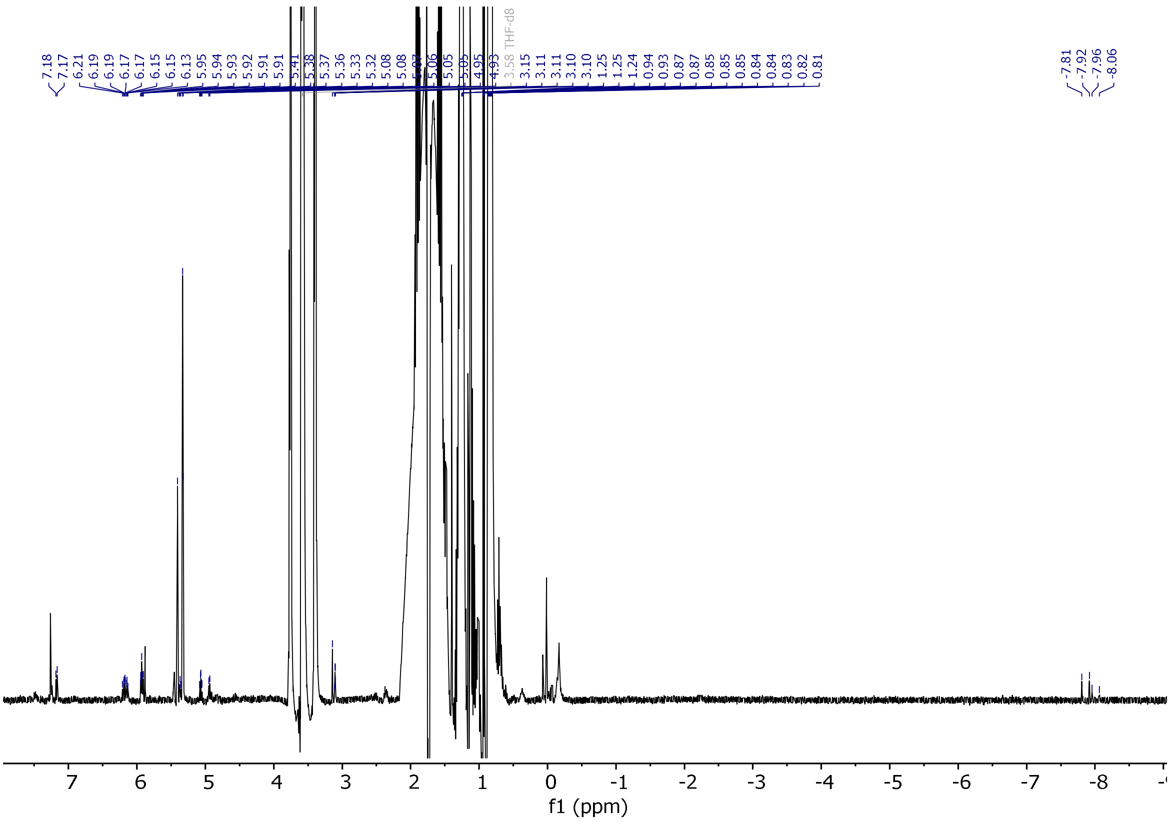

Figure S135. The ^{1}H NMR spectrum after the reaction between **1** and ^{n}BuLi (crude mixture, THF, 298 K, “PRESAT” function was used for solvent signals suppression).

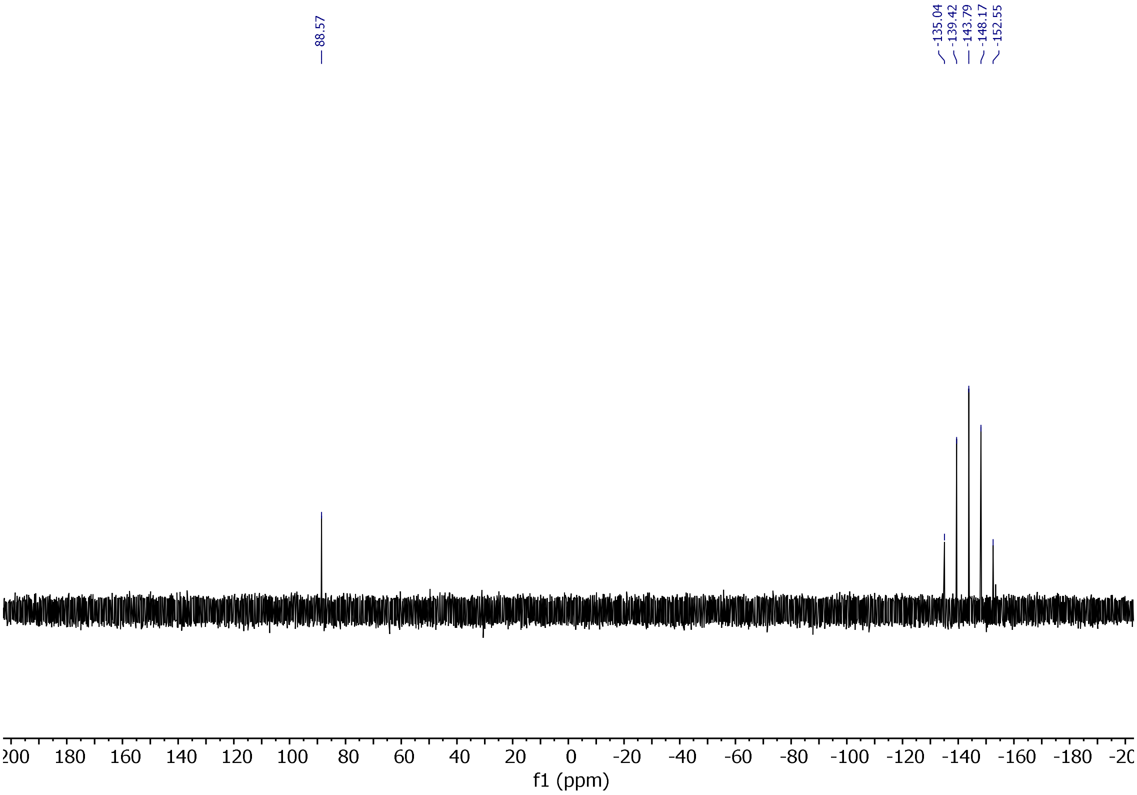

Figure S136. The ^{31}P NMR spectrum after the reaction between **1** and ^{n}BuLi (crude mixture, THF, 298 K).

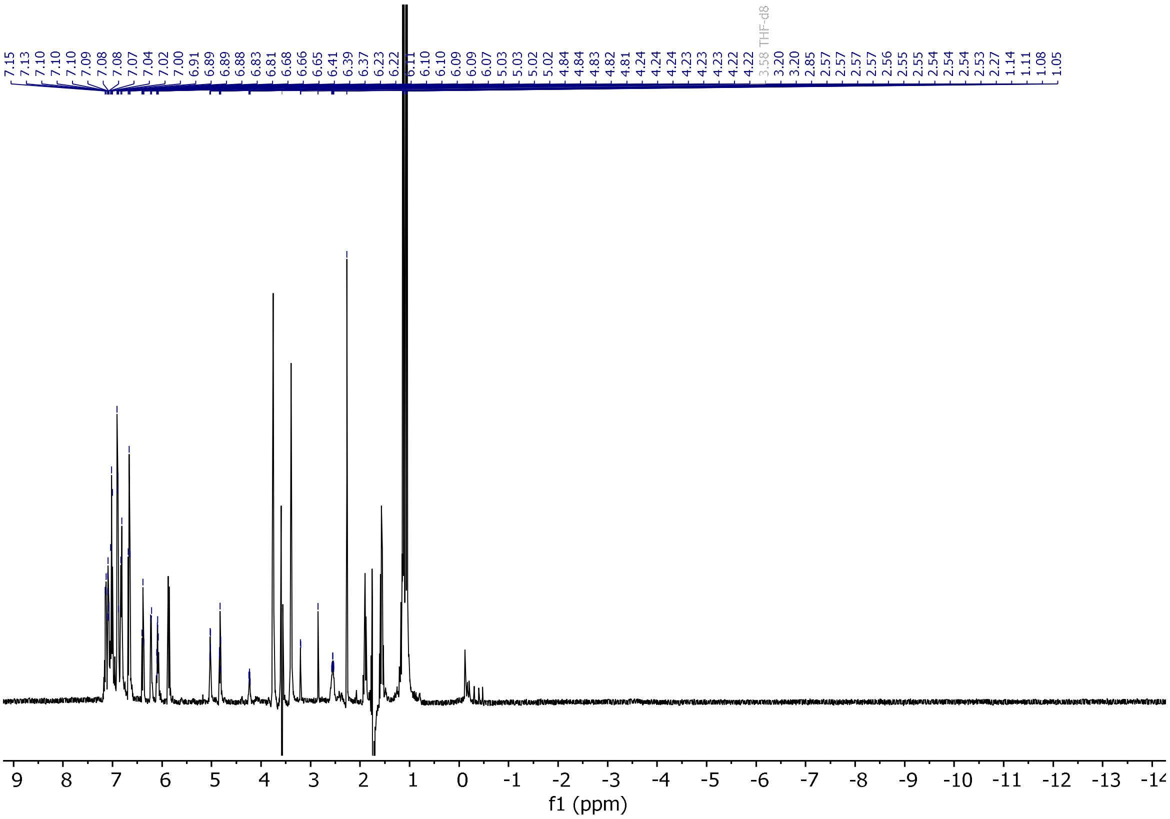

Figure S137. The ^{1}H NMR spectrum after the reaction between **1** and BnK (crude mixture, THF, 298 K, “PRESAT” function was used for solvent signals suppression).

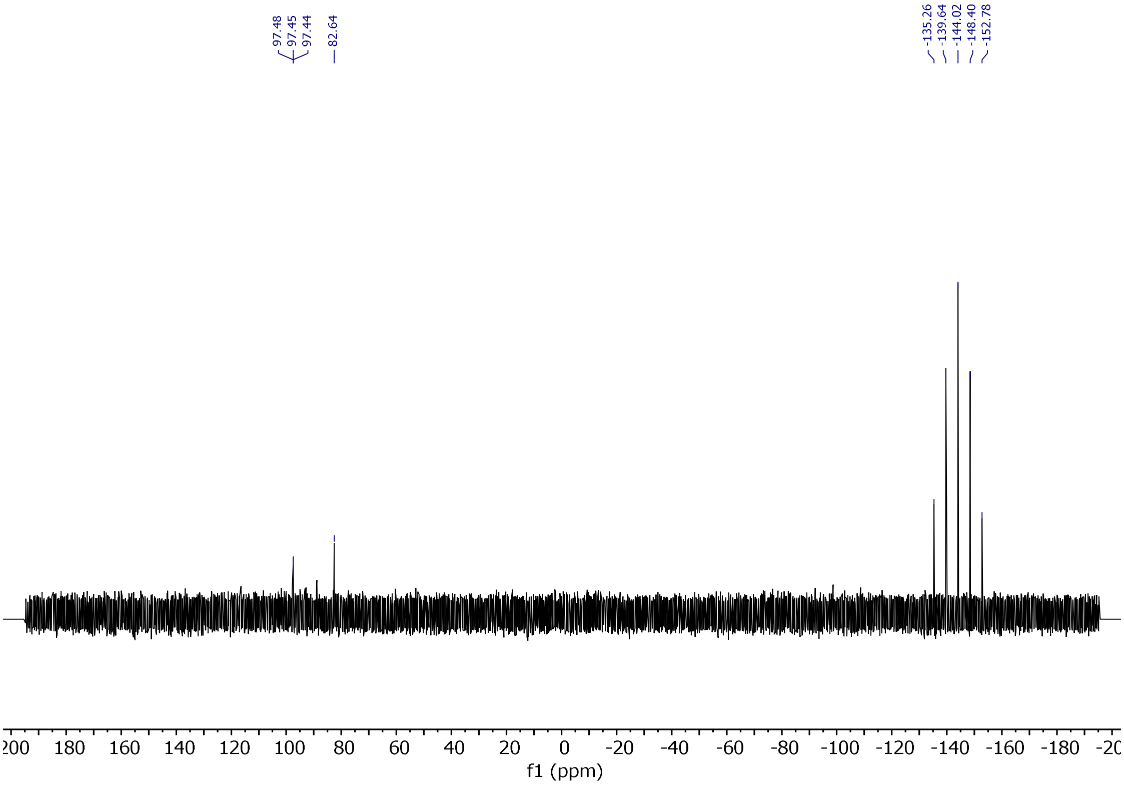

Figure S138. The ^{31}P NMR spectrum after the reaction between **1** and BnK (crude mixture, THF, 298 K).

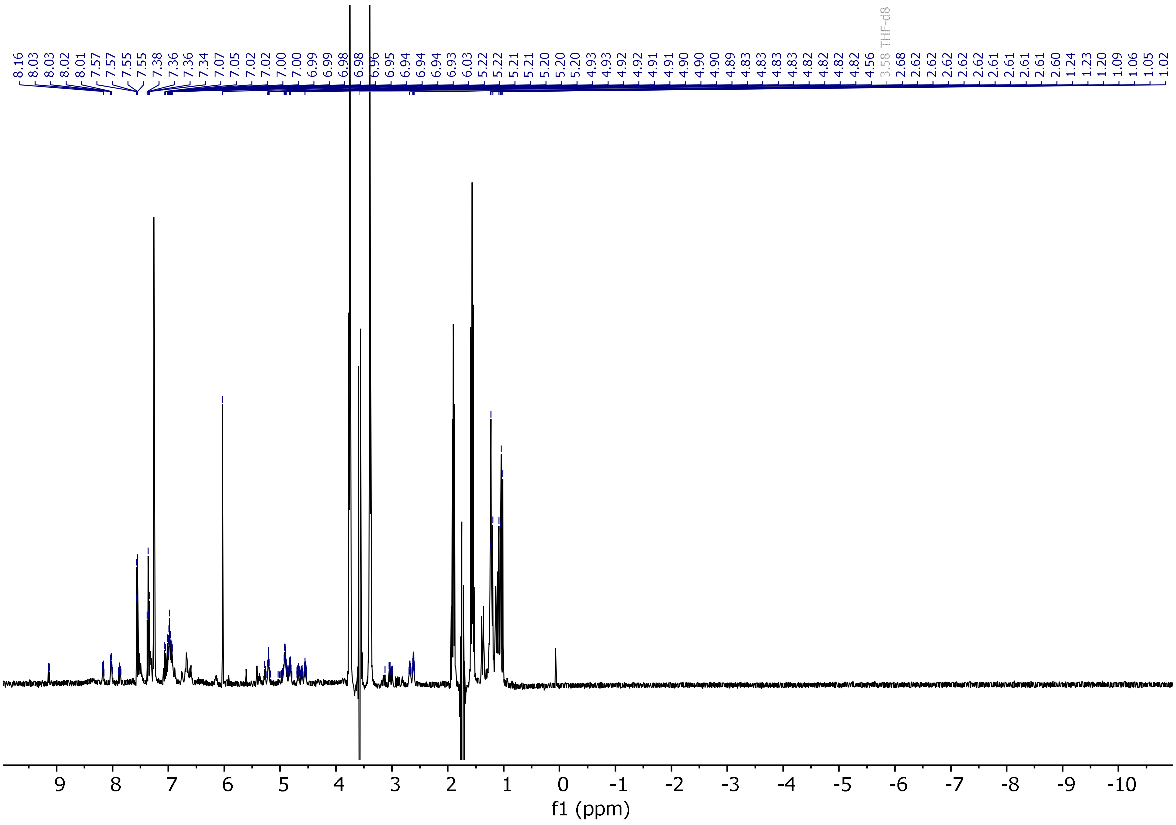

Figure S139. The ^{1}H NMR spectrum after the reaction between **1** and PhMgBr (crude mixture, THF, 298 K, “PRESAT” function was used for solvent signals suppression).

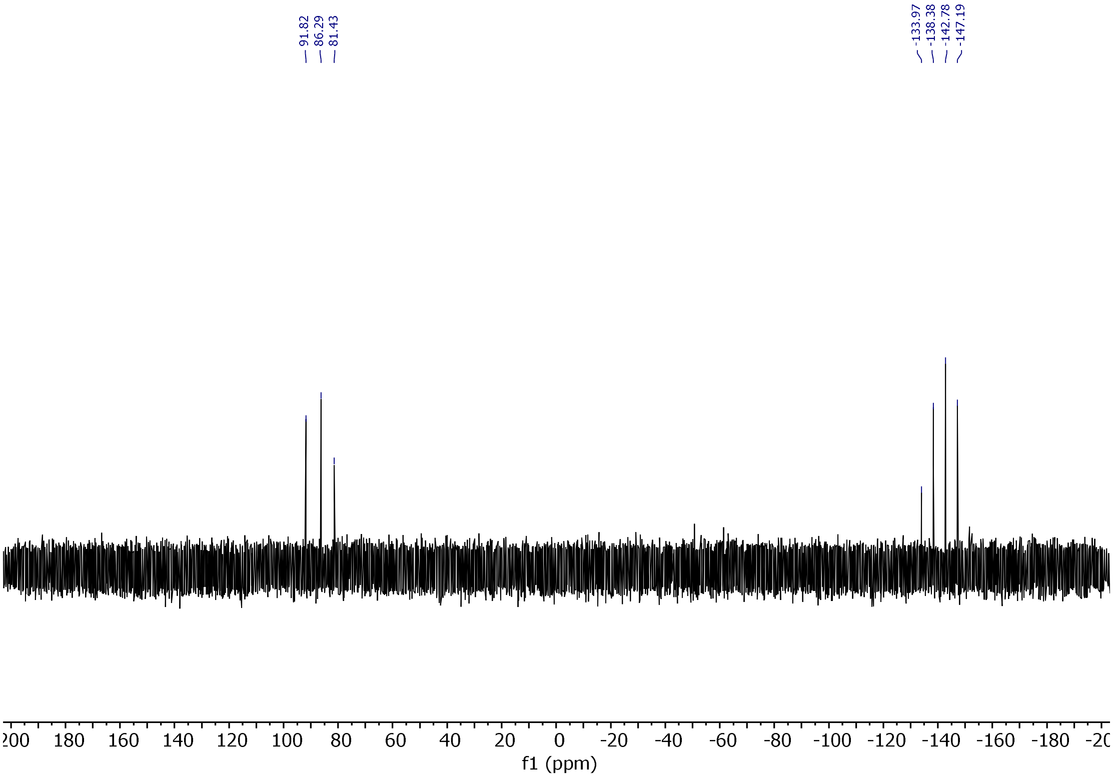

Figure S140. The ^{31}P NMR spectrum after the reaction between **1** and PhMgBr (crude mixture, THF, 298 K).

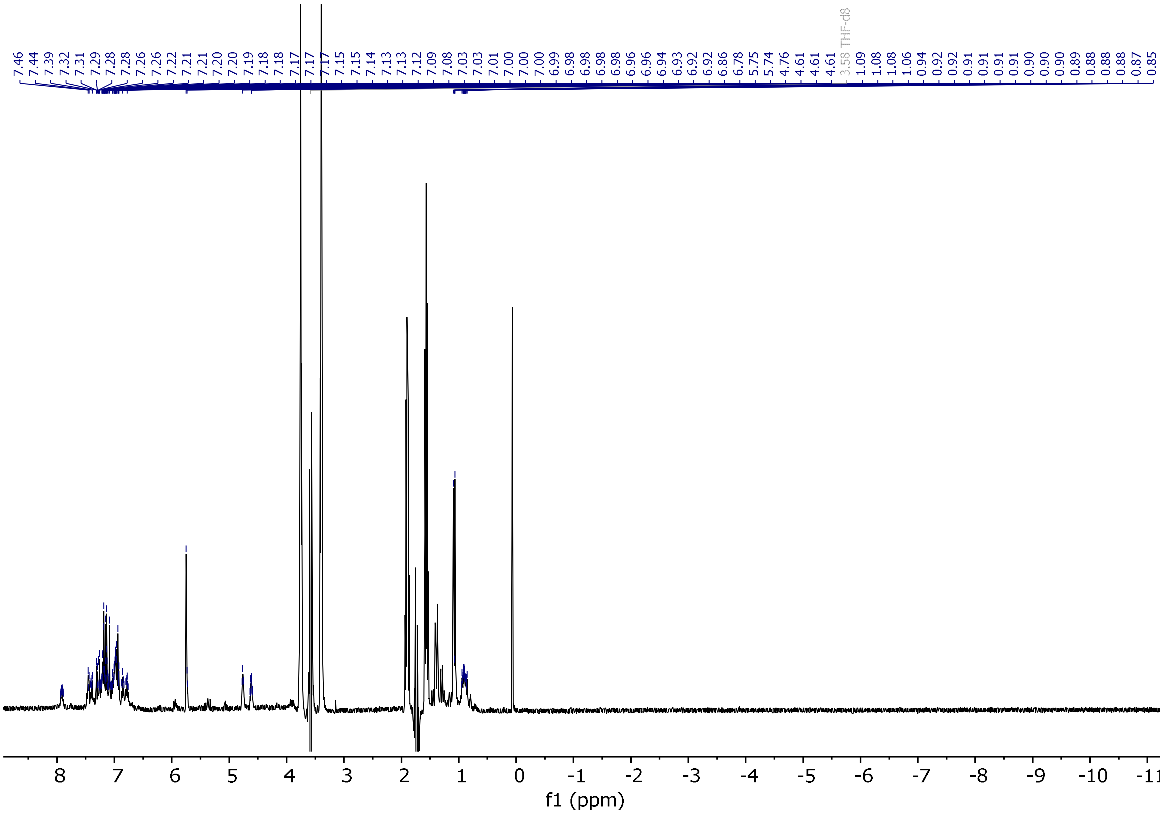

Figure S141. The ^{1}H NMR spectrum after the reaction between **1** and PhCCLi (crude mixture, THF, 298 K, "PRESAT" function was used for solvent signals suppression).

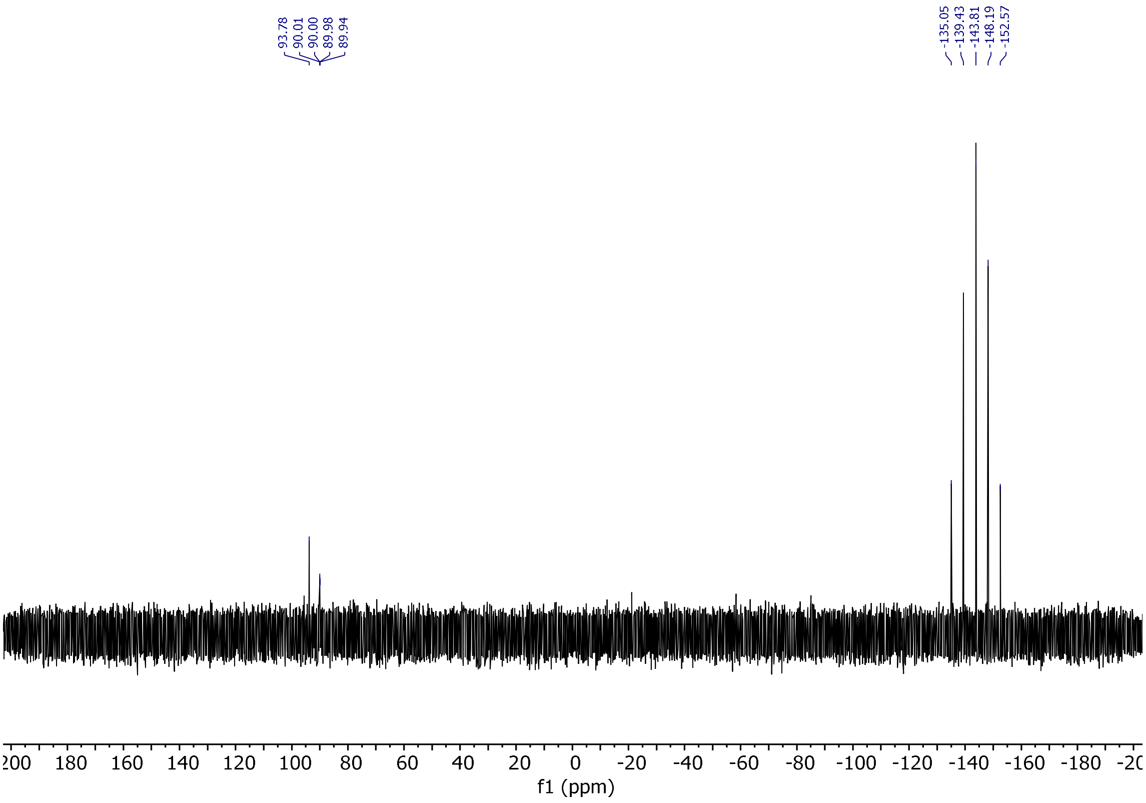

Figure S142. The ^{31}P NMR spectrum after the reaction between **1** and PhCCLi (crude mixture, THF, 298 K).

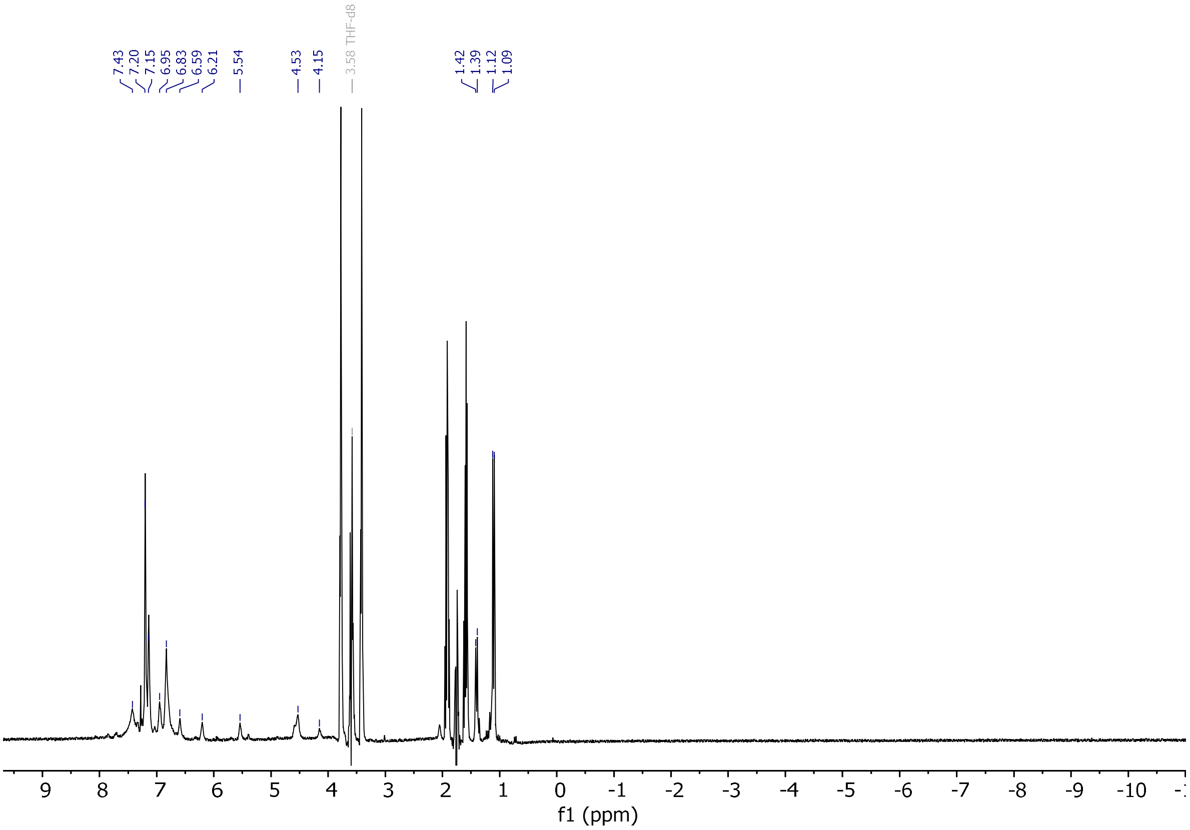

Figure S143. The ^{1}H NMR spectrum after the reaction between **1** and $KPPh_2$ (crude mixture, THF, 298 K, "PRESAT" function was used for solvent signals suppression).

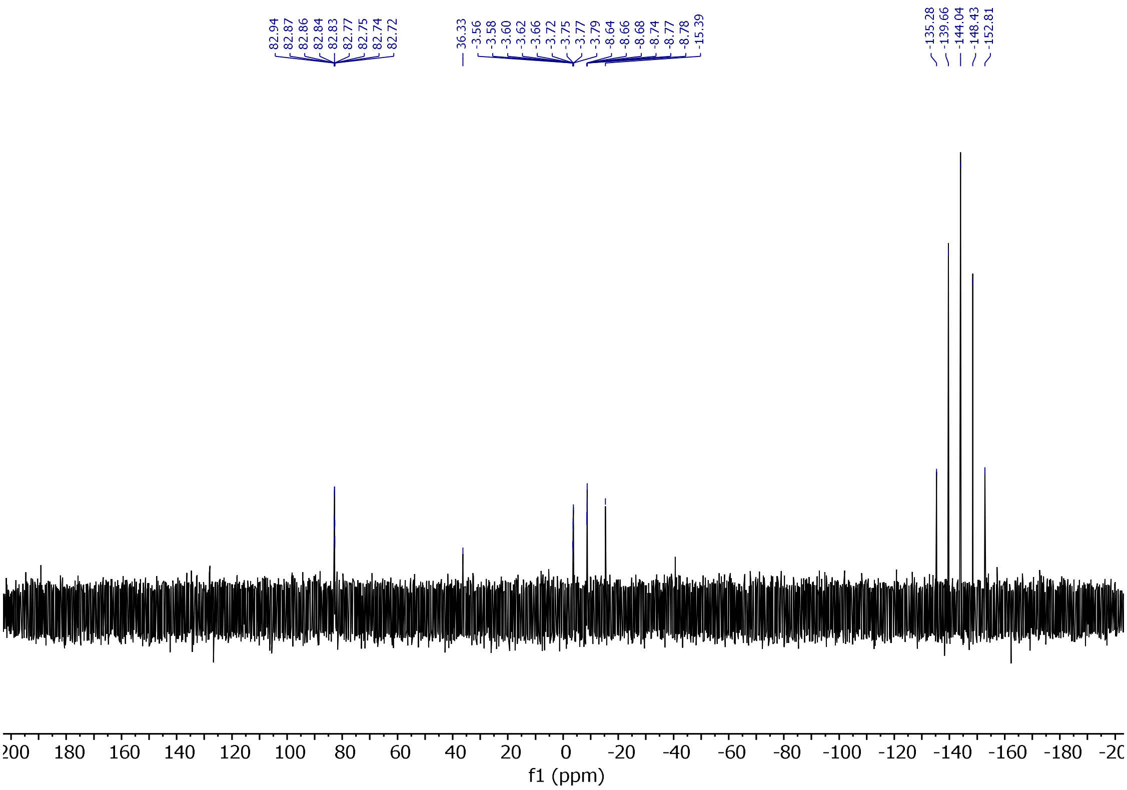

Figure S144. The ^{31}P NMR spectrum after the reaction between **1** and $KPPh_2$ (crude mixture, THF, 298 K).

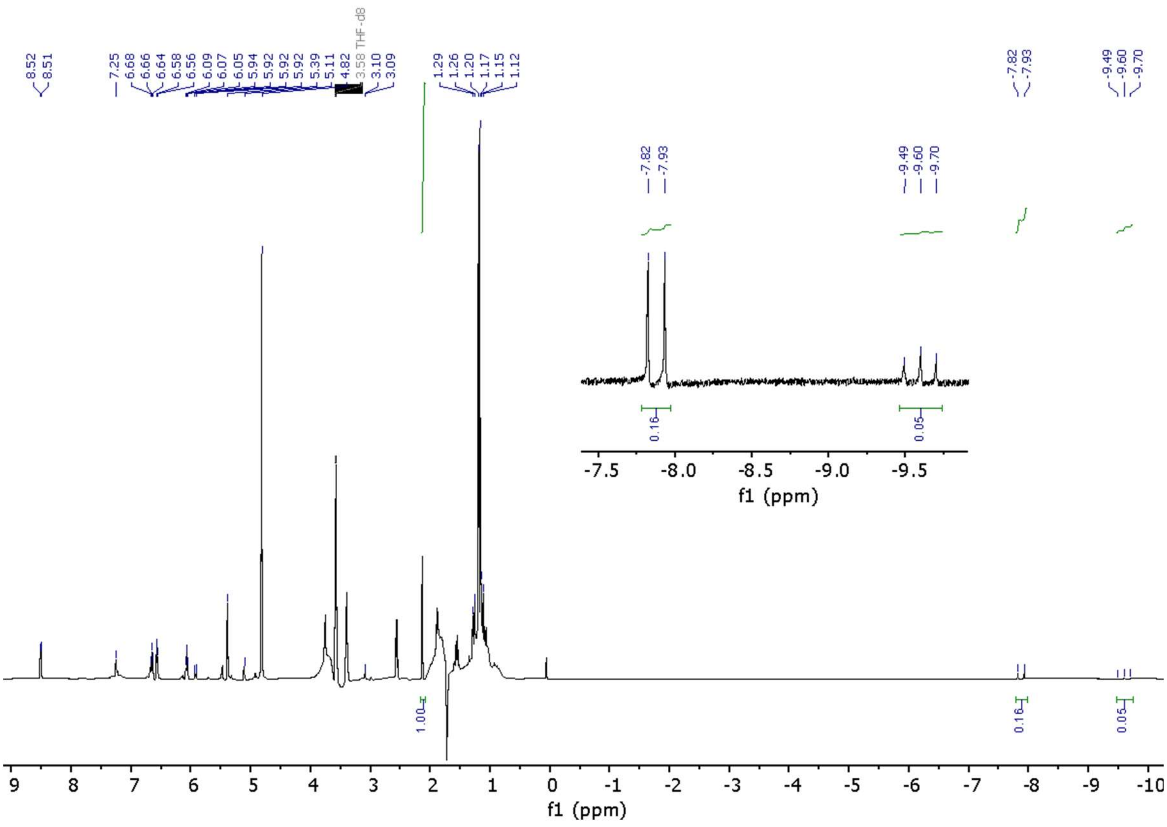

Figure S145. The ^{1}H PRESAT qNMR spectrum after the reaction between **1** and VinMgBr (crude mixture, internal standard (hexamethylbenzene), THF, 298 K).

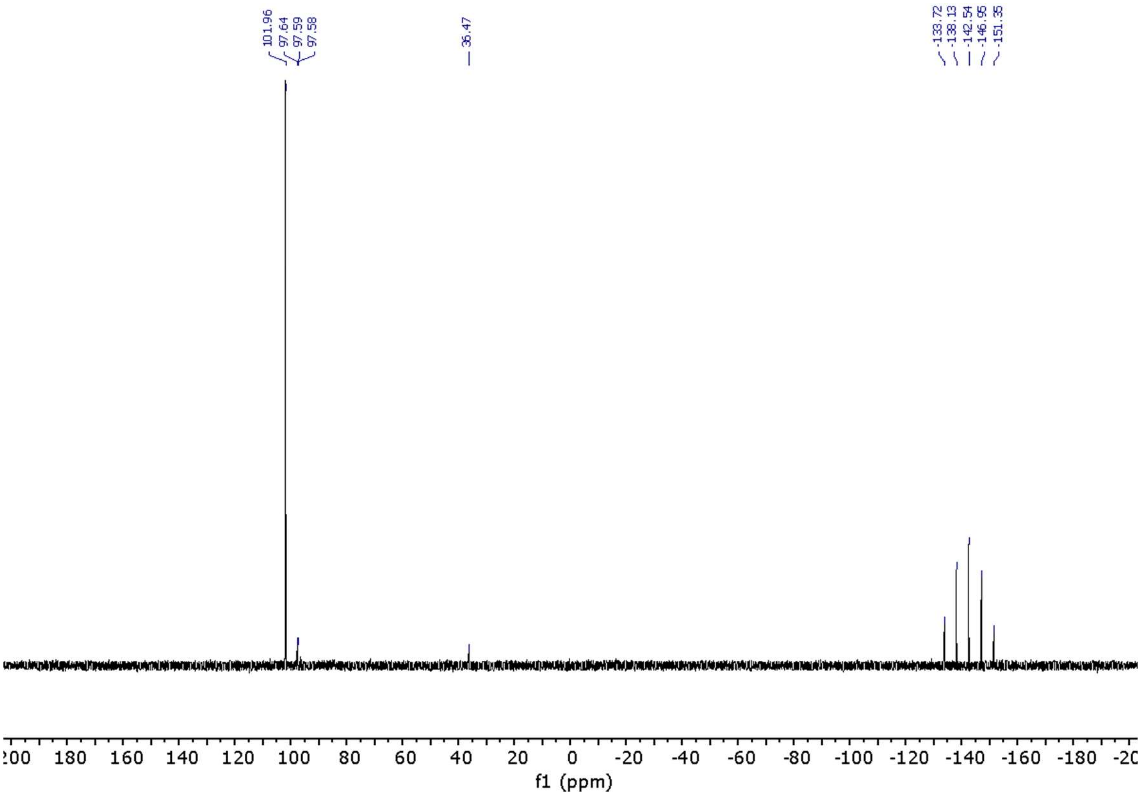

Figure S146. The ^{31}P NMR spectrum after the reaction between **1** and VinMgBr (crude mixture, internal standard (hexamethylbenzene), THF, 298 K).

Type	Nucleophile	Product	Yield, %
N centered	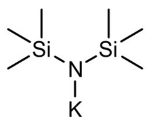	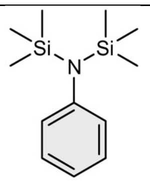	40
P centered	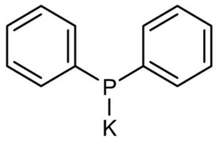	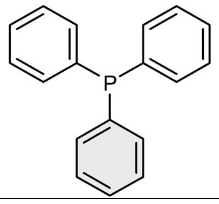	6
O centered	KOH	Crude NMR showed some unidentified hydride species.	–
S centered	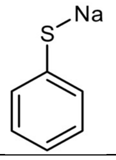	Substitution of the benzene with thiolate ligands	–
C_{sp3} centered	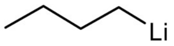	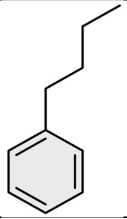	9
	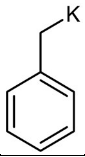	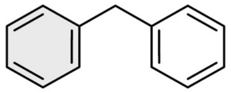	31
C_{sp2} centered	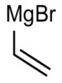	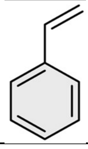	21**
	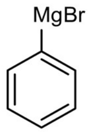	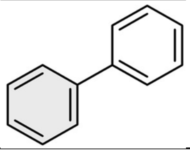	35
C_{sp} centered	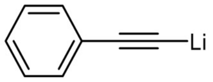	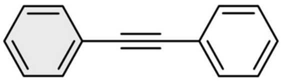	4

Table S4. The scope of the reaction*. The absence of the S_NArH or low yields in the cases of some nucleophiles is probably caused by side reactions (see comments in the table).

The reactions were performed via the standard protocol as described for the synthesis of **4. **The corresponding complex was quantified instead (see above).*

S5 GC Measurements

GC FID was used for quantification of the substituted benzenes and the GC was calibrated using a five-point calibration procedure using nitrobenzene as an internal standard (IS). The following oven temperature program was used for all GC measurements including the calibration ones.

**Initial temperature**	70 °C for 2 min
**Ramp 1**	25 deg/min to 190 °C, then hold for 2 min
**Ramp 2**	30 deg/min to 275 °C, then hold for 5 min

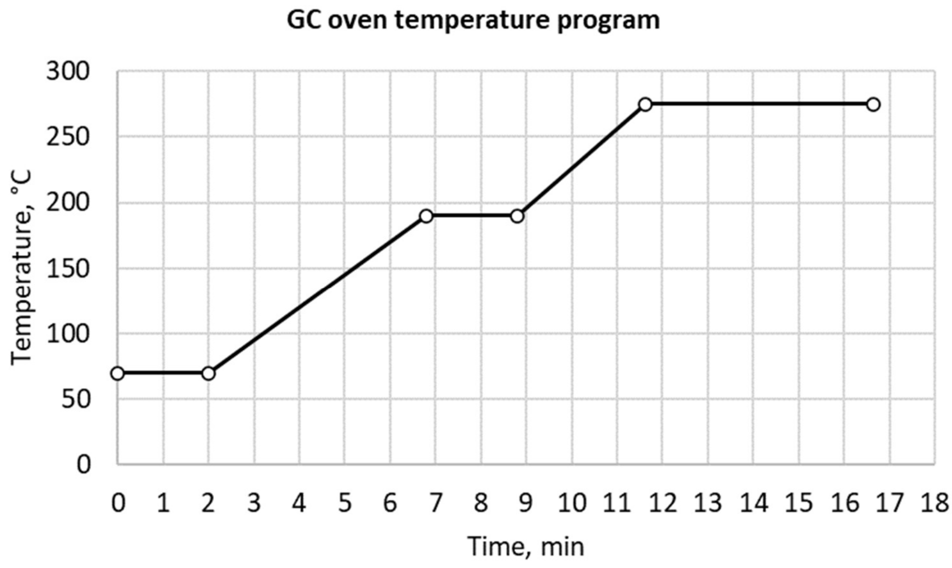

Figure S147. The GC oven program is used for the quantification of the S_NArH products.

S5.1 GC calibration curves

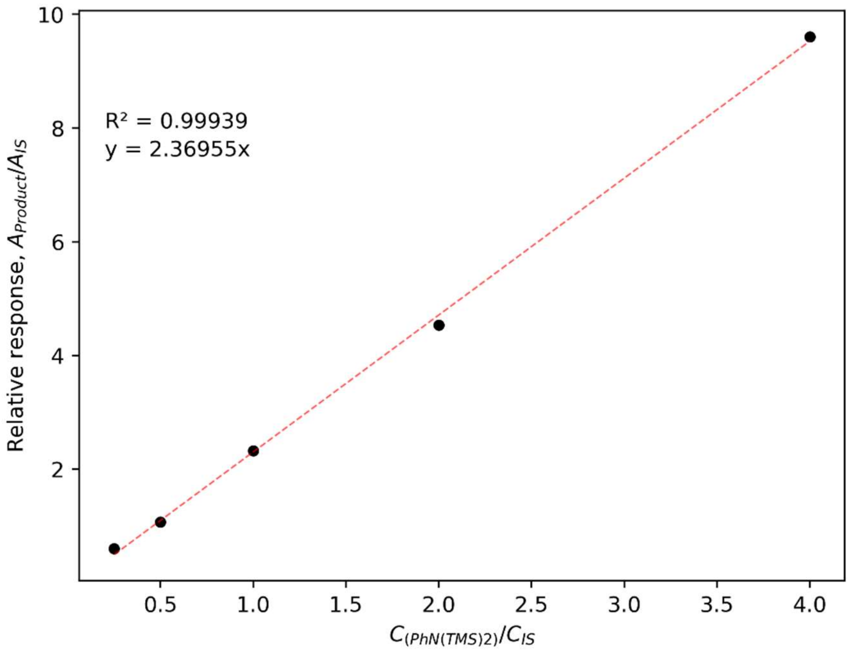

Figure S148. Calibration curve for quantification of **N,N-bis(trimethylsilyl)aniline** via GC-FID analysis.

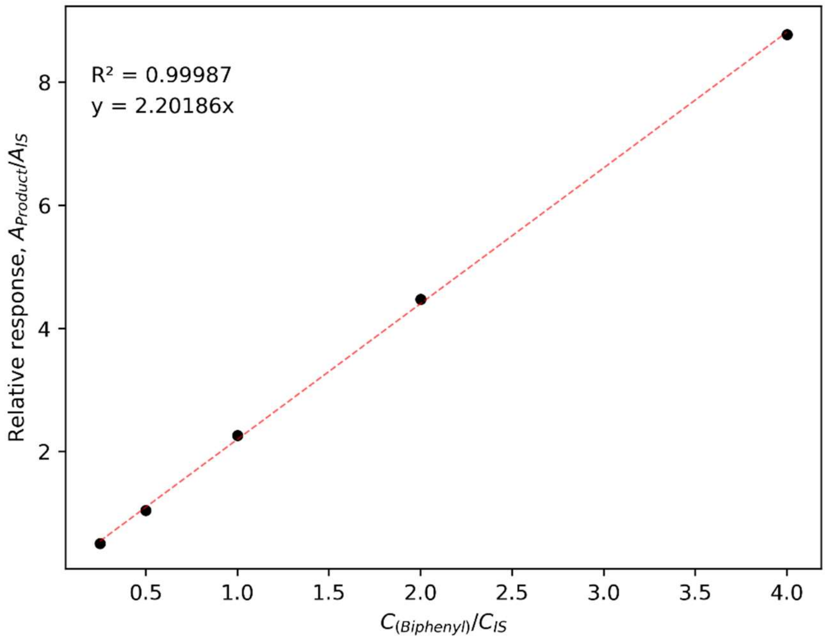

Figure S149. Calibration curve for quantification of **biphenyl** via GC-FID analysis.

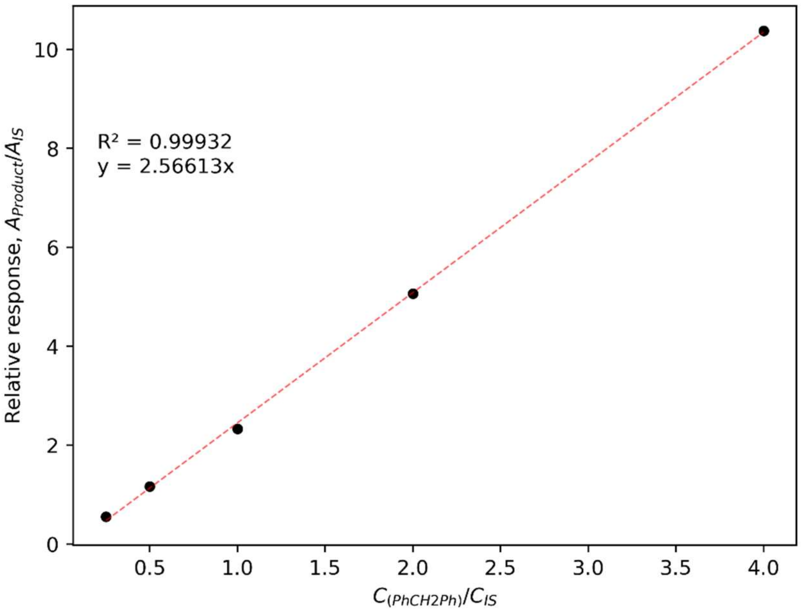

Figure S150. Calibration curve for quantification of **diphenylmethane** via GC-FID analysis.

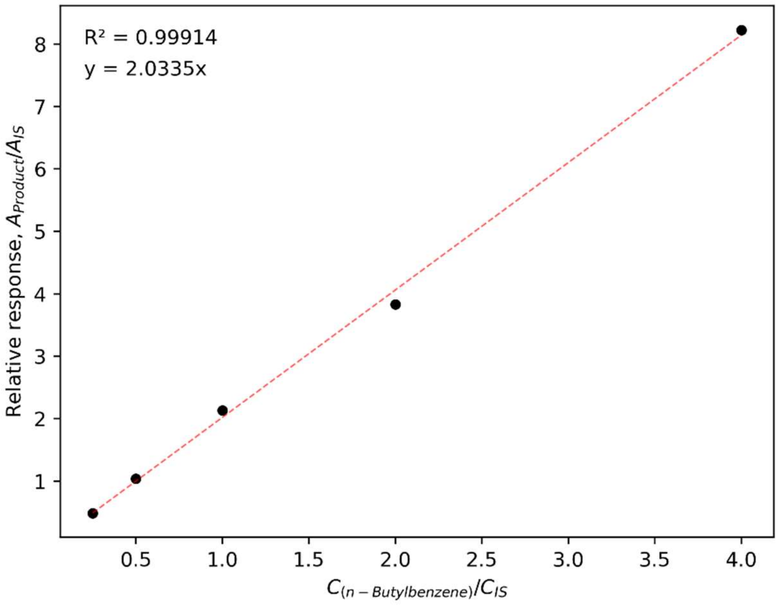

Figure S151. Calibration curve for quantification of ***n*****-butylbenzene** via GC-FID analysis.

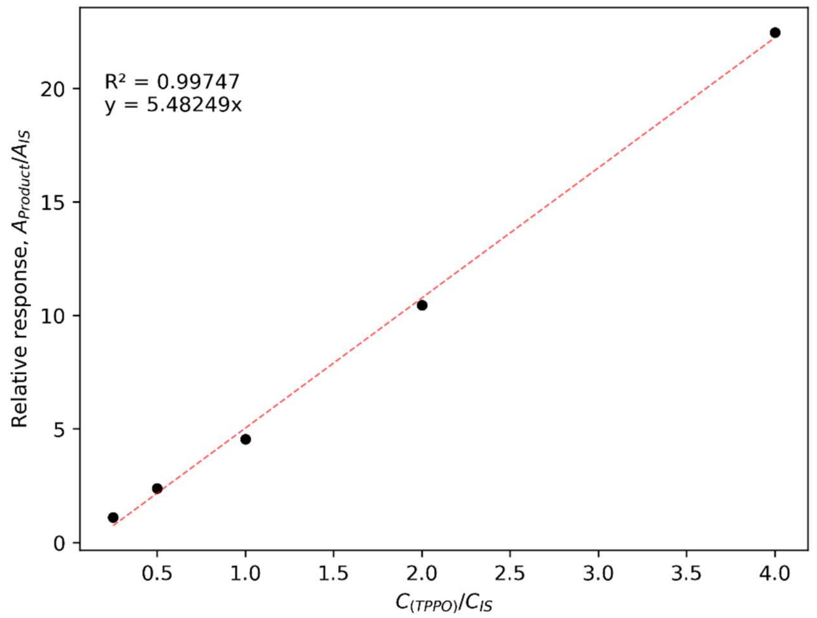

Figure S152. Calibration curve for quantification of **triphenylphosphine oxide** via GC-FID analysis.

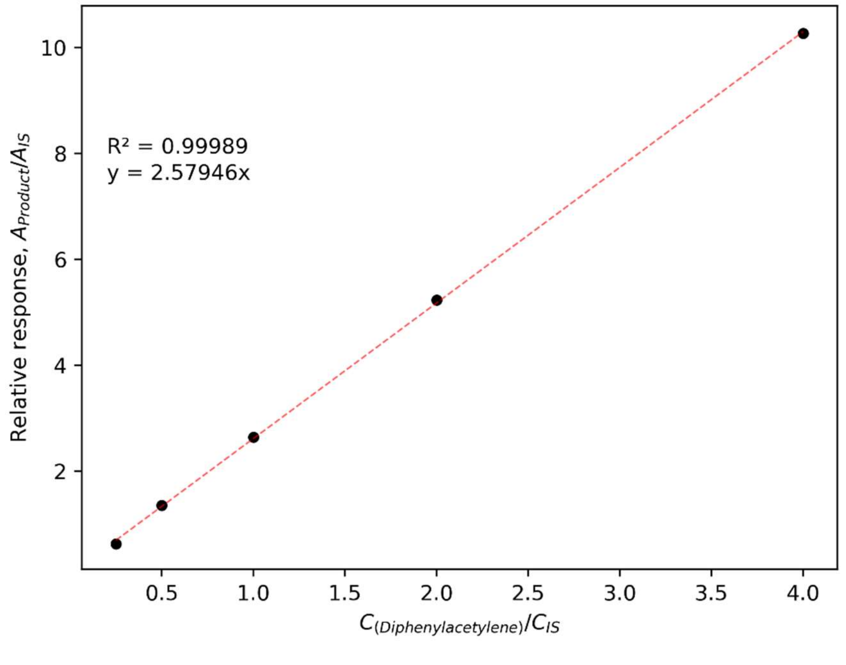

Figure S153. Calibration curve for quantification of **diphenylacetylene** via GC-FID analysis.

The script used for the GC calibration calculations as well as plotting the graphs (Python 3.11.4)

```
import matplotlib.pyplot as plt
from statsmodels.formula.api import ols
import numpy as np
import pandas as pd
import seaborn as sns

GC_data = [[21789.74, 209306.28],           #raw GC data in the format (IS, Product)
        [22107.86, 100275.62],
        [21184.42, 49173.86],
        [20958.85, 22454.87],
        [18556.38, 11113.20]]

name = 'PhN(TMS)2'              #name of the compound

cal_entry = [1, 2, 3, 4, 5]                 #number of entry
cal_ratios = [i[1]/i[0] for i in GC_data]                #ratios Product/IS

conc = [4]              #concentrations of the product
for i in range(4):
   a = 4
   conc.append(conc[-1]/2)

data_cal = pd.DataFrame(np.array([cal_entry, cal_ratios, conc]).T, columns=['entry', 'ratios', 'conc'])
R = ols("ratios ~ conc -1", data = data_cal).fit()

print("Interception: ", R.params[0])
print("R^2: ",R.rsquared)


ax = sns.lineplot(data=data_cal, x="conc", y='ratios', errorbar='sd', linestyle='', err_style='bars', marker='o', 
color='black')

sns.regplot(data=data_cal, x="conc", y='ratios', scatter=False, ax=ax, ci = False, line_kws = {"color": "red", 
"alpha": 0.6, 'linewidth': .8}).lines[2].set_linestyle("--")

ax.text(0.2, 8, "R\u00b2 = " + str(round(R.rsquared,5)))
ax.text(0.2, 7.5, "y = " + str(round(R.params[0],5)) + "x")
plt.xlabel('$C_{(' + name + ')} / C_{IS}$')
plt.ylabel('Relative response, $A_{Product} / A_{IS}$')
plt.savefig(name + '.png', dpi=400)
sns.despine()
plt.show()
```

S5.2 GC chromatograms of the products

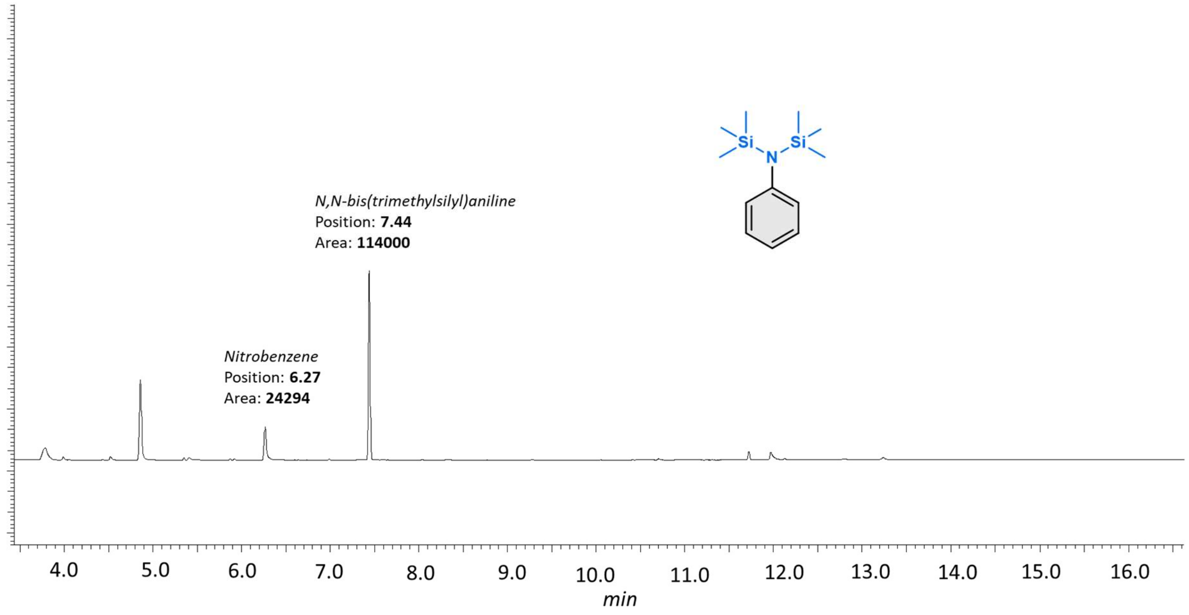

Figure S154. The GC chromatogram for **N,N-bis(trimethylsilyl)aniline**.

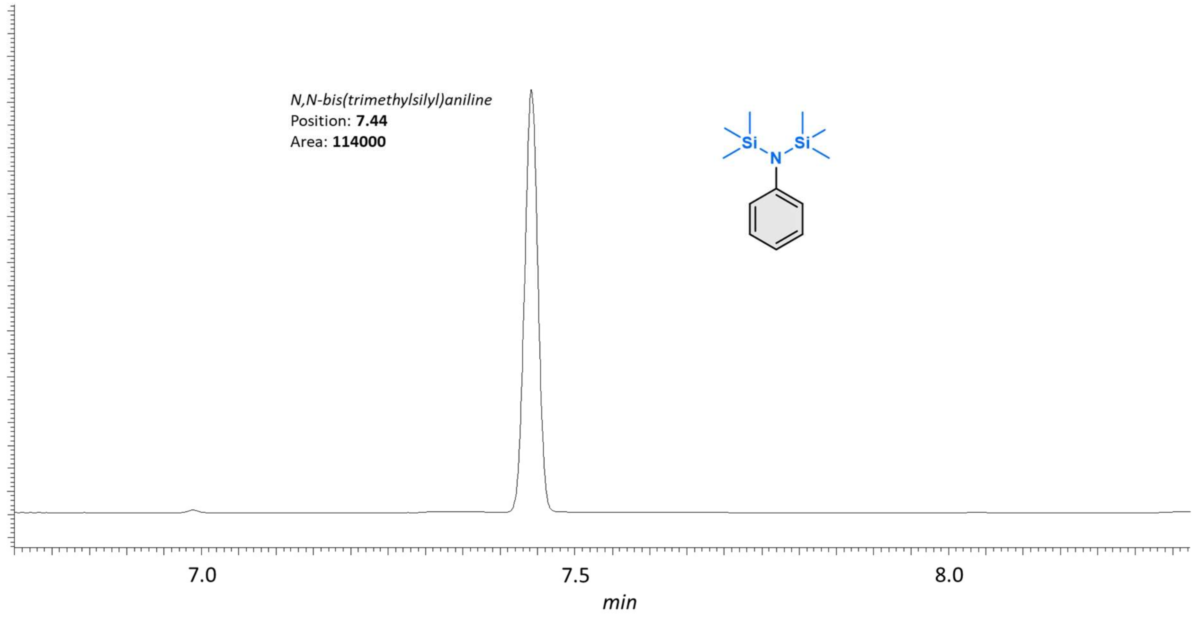

Figure S155. The GC chromatogram for **N,N-bis(trimethylsilyl)aniline** (zoomed in).

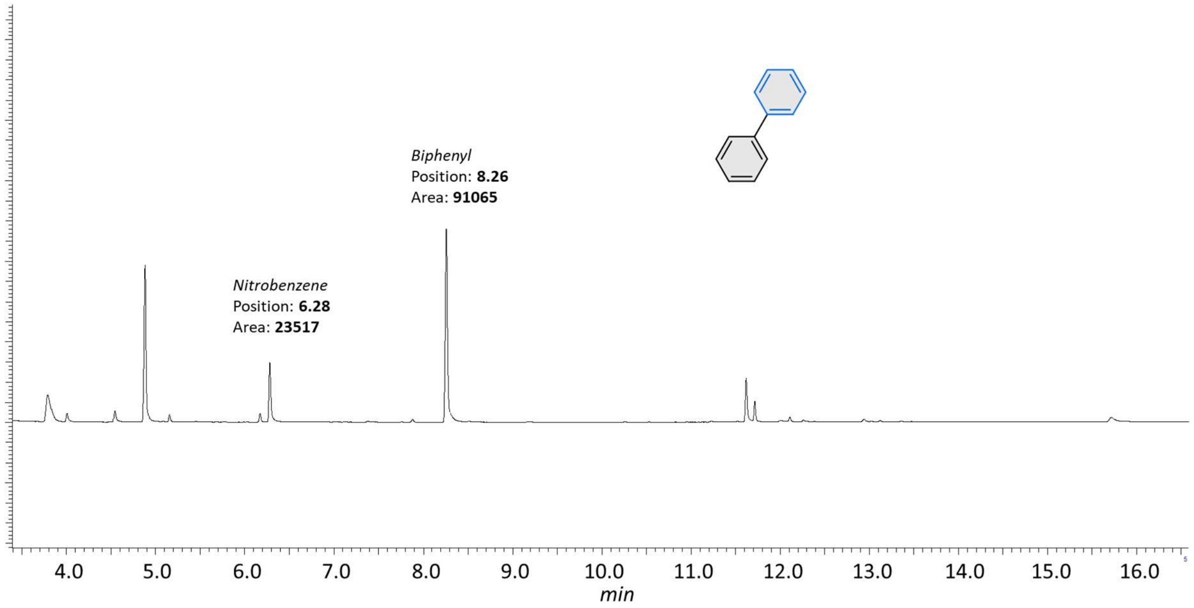

Figure S156. The GC chromatogram for **biphenyl** (traces of *o*-terphenyl were detected).

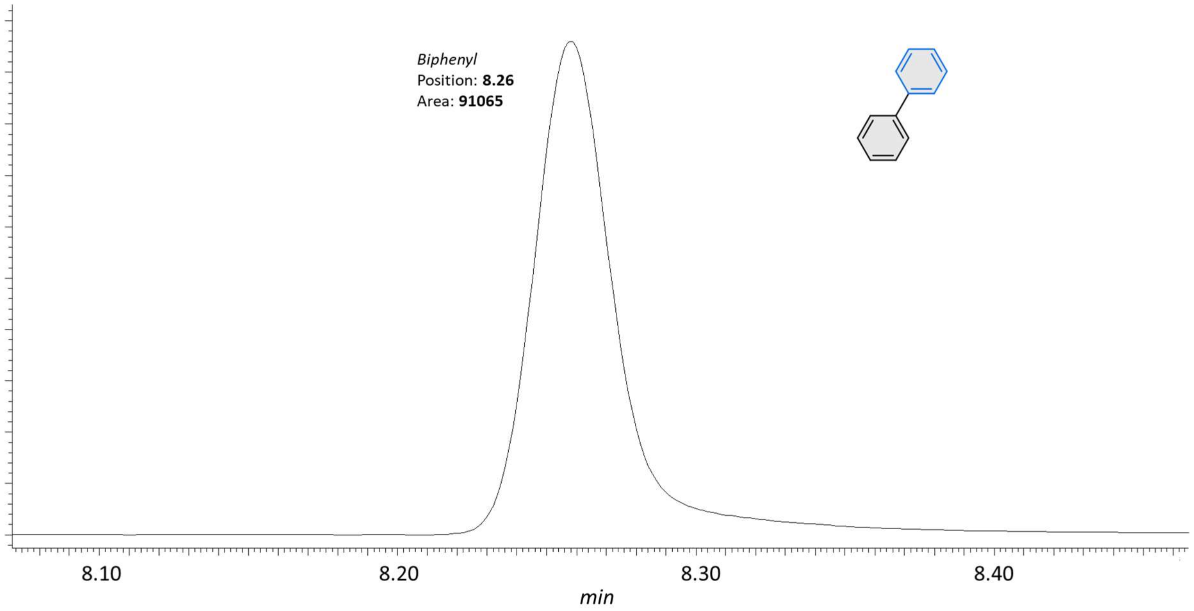

Figure S157. The GC chromatogram for **biphenyl** (zoomed in).

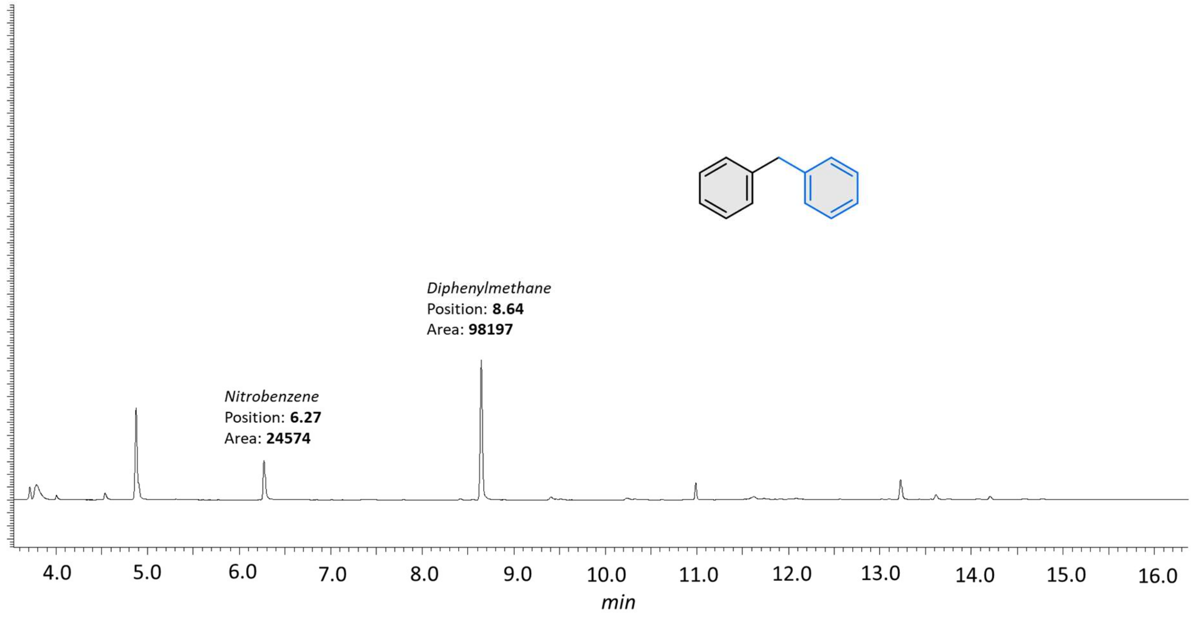

Figure S158. The GC chromatogram for **diphenylmethane** (traces of dibenzylbenzenes were detected).

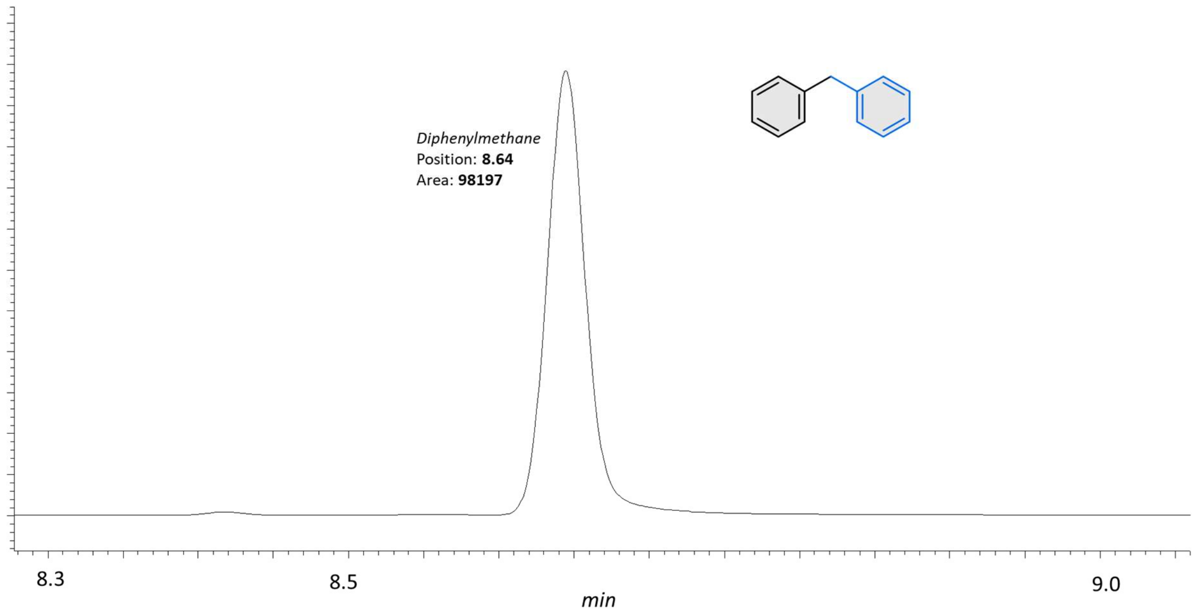

Figure S159. The GC chromatogram for **diphenylmethane** (zoomed in).

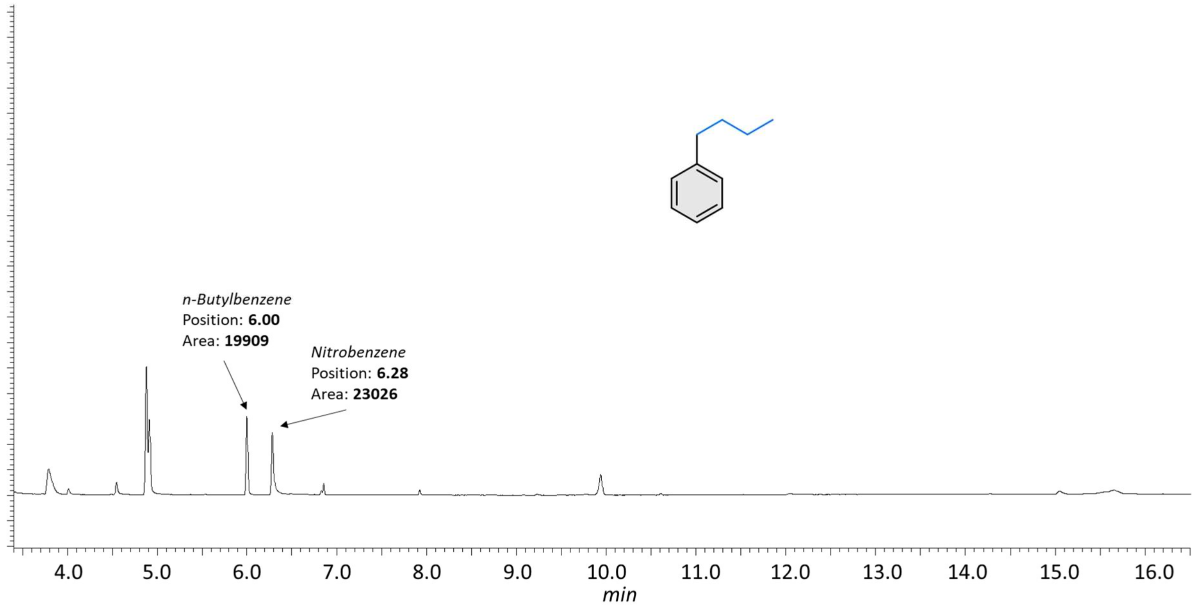

Figure S160. The GC chromatogram for ***n*-butylbenzene** (traces of dibutylbenzenes were detected).

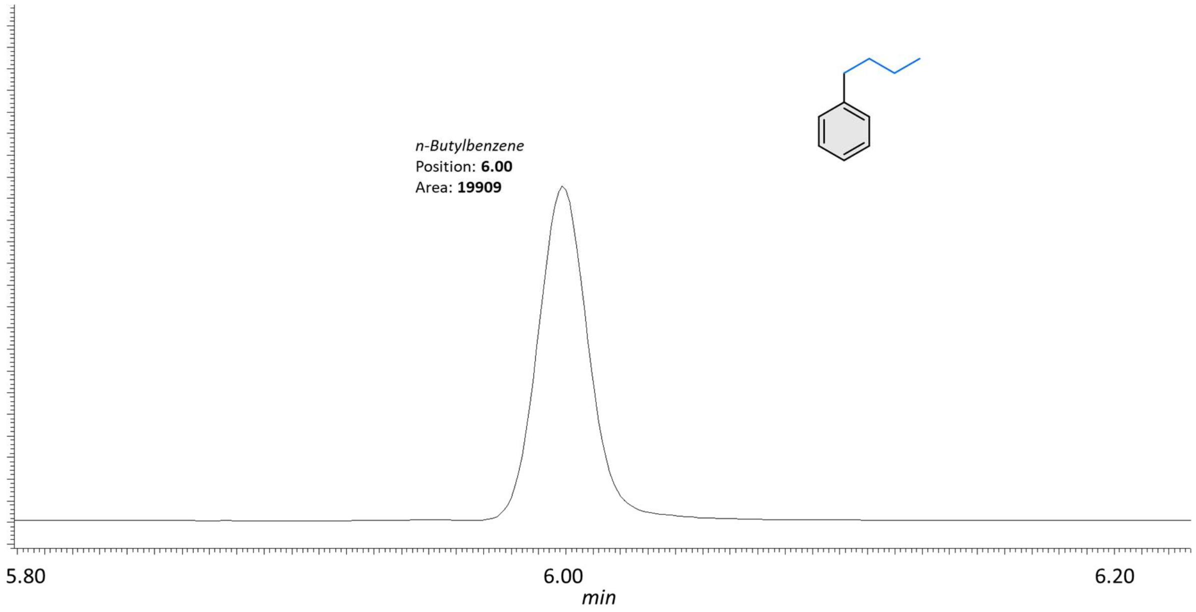

Figure S161. The GC chromatogram for ***n*-butylbenzene** (zoomed in).

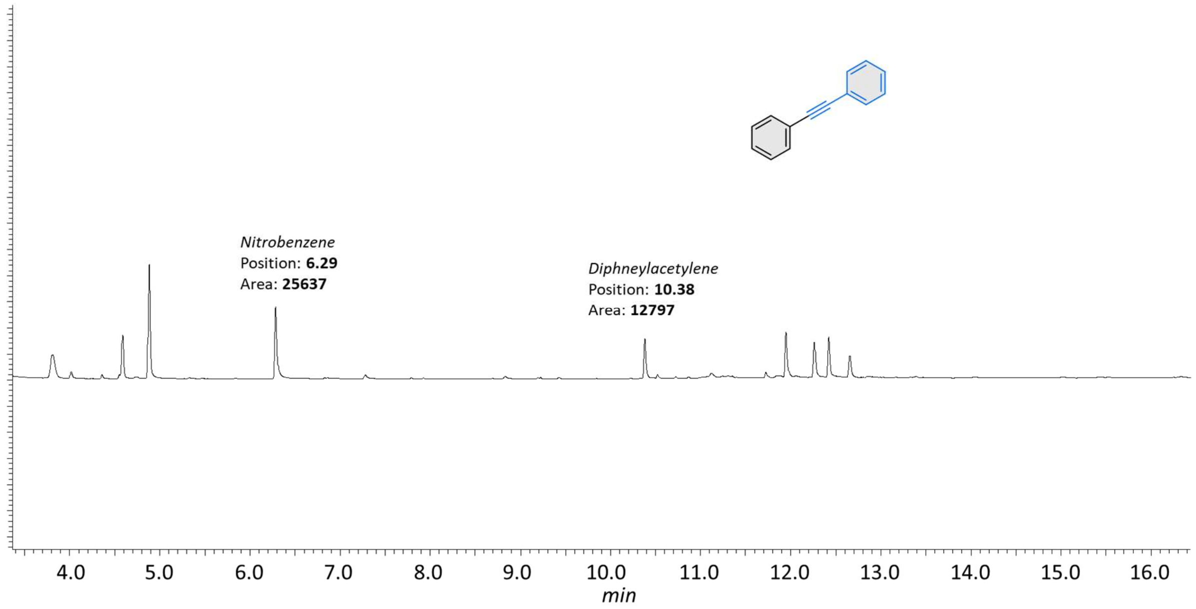

Figure S162. The GC chromatogram for **diphenylacetylene**.

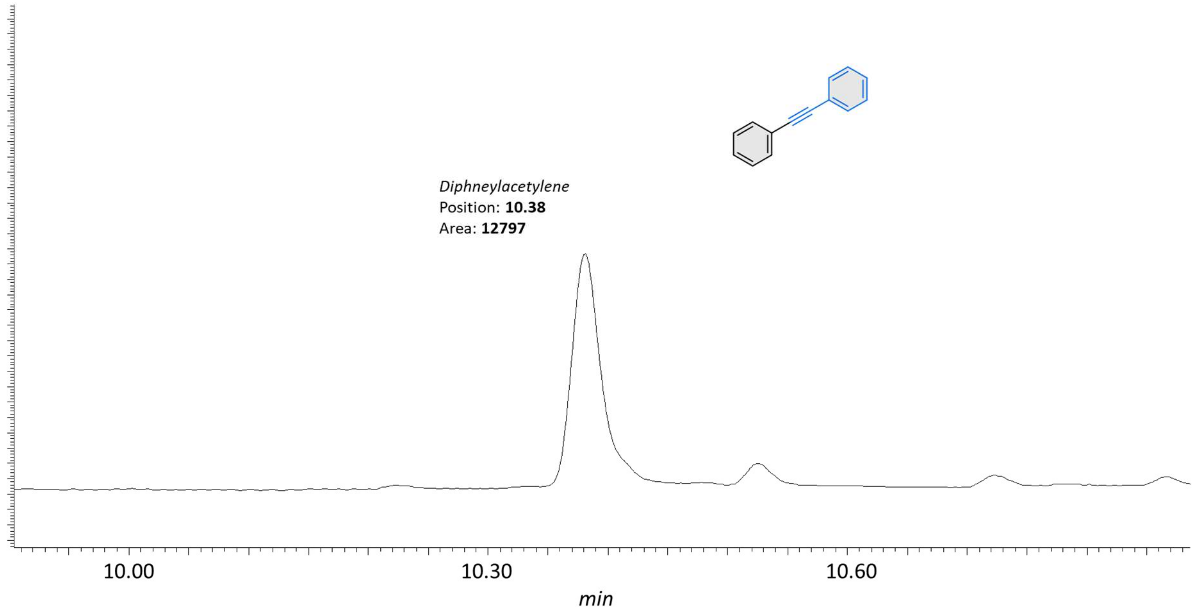

Figure S163. The GC chromatogram for **diphenylacetylene** (zoomed in).

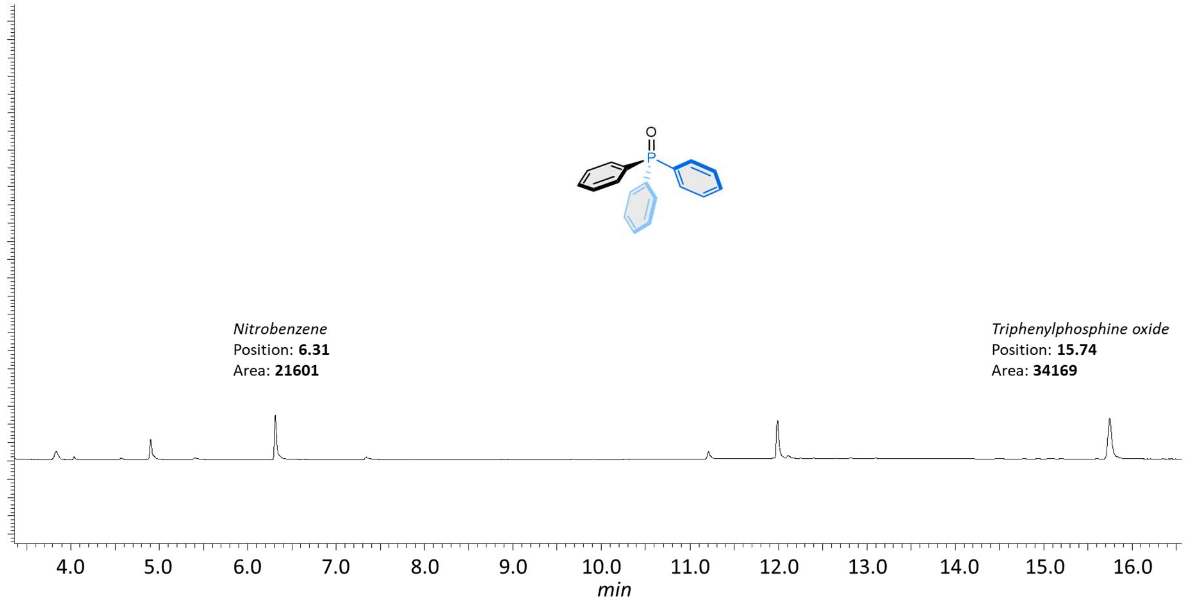

Figure S164. The GC chromatogram for **triphenylphosphine oxide**.

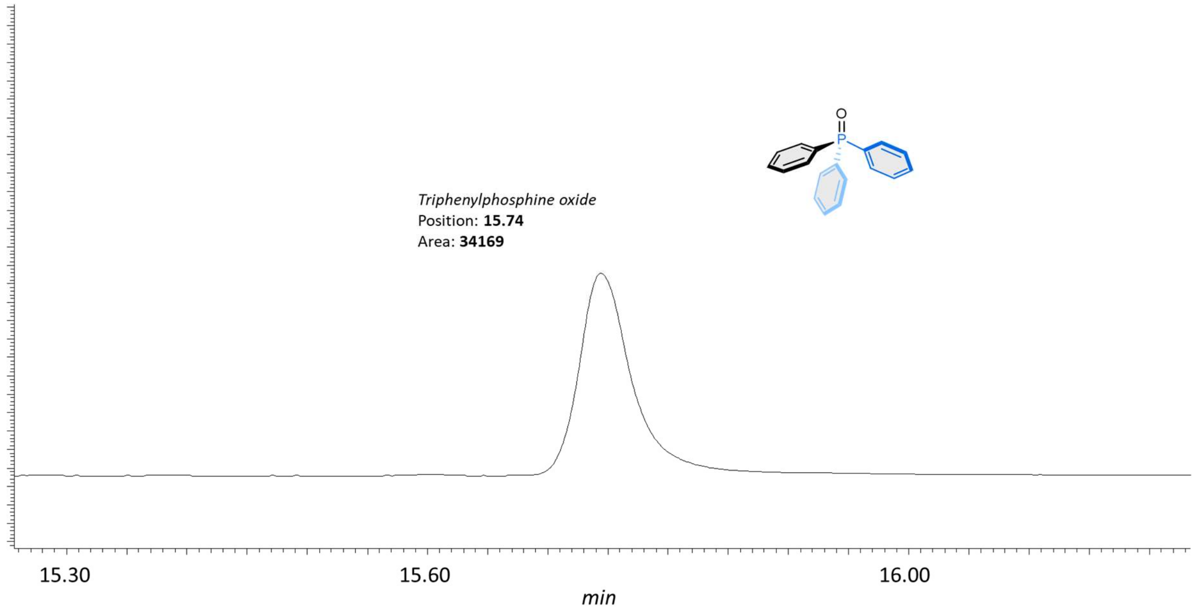

Figure S165. The GC chromatogram for **triphenylphosphine oxide** (zoomed in).

S6 Crystal Structures

S6.1 X-ray crystal structure determination of **1**

$[C_{20}H_{30}ClNPRu](PF_6) \cdot CH_2Cl_2$, Fw = 681.83, orange needle, 0.58 · 0.12 · 0.07 mm^3, monoclinic, $P2_1/c$ (no. 14), a = 8.7180(4), b = 18.4723(8), c = 16.8326(6) Å, β = 91.306(2) , V = 2710.1(2) $Å^3$, Z = 4, D_x = 1.671 g/cm^3, μ = 1.05 mm^{-1}. The diffraction experiment was performed on a Bruker Kappa ApexII diffractometer with a sealed tube and Triumph monochromator (λ = 0.71073 Å) at a temperature of 150(2) K up to a resolution of $(\sin \theta/\lambda)_{max}$ = 0.65 $Å^{-1}$. Intensity integration was performed using the Eval15 software[36]. A numerical absorption correction and scaling were performed with SADABS[37] (correction range 0.57-0.94). A total of 62917 reflections were measured, 6236 reflections were unique (R_{int} = 0.049), and 5088 reflections were observed [$I>2\sigma(I)$]. The structure was solved with Patterson superposition methods using SHELXT.[38] Structure refinement was performed with SHELXL-2018[39] on F^2 of all reflections. Non-hydrogen atoms were refined freely with anisotropic displacement parameters. The dichloromethane molecule was refined with a disorder model. Hydrogen atoms of the metal complex were located in difference Fourier maps. Hydrogen atoms of the solvent were introduced in calculated positions. Hydrogen atoms of the coordinated benzene ligand were refined freely with isotropic displacement parameters. All other hydrogen atoms were refined with a riding model. 365 Parameters were refined with 57 restraints (concerning geometry and displacement parameters in the dichloromethane molecule). R1/wR2 [$I > 2\sigma(I)$]: 0.0238 / 0.0552. R1/wR2 [all refl.]: 0.0326 / 0.0568. S = 1.096. Residual electron density between –0.42 and 0.61 $e/Å^3$. Geometry calculations and checking for higher symmetry were performed with the PLATON program.[40]

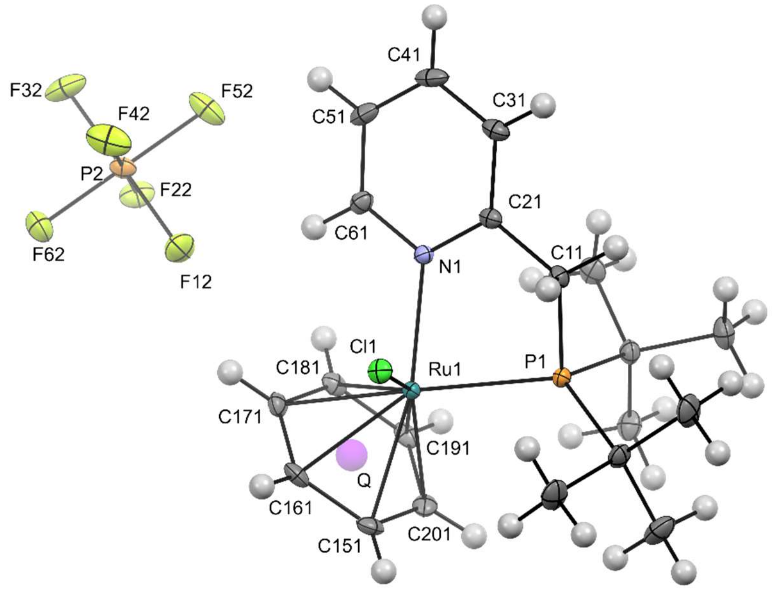

Figure S166. The molecular structure of compound **2** (ellipsoids drawn at the 30% probability level). A disordered DCM molecule is omitted for clarity. The hydrogens are drawn as fixed-size spheres of 0.2 Å radius. Q is the centroid of the benzene ring.

Distance	Å	Distance	Å
Ru(1)-C(151)	2.2182(19)	Ru(1)-P(1)	2.3785(5)
Ru(1)-C(161)	2.2242(19)	P(1)-C(11)	1.8385(19)
Ru(1)-C(171)	2.2406(19)	C(11)-C(21)	1.501(3)
Ru(1)-C(181)	2.2051(19)	C(21)-N(1)	1.360(2)
Ru(1)-C(191)	2.2173(19)	N(1)-C(61)	1.351(2)
Ru(1)-C(201)	2.2038(19)	C(61)-C(51)	1.384(3)
Ru(1)-Q	1.7158(9)	C(51)-C(41)	1.375(3)
Ru(1)-Cl(1)	2.4200(5)	C(41)-C(31)	1.378(3)
Ru(1)-N(1)	2.1244(15)	C(31)-C(21)	1.392(2)
Ru ring slippage*	0.023	C(151)-C(161)	1.413(3)
		C(161)-C(171)	1.392(3)
		C(171)-C(181)	1.419(3)
		C(181)-C(191)	1.399(3)
		C(191)-C(201)	1.410(3)
		C(201)-C(151)	1.404(3)

Table S5. Selected distances in the X-ray crystal structure of **1**. *Distance between perpendicular projection of Ru on the benzene ring L.S.-plane and ring centroid.

Angle	°	Angle	°
Ru(1)-Q-C(151)	89.78(9)	N(1)-Ru(1)-P(1)	79.54(4)
Ru(1)-Q-C(161)	90.73(9)	N(1)-Ru(1)-Cl(1)	80.62(4)
Ru(1)-Q-C(171)	90.85(9)	N(1)-Ru(1)-Q	132.03(5)
Ru(1)-Q-C(181)	89.46(9)	P(1)-Ru(1)-Q	132.66(4)
Ru(1)-Q-C(191)	89.94(9)	Cl(1)-Ru(1)-Q	122.93(3)
Ru(1)-Q-C(201)	89.23(9)	P(1)-Ru(1)-Cl(1)	92.243(17)
N(1)-C(21)-C(11)-P(1)	24.4(2)	(Ru(1)-Q)-(normal of benzene)	0.70(7)

Table S6. Selected angles and torsion angles in the X-ray crystal structure of **1**.

S6.2 X-ray crystal structure determination of **4**

X-ray crystal structure determination of ***4***

$C_{26}H_{47}N_2PRuSi_2$, Fw = 575.87, orange block, 0.41 · 0.40 · 0.17 mm^3, monoclinic, $P2_1/c$ (no. 14), a = 13.9298(4), b = 16.2626(4), c = 13.8275(4) Å, β = 112.534(2) , V = 2893.25(15) Å^3, Z = 4, D_x = 1.322 g/cm^3, μ = 0.70 mm^{-1}. The diffraction experiment was performed on a Bruker Kappa ApexII diffractometer with a sealed tube and Triumph monochromator (λ = 0.71073 Å) at a temperature of 150(2) K up to a resolution of $(\sin \theta/\lambda)_{max}$ = 0.65 Å^{-1}. The crystal was broken into several fragments. Two orientation matrices were used for the intensity integration of the major fragments using the Eval15 software[36]. Only the non-overlapping reflections were used for structure solution and refinement. A multi-scan absorption correction and scaling were performed with SADABS[37] (correction range 0.68-0.75). A total of 41381 reflections were measured, 6635 reflections were unique (R_{int} = 0.021), and 6179 reflections were observed [I>2 σ(I)]. The structure was solved with Patterson superposition methods using SHELXT.[38] Structure refinement was performed with SHELXL-2018[39] on F^2 of all reflections. Non-hydrogen atoms were refined freely with anisotropic displacement parameters. All hydrogen atoms were located in difference Fourier maps. Metal-bound hydrogen atom H1 and hydrogens H16-H20 of the coordinated phenyl group were refined freely with isotropic displacement parameters. All other hydrogen atoms were refined with a riding model. 329 Parameters were refined with no restraints. R1/wR2 [I > 2 σ(I)]: 0.0200 / 0.0507. R1/wR2 [all refl.]: 0.0217 / 0.0516. S = 1.045. Residual electron density between –0.42 and 0.49 e/Å^3. Geometry calculations and checking for higher symmetry were performed with the PLATON program.[40]

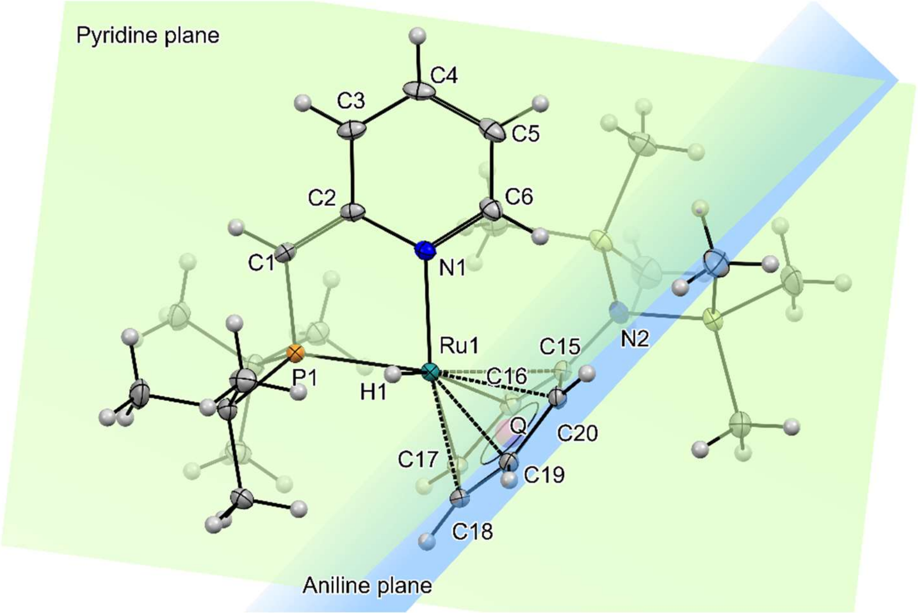

Figure S167. The molecular structure of compound **4** (ellipsoids drawn at the 30% probability level). Two least-squares mean planes and a centroid of the aromatic aniline ring are shown. Aniline plane is defined via C(15), C(16), C(17), C(18), C(19), C(20) atoms, pyridine plane – N(1), C(2), C(3), C(4), C(5), C(6). Q is the centroid of the aniline ring.

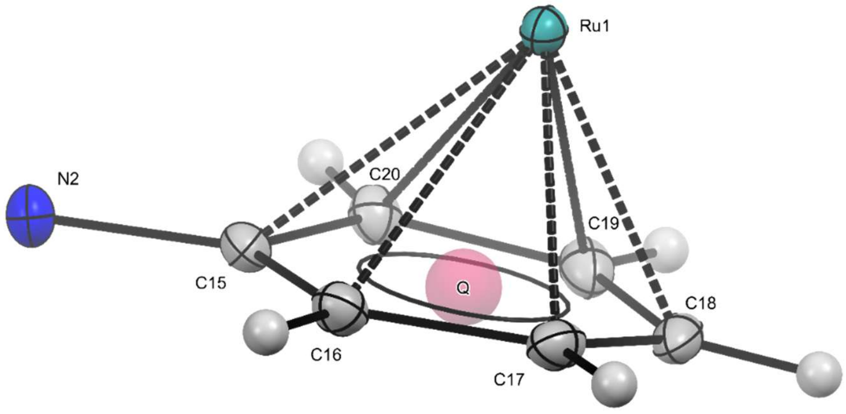

Figure S168. The molecular structure of compound **4** (ellipsoids drawn at the 30% probability level). Only the metal center with the coordinated $PhN(TMS)_2$ is shown. The $SiMe_3$ groups are omitted for clarity. Q is the centroid of the aniline ring.

Distance	Å	Distance	Å
Ru(1)-C(15)	2.4275(13)	Ru(1)-N(1)	2.0961(11)
Ru(1)-C(16)	2.3775(14)	Ru(1)-H(1)	1.56(2)
Ru(1)-C(17)	2.2091(14)	Ru(1)-P(1)	2.3325(3)
Ru(1)-C(18)	2.2055(14)	P(1)-C(1)	1.7561(15)
Ru(1)-C(19)	2.1776(14)	C(1)-C(2)	1.382(2)
Ru(1)-C(20)	2.2409(13)	C(2)-N(1)	1.3910(18)
Ru(1)-Q	1.7821(7)	C(2)-C(3)	1.4415(19)
C(15)-$Aniline_{plane}$	0.0375(14)	C(3)-C(4)	1.359(2)
C(16)-$Aniline_{plane}$	0.0099(16)	C(4)-C(5)	1.407(2)
C(17)-$Aniline_{plane}$	0.0493(16)	C(5)-C(6)	1.370(2)
C(18)-$Aniline_{plane}$	0.0405(16)	N(1)-C(6)	1.3555(18)
C(19)-$Aniline_{plane}$	0.0076(16)	C(15)-C(16)	1.4054(19)
C(20)-$Aniline_{plane}$	0.0463(15)	C(16)-C(17)	1.4229(19)
Ru ring slippage*	0.194	C(17)-C(18)	1.408(2)
Ru(1)-$Pyridine_{plane}$	0.1287(3)	C(18)-C(19)	1.406(2)
N(2)-$Aniline_{plane}$	0.1449(13)	C(19)-C(20)	1.4174(19)
		C(20)-C(15)	1.4312(19)

Table S7. Selected distances in the X-ray crystal structure of **4**. *Distance between the perpendicular projection of Ru on the aniline ring L.S.-plane and ring centroid.

Angle	°	Angle	°
Ru(1)-Q-C(15)	97.21(6)	N(1)-Ru(1)-P(1)	81.75(3)
Ru(1)-Q-C(16)	95.51(7)	N(1)-Ru(1)-H(1)	80.7(7)
Ru(1)-Q-C(17)	87.31(7)	N(1)-Ru(1)-Q	131.28(4)
Ru(1)-Q-C(18)	85.91(7)	P(1)-Ru(1)-Q	140.17(3)
Ru(1)-Q-C(19)	85.20(7)	P(1)-Ru(1)-H(1)	75.4(7)
Ru(1)-Q-C(20)	88.74(7)	C(1)-P(1)-Ru(1)	101.08(5)
(Ru(1)-Q)-(normal of $Aniline_{plane}$)	6.25(5)	H(1)-Ru(1)-Q	124.7(8)
N(1)-C(2)-C(1)-P(1)	2.10(19)	P(1)-Ru(1)-Q-C15	125.77(7)

Table S8. Selected angles and torsion angles in the X-ray crystal structure of **4**.

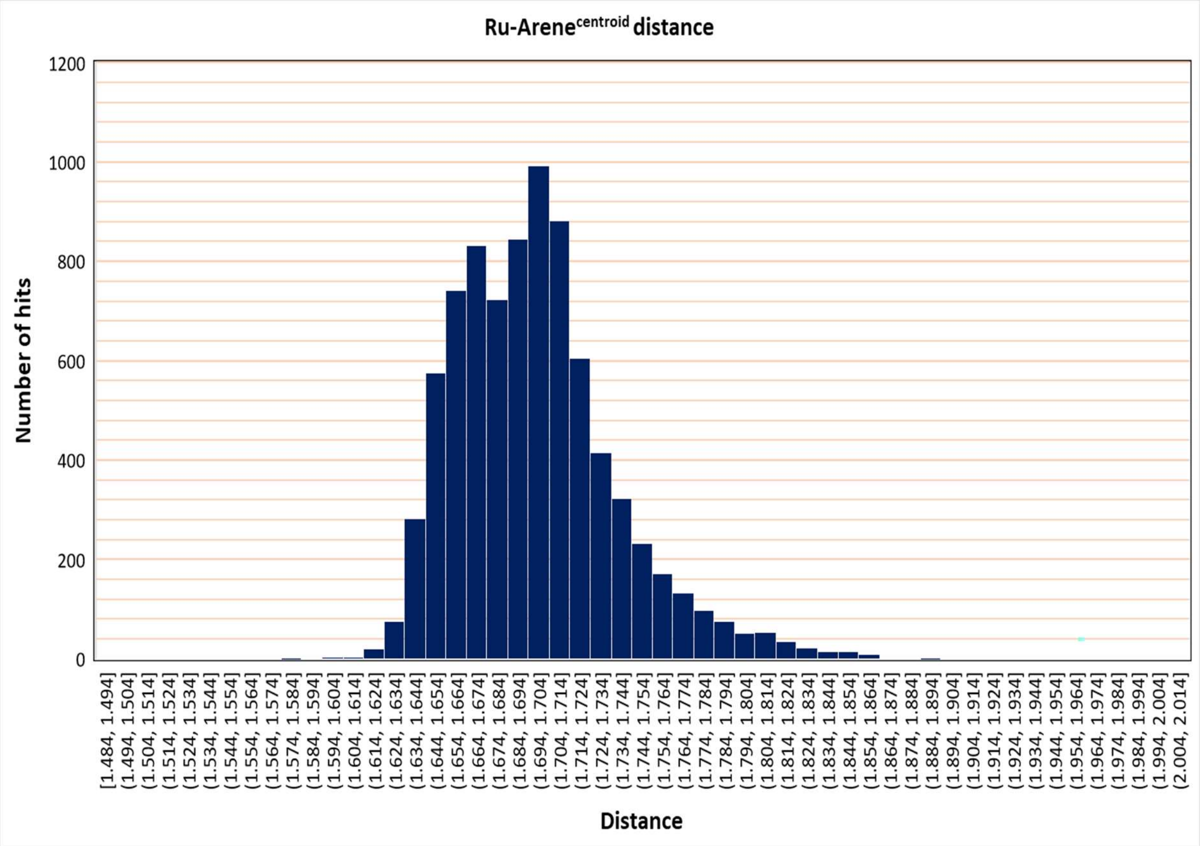

Figure S169. The histogram of a search in the Cambridge Structure Database (as of October 2023)[41] shows that only 4% of structures with η^6-arene-Ru bonds* have longer Ru-Arene^{Centroid} distances than 1.78 Å. It can be explained by very bulky $N(TMS)_2$ substituent in the case of complex **4**.

**In the ConQuest search the η^6 coordination type was shown as 6 "any" bonds between Ru and all 6 carbon atoms of the arene (with any substituents in the ring). The Arene^{Centroid} was found by the standard tools available in the software package. The distance between Ru and the Arene^{Centroid} was defined from 0 to 5 Å.*

S6.3 X-ray crystal structure determination of **(^{tBu}PN)$_2$RuCl$_2$**

$C_{28}H_{48}Cl_2N_2P_2Ru$, Fw = 646.59, orange block, 0.28 · 0.26 · 0.21 mm^3, monoclinic, P2$_1$/c (no. 14), a = 17.0703(3), b = 22.3344(5), c = 16.3747(4) Å, β = 98.020(1)°, V = 6181.8(2) Å^3, Z = 8, D_x = 1.389 g/cm^3, μ = 0.80 mm^{-1}. The diffraction experiment was performed on a Bruker Kappa ApexII diffractometer with a sealed tube and Triumph monochromator (λ = 0.71073 Å) at a temperature of 150(2) K up to a resolution of $(\sin \theta/\lambda)_{max}$ = 0.70 Å^{-1}. Intensity integration was performed using the Eval15 software[36]. A multi-scan absorption correction and scaling were performed with SADABS[37] (correction range 0.68-0.75). A total of 198737 reflections were measured, 18016 reflections were unique (R_{int} = 0.048), and 15032 reflections were observed [I>2σ(I)]. The structure was solved with Patterson superposition methods using SHELXT.[38] Structure refinement was performed with SHELXL-2018[39] on F^2 of all reflections. Non-hydrogen atoms were refined freely with anisotropic displacement parameters. Hydrogen atoms were introduced in calculated positions and refined with a riding model. 655 Parameters were refined with no restraints. R1/wR2 [I > 2σ(I)]: 0.0230 / 0.0542. R1/wR2 [all refl.]: 0.0317 / 0.0566. S = 1.035. Residual electron density between –0.37 and 0.46 e/Å^3. Geometry calculations and checking for higher symmetry were performed with the PLATON program.[40]

The structure is similar to the previously reported one with ^{iPr}PN ligand[42] and shows similar geometric parameters (see Table S9). The comparison of the distances and angles in both structures shows that they are very close. Noteworthy, the structures were obtained at different temperatures that can affect the geometric parameters. One of the most distinct differences lies in significantly elongated Ru-P bonds for the complex with ^{tBu}PN ligand (2.3713(5) – 2.3857(5) Å) compared to the one with ^{iPr}PN ligand (2.310(2) – 2.312(2) Å). Another striking structural feature of ***trans*-(^{tBu}PN)$_2$RuCl$_2$** is the almost ideal position of N11-N12-Ru1-P1-P2 in the same plane (the biggest out-of-plane distance was found for N atoms: 0.035 – 0.046 Å). Since the *i*Pr groups induce less steric hindrance, it is possible to twist two ^{iPr}PN ligands in ***trans*-(^{iPr}PN)$_2$RuCl$_2$** relating to each other. It imposes big out-of-plane positions for the N and P atoms (0.130 – 0.131 and 0.162 – 0.165 Å, respectively)

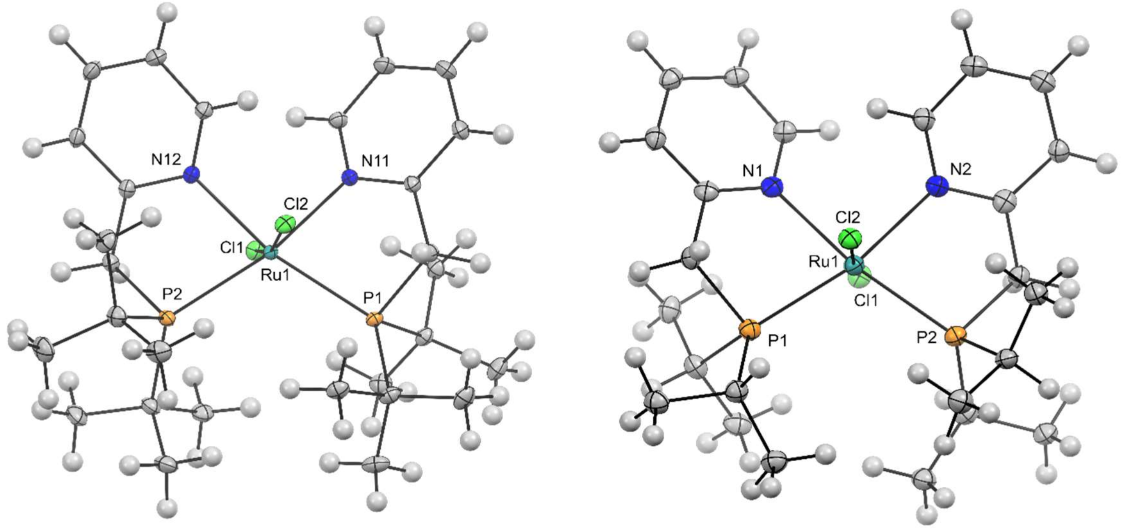

Figure S170. The molecular structure of compounds ***trans*-(^{tBu}PN)$_2$RuCl$_2$** (*left*, this work) and ***trans*-(^{iPr}PN)$_2$RuCl$_2$** (*right*, R. Langer et al.)[42] (ellipsoids drawn at the 30% probability level). Hydrogens are drawn as fixed-size spheres of 0.2 Å radius. For ***trans*-(^{tBu}PN)$_2$RuCl$_2$** only one symmetry-independent molecule is depicted.

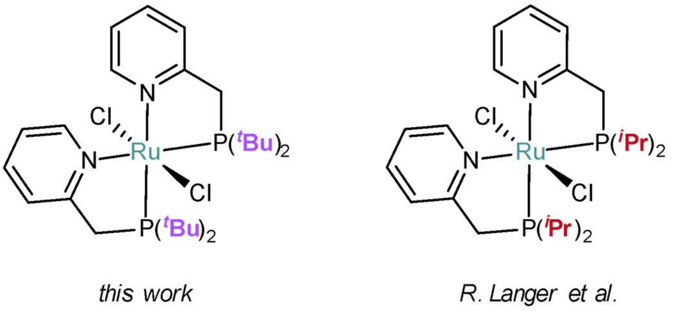

Figure S171. The schematic representations of ***trans*-(^{tBu}PN)$_2$RuCl$_2$** (*left*, this work) and ***trans*-(^{iPr}PN)$_2$RuCl$_2$** (*right*, R. Langer et al.).[42]

**Crystallographic details**	***Trans*-(^{tBu}PN)$_2$RuCl$_2$** (this work)	***Trans*-(^{iPr}PN)$_2$RuCl$_2$** (R. Langer et al.)[42]
Space group	Monoclinic, P2$_1$/c	Triclinic, P -1
Z; Z’	8; 2	2; 1
T, K	150	100
**Distance, Å**		
Ru-N	2.124(1) – 2.146(1)	2.154(4) – 2.160(5)
Ru-Cl	2.4204(6) – 2.4361(5)	2.432(2) – 2.442(2)
Ru-P	2.3713(5) – 2.3857(5)	2.310(2) – 2.312(2)
(N-N-Ru-P-P)^{plane}-Ru	0.009 – 0.010	0.001
(N-N-Ru-P-P)^{plane}-P	0.026 – 0.037	0.130 – 0.131
(N-N-Ru-P-P)^{plane}-N	0.035 – 0.046	0.162 – 0.165
**Angle, °**		
Py-Py planes angle	35.69 – 36.28	40.55
Cl-Ru-Cl	165.74(2) – 165.99(2)	172.93(5)
P-Ru-P	112.80(2) – 113.49(2)	111.64(5)
N-Ru-N	90.74(5) – 91.69(5)	90.7(2)

Table S9. Comparison table of selected distances and angles in the X-ray crystal structures of ***trans*-(^{tBu}PN)$_2$RuCl$_2$** (this work) and ***trans*-(^{iPr}PN)$_2$RuCl$_2$** (R. Langer et al.).[42]

CCDC 2355856-2355858 contain the supplementary crystallographic data for this paper. These data can be obtained free of charge from The Cambridge Crystallographic Data Centre via http://www.ccdc.cam.ac.uk/data_request/cif.

S8 References

1. Burrows, J., Kamo, S., Koide, K. Scalable Birch reduction with lithium and ethylenediamine in tetrahydrofuran. Science, 2021, 374, 741–746.
2. Goldsmith, C. R., Jonas, R. T., Stack, T. D. P. C—H Bond Activation by a Ferric Methoxide Complex: Modeling the Rate-Determining Step in the Mechanism of Lipoxygenase. J. Am. Chem. Soc., 2002, 124, 83–96.
3. Cowley, R. E. et al. Selectivity and Mechanism of Hydrogen Atom Transfer by an Isolable Imidoiron(III) Complex. J. Am. Chem. Soc., 2011, 133, 9796–9811.
4. A. Bennett, M., K. Smith, A. Arene ruthenium(II) complexes formed by dehydrogenation of cyclohexadienes with ruthenium(III) trichloride. J. Chem. Soc. Dalton Trans., 1994, 0, 233–241.
5. Parr, R. G., Weitao, Y. Density-Functional Theory of Atoms and Molecules. Oxford, Oxford University Press, 1994.
6. Neese, F. The ORCA program system. WIREs Comput. Mol. Sci., 2012, 2, 73–78.
7. Neese, F. Software update: the ORCA program system, version 4.0. WIREs Comput. Mol. Sci., 2018, 8, e1327.
8. Neese, F. Software update: The ORCA program system—Version 5.0. WIREs Comput. Mol. Sci., 2022, 12, e1606.
9. Becke, A. D. A new mixing of Hartree–Fock and local density-functional theories. J. Chem. Phys., 1993, 98, 1372–1377.
10. Lee, C., Yang, W., Parr, R. G. Development of the Colle-Salvetti correlation-energy formula into a functional of the electron density. Phys. Rev. B Condens. Matter, 1988, 37, 785–789.
11. Grimme, S., Antony, J., Ehrlich, S., Krieg, H. A consistent and accurate ab initio parametrization of density functional dispersion correction (DFT-D) for the 94 elements H-Pu. J. Chem. Phys., 2010, 132, 154104.
12. Grimme, S., Ehrlich, S., Goerigk, L. Effect of the damping function in dispersion corrected density functional theory. J. Comput. Chem., 2011, 32, 1456–1465.
13. Slater, J. C., Phillips, J. C. Quantum Theory of Molecules and Solids Vol. 4: The Self-Consistent Field for Molecules and Solids. Phys. Today, 1994, 27, 49–50.
14. Vosko, S. H., Wilk, L., Nusair, M. Accurate spin-dependent electron liquid correlation energies for local spin density calculations: a critical analysis. Can. J. Phys., 1980, 58, 1200–1211.
15. Becke, A. D. Density-functional exchange-energy approximation with correct asymptotic behavior. Phys. Rev. A, 1988, 38, 3098–3100.
16. Becke, A. D. Density-functional thermochemistry. III. The role of exact exchange. J. Chem. Phys., 1993, 98, 5648–5652.
17. Weigend, F., Ahlrichs, R. Balanced basis sets of split valence, triple zeta valence and quadruple zeta valence quality for H to Rn: Design and assessment of accuracy. Phys. Chem. Chem. Phys., 2005, 7, 3297–3305.
18. Andrae, D., Häußermann, U., Dolg, M., Stoll, H., Preuß, H. Energy-adjustedab initio pseudopotentials for the second and third row transition elements. Theor. Chim. Acta, 1990, 77, 123–141.
19. Neese, F. An improvement of the resolution of the identity approximation for the formation of the Coulomb matrix. J. Comput. Chem., 2003, 24, 1740–1747.
20. Helmich-Paris, B., De Souza, B., Neese, F., Izsák, R. An improved chain of spheres for exchange algorithm. J. Chem. Phys., 2001, 155, 104109.
21. Weigend, F. Accurate Coulomb-fitting basis sets for H to Rn. Phys. Chem. Chem. Phys., 2006, 8, 1057–1065.
22. Marenich, A. V., Cramer, C. J., Truhlar, D. G. Universal Solvation Model Based on Solute Electron Density and on a Continuum Model of the Solvent Defined by the Bulk Dielectric Constant and Atomic Surface Tensions. J. Phys. Chem. B, 2009, 113, 6378–6396.

23. Epifanovsky, E. et al. Software for the frontiers of quantum chemistry: An overview of developments in the Q-Chem 5 package. J. Chem. Phys., 2021, 155, 084801.
24. Foresman, J. B., Head-Gordon, M., Pople, J. A., Frisch, M. J. Toward a systematic molecular orbital theory for excited states. J. Phys. Chem., 1992, 96, 135–149.
25. Hirata, S., Head-Gordon, M. Time-dependent density functional theory within the Tamm–Dancoff approximation. Chem. Phys. Lett., 1999, 314, 291–299.
26. Elsemongy, M.M., Abdel-Khalek, A. A. Gibbs energies of transfer of individual ions from water into tetrahydrofuran and its aqueous mixtures. Thermochim. Acta, 1991, 181, 79–94.
27. Tissandier MD, Cowen KA, Feng WY, Gundlach E, Cohen MH, Earhart AD, et al. The Proton's Absolute Aqueous Enthalpy and Gibbs Free Energy of Solvation from Cluster-Ion Solvation Data. J Phys Chem A. 1998 Oct 1;102(40):7787–94.
28. Yang, W., Mortier, W. J. The use of global and local molecular parameters for the analysis of the gas-phase basicity of amines. J. Am. Chem. Soc., 1986, 108, 5708–5711.
29. Bultinck, P., Fias, S., Van Alsenoy, C., Ayers, P. W., Carbó-Dorca, R. Critical thoughts on computing atom condensed Fukui functions. J. Chem. Phys., 2007, 127, 034102.
30. Hirshfeld, F. L. Bonded-atom fragments for describing molecular charge densities. Theor. Chim. Acta, 1977, 44, 129–138.
31. Humphrey, W., Dalke, A., Schulten, K. VMD: Visual molecular dynamics. J. Mol. Graph., 1996, 14, 33–38.
32. Beek, C. B. van, Leest N.P. van, Lutz M.,de Vos S.D.,Klein Gebbink R.J.M., de Bruin B., Broere D.L.J.. Combining metal–metal cooperativity, metal–ligand cooperativity and chemical non-innocence in diiron carbonyl complexes. Chem. Sci., 2022, 13, 2094–2104.
33. Shvydkiy, N. V., Perekalin, D. S. Reactions of arene replacement in transition metal complexes. Coord. Chem. Rev., 2020, 411, 213238.
34. Kharasch, M. S., Fields, E. K. Factors Determining the Course and Mechanisms of Grignard Reactions. IV. The Effect of Metallic Halides on the Reaction of Aryl Grignard Reagents and Organic Halides1. J. Am. Chem. Soc., 1941, 63, 2316–2320.
35. Seyferth, D. The Grignard Reagents. Organometallics 28, 1598–1605 (2009).
36. Schreurs, A. M., Xian, X., Kroon-Batenburg, L. M. EVAL15: a diffraction data integration method based on ab initio predicted profiles. J. Appl. Crystallogr., 2010, 43, 70–82.
37. Krause, L., Herbst-Irmer, R., Sheldrick, G. M., Stalke, D. Comparison of silver and molybdenum microfocus X-ray sources for single-crystal structure determination. J. Appl. Crystallogr., 2015, 48, 3–10.
38. Sheldrick, G. M. SHELXT–Integrated space-group and crystal-structure determination. Acta Crystallogr. Sect. Found. Adv., 2015, 71, 3–8.
39. Sheldrick, G. M. Crystal structure refinement with SHELXL. Acta Crystallogr. Sect. C Struct. Chem. 2015, 71, 3–8.
40. Spek, A. L. Structure validation in chemical crystallography. Acta Crystallogr. D Biol. Crystallogr., 2009, 65, 148–155.
41. Groom, C. R., Bruno, I. J., Lightfoot, M. P., Ward, S. C. The Cambridge Structural Database. Acta Crystallogr. Sect. B Struct. Sci. Cryst. Eng. Mater., 2016, 72, 171–179.
42. Langer, R., Gese, A., Gesevičius, D., Jost, M., Langer, B.R., Schneck, F., Venker, A. and Xu, W. Formation of Different Isomers of Phosphine–Imidazolyl and –Pyridyl Ruthenium(II) Complexes Affecting the Catalyst Activity in the Acceptorless Dehydrogenation of Alcohols. Eur. J. Inorg. Chem. 2015, 696–705.
